# Photo-mediated selective deconstructive geminal dihalogenation of trisubstituted alkenes

**DOI:** 10.1038/s41467-020-18274-2

**Published:** 2020-09-08

**Authors:** Han Wang, Ren Wei Toh, Xiangcheng Shi, Tonglin Wang, Xu Cong, Jie Wu

**Affiliations:** 1 Department of Chemistry, National University of Singapore, 3 Science Drive 3, Singapore, 117543 Republic of Singapore; 2 College of Chemistry and Chemical Engineering, Northwest Normal University, 730070 Lanzhou, Gansu China; 3 National University of Singapore (Suzhou) Research Institute, 377 Lin Quan Street, Suzhou Industrial Park, 215123 Suzhou, Jiangsu China

**Keywords:** Photocatalysis, Synthetic chemistry methodology, Synthetic chemistry methodology

## Abstract

Selective deconstructive functionalization of alkenes, other than the well-established olefin metathesis and ozonolysis, to produce densely functionalized molecular scaffolds is highly attractive but challenging. Here we report an efficient photo-mediated deconstructive germinal dihalogenation of carbon-carbon double bonds. A wide range of geminal diiodoalkanes and bromo(iodo)alkanes (>40 examples) are directly prepared from various trisubstituted alkenes, including both cyclic and acyclic olefins. This C=C cleavage is highly chemoselective and produces geminal dihalide ketones in good yields. Mechanistic investigations suggest a formation of alkyl hypoiodites from benzyl alcohols and *N*-iodoimides, which undergo light-induced homolytic cleavage to generate active oxygen radical species.

## Abstract (web summary)

Efficient synthetic pathways to geminal dihalides are quite limited, despite their versatility as chemical building blocks. Here, the authors report a photo-mediated deconstructive fragmentation of cyclic and acyclic trisubstituted alkenes to access a variety of geminal dihalides.

## Introduction

In organic synthesis, common functionalization usually focuses on the installation or modification of functional groups without significantly changing the backbones of molecules. In stark contrast, deconstructive functionalization is attractive as it can drastically change the scaffolds of molecules to introduce new chemical space, unmask dormant functional groups, and create functionalities tethered at a predefined distance determined by ring sizes of the reactants.

**Fig. 1:**
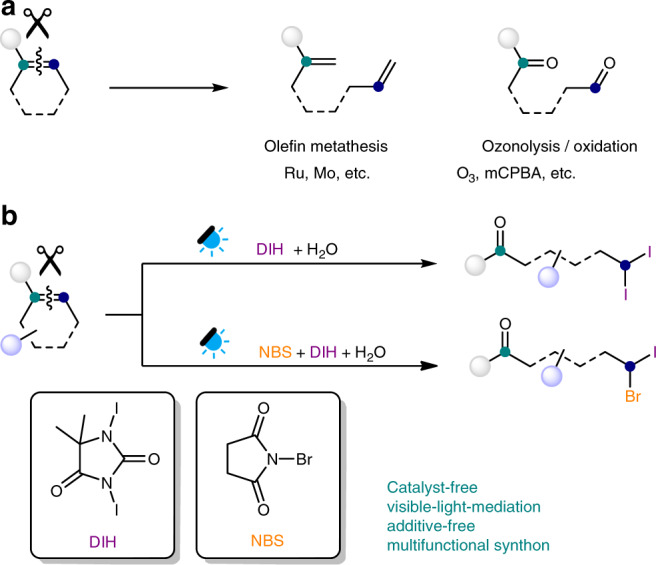
Deconstructive functionalization of alkenes. **a** Common strategies for C=C bond cleavage. **b** Visible-light-mediated deconstructive oxidative geminal dihalogenation of trisubstituted alkenes (this work). DIH 1,3-diiodo-5,5-dimethyl-hydantoin, NBS *N*-bromosuccinimide, mCPBA *meta*-chloroperoxybenzoic acid.

The carbon-carbon double bond is one of the most fundamental functionalities in organic molecules. Various methods have been developed to convert alkenes to important intermediates and fine chemical products, which play vital roles in the fields of material science, biochemistry, pharmaceutical science, and the chemical industry^1–8^. Deconstructive functionalization of alkenes has been well developed to introduce two functional groups at different sites of olefins (Fig. 1a). For instance, transition-metal-catalyzed C=C bond cleavage processes, such as olefin metathesis, have found wide application in natural product and material synthesis^9–12^. Ozonolysis and other similar oxidations with various organic and inorganic oxidants were robust to introduce two carbonyl derivatives from a single C=C bond^13–17^. Aside from such well-established strategies, other types of deconstructive functionalization of alkenes producing densely functionalized scaffolds remain rare and challenging^18–27^.

Organohalides are versatile building blocks in synthetic chemistry. They are widely utilized as precursors in transition-metal-catalyzed cross-coupling, radical reactions, nucleophilic substitutions, and metal-halide exchanges^28–30^. Among them, geminal dihalides represent a unique class of compounds and have been used as carbene precursors and multi-functional synthons. However, efficient synthetic pathways to synthesize geminal dihalides are quite limited, which significantly restricts the investigation and wide application of this unique family of compounds^31–35^. Herein, we report a direct synthetic route to geminal dihalides by photo-mediated deconstructive fragmentation of cyclic or acyclic trisubstituted alkenes (Fig. 1b).

## Results

### Reaction optimization

We initially designed a cascade hydrohalogenation of trisubstituted alkenes and subsequent photo-induced *β*-scission of the generated alcohol intermediates. Based on previous reports from groups of Chen and Zhu on photo-mediated conversion of alcohols to oxygen radicals^36–42^, our study commenced by using 1-phenyl-1-cyclohexene (**1**) as a model substrate. As illustrated in Table 1, treatment of **1** with eosin Y (1 mol%) as the photocatalyst, *N*-iodosuccinimide (NIS, 4 equiv), H_2_O (50 equiv) and acetoxyl benziodoxole (BIOAc, 2 equiv) in MeCN under blue light-emitting diode (LED) irradiation, the desired product, 6,6-diiodo-1-phenylhexan-1-one (**2**) was obtained in 40% yield (entry 1). Further investigation revealed that a similar result could be obtained in the absence of any photocatalyst (entry 2). To our surprise, **2** could be generated in 28% yield even without BIOAc (entry 3). A moderate temperature (50 °C) significantly accelerated the reaction (entries 4–5). Evaluation of solvents indicated that a mixed solvent of EtOAc/MeNO_2_ (10:1) was the optimal choice, leading to the generation of **2** in 81% yield (entries 6–9). Using 1,3-diiodo-5,5-dimethyl-hydantoin (DIH) as the iodination agent afforded an improved yield compared to that with NIS (entry 10). Light irradiation was essential as no product **2** could be detected in the absence of light (entry 11).

**Table 1:** Optimization of oxidative deconstructive geminal diiodination^a^.


entry	catalyst	solvent	additive (x equiv)	yield (%)^b^
1	eosin Y	MeCN	BIOAc (2)	40
2	–	MeCN	BIOAc (2)	41
3	–	MeCN	–	28
4^c^	–	MeCN	–	60
5^d^	–	MeCN	–	41
6^c^	–	DCE	–	34
7^c^	–	EtOAc	–	79
8^c^	–	acetone	–	n.d.
9^*c*^	–	EtOAc/MeNO_2_ (10:1)	–	81
10^c,e^	–	EtOAc/MeNO_2_ (10:1)	–	91(84)^f^
11^c,e,g^	–	EtOAc/MeNO_2_ (10:1)	–	n.d.

*NIS* N-iodosuccinimide, *DCE* 1,2-dichloroethane, *n.d.* not determined.

^a^Standard conditions: **1** (0.2 mmol), NIS (0.8 mmol), and H_2_O (10 mmol) in solvent (0.1 M), irradiated under blue LED lamps (80 W) for 36 h at 30 °C.

^b^Yields determined by analysis of the crude ^1^H-NMR spectra using dibromomethane as an internal standard.

^c^Reaction was performed at 50 °C.

^d^Reaction was performed at 80 °C.

^e^DIH (0.4 mmol, 2 equiv) was used instead of NIS.

^f^Isolated yields.

^g^No light irradiation.

### Substrate scope

**Fig. 2:**
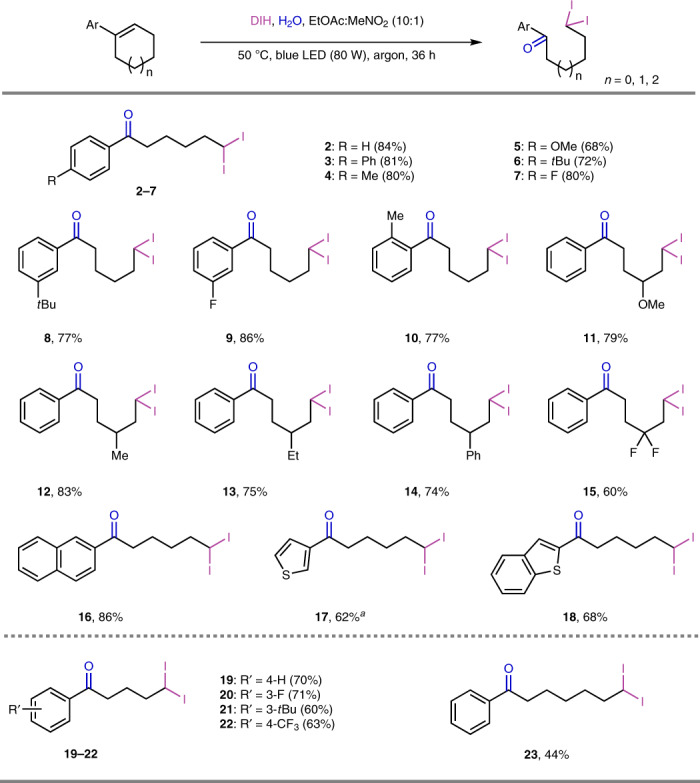
Scope of oxidative deconstructive geminal diiodination of cyclic alkenes. Isolated yields unless otherwise indicated. Performed with alkene (0.2 mmol), H_2_O (10 mmol), DIH (0.4 mmol) in EtOAc:MeNO_2_ (10:1), irradiated under blue LED lamps (80 W) for 36 h at 50 °C. ^*a*^4 equiv NIS instead of DIH.

With the optimized conditions established, we investigated the scope of the deconstructive geminal diiodination of cyclic alkenes. As shown in Fig. 2, a variety of aryl-substituted cyclohexene derivatives underwent deconstructive geminal diiodination effectively to deliver products **2**–**15** in moderate to good yields. Cyclohexenes containing aryl rings with electronically distinct substituents in the *ortho*-, *meta*- or *para*-position afforded products **2**–**10** in similar yields, with the exception of a compound bearing a strong electron-donating methoxy substituent, with which electrophilic aryl iodination occurred in the presence of DIH, resulting in a lower yield of product **5**. Various substituents on the cyclohexene ring, including methoxy, alkyl, phenyl, difluoro, were well tolerated, giving products **11**–**15** with similar yields. Naphthyl and hetero-aryl-substituted cyclohexenes were also suitable substrates, and underwent ring-opening and geminal diiodination to deliver compounds **16**–**18** with yields of 62–86%. Because the thiophene ring is very reactive towards electrophilic substitution, NIS was applied in place of DIH to avoid the iodination of the thiophene ring in the synthesis of **17**. The deconstructive geminal diiodination is not limited to cyclohexene derivatives; a range of aryl-substituted cyclopentene and cycloheptene derivatives were all viable in this deconstructive functionalization to achieve products **19**–**23** in moderate to good yields. Variation of the size of the ring containing the double bond led to geminal diiodide ketone products bearing carbon chains with varying lengths.

**Fig. 3:**
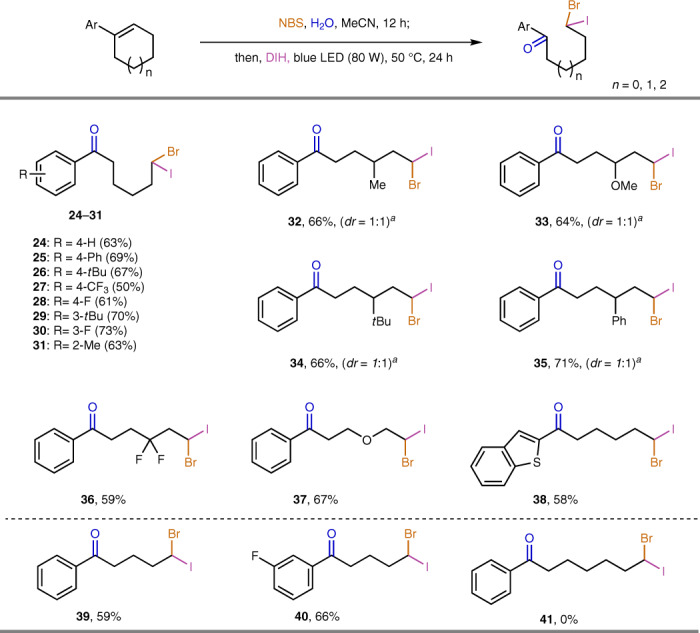
Scope of oxidative deconstructive geminal bromo-iodination of cyclic alkenes. Isolated yields unless otherwise indicated. Performed with alkene (0.2 mmol), NBS (0.21 mmol), H_2_O (10 mmol), DIH (0.3 mmol) in MeCN. See Supplementary Information for experimental details. ^*a*^*Dr* values were determined by analysis of ^1^H NMR spectra of the crude product mixture.

Besides the use of DIH to generate geminal diiodo products, this strategy can be further expanded to produce potentially more useful bromo(iodo)alkanes by modifying the optimal reaction conditions by addition of NBS (Fig. 3). This cascade transformation was applied to a variety of aryl-substituted cyclic alkenes to afford the corresponding geminal bromo-iodination products. A variety of functional groups on the aryl rings or the cyclohexene scaffolds were well tolerated and delivered products **24**–**36** in moderate to good yields. 3,6-Dihydropyran was a good substrate for this cascade reaction thus further enriching the skeletal diversification (**37**). The robustness of this strategy was further explored with heteroaryl rings and cyclopentenes which generated the corresponding bromo-iodide arylketone products **38**–**40** in moderate yields. However, 1-phenyl-1-cycloheptene did not afford the corresponding bromo(iodo)alkane product **41** under our reaction conditions.

**Fig. 4:**
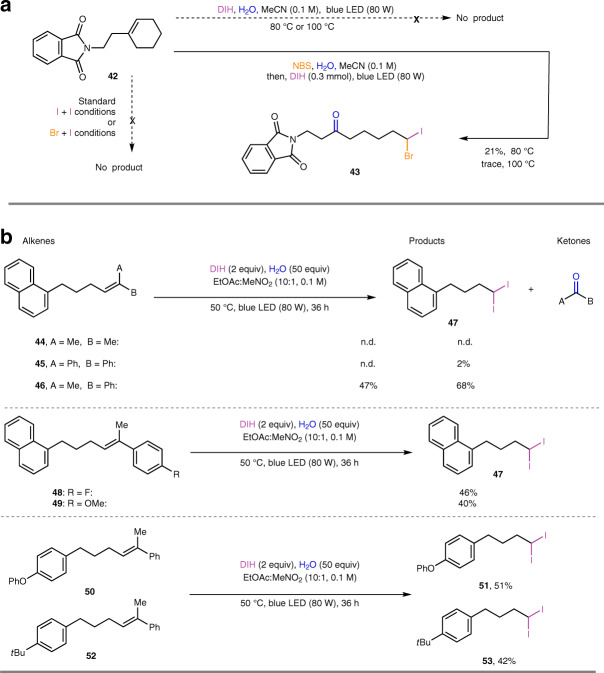
Investigation of other trisubstituted alkenes. **a** Trialkyl-substituted cyclohexenes. **b** Acyclic trisubstituted alkenes.

As illustrated in Fig. 4a, trialkyl-substituted alkene **42** was subjected to the standard conditions for geminal diiodination and bromo-iodination, but no corresponding product could be generated. A high temperature (80 °C) could not promote geminal diiodination of **42** either, while bromo-iodination product **43** could be isolated in 21% yield. However, further increasing the temperature to 100 °C afforded only a trace amount of **43**. Control experiments (Supplementary Fig. 2) indicated the instability of halohydrins at high temperatures and the ineffectiveness of trialkyl-substituted halohydrins in the deconstructive iodination step. These results highlighted the importance of a suitable temperature and aromatic substituents for the success of the deconstructive geminal dihalogenation. To further expand the scope of this reaction, acyclic trisubstituted olefins were evaluated. As shown in Fig. 4b, acyclic alkenes **44**–**46** with different substituent patterns were subjected to the optimal diiodination conditions, and only the gem-alkyl-aryl-substituted olefin **46** delivered the corresponding diiodide product **47** in a useful yield (47%). Further tuning of the electronic properties in the vinyl aryl substituent (**48** and **49**) resulted in product **47** with similar yields. The deconstructive geminal diiodination proceeded readily with alkenes bearing different remote functionalities (**50** and **52**), affording products **51** and **53**, respectively, in moderate yields.

### Mechanistic elucidation with supporting evidence

**Fig. 5:**
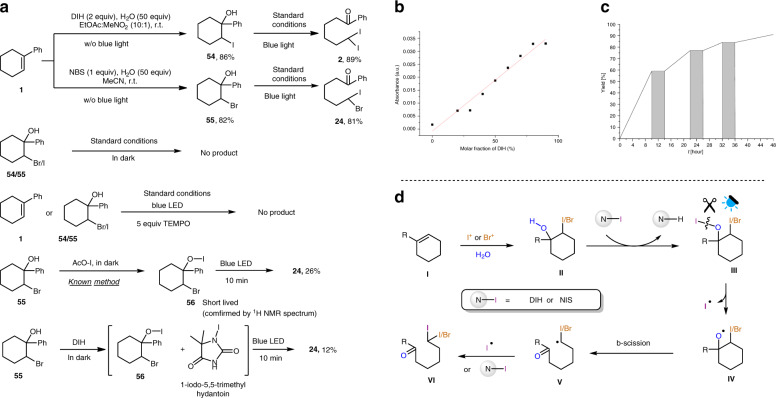
Plausible mechanism with supporting evidence. **a** Control experiments. **b** UV-Vis measurements of stoichiometry between **55** and DIH @ 450 nm. **c** Light on-off experiments of deconstructive geminal diiodination of **1**. **d** Plausible mechanism.

Various control experiments were performed to elucidate the reaction mechanism. First, **1** was treated with DIH or NBS and water in the absence of light to afford iodohydrin **54** or bromohydrin **55**, respectively. Tertiary alcohols **54** and **55** were subjected to the standard light-promoted conditions and afforded products **2** and **24**, respectively, but gave no product in the absence of light (Fig. 5a). These control experiments showed that the iodohydrin and bromohydrin were intermediates in the cascade one-pot reaction. When TEMPO was added into the reaction mixture, no dihalide product could be detected, which supported a radical based reaction mechanism. ESR measurements also indicated the presence of radical species when reacting DIH with **55** in MeCN under light irradiation (Supplementary Figs. 9 and 10).

Based on previous literatures^43^, alcohols can react with acetyl hypoiodite (AcOI) to generate alkyl hypoiodites. The O-I bonds of the alkyl hypoiodites could undergo homolytic cleavage under light irradiation to deliver a transient alkoxy radical species. We, therefore, prepared **56** by treating **55** with acetyl hypoiodite in dark, and generation of the unstable intermediate **56** was confirmed by ^1^H NMR analysis of the reaction mixture (Supplementary Fig. 3). Irradiation of **56** formed in situ with blue LED lamps for 10 min afforded product **24** in 26% yield. When a mixture of **55** and DIH in MeCN, reacting in the dark, was examined by ^1^H NMR and GC-MS analysis, **56** and 1-iodo-5,5-dimethylhydantoin were detected (Supplementary Fig. 4–6). This result indicates the generation of an alkyl hypoiodite from a phenyl-substituted alcohol and DIH. Even though we cannot fully exclude the possibility of formation of an electron donor-acceptor (EDA) complex between **55** and DIH, UV-Vis measurements of different stoichiometry between **55** and DIH did not follow the curve of Job’s plot, which is normally observed for an EDA complex (Fig. 5b). Light on/off experiments indicated that a long radical chain process is unlikely. As illustrated in Fig. 5c, the product formation ceased immediately when the light source was periodically switched off and resumed when light was turned on.

Based on existing related literatures^43–47^ and all the experimental results described above, a plausible mechanism was proposed and is described in Fig. 5d. The cascade reaction is initiated by hydroxyhalogenation of alkene **I** in the presence of water and an electrophilic halogen source^44^. The resulting halohydrin **II** reacts with DIH to deliver the alkyl hypoiodite intermediate **III**. Light irradiation induces the homolysis of the labile I–O bond to generate the reactive oxygen radical species **IV**^43,45,46^. Carbon radical **V** is formed by β-scission of radical **IV** and subsequent iodination with the iodine radical or DIH accomplishes product **VI**^43,46–51^.

**Fig. 6:**
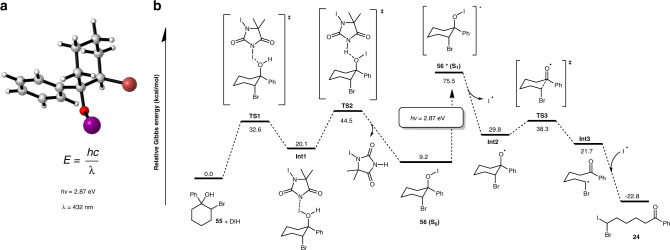
DFT calculations for the ring-opening iodination of 55 with DIH. **a** Optimized geometry of **55** based on DFT calculation. **b** Free energy profiles for the ring-opening iodination.

To further support the proposed ring-opening iodination, density functional theory (DFT) calculations were conducted on the model reaction of **55** with DIH. The resulting energy profiles of the reaction process are displayed in Fig. 6. The complexation of **55** and DIH leads to a zwitterionic intermediate **Int1** through **TS1** with active free energy of 32.6 kcal/mol. This relatively high active free energy may explain why an elevated temperature is essential for an efficient transformation (Table 1, entry 3 vs 4). The iodine atom between the two carbonyl groups is more electrophilic than the other iodine atom in DIH, and undergoes complexation with alcohol **55**. The subsequent heterolysis of the O-I bond occurs together with the alcohol deprotonation through a transition state **TS2** to give **56** and dimethyliodohydantoin with a barrier of 24.4 kcal/mol relative to **Int1**. Under blue light excitation, the ground-state **56 (S**_**0**_**)** is pumped to the first singlet excited-state 56* (S_**1**_**)** with excitation energy of 66.3 kcal/mol (2.87 eV) according to time-dependent density functional theory (TD-DFT) calculations. Homolytic cleavage of the O-I bond in 56* produces the active oxygen radical **Int2**. The subsequent *β*-scission is a facile process with a barrier of 8.5 kcal/mol to give the transient carbon radical **Int3**, followed by exergonic trapping with the iodine radical to accomplish the generation of product **24**. The geometry of the unstable alkyl hypoiodite **56** was optimized and the calculated maximum light absorption is at 432 nm (Fig. 6a), which is close to the blue LED maximum emission (456 nm) used in this study.

### Further synthetic applications

**Fig. 7:**
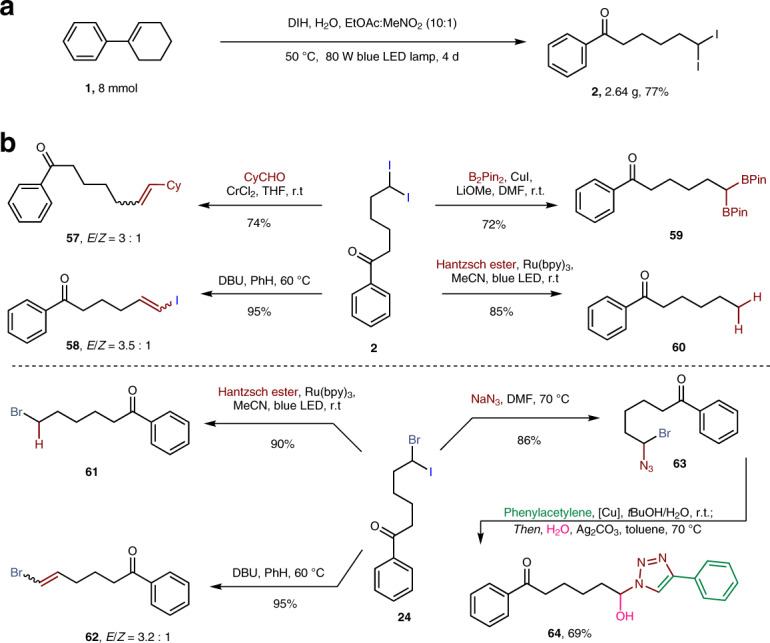
Gram-scale synthesis and further synthetic diversification. **a** Gram-scale synthesis. **b** Synthetic diversification. B_2_Pin_2_ bis (pinacolato)diboron, DBU 1,8-diazabicyclo (5.4.0)undec- 7-ene.

The oxidative geminal diiodination reaction could be achieved on gram quantities by prolonging the reaction time (Fig. 7a). To further demonstrate the synthetic utility of our methods, derivatization of generated geminal dihalide products was attempted (Fig. 7b). The diiodo compound **2** could be easily converted to disubstituted alkene **57** with moderate stereoselectivity by Takai-Utimoto olefination^52,53^. Base-promoted elimination of **2** led to vinyl iodide **58** in excellent yield with a moderate *E*/*Z* selectivity^54^. Synthetically useful geminal bis(boronate) (**59**) was easily produced by subjecting the diiodo compound **2** to copper-catalyzed boronation^55^. A photoredox catalyzed deiodination using Hantzsch ester as the hydrogen source reduced **2** to **60** bearing an unsubstituted alkyl chain. More interestingly, highly selective derivatization of the iodide in the bromo(iodo)alkane products could be achieved. The photoredox reaction using Hantzsch ester converted **24** to alkylbromide **61** in 90% yield. Based-promoted elimination of **24** selectively produced the corresponding vinyl bromide **62**. Bromo-iodide **24** could undergo a selective S_N_2 reaction with sodium azide to afford **63** in 86% yield. Subjection of this azide (**63**) to copper-catalyzed azide-alkyne cycloaddition afforded a bromo triazole^56^, which could undergo further nucleophilic substitution to deliver *α*-functionalized triazole **64** in 69% yield.

## Discussion

In summary, we herein report a simple protocol for deconstructive geminal diiodination and bromo-iodination of trisubstituted alkenes under visible light irradiation. The success of this transformation relies on the formation of a labile alkyl hypoiodite intermediate between the halohydrin and DIH. The protocol is distinguished by its operational simplicity, metal-free and catalyst-free characters, a wide scope of both cyclic and acyclic alkenes, delivery of useful and otherwise difficultly accessible synthons, and controllable chain length of products by choosing alkenes with different ring sizes.

## Methods

### General procedure of the deconstructive geminal diiodination

Alkene (0.2 mmol, 1 equiv) and 1,3-diiodo-5,5-dimethylhydantoin (0.4 mmol, 2 equiv), H_2_O (10 mmol, 50 equiv), and EtOAc:MeNO_2_ (10:1, 2 mL) were added to a schlenk tube (10 mL) equipped with a magnetic stirring bar. Then, the reaction mixture was operated by freeze-pump-thaw procedures three times and backfilled with argon. The resulting solution was irradiated by blue LED lamps (2 × 40 W) and magnetically stirred at 50 °C. After 36 h, the reaction solution was concentrated, and the product was purified by column chromatography (SiO_2_). The diastereomeric ratio was determined by ^1^H NMR of the crude product mixture. See Supplementary Methods for details.

### General procedure of the deconstructive bromo-iodination

Alkene (0.2 mmol) and *N*-bromosuccinimide (0.21 mmol, 1 equiv), H_2_O (10 mmol, 50 equiv), and MeCN (2 mL) were added to a schlenk tube (10 mL) equipped with a magnetic stirring bar. The reaction mixture was operated by freeze-pump-thaw procedures for three times and backfilled with argon. The resulting solution was magnetically stirred at 50 °C for 12 h. Then, 1,3-diiodo-5,5-dimethylhydantoin (0.3 mmol, 1.5 equiv) was added to the reaction mixture under argon. The reaction mixture was irradiated by blue LED lamps (2 × 40 W) and magnetically stirred at 50 ^o^C. After 24 h, the reaction solution was concentrated, and the product was purified by column chromatography (SiO_2_). The diastereomeric ratio was determined by ^1^H NMR of the crude product mixture. See Supplementary Methods for details.

### Computational details

Density functional theory (DFT) calculations were performed for the verification of the mechanism. The geometries optimization in this study (except **56** and 56*) was performed at the (u)B3LYP‐D3(BJ) level of theory. The 6–311 + g(d,p) basis set was used for all H, C, N, and O atoms, and the Stuttgart–Dresden basis set (SDD) was employed for Br and I atoms. The nature of the stationary points (minima with no imaginary frequency or transition states with one imaginary frequency) was confirmed. The free energies of the optimized geometries were calculated at the same level of theory, taking into account the solvent effect of acetonitrile using Solvent Polarizable Continuum Model (PCM). Unless specified otherwise, the Gibbs free energy was used throughout. Considering the deviation in the free energies is ~1.89 kcal/mol from the standard state (1 atm) to 1 M in solution, we reduced by 1.89 kcal/mol to the free energy for additional steps and added by 1.89 kcal/mol for the dissociation steps^57^. For transition state, intrinsic reaction coordinate (IRC) calculations were performed to verify whether it connected with correct reactants and products or intermediates. Time-dependent density functional theory (TD-DFT) was performed to calculate the vertical excitation energies of the photodissociation process, using the CAM-B3LYP‐D3(BJ)^58^ level of theory with the same basis set. All calculations were performed using the Gaussian 16 Rev. A.03 software suite^59^. The geometries were realized using CYLview, 1.0^60^.

## Supplementary information

Supplementary Information

Peer Review File

## Data Availability

The authors declare that all other data supporting the findings of this study are available within the article and Supplementary Information files, and also are available from the corresponding author upon reasonable request.

## References

[1] Hoveyda AH, Evans DA, Fu GC (1993). Substrate-directable chemical reactions. Chem. Rev..

[2] Grubbs RH, Chang S (1998). Recent advances in olefin metathesis and its application in organic synthesis. Tetrahedron.

[3] McDonald RI, Liu G, Stahl SS (2011). Palladium(II)-catalyzed alkene functionalization via nucleopalladation: stereochemical pathways and enantioselective catalytic applications. Chem. Rev..

[4] Vasseur A, Bruffaerts J, Marek I (2016). Remote functionalization through alkene isomerization. Nat. Chem..

[5] Dong Z, Ren Z, Thompson SJ, Xu Y, Dong G (2017). Transition-metal-catalyzed C–H alkylation using alkenes. Chem. Rev..

[6] Lan X-W, Wang N-X, Xing Y (2017). Recent advances in radical difunctionalization of simple alkenes. Eur. J. Org. Chem.

[7] Lee JH, Choi S, Hong KB (2019). Alkene difunctionalization using hypervalent iodine reagents: progress and developments in the past ten years. Molecules.

[8] Lin J, Song R-J, Hu M, Li J-H (2019). Recent advances in the intermolecular oxidative difunctionalization of alkenes. Chem. Rec..

[9] Grubbs RH, Miller SJ, Fu GC (1995). Ring-closing metathesis and related processes in organic synthesis. Acc. Chem. Res..

[10] Hoveyda AH, Zhugralin AR (2007). The remarkable metal-catalysed olefin metathesis reaction. Nature.

[11] Grubbs, R. H. & O’Leary, D. J. *Handbook of metathesis, Volume 2: Applications in Organic Synthesis*, 2nd edn. (Wiley-VCH, Weinhein, 2015).

[12] Shen X (2017). Kinetically *E*-selective macrocyclic ring-closing metathesis. Nature.

[13] Bailey PS (1958). The reactions of ozone with organic compounds. Chem. Rev..

[14] Ornum SGV, Champeau RM, Pariza R (2006). Ozonolysis applications in drug synthesis. Chem. Rev..

[15] Daw P (2014). A highly efficient catalyst for selective oxidative scission of olefins to aldehydes: abnormal-NHC–Ru(II) complex in oxidation chemistry. J. Am. Chem. Soc..

[16] Wang T, Jing X, Chen C, Yu L (2017). Organoselenium-catalyzed oxidative C═C bond cleavage: a relatively green oxidation of alkenes into carbonyl compounds with hydrogen peroxide. J. Org. Chem..

[17] Yu W, Zhao Z (2019). Catalyst-free selective oxidation of diverse olefins to carbonyls in high yield enabled by light under mild conditions. Org. Lett..

[18] Wang T, Jiao N (2013). TEMPO-catalyzed aerobic oxygenation and nitrogenation of olefins via C═C double-bond cleavage. J. Am. Chem. Soc..

[19] Liu H, Feng M, Jiang X (2014). Unstrained carbon-carbon bond cleavage. Chem. Asian J..

[20] Zong X, Zheng Q-Z, Jiao N (2014). NBS mediated nitriles synthesis through C=C double bond cleavage. Org. Biomol. Chem..

[21] Chen W-L (2018). Synthesis of 2-aminobenzonitriles through nitrosation reaction and sequential iron(III)-catalyzed C–C bond cleavage of 2-arylindoles. Org. Lett..

[22] Sivaguru P, Wang Z, Zanoni G, Bi X (2019). Cleavage of carbon–carbon bonds by radical reactions. Chem. Soc. Rev..

[23] Li J, Wei J, Zhu B, Wang T, Jiao N (2019). Cu-catalyzed oxygenation of alkene-tethered amides with O_2_ via unactivated C=C bond cleavage: a direct approach to cyclic imides. Chem. Sci..

[24] Wu X, Zhu C (2018). Recent advances in ring-opening functionalization of cycloalkanols by C–C σ-Bond cleavage. Chem. Rec..

[25] Wu X, Zhu C (2019). Recent advances in alkoxy radical-promoted C–C and C–H bond functionalization starting from free alcohols. Chem. Commun..

[26] Lin R, Chen F, Jiao N (2012). Metal-free, NHPI catalyzed oxidative cleavage of C-C double bond using molecular oxygen as oxidant. Org. Lett..

[27] Hu W (2018). Palladium-catalyzed intermolecular oxidative coupling reactions of (*Z*)-enamines with isocyanides through selective β-C(sp^2^)-H and/or C=C bond cleavage. Chin. J. Chem..

[28] Hughes ED (1951). Reactions of halides in solution. Q. Rev. Chem. Soc..

[29] Saikia I, Borah AJ, Phukan P (2016). Use of bromine and bromo-organic compounds in organic synthesis. Chem. Rev..

[30] Biffis A, Centomo P, Zotto AD, Zecca M (2018). Pd metal catalysts for cross-couplings and related reactions in the 21st century: a critical review. Chem. Rev..

[31] Conly JC (1953). Applications of the hunsdiecker silver salt degradation. The preparation of dibromides and tribromides. J. Am. Chem. Soc..

[32] Doyle MP, Siegfried B (1976). Oxidative deamination of primary amines: selective synthesis of geminal dihalides. J. Chem. Soc. Chem. Commun.

[33] Lansinger JM, Ronald RC (1979). Reactions of aromatic aldehydes with boron halides. Synth. Commun..

[34] Huan Z, Landgrebe JA, Peterson K (1983). Deoxygenation of aldehydes and ketones, a new general reaction of dibromocarbene and dibromocarbonyl ylides. Tetrahedron Lett..

[35] Cloarec J-M, Charette AB (2004). Highly efficient two-step synthesis of C-sp^3^-centered geminal diiodides. Org. Lett..

[36] Zhao H, Fan X, Yu J, Zhu C (2015). Silver-catalyzed ring-opening strategy for the synthesis of β- and γ-fluorinated ketone. J. Am. Chem. Soc..

[37] Ren R, Zhao H, Huan L, Zhu C (2015). Manganese-catalyzed oxidative azidation of cyclobutanols: regiospecific synthesis of alkyl azides by C-C bond cleavage. Angew. Chem. Int. Ed..

[38] Jia K, Zhang F, Huang H, Chen Y (2016). Visible-light-induced alkoxyl radical generation enables selective C(sp^3^)-C(sp^3^) bond cleavage and functionalizations. J. Am. Chem. Soc..

[39] Jia K, Pan Y, Chen Y (2017). Selective carbonyl-C(sp3) bond cleavage to construct ynamides, ynoates, and ynones by photoredox catalysis. Angew. Chem. Int. Ed..

[40] Wang D, Mao J, Zhu C (2018). Visible light-promoted ring-opening functionalization of unstrained cycloalkanols via inert C-C bond scission. Chem. Sci..

[41] Wu X (2018). Metal-free alcohol-directed regioselective heteroarylation of remote unactivated C(sp3)-H bonds. Nat. Commun..

[42] Ji M, Wu Z, Zhu C (2019). Visible-light-induced consecutive C–C bond fragmentation and formation for the synthesis of elusive unsymmetric 1,8-dicarbonyl compounds. Chem. Commun..

[43] Beebe T (1975). Oxidation of alcohols with acetyl hypoiodite. J. Org. Chem..

[44] Yang Y, Su C, Huang X, Liu Q (2009). Halohydroxylation of alkylidenecyclopropanes using *N*-halosuccinimide (NXS) as the halogen source: an efficient synthesis of halocyclopropylmethanol and 3-halobut-3-en-1-ol derivatives. Tetrahedron Lett..

[45] Janjatovic J, Majerski Z (1980). Synthesis of adamantanoid ketones from bridgehead alcohols by the hypoiodite thermolysis-cyclization sequence. J. Org. Chem..

[46] Courtneidge JL, Lusztyk J, Pagé D (1994). Alkoxyl radicals from alcohols. Spectroscopic detection of intermediate alkyl and acyl hypoiodites in the Suárez and Beebe reactions. Tetrahedron Lett..

[47] Suginome H, Takeda T, Itoh M, Nakayama Y, Kobayashi K (1995). Photoinduced molecular transformations. Part 152. Ring expansion based on a sensitized [2 + 2] photoaddition of enol ethers of cyclic ketones with olefins, followed by a β-scission of alkoxyl radicals generated from the resulting cyclobutanols. Two-carbon ring expansion of β-indanone, β-tetralone and β-suberone. J. Chem. Soc. Perkin Trans..

[48] Guo J-J (2016). Photocatalytic C−C bond cleavage and amination of cycloalkanols by cerium(III) chloride complex. Angew. Chem. Int. Ed..

[49] Shi J-L, Wang Y, Wang Z, Dou B, Wang J (2020). Ring-opening iodination and bromination of unstrained cycloalkanols through β-scission of alkoxy radicals. Chem. Commun..

[50] Roque JB, Kuroda Y, Göttemann LT, Sarpong R (2018). Deconstructive diversification of cyclic amines. Nature.

[51] Wang Y (2020). Visible-light-promoted site-specific and diverse functionalization of a C(sp^3^)–C(sp^3^) bond adjacent to an arene. ACS Catal..

[52] Takai K, Nitta K, Utimoto K (1986). Simple and selective method for aldehydes (RCHO)→(*E*)-haloalkenes (RCH:CHX) conversion by means of a haloform-chromous chloride system. J. Am. Chem. Soc..

[53] Okazoe, T., Takai, K. & Utimoto, K. (E)-Selective olefination of aldehydes by means of gem-dichromium reagents derived by reduction of gem-diiodoalkanes with chromium(II) chloride. *J. Am. Chem. Soc*. **109**, 951–953 (1987).

[54] Martínez AG, Alvareza RM, González SM, Subramanian LR, Conrad M (1992). A new procedure for the synthesis of (E)-1-iodo-1-alkenes. Tetrahedron Lett..

[55] Zhang Z-Q (2014). Copper-catalyzed/promoted cross-coupling of gem-diborylalkanes with nonactivated primary alkyl halides: an alternative route to alkylboronic esters. Org. Lett..

[56] Himo F (2005). Copper(I)-catalyzed synthesis of azoles. DFT study predicts unprecedented reactivity and intermediates. J. Am. Chem. Soc..

[57] Zhu C (2019). A multicomponent synthesis of stereodefined olefins via nickel catalysis and single electron/triplet energy transfer. Nat. Catal..

[58] Yanai T, Tew DP, Handy NC (2004). A new hybrid exchange–correlation functional using the coulomb-attenuating method (CAM-B3LYP). Chem. Phys. Lett..

[59] Frisch, M. J. et al. Gaussian 16, revision C.01 (Gaussian, 2016).

[60] Legault, C. Y. *CYLview 1.0b* (Unversité de Sherbrooke, 2009) http://www.cylview.org.

