# Supplementary material for: Photo-mediated selective deconstructive geminal dihalogenation of trisubstituted alkenes
Source: Nat Commun. 2020 Sep 8;11:4462. doi: 10.1038/s41467-020-18274-2 (PMC7479597; doi:10.1038/s41467-020-18274-2)
Supplement: Supplementary file 1 — Supplementary Information [file 41467_2020_18274_MOESM1_ESM.pdf]

Supplementary Information

Photo-mediated Selective Geminal Dihalogenation of Trisubstituted Alkenes

Wang et al.

Supplementary Methods

General Information

Unless otherwise noted, all chemicals were purchased from commercial suppliers (Sigma Aldrich, TCI, Oakwood) and used without further purification. When required, solvents were dried according to general purification methods. Two Kessil PR160 blue LED lamps (40 W) were used as the light source. The product mixtures were analyzed by thin layer chromatography using TLC silica gel plates (Merck-Schuchardt) with fluorescent indicator (λ = 254 nm). The purification of the products was performed by flash column chromatography using silica gel 60 (63-200 μm) from SANPONT. NMR spectra were recorded on Bruker AV-III400 (400 MHZ) or AMX500 (500 MHz) spectrometer in deuterated solvents. Chemical shifts (δ) are reported in parts per million (ppm) and spin-spin coupling constants (J) are given in Hz, while multiplicities are given the standard abbreviations: s (singlet), d (doublet), t (triplet), q (quartet), br (broad), m (multiplet). High resolution mass spectra (HRMS) were recorded on a Finnigan/MAT 95XL-T spectrometer. The diastereomeric ratio (dr) was determined by ^{1}H NMR of the crude product mixture. Absorption spectra were recorded in 1 cm path quartz cuvettes using an Edinburgh FS-5 spectrofluorometer. Continuous wave X-band ESR spectra were obtained with a JEOL (FA200) spectrometer.

Experimental set-up

As experimental set-up, a stirring plate and Kessil PR160 blue LED lamps were used, and the distance between the LED lamps and the reactor is 8 cm. The temperature could be controlled at 30 ^{o}C when a cooling fan was used. Without the cooling fan, the temperature was maintained around 50 ^{o}C, which was applied as our standard reaction conditions. The higher temperature (80 ^{o}C, 100 ^{o}C) was controlled by a water or oil bath with a heating plat equipped with digital temperature control.

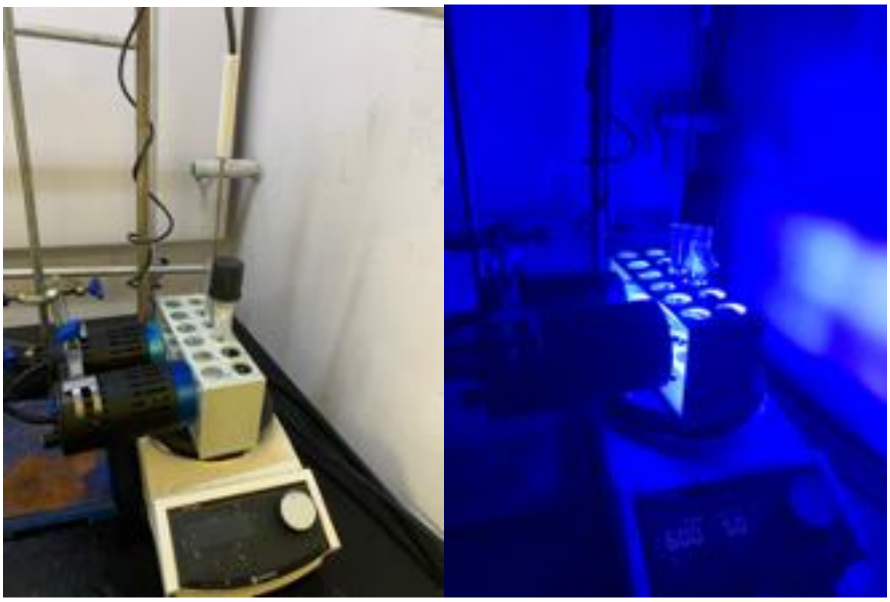

Supplementary Figure 1. Experimental set-up

Deconstructive geminal diiodination

General procedure of the deconstructive geminal diiodination

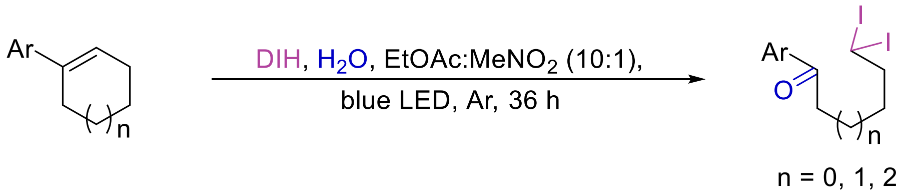

General procedure A:

Alkene (0.2 mmol, 1 equiv) and 1,3-diiodo-5,5-dimethylhydantoin (0.4 mmol, 2 equiv), H_2O (10 mmol, 50 equiv), and EtOAc:$MeNO_2$ (10:1, 2 mL) were added to a schlenk tube (10 mL) equipped with a magnetic stirring bar. Then, the reaction mixture was operated by freeze-pump-thaw procedures for three times and backfilled with argon. The resulting solution was irradiated by blue LED lamps (2*40 W) and magnetically stirred at 50 ^oC. After 36 hours, the reaction solution was concentrated, and the product was purified by column chromatography (SiO_2). The diastereomeric ratio was determined by ^1H NMR of the crude product mixture.

General procedure B:

Alkene (0.2 mmol, 1 equiv) and *N*-iodosuccinimide (0.8 mmol, 4 equiv), H_2O (10 mmol, 50 equiv), and EtOAc:$MeNO_2$ (10:1, 2 mL) were added to a schlenk tube (10 mL) equipped with a magnetic stirring bar. Then, the reaction mixture was operated by freeze-pump-thaw procedures for three times and backfilled with argon. The resulting solution was irradiated by blue LED lamps (2*40 W) and magnetically stirred at 50 ^oC. After 36 hours, the reaction solution was concentrated, and the product was purified by column chromatography (SiO_2).

General procedure C:

Alkene (0.2 mmol, 1 equiv) and 1,3-diiodo-5,5-dimethylhydantoin (0.4 mmol, 2 equiv), H_2O (10 mmol, 50 equiv), and EtOAc:$MeNO_2$ (10:1, 2 mL) were added to a schlenk tube (10 mL) equipped with a magnetic stirring bar. Then, the reaction mixture was operated by freeze-pump-thaw procedures for three times and backfilled with argon. The resulting solution was magnetically stirred at 0 ^oC for 6 hours. After that, the reaction mixture was irradiated by blue LED lamps (2*40 W) and magnetically stirred at 50 ^oC. After 30 hours, the reaction solution was concentrated, and the product was purified by column chromatography (SiO_2).

6,6-diiodo-1-phenylhexan-1-one (2)

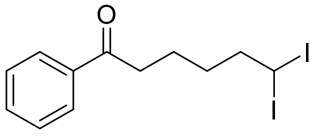

Following the general procedure A afforded the product as a yellow viscous oil (72 mg, 84% yield). ^1H NMR (400 MHz, Chloroform-*d*) δ 8.01 – 7.91 (m, 2H), 7.59 – 7.53 (m, 1H), 7.50 – 7.42 (m, 2H), 5.14 (t, *J* = 6.4 Hz, 1H), 3.02 (t, *J* = 7.2 Hz, 2H)72, 2.48 – 2.35 (m, 2H), 1.81 (dt, *J* = 15.1, 7.4 Hz, 2H), 1.59 – 1.47 (m, 2H); ^{13}C NMR (126 MHz, $CDCl_3$) δ 199.65, 136.89, 133.08, 128.63, 128.01, 48.05, 38.16, 31.55, 22.09; HRMS ESI (m/z): $[M+H]^+$calcd. for $C_{12}H_{15}I_2O$, 428.9207; found, 428.9202.

1-([1,1'-biphenyl]-4-yl)-6,6-diiodohexan-1-one (3)

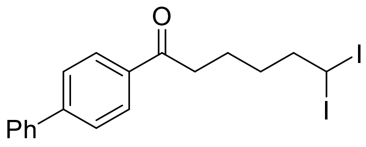

Following the general procedure A afforded the product as a yellow viscous oil (82 mg, 81% yield). ^1H NMR (400 MHz, Chloroform-*d*) δ 8.06 – 7.97 (m, 2H), 7.71 – 7.68 (m, 2H), 7.66 – 7.59 (m, 2H), 7.52 – 7.43 (m, 2H), 7.43 – 7.34 (m, 1H), 5.15 (t, *J* = 6.4 Hz, 1H), 3.04 (t, *J* = 7.3 Hz, 2H), 2.48 – 2.37 (m, 2H), 1.89 – 1.74 (m, 2H), 1.60 – 1.53 (m, 2H); ^{13}C NMR

(126 MHz, $CDCl_3$) δ 199.25, 145.76, 139.86, 135.58, 128.97, 128.63, 128.25, 127.28, 48.06, 38.22, 31.58, 22.17; HRMS ESI (m/z): $[M+H]^+$calcd. for $C_{18}H_{19}I_2O$, 504.9520; found, 504.9518.

6,6-diiodo-1-(p-tolyl)hexan-1-one (4)

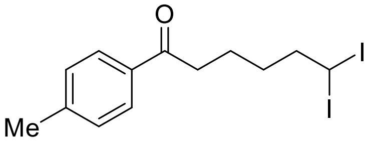

Following the general procedure A afforded the product as a yellow viscous oil (71 mg, 80% yield). ^1H NMR (400 MHz, Chloroform-*d*) δ 7.89 – 7.80 (m, 2H), 7.35 – 7.12 (m, 2H), 5.14 (t, J = 6.4 Hz, 1H), 2.98 (t, J = 7.3 Hz, 2H), 2.52 – 2.39 (m, 5H), 1.85 – 1.71 (m, 2H), 1.54 – 1.42 (m, 2H); ^{13}C NMR (126 MHz, $CDCl_3$) δ 199.34, 143.85, 134.43, 129.29, 128.14, 48.07, 38.05, 31.57, 22.19, 21.64; HRMS ESI (m/z): $[M+H]^+$ calcd. for $C_{13}H_{17}I_2O$, 442.9363; found, 442.9362.

6,6-diiodo-1-(4-methoxyphenyl)hexan-1-one (5)

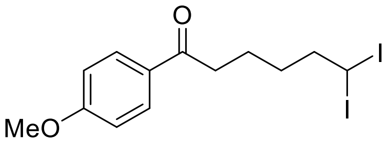

Following the general procedure A afforded the product as a yellow viscous oil (62 mg, 68% yield). ^1H NMR (400 MHz, Chloroform-*d*) δ 8.00 – 7.79 (m, 2H), 7.01 – 6.80 (m, 2H), 5.13 (t, J = 6.5 Hz, 1H), 3.87 (s, 3H), 2.96 (t, J = 7.3 Hz, 2H), 2.54 – 2.32 (m, 2H), 1.87 – 1.73 (m, 2H), 1.56 – 1.43 (m, 2H); ^{13}C NMR (126 MHz, $CDCl_3$) δ 198.25, 163.46, 130.28, 130.00, 113.75, 55.49, 48.08, 37.81, 31.61, 22.30; HRMS ESI (m/z): $[M+H]^+$ calcd. for $C_{13}H_{17}I_2O_2$, 458.9312; found, 458.9303.

1-(4-(tert-butyl)phenyl)-6,6-diiodohexan-1-one (6)

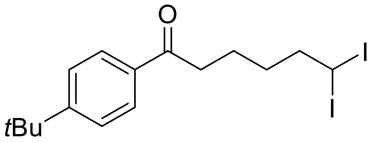

Following the general procedure A afforded the product as a colourless viscous oil (70 mg, 72% yield). ^1H NMR (400 MHz, Chloroform-*d*) δ 7.90 (d, J = 8.6 Hz, 2H), 7.56 – 7.43 (m, 2H), 5.14 (t, J = 6.5 Hz, 1H), 2.99 (t, J = 7.3 Hz, 2H), 2.47 – 2.26 (m, 2H), 1.91 – 1.72 (m, 2H), 1.54 – 1.46 (m, 2H), 1.34 (s, 9H); ^{13}C NMR (126 MHz, $CDCl_3$) δ 199.36, 156.80, 134.33, 128.00, 125.56, 48.08, 38.07, 35.12, 31.58, 31.11, 22.21; HRMS ESI (m/z): $[M+H]^+$ $C_{16}H_{23}I_2O$, 484.9833; found, 484.9833.

1-(4-fluorophenyl)-6,6-diiodohexan-1-one (7)

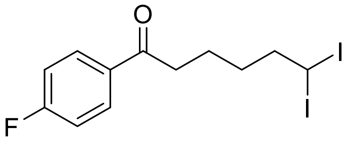

Following the general procedure A afforded the product as a colourless viscous oil (71 mg, 80% yield). ^1H NMR (400 MHz, Chloroform-*d*) δ 8.06 – 7.86 (m, 2H), 7.20 – 7.05 (m, 2H), 5.14 (t, J = 6.4 Hz, 1H), 2.99 (t, J = 7.2 Hz, 2H), 2.51 – 2.30 (m, 2H), 1.88 – 1.71 (m, 2H), 1.64 – 1.44 (m, 2H); ^{13}C NMR (126 MHz, $CDCl_3$) δ 197.98, 166.75, 164.72, 133.33, 130.66, 130.59, 115.80, 115.63, 48.01, 38.07, 31.51, 22.05; HRMS ESI (m/z): $[M+H]^+$ calcd. for $C_{12}H_{14}FI_2O$, 446.9113; found, 446.9109.

1-(3-(tert-butyl)phenyl)-6,6-diiodohexan-1-one (8)

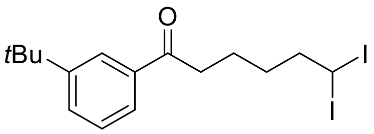

Following the general procedure A afforded the product as a colourless viscous oil (75 mg, 77% yield). ^1H NMR (400 MHz, Chloroform-*d*) δ 8.00 (s, 1H), 7.80 – 7.71 (m, 1H), 7.62 – 7.59 (m, 1H), 7.45 – 7.36 (m, 1H), 5.14 (t, J = 6.4 Hz, 1H), 3.02 (t, J = 7.2 Hz, 2H), 2.49 – 2.39 (m, 2H), 1.87 – 1.77 (m, 2H), 1.58 – 1.49 (m, 2H), 1.36 (s, 9H); ^{13}C NMR (126 MHz, $CDCl_3$) δ 200.04, 151.77, 136.78, 130.25, 128.33, 125.40, 124.72, 48.09, 38.25, 34.89, 31.59, 31.29, 22.20; HRMS ESI (m/z): $[M+H]^+$ calcd. for $C_{16}H_{23}I_2O$, 484.9833; found, 484.9836.

1-(3-fluorophenyl)-6,6-diiodohexan-1-one (9)

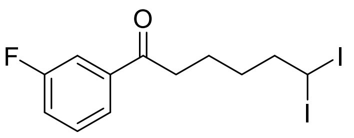

Following the general procedure A afforded the product as a colourless viscous oil (77 mg, 86% yield). ^{1}H NMR (400 MHz, Chloroform-*d*) δ 7.75 – 7.71 (m, 1H), 7.65 – 7.61 (m, 1H), 7.47 – 7.41 (m, 1H), 7.30 – 7.23 (m, 1H), 5.14 (t, *J* = 6.4 Hz, 1H), 2.99 (t, *J* = 7.2 Hz, 2H), 2.46 – 2.38 (m, 2H), 1.86 – 1.76 (m, 2H), 1.58 – 1.49 (m, 2H); ^{13}C NMR (126 MHz, $CDCl_3$) δ 198.30, 163.87, 161.90, 138.93, 130.34, 130.27, 123.76, 123.73, 120.19, 120.02, 114.86, 114.68, 47.98, 38.33, 31.47, 21.95; HRMS ESI (m/z): $[M+H]^+$ $C_{12}H_{14}FI_2O$, 446.9113; found, 446.9102.

6,6-diiodo-1-(o-tolyl)hexan-1-one (10)

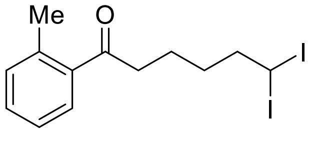

Following the general procedure A afforded the product as a colourless viscous oil (68 mg, 77% yield).^{1}H NMR (400 MHz, Chloroform-*d*) δ 7.65 – 7.59 (m, 1H), 7.41 – 7.33 (m, 1H), 7.28 – 7.22 (m, 2H), 5.13 (t, *J* = 6.4 Hz, 1H), 2.93 (t, *J* = 7.2 Hz, 2H), 2.50 (s, 3H), 2.44 – 2.37 (m, 2H), 1.83 – 1.71 (m, 2H), 1.54 – 1.47 (m, 2H); ^{13}C NMR (126 MHz, $CDCl_3$) δ 203.89, 137.99, 132.00, 131.25, 128.31, 125.69, 48.04, 41.13, 31.49, 22.29, 21.30; HRMS ESI (m/z): $[M+H]^+$ calcd. for $C_{13}H_{17}I_2O$, 442.9363; found, 442.9359.

6,6-diiodo-4-methoxy-1-phenylhexan-1-one (11)

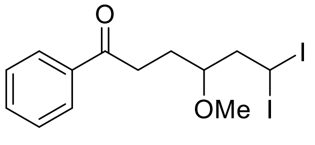

Following the general procedure A afforded the product as a colourless viscous oil (72 mg, 79% yield). ^{1}H NMR (400 MHz, Chloroform-*d*) δ 8.03 – 7.92 (m, 2H), 7.62 – 7.54 (m, 1H), 7.51 – 7.43 (m, 2H), 5.17 (dd, *J* = 10.2, 4.4 Hz, 1H), 3.39 (s, 3H), 3.38 – 3.27 (m, 1H), 3.09 – 2.98 (m, 2H), 2.70 – 2.58 (m, 1H), 2.51 – 2.39 (m, 1H), 2.10 – 1.90 (m, 2H); ^{13}C NMR (126 MHz, $CDCl_3$) δ 199.38, 136.82, 133.17, 128.66, 128.03, 80.49, 57.20, 53.22, 33.38, 25.94; HRMS ESI (m/z): $[M+Na]^+$ calcd. for $C_{13}H_{16}I_2O_2Na$, 480.9132; found, 480.9128.

6,6-diiodo-4-methyl-1-phenylhexan-1-one (12)

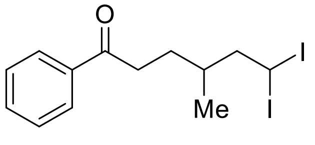

Following the general procedure A afforded the product as a colourless viscous oil (73 mg, 83% yield). ^{1}H NMR (400 MHz, Chloroform-*d*) δ 7.99 – 7.91 (m, 2H), 7.59 – 7.52 (m, 1H), 7.50 – 7.44 (m, 2H), 5.11 (dd, *J* = 8.9, 6.3 Hz, 1H), 3.01 – 2.94 (m, 2H), 2.52 – 2.39 (m, 1H), 2.31 – 2.20 (m, 1H), 1.84 – 1.76 (m, 1H), 1.68 – 1.58 (m, 2H), 0.94 (d, *J* = 6.4 Hz, 3H); ^{13}C NMR (126 MHz, $CDCl_3$) δ 199.84, 136.88, 133.09, 128.64, 128.04, 55.69, 35.85, 35.49, 29.52, 18.02; HRMS ESI (m/z): $[M+H]^+$ calcd. for $C_{13}H_{17}I_2O$, 442.9363; found, 442.9366.

4-ethyl-6,6-diiodo-1-phenylhexan-1-one (13)

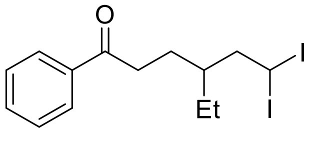

Following the general procedure A afforded the product as a colourless viscous oil (68 mg, 75% yield). ^{1}H NMR (400 MHz, Chloroform-*d*) δ 8.04 – 7.89 (m, 2H), 7.60 – 7.53 (m, 1H), 7.52 – 7.43 (m, 2H), 5.11 (t, *J* = 7.5 Hz, 1H), 3.06 – 2.92 (m, 2H), 2.46 – 2.32 (m, 2H), 1.78 – 1.69 (m, 2H), 1.53 – 1.46 (m, 1H), 1.43 – 1.33 (m, 2H), 0.92 (t, *J* = 7.4 Hz, 3H); ^{13}C NMR (126 MHz, $CDCl_3$) δ 199.89, 136.91, 133.10, 128.65, 128.04, 52.90, 41.19, 35.52, 25.93, 24.54, 10.29; HRMS ESI (m/z): $[M+H]^+$ calcd. for $C_{14}H_{19}I_2O$, 456.9520; found, 456.9515.

6,6-diiodo-1,4-diphenylhexan-1-one (14)

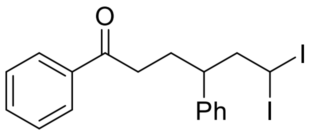

Following the general procedure A afforded the product as a colourless viscous oil (75 mg, 74% yield). ^{1}H NMR (400 MHz, Chloroform-*d*) δ 8.03 – 7.92 (m, 2H), 7.62 – 7.54 (m, 1H), 7.51 – 7.43 (m, 2H), 5.17 (dd, *J* = 10.2, 4.4 Hz, 1H), 3.39 (s, 3H), 3.38 – 3.27 (m, 1H), 3.09 – 2.98 (m, 2H), 2.70 – 2.58 (m, 1H), 2.51 – 2.39 (m, 1H), 2.10 – 1.90 (m, 2H); ^{13}C NMR (126 MHz, $CDCl_3$) δ 199.38, 136.82, 133.17, 128.66, 128.03, 80.49, 57.20, 53.22, 33.38, 25.94; HRMS ESI (m/z): $[M+H]^+$ calcd. for $C_{18}H_{19}I_2O$, 504.9520; found, 504.9512.

4,4-difluoro-6,6-diiodo-1-phenylhexan-1-one (15)

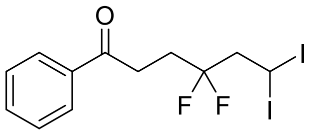

Following the general procedure A afforded the product as a colourless viscous oil (56 mg, 60% yield). ^{1}H NMR (400 MHz, Chloroform-*d*) δ 8.01 – 7.95 (m, 2H), 7.61 – 7.56 (m, 1H), 7.52 – 7.44 (m, 2H), 5.25 (t, *J* = 6.8 Hz, 1H), 3.46 – 3.35 (m, 2H), 3.26 – 3.20 (m, 2H), 2.41 – 2.26 (m, 2H); ^{13}C NMR (126 MHz, $CDCl_3$) δ 197.58, 136.38, 133.46, 128.73, 128.04, 125.34, 123.40, 121.44, 54.77, 54.58, 54.37, 31.21, 31.01, 30.82, 30.67, 30.64, 30.62; HRMS ESI (m/z): $[M+Na]^+$ calcd. for $C_{12}H_{12}F_2I_2ONa$, 486.8838; found, 486.8830.

6,6-diiodo-1-(naphthalen-2-yl)hexan-1-one (16)

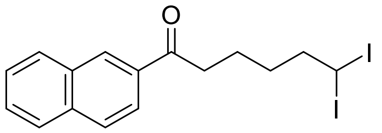

Following the general procedure A afforded the product as a colourless solid (82 mg, 86% yield). ^{1}H NMR (400 MHz, Chloroform-*d*) δ 8.47 (d, *J* = 1.7 Hz, 1H), 8.03 (dd, *J* = 8.6, 1.8 Hz, 1H), 7.97 (dd, *J* = 8.2, 1.3 Hz, 1H), 7.89 (t, *J* = 8.4 Hz, 2H), 7.65 – 7.52 (m, 2H), 5.16 (t, *J* = 6.4 Hz, 1H), 3.15 (t, *J* = 7.3 Hz, 2H), 2.49 – 2.41 (m, 2H), 1.93 – 1.81 (m, 2H), 1.63 – 1.52 (m, 2H); ^{13}C NMR (126 MHz, $CDCl_3$) δ 199.61, 135.60, 134.23, 132.54, 129.64, 129.56, 128.50, 128.47, 127.81, 126.82, 123.85, 48.07, 38.26, 31.62, 22.26; HRMS ESI (m/z): $[M+H]^+$ calcd. for $C_{16}H_{17}I_2O$, 478.9363; found, 478.9362.

6,6-diiodo-1-(thiophen-3-yl)hexan-1-one (17)

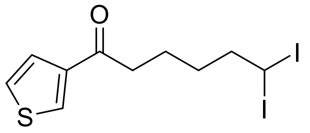

Following the general procedure B afforded the product as a colourless oil (54 mg, 62% yield). ^{1}H NMR (400 MHz, Chloroform-*d*) δ 8.05 (dd, *J* = 2.9, 1.3 Hz, 1H), 7.55 (dd, *J* = 5.1, 1.3 Hz, 1H), 7.32 (dd, *J* = 5.1, 2.9 Hz, 1H), 5.13 (t, *J* = 6.4 Hz, 1H), 2.92 (t, *J* = 7.3 Hz, 2H), 2.48 – 2.34 (m, 2H), 1.85 – 1.74 (m, 2H), 1.58 – 1.45 (m, 2H); ^{13}C NMR (126 MHz, $CDCl_3$) δ 194.01, 142.25, 131.80, 126.92, 126.43, 48.03, 39.44, 31.54, 22.08; HRMS ESI (m/z): $[M+H]^+$ calcd. for $C_{10}H_{13}I_2OS$, 434.8771; found, 434.8778.

1-(benzo[b]thiophen-2-yl)-6,6-diiodohexan-1-one (18)

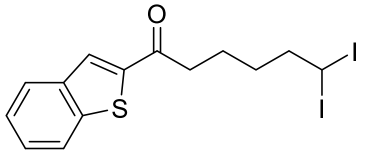

Following the general procedure A afforded the product as a colourless oil (66 mg, 68% yield). ^{1}H NMR (400 MHz, Chloroform-*d*) δ 7.97 (d, *J* = 0.8 Hz, 1H), 7.93 – 7.85 (m, 2H), 7.50 – 7.39 (m, 2H), 5.14 (t, *J* = 6.4 Hz, 1H), 3.06 (t, *J* = 7.3 Hz, 2H), 2.48 – 2.38 (m, 2H), 1.86 (dt, *J* = 15.2, 7.4 Hz, 2H), 1.57 – 1.51 (m, 2H); ^{13}C NMR (126 MHz, $CDCl_3$) δ 194.08, 143.58, 142.47, 139.11, 128.89, 127.43, 125.91, 125.04, 123.02, 47.95, 38.84, 31.50, 22.42; HRMS ESI (m/z): $[M+H]^+$ calcd. for $C_{14}H_{15}I_2OS$, 484.8927; found, 484.8928.

5,5-diiodo-1-phenylpentan-1-one (19)

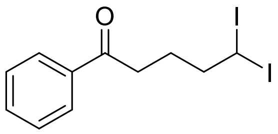

Following the general procedure C afforded the product as a colourless oil (58 mg, 70% yield). ^{1}H NMR (400 MHz, Chloroform-*d*) δ 8.03 – 7.89 (m, 2H), 7.63 – 7.51 (m, 1H), 7.51 – 7.37 (m, 2H), 5.16 (t, *J* = 6.5 Hz, 1H), 3.05 (t, *J* = 7.0 Hz, 2H), 2.52 – 2.39 (m, 2H), 1.97 – 1.81 (m, 2H); ^{13}C NMR (126 MHz, $CDCl_3$) δ 199.00, 136.68, 133.21, 128.67, 128.01, 47.48, 36.21, 26.38; HRMS ESI (m/z): $[M+H]^+$ calcd. for $C_{11}H_{13}I_2O$, 414.9050; found, 414.9043.

1-(3-fluorophenyl)-5,5-diiodopentan-1-one (20)

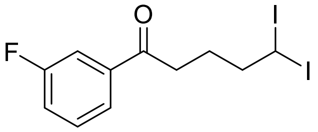

Following the general procedure C afforded the product as a colourless oil (61 mg, 71% yield). ^{1}H NMR (400 MHz, Chloroform-*d*) δ 7.78 – 7.68 (m, 1H), 7.68 – 7.57 (m, 1H), 7.52 – 7.41 (m, 1H), 7.35 – 7.28 (m, 1H), 5.16 (t, *J* = 6.4 Hz, 1H), 3.03 (t, *J* = 7.0 Hz, 2H), 2.59 – 2.37 (m, 2H), 2.00 – 1.79 (m, 2H); ^{13}C NMR (126 MHz, $CDCl_3$) δ 197.70, 163.89, 161.91, 138.75, 138.71, 130.39, 130.33, 123.75, 123.73, 120.33, 120.16, 114.87, 114.69, 47.33, 36.40, 26.23; HRMS ESI (m/z): $[M+H]^+$ calcd. for $C_{11}H_{12}FI_2O$, 432.8956, found 432.8954.

1-(3-(tert-butyl)phenyl)-5,5-diiodopentan-1-one (21)

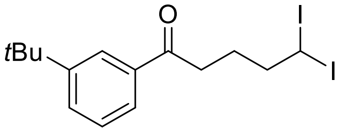

Following the general procedure C afforded the product as a colourless oil (56 mg, 60% yield). ^{1}H NMR (400 MHz, Chloroform-*d*) δ 8.06 – 7.94 (m, 1H), 7.76 – 7.70 (m, 1H), 7.66 – 7.54 (m, 1H), 7.40 (t, *J* = 7.8 Hz, 1H), 5.17 (t, *J* = 6.4 Hz, 1H), 3.05 (t, *J* = 7.0 Hz, 2H), 2.55 – 2.39 (m, 2H), 1.99 – 1.84 (m, 2H); ^{13}C NMR (126 MHz, $CDCl_3$) δ 199.38, 151.84, 136.53, 130.38, 128.37, 125.38, 124.73, 47.53, 36.28, 34.89, 31.27, 26.48; HRMS ESI (m/z): $[M+H]^+$ calcd. for $C_{15}H_{21}I_2O$, 470.9676; found, 470.9681.

5,5-diiodo-1-(4-(trifluoromethyl)phenyl)pentan-1-one (22)

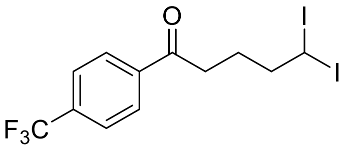

Following the general procedure C afforded the product as a colourless oil (61 mg, 63% yield). ^{1}H NMR (400 MHz, Chloroform-*d*) δ 8.15 – 7.93 (m, 2H), 7.81 – 7.66 (m, 2H), 5.17 (t, *J* = 6.4 Hz, 1H), 3.08 (t, *J* = 7.0 Hz, 2H), 2.59 – 2.41 (m, 2H), 1.95 – 1.84 (m, 2H); ^{13}C NMR (126 MHz, $CDCl_3$) δ 197.95, 139.26, 134.68, 134.42, 128.34, 125.82, 125.79, 125.76, 125.73, 124.63, 122.47, 47.27, 36.56, 26.14; HRMS ESI (m/z): $[M+H]^+$ calcd. for $C_{12}H_{12}F_3I_2O$, 482.8924; found, 482.8918.

7,7-diiodo-1-phenylheptan-1-one (23)

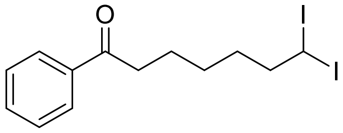

Following the general procedure C afforded the product as a colourless oil (39 mg, 44% yield). ^{1}H NMR (400 MHz, Chloroform-*d*) δ 8.01 – 7.87 (m, 2H), 7.62 – 7.51 (m, 1H), 7.52 – 7.40 (m, 2H), 5.13 (t, *J* = 6.5 Hz, 1H), 2.99 (t, *J* = 7.3 Hz, 2H), 2.43 – 2.29 (m, 2H), 1.86 – 1.70 (m, 2H), 1.52 – 1.34 (m, 4H); ^{13}C NMR (126 MHz, $CDCl_3$) δ 200.08, 137.00, 132.99, 128.60, 128.03, 48.00, 38.25, 31.66, 27.28, 23.92; HRMS ESI (m/z): $[M+H]^+$ calcd. for $C_{13}H_{17}I_2O$, 442.9363; found, 442.9352.

Deconstructive bromo-iodination

General procedure of the deconstructive bromo-iodination

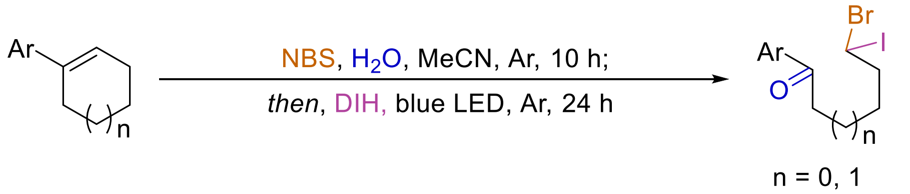

General procedure D:

Alkene (0.2 mmol) and *N*-bromosuccinimide (0.21 mmol, 1 equiv), H_2O (10 mmol, 50 equiv), and MeCN (2 mL) were added to a schlenk tube (10 mL) equipped with a magnetic stirring bar. The reaction mixture was operated by freeze-pump-thaw procedures for three times and backfilled with argon. The resulting solution was magnetically stirred at 50°C for 12 hours. Then, 1,3-diiodo-5,5-dimethylhydantoin (0.3 mmol, 1.5 equiv) was added to the reaction mixture under inert condition. The reaction mixture was irradiated by blue LED lamps (2*40 W) and magnetically stirred at 50 °C. After 24 hours, the reaction solution was concentrated, and the product was purified by column chromatography (SiO_2). The diastereomeric ratio was determined by ^1H NMR of the crude product mixture.

General procedure E:

Alkene (0.2 mmol) and *N*-bromosuccinimide (0.21 mmol, 1 equiv), H_2O (10 mmol, 50 equiv), and MeCN (2 mL) were added to a schlenk tube (10 mL) equipped with a magnetic stirring bar. The reaction mixture was operated by freeze-pump-thaw procedures for three times and backfilled with argon. The resulting solution was magnetically stirred in an ice-bath and slowly warmed to room temperature. After 12 hours, 1,3-diiodo-5,5-dimethylhydantoin (0.3 mmol, 1.5 equiv) was added to the reaction mixture under inert condition. The reaction mixture was irradiated by blue LED lamps (2*40 W) and magnetically stirred at 50 °C. After 24 hours, the reaction solution was concentrated, and the product was purified by column chromatography (SiO_2).

6-bromo-6-iodo-1-phenylhexan-1-one (24)

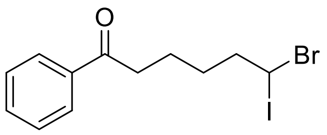

Following the general procedure D afforded the product as a yellow oil (48 mg, 63% yield). ^1H NMR (400 MHz, Chloroform-*d*) δ 7.98 – 7.90 (m, 2H), 7.60 – 7.53 (m, 1H), 7.51 – 7.41 (m, 2H), 5.57 (t, *J* = 6.3 Hz, 1H), 3.02 (t, *J* = 7.2 Hz, 2H), 2.52 – 2.37 (m, 2H), 1.86 – 1.75 (m, 2H), 1.68 – 1.53 (m, 2H); ^{13}C NMR (126 MHz, $CDCl_3$) δ 199.65, 136.89, 133.09, 128.63, 128.01, 46.91, 38.15, 29.61, 22.45, 11.92; HRMS ESI (m/z): $[M+H]^+$ calcd. for $C_{12}H_{15}BrIO$, 380.9345; found, 380.9350.

1-([1,1'-biphenyl]-4-yl)-6-bromo-6-iodohexan-1-one (25)

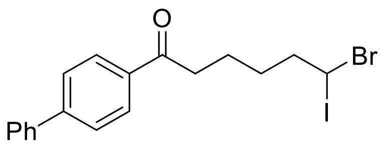

Following the general procedure D afforded the product as a yellow oil (63 mg, 69% yield). ^1H NMR (400 MHz, Chloroform-*d*) δ 8.05 – 8.01 (m, 2H), 7.72 – 7.67 (m, 2H), 7.66 – 7.60 (m, 2H), 7.50 – 7.45 (m, 2H), 7.43 – 7.37 (m, 1H), 5.58 (t, *J* = 6.3 Hz, 1H), 3.05 (t, *J* = 7.2 Hz, 2H), 2.51 – 2.43 (m, 2H), 1.87 – 1.78 (m, 2H), 1.67 – 1.61 (m, 2H); ^{13}C NMR (126 MHz, $CDCl_3$) δ 199.24, 145.78, 139.86, 135.58, 128.97, 128.62, 128.25, 127.27, 46.93, 38.20, 29.64, 22.52, 11.92; HRMS ESI (m/z): $[M+H]^+$ calcd. for $C_{18}H_{19}BrIO$, 456.9658; found, 456.9660.

6-bromo-1-(4-(tert-butyl)phenyl)-6-iodohexan-1-one (26)

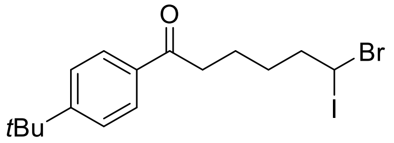

Following the general procedure D afforded the product as a yellow oil (59 mg, 67% yield). ^{1}H NMR (400 MHz, Chloroform-*d*) δ 7.98 – 7.79 (m, 2H), 7.58 – 7.38 (m, 2H), 5.56 (t, *J* = 6.3 Hz, 1H), 2.99 (t, *J* = 7.2 Hz, 2H), 2.44 (tt, *J* = 8.1, 6.5 Hz, 2H), 1.85 – 1.73 (m, 2H), 1.62 – 1.57 (m, 2H), 1.34 (s, 9H); ^{13}C NMR (126 MHz, $CDCl_3$) δ 199.34, 156.82, 134.33, 127.99, 125.56, 46.93, 38.04, 35.12, 31.10, 29.65, 22.55, 11.95; HRMS ESI (m/z): $[M+H]^+$ calcd. for $C_{16}H_{23}IOBr$, 436.9971; found, 436.9967.

6-bromo-6-iodo-1-(4-(trifluoromethyl)phenyl)hexan-1-one (27)

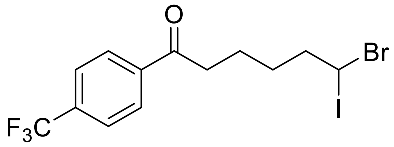

Following the general procedure D afforded the product as a yellow oil (45 mg, 50% yield). ^{1}H NMR (400 MHz, Chloroform-*d*) δ 8.13 – 7.95 (m, 2H), 7.84 – 7.68 (m, 2H), 5.57 (t, *J* = 6.3 Hz, 1H), 3.04 (t, *J* = 7.2 Hz, 2H), 2.49 – 2.36 (m, 2H), 1.90 – 1.74 (m, 2H), 1.64 – 1.59 (m, 2H); ^{13}C NMR (126 MHz, $CDCl_3$) δ 198.54, 139.49, 134.82, 134.56, 134.30, 134.04, 128.32, 125.77, 125.74, 125.71, 125.68, 124.66, 122.49, 46.81, 38.46, 29.49, 22.21, 11.63; HRMS ESI (m/z): $[M+H]^+$ calcd for $C_{13}H_{14}F_3IOBr$, 448.9219; found, 448.9201.

6-bromo-1-(4-fluorophenyl)-6-iodohexan-1-one (28)

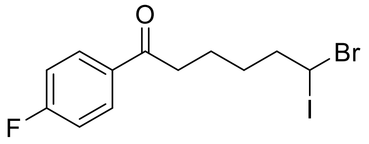

Following the general procedure D afforded the product as a yellow oil (49 mg, 61% yield). ^{1}H NMR (400 MHz, Chloroform-*d*) δ 8.02 – 7.95 (m, 2H), 7.17 – 7.10 (m, 2H), 5.57 (t, *J* = 6.3 Hz, 1H), 2.99 (t, *J* = 7.2 Hz, 2H), 2.49 – 2.40 (m, 2H), 1.85 – 1.74 (m, 2H), 1.64 – 1.57 (m, 2H); ^{13}C NMR (126 MHz, $CDCl_3$) δ 197.97, 166.75, 164.73, 133.30, 130.66, 130.58, 115.80, 115.63, 46.87, 38.06, 29.57, 22.40, 11.81; HRMS ESI (m/z): $[M+H]^+$ $C_{12}H_{14}FIOBr$, 398.9251; found, 398.9260.

6-bromo-1-(3-(tert-butyl)phenyl)-6-iodohexan-1-one (29)

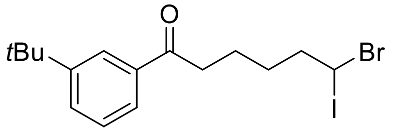

Following the general procedure D afforded the product as a yellow oil (61 mg, 70% yield). ^{1}H NMR (400 MHz, Chloroform-*d*) δ 8.00 (t, *J* = 1.9 Hz, 1H), 7.76 (dt, *J* = 7.7, 1.4 Hz, 1H), 7.66 – 7.58 (m, 1H), 7.40 (t, *J* = 7.7 Hz, 1H), 5.57 (t, *J* = 6.3 Hz, 1H), 3.02 (t, *J* = 7.2 Hz, 2H), 2.53 – 2.39 (m, 2H), 1.80 (dt, *J* = 8.6, 7.0 Hz, 2H), 1.64 – 1.58 (m, 2H), 1.36 (s, 9H); ^{13}C NMR (126 MHz, $CDCl_3$) δ 200.02, 151.77, 136.76, 130.24, 128.32, 125.38, 124.70, 46.95, 38.22, 34.88, 31.27, 29.65, 22.54, 11.94; HRMS ESI (m/z): $[M+Na]^+$ calcd. for $C_{16}H_{22}BrIONa$, 458.9791; found, 458.9779.

6-bromo-1-(3-fluorophenyl)-6-iodohexan-1-one (30)

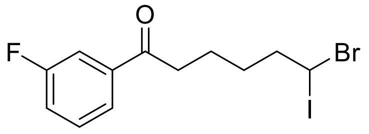

Following the general procedure D afforded the product as a yellow oil (58 mg, 73% yield). ^{1}H NMR (400 MHz, Chloroform-*d*) δ 7.78 – 7.71 (m, 1H), 7.67 – 7.60 (m, 1H), 7.50 – 7.41 (m, 1H), 7.30 – 7.23 (m, 1H), 5.57 (t, *J* = 6.3 Hz, 1H), 3.00 (t, *J* = 7.2 Hz, 2H), 2.49 – 2.40 (m, 2H), 1.84 – 1.76 (m, 2H), 1.65 – 1.58 (m, 2H); ^{13}C NMR (126 MHz, $CDCl_3$) δ 198.29, 163.89, 161.91, 138.94, 130.33, 130.27, 123.74, 123.72, 120.18, 120.01, 114.86, 114.68, 46.85, 38.31, 29.52, 22.30, 11.73; HRMS ESI (m/z): $[M+H]^+$ calcd. for $C_{12}H_{14}FIOBr$, 398.9251; found, 398.9248.

6-bromo-6-iodo-1-(o-tolyl)hexan-1-one (31)

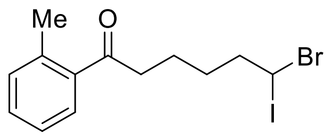

Following the general procedure D afforded the product as a yellow oil (50 mg, 63% yield). ^{1}H NMR (400 MHz, Chloroform-*d*) δ 7.62 (dd, *J* = 7.6, 1.5 Hz, 1H), 7.41 – 7.35 (m, 1H), 7.29 – 7.21 (m, 2H), 5.56 (t, *J* = 6.3 Hz, 1H), 2.93 (t, *J* = 7.2 Hz, 2H), 2.49 (s, 3H), 2.48 – 2.36 (m, 2H), 1.85 – 1.74 (m, 2H), 1.64 – 1.58 (m, 2H); ^{13}C NMR (126 MHz, $CDCl_3$) δ 203.86, 137.99, 132.00, 131.26, 128.30, 125.69, 46.90, 41.11, 29.55, 22.62, 21.29, 11.87; HRMS ESI (m/z): $[M+Na]^+$ calcd. for $C_{13}H_{16}BrIONa$, 416.9321; found, 416.9317.

6-bromo-6-iodo-4-methyl-1-phenylhexan-1-one (32)

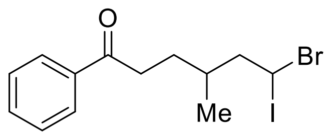

Following the general procedure D afforded the product as a yellow oil (52 mg, 66% yield). *dr* = 1:1. ^{1}H NMR (400 MHz, Chloroform-*d*) δ 7.99 – 7.94 (m, 2H), 7.59 – 7.54 (m, 1H), 7.50 – 7.43 (m, 2H), 5.62 – 5.57 (m, 0.5H), 5.57 – 5.53 (m, 0.5H), 3.05 – 2.97 (m, 2H), 2.55 – 2.44 (m, 1H), 2.42 – 2.34 (m, 0.5H), 2.25 – 2.18 (m, 0.5H), 1.87 – 1.77 (m, 2H), 1.68 – 1.60 (m, 1H), 0.98 (d, *J* = 6.5 Hz, 1.5H), 0.95 (d, *J* = 6.5 Hz, 1.5H); ^{13}C NMR (126 MHz, $CDCl_3$) δ 199.81, 136.89, 133.09, 128.64, 128.03, 54.64, 54.31, 35.84, 33.98, 33.65, 29.93, 29.72, 18.43, 18.17, 10.77, 9.95; HRMS ESI (m/z): $[M+H]^+$ calcd. for $C_{13}H_{17}BrIO$, 394.9502; found, 394.9501.

6-bromo-6-iodo-4-methoxy-1-phenylhexan-1-one (33)

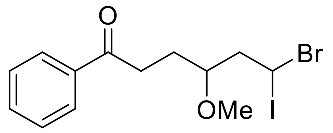

Following the general procedure D afforded the product as a yellow oil (53 mg, 64% yield). *dr* = 1:1. ^{1}H NMR (400 MHz, Chloroform-*d*) δ 8.04 – 7.87 (m, 2H), 7.64 – 7.52 (m, 1H), 7.53 – 7.42 (m, 2H), 5.70 – 5.57 (m, 1H), 3.56 – 3.50 (m, 0.5H), 3.47 – 3.43 (m, 0.5H), 3.39 (s, 1.5H), 3.38 (s, 1.5H), 3.11 – 2.97 (m, 2H), 2.81 – 2.73 (m, 0.5H), 2.56 – 2.45 (m, 1.5H), 2.06 – 1.92 (m, 2H); ^{13}C NMR (126 MHz, $CDCl_3$) δ 199.37, 136.83, 133.16, 128.65, 128.01, 79.60, 78.74, 57.28, 57.11, 52.61, 51.60, 33.34, 33.26, 26.31, 26.18, 8.78, 7.08; HRMS ESI (m/z): $[M+Na]^+$ calcd. for $C_{13}H_{16}BrIO_2Na$, 432.9271; found, 432.9268.

6-bromo-4-(tert-butyl)-6-iodo-1-phenylhexan-1-one (34)

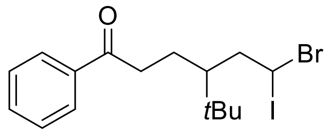

Following the general procedure D afforded the product as a yellow oil (58 mg, 66% yield). *dr* = 1:1. ^{1}H NMR (400 MHz, Chloroform-*d*) δ 7.98 – 7.79 (m, 2H), 7.58 – 7.38 (m, 2H), 5.56 (t, *J* = 6.3 Hz, 1H), 2.99 (t, *J* = 7.2 Hz, 2H), 2.44 (tt, *J* = 8.1, 6.5 Hz, 2H), 1.85 – 1.73 (m, 2H), 1.62 – 1.57 (m, 2H), 1.34 (s, 9H); ^{13}C NMR (126 MHz, $CDCl_3$) δ 199.34, 156.82, 134.33, 127.99, 125.56, 46.93, 38.04, 35.12, 31.10, 29.65, 22.55, 11.95; HRMS ESI (m/z): $[M+Na]^+$ calcd. for $C_{16}H_{22}BrIONa$, 458.9791; found, 458.9779.

6-bromo-6-iodo-1,4-diphenylhexan-1-one (35)

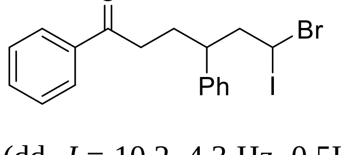

Following the general procedure D afforded the product as a yellow oil (65 mg, 71% yield). *dr* = 1:1. ^{1}H NMR (400 MHz, Chloroform-*d*) δ 7.85 – 7.80 (m, 2H), 7.56 – 7.49 (m, 1H), 7.44 – 7.37 (m, 2H), 7.36 – 7.31 (m, 2H), 7.29 – 7.24 (m, 1H), 7.23 – 7.17 (m, 2H), 5.03 (dd, *J* = 10.3, 3.8 Hz, 0.5H), 4.97 (dd, *J* = 10.2, 4.3 Hz, 0.5H), 2.96 – 2.56 (m, 5H), 2.19 – 2.02 (m, 2H); ^{13}C NMR (126 MHz, $CDCl_3$) δ 199.56, 141.24, 141.15, 136.80, 133.02, 129.08, 129.06, 128.53, 127.94, 127.78, 127.71, 127.69, 127.31, 127.26, 54.59, 53.67, 46.10, 45.51, 36.20, 36.17, 29.91, 11.16, 9.18; HRMS ESI (m/z): $[M+Na]^+$ calcd. for $C_{18}H_{18}BrIONa$, 478.9478; found, 478.9476.

6-bromo-4,4-difluoro-6-iodo-1-phenylhexan-1-one (36)

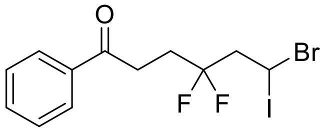

Following the general procedure D afforded the product as a yellow oil (49 mg, 59% yield). ^{1}H NMR (400 MHz, Chloroform-*d*) δ 8.02 – 7.90 (m, 2H), 7.66 – 7.53 (m, 1H), 7.53 – 7.44 (m, 2H), 5.78 – 5.64 (m, 1H), 3.38 – 3.18 (m, 4H), 2.49 – 2.22 (m, 2H); ^{13}C NMR (126 MHz, $CDCl_3$) δ 197.57, 136.38, 133.46, 128.75, 128.73, 128.06, 128.04, 124.85, 122.90, 120.96, 53.83, 53.62, 53.42, 31.22, 31.03, 30.83, 30.74, 30.71, 30.68; HRMS ESI (m/z): $[M+H]^+$ calcd. for $C_{12}H_{13}F_2IOBr$, 416.9157; found, 416.9152.

3-(2-bromo-2-iodoethoxy)-1-phenylpropan-1-one (37)

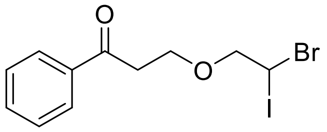

Following the general procedure D afforded the product as a colourless oil (51 mg, 67% yield). ^{1}H NMR (400 MHz, Chloroform-*d*) δ 8.01 – 7.93 (m, 2H), 7.62 – 7.54 (m, 1H), 7.51 – 7.44 (m, 2H), 5.49 (dd, *J* = 6.9, 6.0 Hz, 1H), 4.07 – 3.85 (m, 4H), 3.30 (t, *J* = 6.4 Hz, 2H); ^{13}C NMR (126 MHz, $CDCl_3$) δ 197.92, 136.85, 133.31, 128.65, 128.16, 79.10, 66.50, 38.67, 9.30; HRMS ESI (m/z): $[M+Na]^+$ calcd. for $C_{11}H_{12}BrIO_2Na$, 404.8958; found, 404.8955.

1-(benzo[b]thiophen-2-yl)-6-bromo-6-iodohexan-1-one (38)

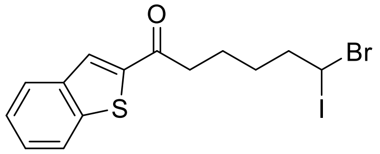

Following the general procedure D afforded the product as a colourless oil (51 mg, 58% yield). ^{1}H NMR (400 MHz, Chloroform-*d*) δ 7.97 (d, *J* = 0.8 Hz, 1H), 7.89 (td, *J* = 7.7, 1.1 Hz, 2H), 7.53 – 7.37 (m, 2H), 5.57 (t, *J* = 6.3 Hz, 1H), 3.06 (t, *J* = 7.3 Hz, 2H), 2.51 – 2.38 (m, 2H), 1.89 – 1.78 (m, 2H), 1.72 – 1.60 (m, 2H); ^{13}C NMR (126 MHz, $CDCl_3$) δ 194.07, 143.57, 142.47, 139.11, 128.89, 127.44, 125.91, 125.04, 123.02, 46.81, 38.82, 29.55, 22.77, 11.68; HRMS ESI (m/z): $[M+Na]^+$ calcd. for $C_{14}H_{14}BrIONaS$, 458.8886; found, 458.8890.

5-bromo-5-iodo-1-phenylpentan-1-one (39)

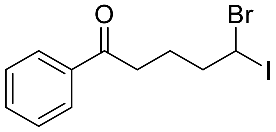

Following the general procedure E afforded the product as a colourless oil (43 mg, 59% yield). ^{1}H NMR (400 MHz, Chloroform-*d*) δ 8.03 – 7.87 (m, 2H), 7.63 – 7.54 (m, 1H), 7.52 – 7.43 (m, 2H), 5.59 (t, *J* = 6.3 Hz, 1H), 3.06 (t, *J* = 7.0 Hz, 2H), 2.57 – 2.43 (m, 2H), 2.03 – 1.88 (m, 2H); ^{13}C NMR (126 MHz, $CDCl_3$) δ 198.99, 136.69, 133.22, 128.68, 128.00, 46.34, 36.53, 24.45, 11.24; HRMS ESI (m/z): $[M+Na]^+$ calcd. for $C_{11}H_{12}BrIONa$, 388.9008; found, 388.8997.

5-bromo-1-(3-fluorophenyl)-5-iodopentan-1-one (40)

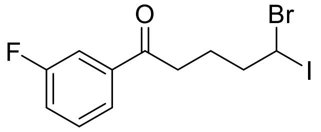

Following the general procedure E afforded the product as a colourless oil (51 mg, 66% yield). ^{1}H NMR (400 MHz, Chloroform-*d*) δ 7.73 (ddd, *J* = 7.7, 1.6, 1.0 Hz, 1H), 7.64 (ddd, *J* = 9.4, 2.7, 1.6 Hz, 1H), 7.46 (td, *J* = 8.0, 5.5 Hz, 1H), 7.31 – 7.25 (m, 1H), 5.59 (t, *J* = 6.3 Hz, 1H), 3.04 (t, *J* = 7.0 Hz, 2H), 2.55 – 2.43 (m, 2H), 2.01 – 1.93 (m, 2H); ^{13}C NMR (126 MHz, $CDCl_3$) δ 197.68, 163.89, 161.92, 138.77, 138.72, 130.39, 130.33, 123.74, 123.72, 120.34, 120.17, 114.86, 114.69, 46.20, 36.73, 24.29, 11.01; HRMS ESI (m/z): $[M+H]^+$ calcd. for $C_{11}H_{12}BrFIO$, 384.9095; found, 384.9092.

Investigation of other trisubstituted alkenes

a) Trialkyl-substituted cyclohexene

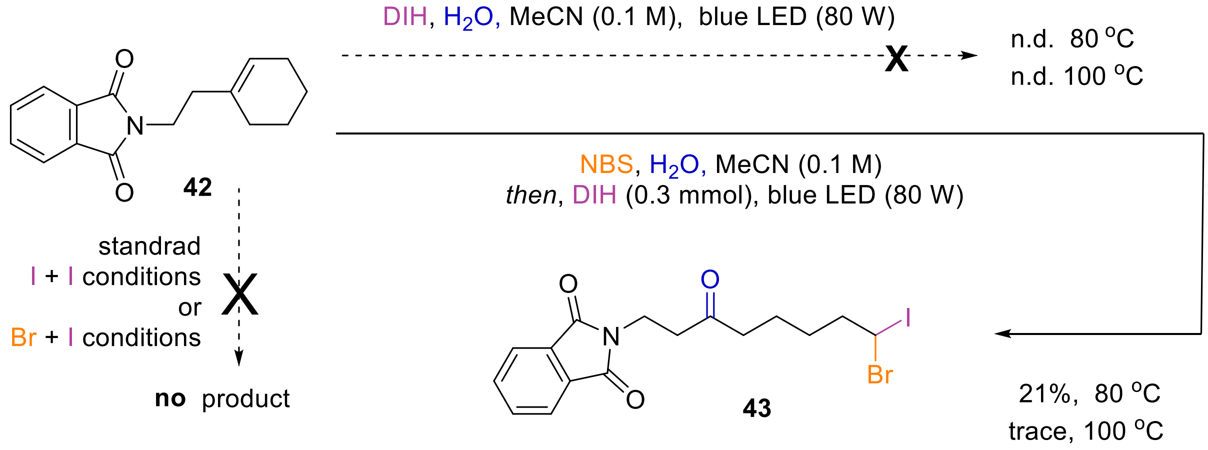

Following general procedure A, treatment of **42** with DIH in the presence of H_2O under blue LED irradiation, could not produce the corresponding diiodination product.

Following general procedure D, treatment of **42** with NBS and DIH in the presence of H_2O under blue LED irradiation, could not produce the corresponding bromo-iodination product.

80 °C conditions:

42 (0.2 mmol) and *N*-bromosuccinimide (0.21 mmol, 1.05 equiv), H_2O (10 mmol, 50 equiv), and MeCN (2 mL) were added to a 10 mL schlenk tube (10 mL) equipped with a magnetic stirring bar. The reaction mixture was operated by freeze-pump-thaw procedures for three times and backfilled with argon. The resulting solution was magnetically stirred at room temperature for 12 hours. Then, 1,3-diiodo-5,5-dimethylhydantoin (0.3 mmol, 1.5 equiv) was added to the reaction mixture, and the reaction mixture was irradiated by blue LED lamps (2*40 W) and magnetically stirred at 80 °C. After 24 hours, the reaction solution was concentrated, and the product was purified by column chromatography (SiO_2). Product **43** was obtained as in 22% yield.

Product **43** was obtained as a colourless solid: ^{1}H NMR (400 MHz, Chloroform-*d*) δ 7.84 – 7.79 (m, 2H), 7.73 – 7.70 (m, 2H), 5.52 (t, *J* = 6.3 Hz, 1H), 3.99 – 3.91 (m, 2H), 2.84 (t, *J* = 7.3 Hz, 2H), 2.48 (t, *J* = 7.2 Hz, 2H), 2.42 – 2.33 (m, 2H), 1.64 – 1.60 (m, 2H), 1.54 – 1.45 (m, 2H); ^{13}C NMR (126 MHz, $CDCl_3$) δ 207.36, 168.10, 134.03, 132.03, 123.31, 46.76, 42.28, 40.71, 33.03, 29.34, 21.74, 11.74; HRMS ESI (m/z): $[M+Na]^+$ calcd. for $C_{16}H_{17}BrNIO_3Na$, 499.9329; found, 499.9333.

Temperature effect on halohydrin formation

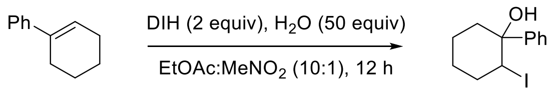

temperature	30 °C	50 °C	80 °C	100 °C
yield	86%	9%	n.d.	n.d.

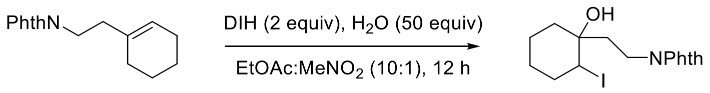

temperature	30 °C	50 °C	80 °C	100 °C
yield	92%	5%	2%	n.d.

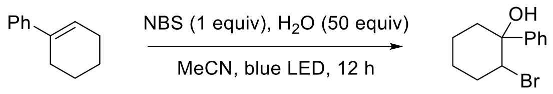

temperature	30 °C	50 °C	80 °C	100 °C
yield	82%	85%	8%	n.d.

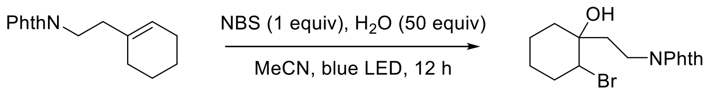

temperature	30 °C	50 °C	80 °C	100 °C
yield	93%	99%	37%	n.d.

Temperature effect on deconstructive iodination

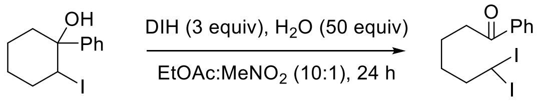

temperature	30 °C	50 °C	80 °C	100 °C
yield	38%	89%	45%	n.d.

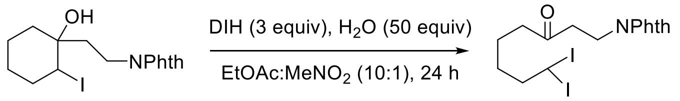

temperature	30 °C	50 °C	80 °C	100 °C
yield	n.d.	2%	n.d.	n.d.

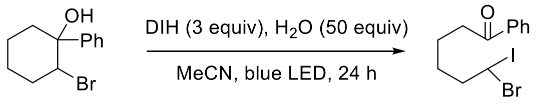

temperature	30 °C	50 °C	80 °C	100 °C
yield	25%	81%	48%	n.d.

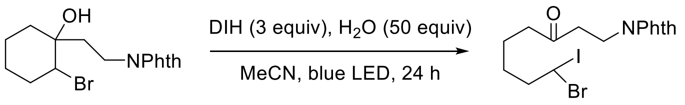

temperature	30 °C	50 °C	80 °C	100 °C
yield	n.d.	9%	48%	4%

Supplementary Figure 2. Evaluation of the effect of temperature on reaction outcomes

The generation and stability of the halohydrin and hypoiodite intermediates are the keys for the success of geminal diiodination and bromo-iodination. Control experiments were performed to evaluate the temperature effect on the halohydrin formation and deconstructive iodination with both aryl substituted and trialkyl substituted cyclic alkenes. As illustrated in Supplementary Figure 2, the formation of halohydrins was sensitive to the reaction temperature, and bromohydrins possessed better thermal stability than that with iodohydrins. On the other hand, higher temperature would accelerate the deconstructive halogenation. However, compared to aryl substituted alcohols, the trialkyl substituted alcohols were not effective in the deconstructive iodination step. A suitable reaction temperature was therefore important to achieve an effective deconstructive geminal dihalogenation.

b) Acyclic trisubstituted alkenes

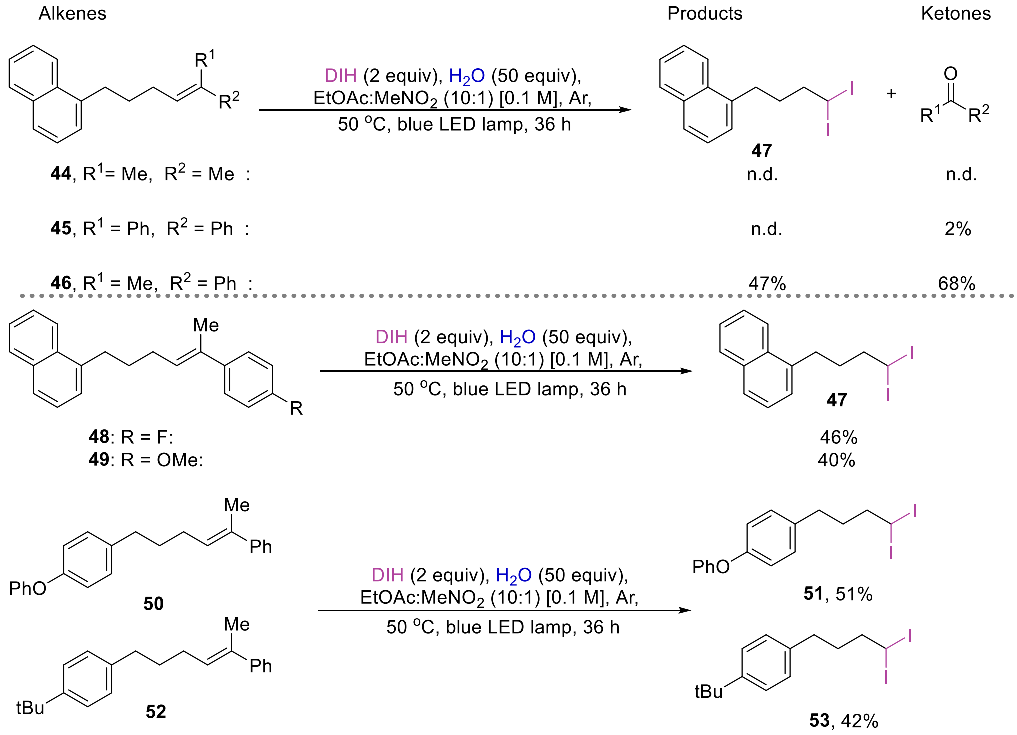

Following general procedure A, alkenes (**44, 45, 46, 48, 49, 50, 52**) (0.1 mmol, 1 equiv) were treated with 1,3-diiodo-5,5-dimethylhydantoin (0.2 mmol, 2 equiv), H_2O (5 mmol, 50 equiv), and EtOAc:$MeNO_2$ (10:1, 1 mL) under the irradiation of blue LED lamps. After 36 hours, the reaction solution was concentrated, and the product was purified by column chromatography (SiO_2). Corresponding products were obtained.

Product **47** was obtained as colorless oil: ^{1}H NMR (400 MHz, Chloroform-*d*) δ 8.10 – 7.97 (m, 1H), 7.93 – 7.83 (m, 1H), 7.77 – 7.71 (m, 1H), 7.56 – 7.46 (m, 2H), 7.43 – 7.38 (m, 1H), 7.36 – 7.28 (m, 1H), 5.14 (t, *J* = 6.5 Hz, 1H), 3.20 – 3.07 (m, 2H), 2.58 – 2.44 (m, 2H), 1.98 – 1.86 (m, 2H); ^{13}C NMR (126 MHz, $CDCl_3$) δ 137.30, 133.93, 131.71, 128.87, 126.97, 126.02, 125.95, 125.56, 125.52, 123.59, 48.09, 32.84, 31.02; HRMS ESI (m/z): $[M+H]^+$ calcd. for $C_{14}H_{15}I_2$, 436.9258; found, 436.9260.

Product **51** was obtained as pale yellow viscous oil: ^{1}H NMR (400 MHz, Chloroform-*d*) δ 7.37 – 7.28 (m, 2H), 7.18 – 7.12 (m, 2H), 7.11 – 7.05 (m, 1H), 7.03 – 6.90 (m, 4H), 5.13 (t, *J* = 6.4 Hz, 1H), 2.71 – 2.58 (m, 2H), 2.46 – 2.34 (m, 2H), 1.81 – 1.72 (m, 2H); ^{13}C NMR (126 MHz, $CDCl_3$) δ 157.50, 155.44, 136.15, 129.69, 129.55, 123.04, 119.07, 118.63, 47.58, 33.54, 33.10; HRMS ESI (m/z): $[M+H]^+$ calcd. for $C_{16}H_{17}I_2O$, 478.9363; found, 478.9370.

Product **53** was obtained as pale yellow viscous oil: ^{1}H NMR (400 MHz, Chloroform-*d*) δ 7.32 (d, *J* = 8.3 Hz, 2H), 7.12 (d, *J* = 8.3 Hz, 2H), 5.12 (t, *J* = 6.5 Hz, 1H), 2.64 (t, *J* = 7.7 Hz, 2H), 2.50 – 2.37 (m, 2H), 1.77 (t, *J* = 7.7 Hz, 2H), 1.31 (s, 9H); ^{13}C NMR (126 MHz, $CDCl_3$) δ 148.93, 138.14, 127.98, 125.35, 47.74, 34.39, 33.45, 33.27, 31.40; HRMS ESI (m/z): $[M+H]^+$ calcd. for $C_{14}H_{21}I_2$, 442.9727; found, 442.9731.

Mechanistic studies

Control experiments

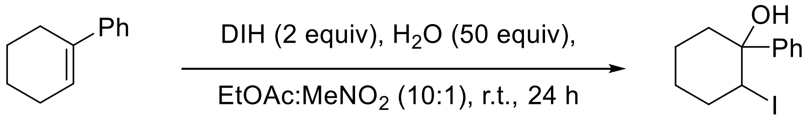

1 **54**

1 (0.1 mmol, 1 equiv), 1,3-diiodo-5,5-dimethylhydantoin (0.2 mmol, 2 equiv), H_2O (5 mmol, 50 equiv), and EtOAc:$MeNO_2$ (10:1, 1 mL) were added to a schlenk tube (10 mL) equipped with a magnetic stirring bar. Then, the reaction mixture was operated by freeze-pump-thaw procedures for three times and backfilled with argon. The resulting solution was magnetically stirred at room temperature. After 24 hours, the reaction solution was concentrated, and the product was purified by column chromatography (SiO_2).

Product **54** was obtained as a white solid (26 mg, 86%). ^{1}H NMR (400 MHz, Chloroform-*d*) δ 7.53 – 7.46 (m, 2H), 7.41 – 7.30 (m, 3.5H), 7.16 – 7.09 (m, 0.4H), 4.73 – 4.64 (m, 1H), 2.79 – 2.68 (m, 1H), 2.40 – 2.29 (m, 1H), 2.15 – 2.06 (m, 2H), 1.87 – 1.80 (m, 2H), 1.72 – 1.64 (m, 2H). The spectroscopic data are in accordance with the reported data.[1]

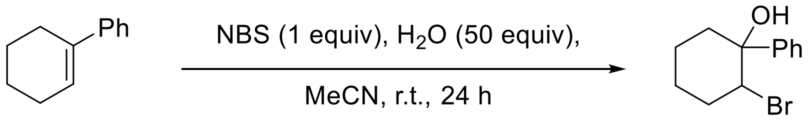

1 **55**

1 (0.1 mmol, 1 equiv), *N*-Bromosuccinimide (0.1 mmol, 1 equiv), H_2O (5 mmol, 50 equiv), and MeCN (1 mL) were added to a schlenk tube (10 mL) equipped with a magnetic stirring bar. Then, the reaction mixture was operated by freeze-pump-thaw procedures for three times and backfilled with argon. The resulting solution was magnetically stirred at room temperature. After 24 hours, the reaction solution was concentrated, and the product was purified by column chromatography (SiO_2).

Product **55** was obtained as a white solid (21 mg, 82%). ^{1}H NMR (400 MHz, Chloroform-*d*) δ 7.54 – 7.48 (m, 2H), 7.40 – 7.35 (m, 2H), 7.33 – 7.29 (m, 1H), 4.47 (td, *J* = 3.7, 2.0 Hz, 1H), 2.70 – 2.58 (m, 1H), 2.52 – 2.42 (m, 1H), 2.09 (dtd, *J* = 14.7, 3.7, 2.1 Hz, 1H), 1.87 – 1.79 (m, 3H), 1.74 – 1.68 (m, 1H), 1.60 (dd, *J* = 8.3, 4.2 Hz, 1H). The spectroscopic data are in accordance with the reported data.[2]

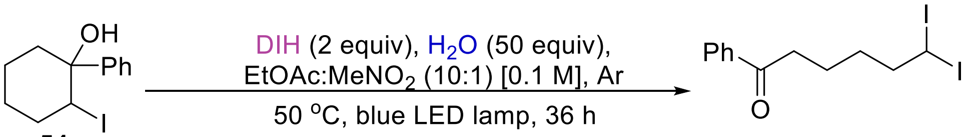

54 **2**, 89%

54 (0.1 mmol, 1 equiv), 1,3-diiodo-5,5-dimethylhydantoin (0.2 mmol, 2 equiv), H_2O (5 mmol, 50 equiv), and EtOAc:$MeNO_2$ (10:1, 1 mL) were added to a schlenk tube (10 mL) equipped with a magnetic stirring bar. Then, the reaction mixture was operated by freeze-pump-thaw procedures for three times and backfilled with argon. The resulting solution was irradiated by blue LED lamps (2*40 W) and magnetically stirred. After 36 hours, the reaction solution was concentrated, and the product was purified by column chromatography (SiO_2).

Product **2** was obtained as a yellow viscous oil (38 mg, 89% yield).

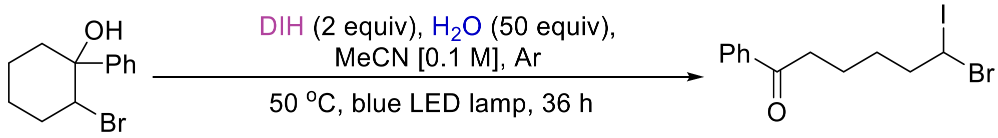

55 **24**, 81%

55 (0.1 mmol, 1 equiv), 1,3-diiodo-5,5-dimethylhydantoin (0.2 mmol, 2 equiv), H_2O (5 mmol, 50 equiv), and MeCN (1 mL) were added to a schlenk tube (10 mL) equipped with a magnetic stirring bar. Then, the reaction mixture was operated by freeze-pump-thaw procedures for three times and backfilled with argon. The resulting solution was irradiated by blue LED lamps (2*40 W) and magnetically stirred. After 36 hours, the reaction solution was concentrated, and the product was purified by column chromatography (SiO_2).

Product **24** was obtained as a yellow oil (31 mg, 81% yield).

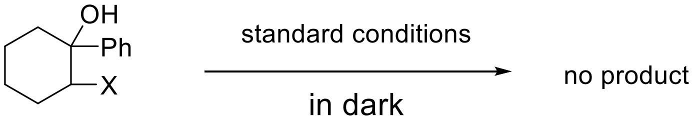

54/55

54 (0.1 mmol, 1 equiv), 1,3-diiodo-5,5-dimethylhydantoin (0.2 mmol, 2 equiv), H_2O (5 mmol, 50 equiv), and EtOAc:$MeNO_2$ (10:1, 1 mL) were added to a schlenk tube (10 mL) equipped with a magnetic stirring bar. Then, the reaction mixture was operated by freeze-pump-thaw procedures for three times and backfilled with argon. The resulting solution was magnetically stirred at 50 °C. After 36 hours, the reaction solution was concentrated.

No product **2** was detected.

55 (0.1 mmol, 1 equiv), 1,3-diiodo-5,5-dimethylhydantoin (0.2 mmol, 2 equiv), H_2O (5 mmol, 50 equiv), and EtOAc:$MeNO_2$ (10:1, 1 mL) were added to a schlenk tube (10 mL) equipped with a magnetic stirring bar. Then, the reaction mixture was operated by freeze-pump-thaw procedures for three times and backfilled with argon. The resulting solution was magnetically stirred at 50 °C. After 36 hours, the reaction solution was concentrated.

No product **24** was detected.

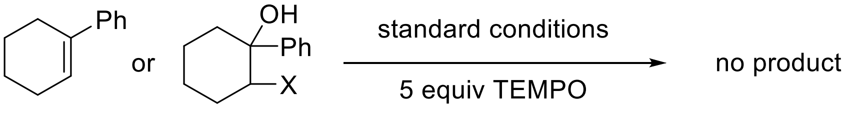

1 **54/55**

1 (0.1 mmol, 1 equiv), 1,3-diiodo-5,5-dimethylhydantoin (0.2 mmol, 2 equiv), H_2O (5 mmol, 50 equiv), TEMPO (0.5 mmol, 5 equiv), and EtOAc:$MeNO_2$ (10:1, 1 mL) were added to a schlenk tube (10 mL) equipped with a magnetic stirring bar. Then, the reaction mixture was operated by freeze-pump-thaw procedures for three times and backfilled with argon. The resulting solution was irradiated by blue LED lamps (2*40 W) and magnetically stirred at 50 °C. After 36 hours, the reaction solution was concentrated.

No product **2** was detected.

54 (0.1 mmol, 1 equiv), 1,3-diiodo-5,5-dimethylhydantoin (0.2 mmol, 2 equiv), H_2O (5 mmol, 50 equiv), TEMPO (0.5 mmol, 5 equiv), and EtOAc:$MeNO_2$ (10:1, 1 mL) were added to a schlenk tube (10 mL) equipped with a magnetic stirring bar. Then, the reaction mixture was operated by freeze-pump-thaw

procedures for three times and backfilled with argon. The resulting solution was irradiated by blue LED lamps (2*40 W) and magnetically stirred at 50 °C. After 36 hours, the reaction solution was concentrated. No product **2** was detected.

55 (0.1 mmol, 1 equiv), 1,3-diiodo-5,5-dimethylhydantoin (0.2 mmol, 2 equiv), H_2O (5 mmol, 50 equiv), TEMPO (0.5 mmol, 5 equiv), and EtOAc:$MeNO_2$ (10:1, 1 mL) were added to a schlenk tube (10 mL) equipped with a magnetic stirring bar. Then, the reaction mixture was operated by freeze-pump-thaw procedures for three times and backfilled with argon. The resulting solution was irradiated by blue LED lamps (2*40 W) and magnetically stirred at 50 °C. After 36 hours, the reaction solution was concentrated. No product **24** was detected.

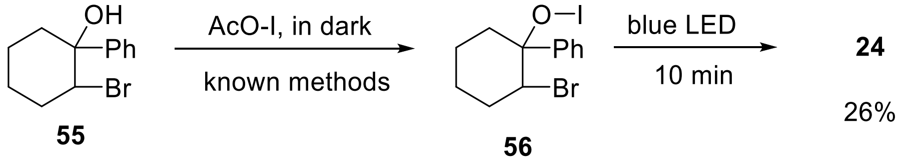

Note: AcOI was prepared following reported method as an acetic acid solution.[3]

55 (0.05 mmol) and $CDCl_3$ (1 mL) were added to a schlenk tube (5 mL) equipped with a magnetic stirring bar. Then, the reaction mixture was operated by freeze-pump-thaw procedures for three times and backfilled with argon. AcOI (0.1 mmol) was added to the reaction mixture and stirred for 10 min in dark. The resulting solution was directly used for NMR and HRMS analysis. Then, the reaction solution was irradiated by blue LED lamps (2*40 W) and magnetically stirred at 50 °C. After 10 min, the reaction solution was concentrated, and the product was purified by column chromatography (SiO_2).
Product **24** was obtained in 26% yield.

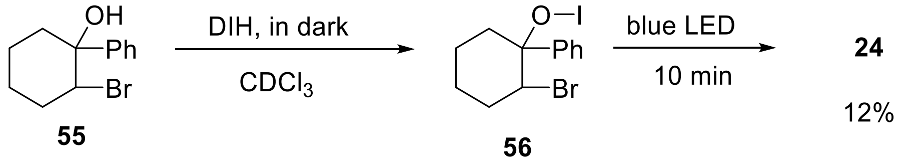

55 (0.05 mmol) and $CDCl_3$ (1 mL) were added to a schlenk tube (5 mL) equipped with a magnetic stirring bar. Then, the reaction mixture was operated by freeze-pump-thaw procedures for three times and backfilled with argon. DIH (0.1 mmol) was added to the reaction mixture and stirred for 10 min in dark. The resulting solution was directly used for NMR and HRMS analysis. Then, the reaction solution was irradiated by blue LED lamps (2*40 W) and magnetically stirred at 50 °C. After 10 min, the reaction solution was concentrated, and the product was purified by column chromatography (SiO_2).
Product **24** was obtained in 12% yield.

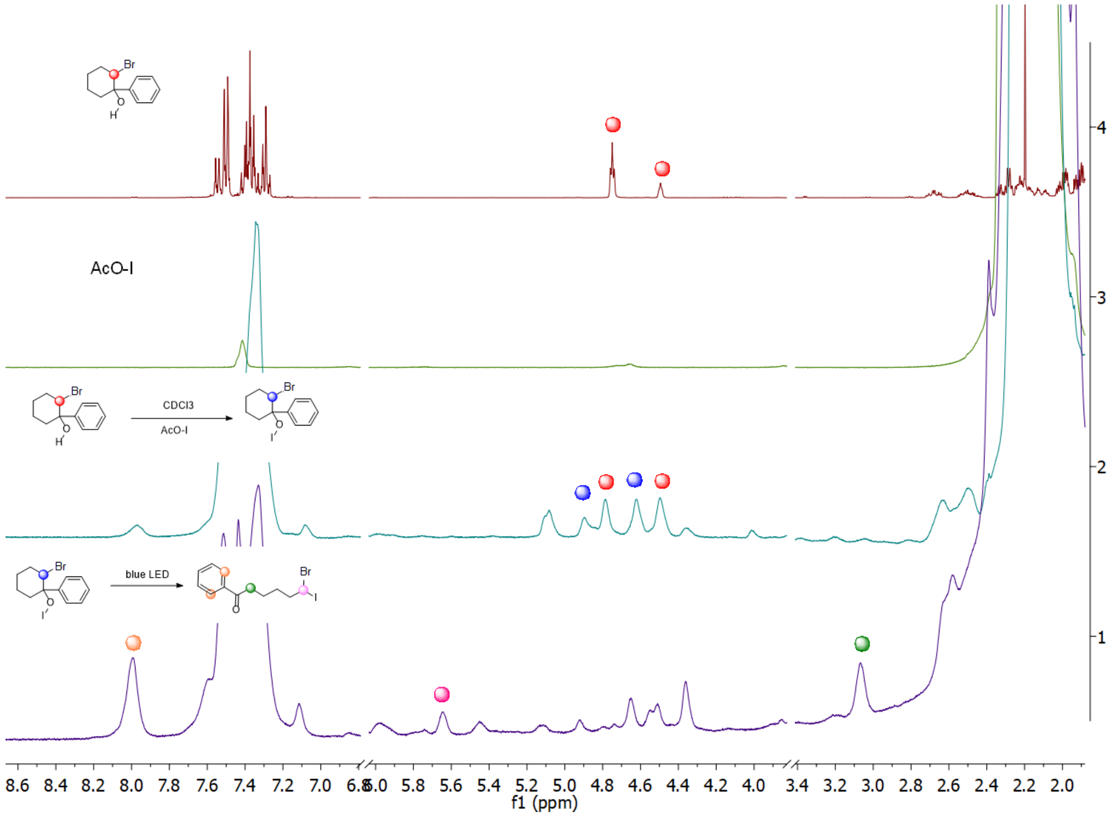

Supplementary Figure 3. ^{1}H-NMR spectra (400MHz, CD3Cl) of a mixture of **55** (1.0 equiv) and AcOI (2.0 equiv).

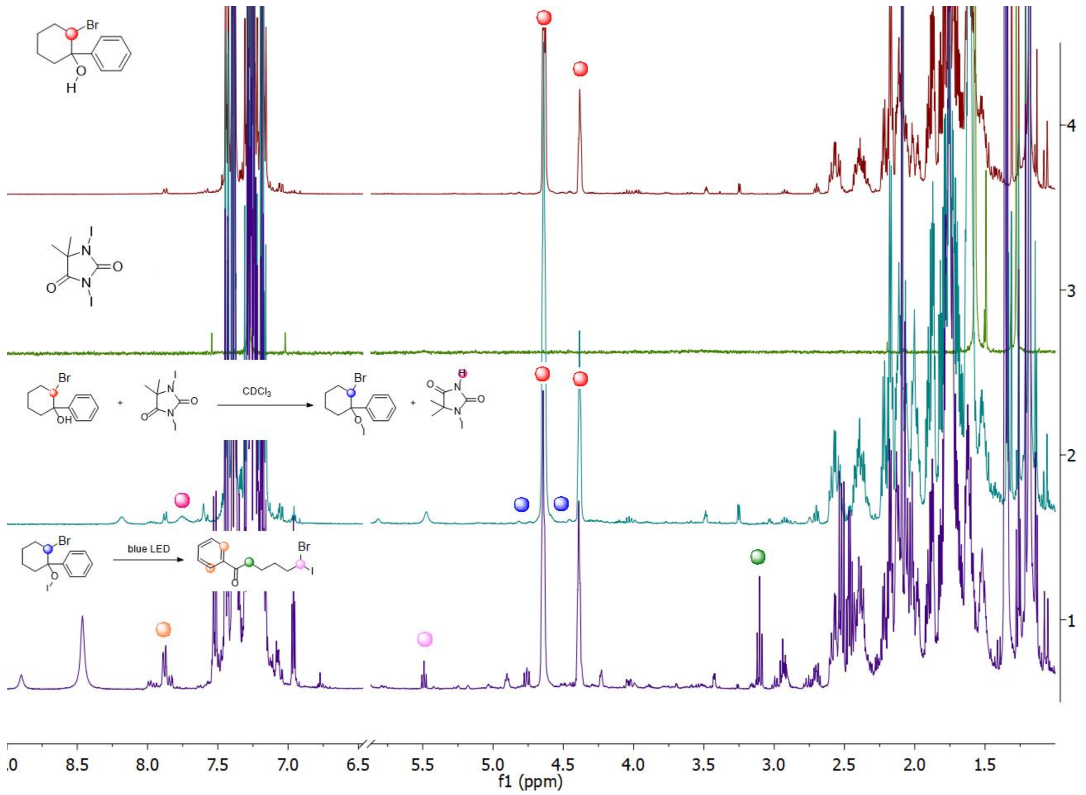

Supplementary Figure 4. ^{1}H-NMR spectra (400MHz, CD3Cl) of a mixture of **55** (1.0 equiv) and DIH (2.0 equiv).

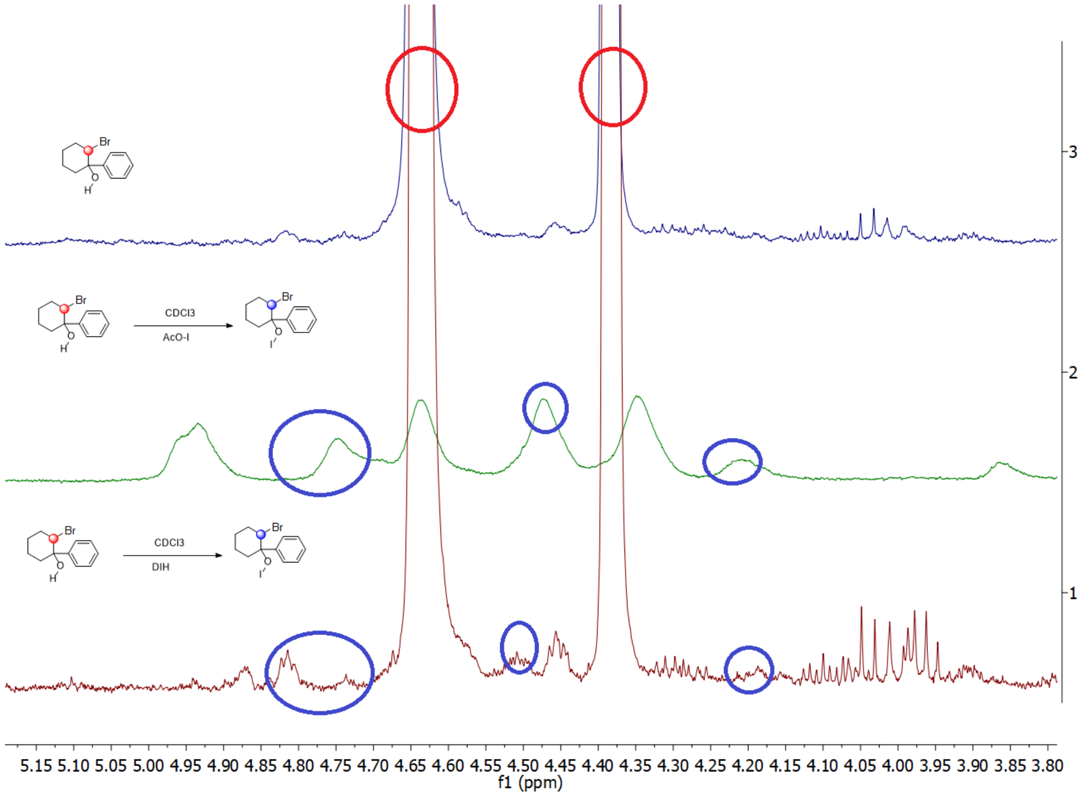

Supplementary Figure 5. ^{1}H-NMR spectra (400MHz, CD_3Cl) for detecting the generation of **56**.

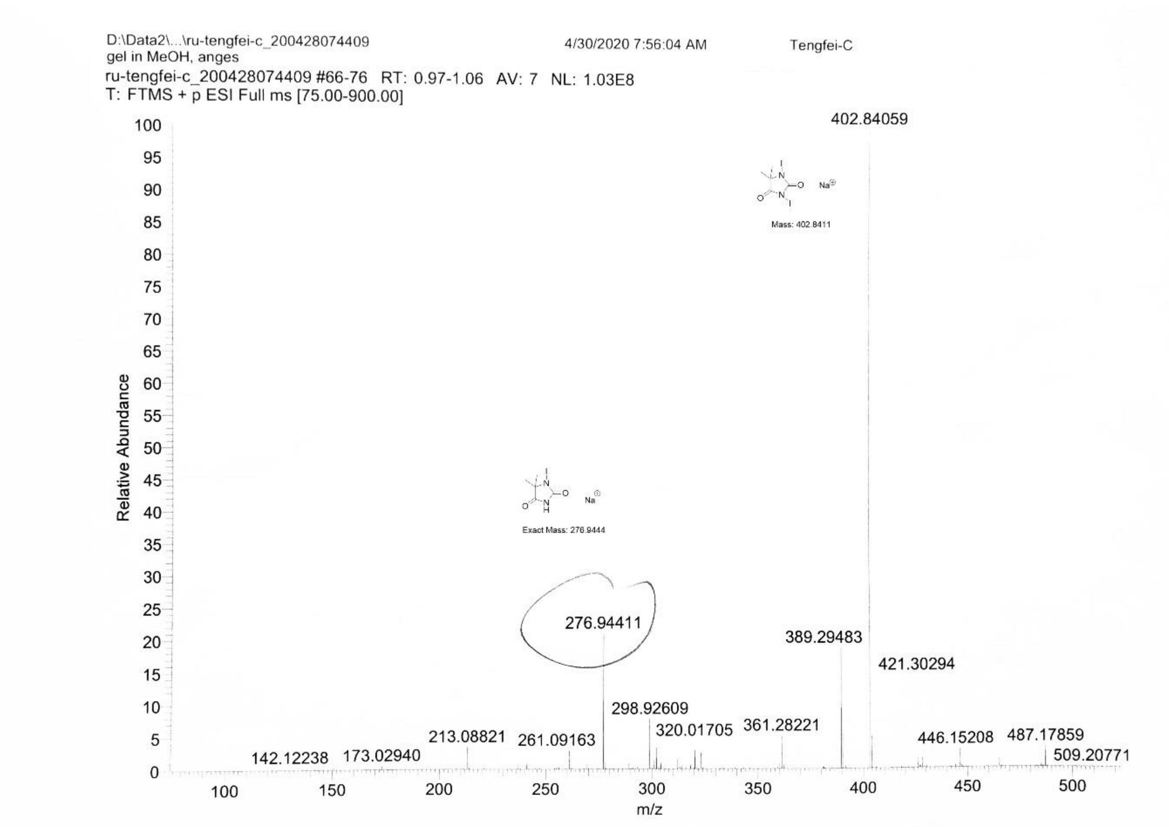

Supplementary Figure 6. Mass spectrum for detecting 1-iodo-5,5-trimethyl hydantoin in the mixture of DIH and **55**

UV-Vis measurement of stoichiometry between alcohol 55 and DIH (450nm)

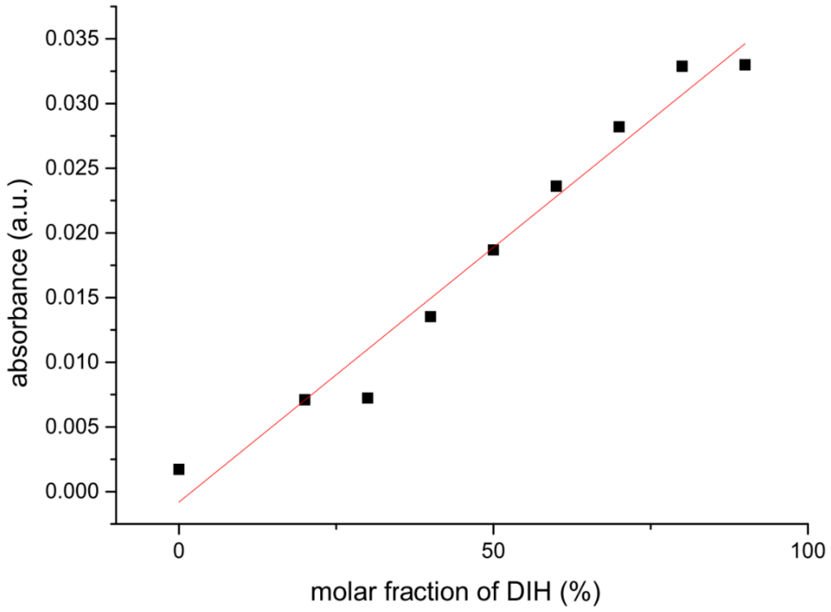

Supplementary Figure 7. UV-Vis measurement of different stoichiometry between alcohol **55** and DIH in MeCN [0.02M] (450nm).

The UV-Vis measurement was performed to evaluate the possible formation of an EDA complex between alcohol **55** and DIH. We measured the absorption of MeCN solutions at 450 nm with different **55**/DIH ratios with constant concentration (0.02 M) of the two components. All the absorption spectra were recorded in 1 cm path quartz cuvettes using an Edinburgh FS-5 Spectrofluorometer. The absorbance values were plotted against the molar fraction (%) of DIH. The absorbance at 50% molar fraction of DIH is not the maximal, which indicates that the formation of an EDA complex is unlikely.

On-off experiments

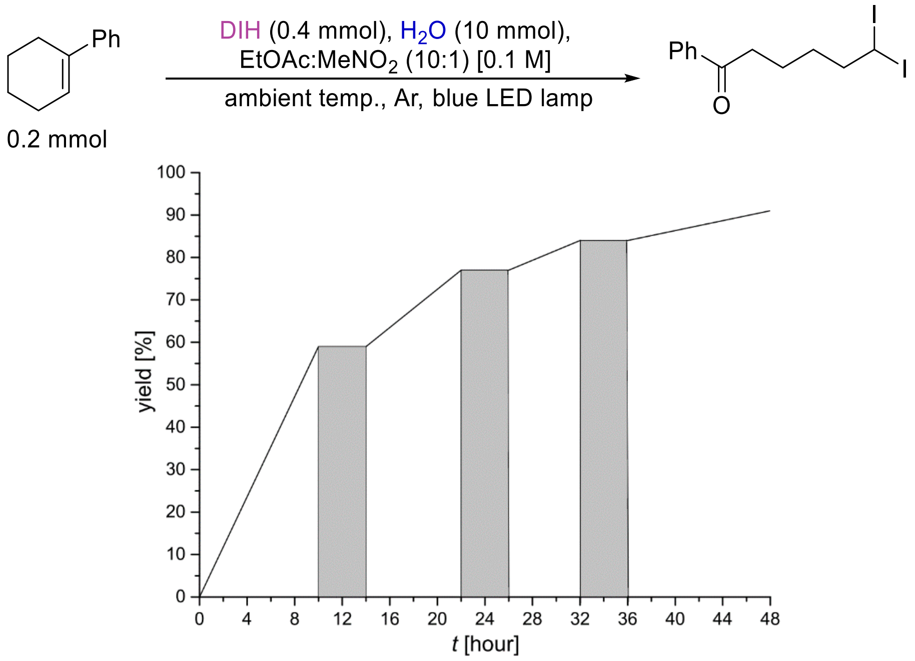

Supplementary Figure 8. Light on-off experiment

Time profile of the transformation with the light ON/OFF over time. Yields were determined by crude ^{1}H NMR spectra using dibromomethane as an internal standard. To examine the impact of light, we conducted experiments under alternating periods of irradiation and darkness following the general procedure A. These resulted in total interruption of the reaction progress in the absence of light and recuperation of reactivity on further illumination. The results demonstrated that light was a necessary component of the reaction. Even though we could not fully rule out a radical-chain process, the data shown that any chain-propagation process must be short-lived.

Electron spin resonance (ESR) spectra

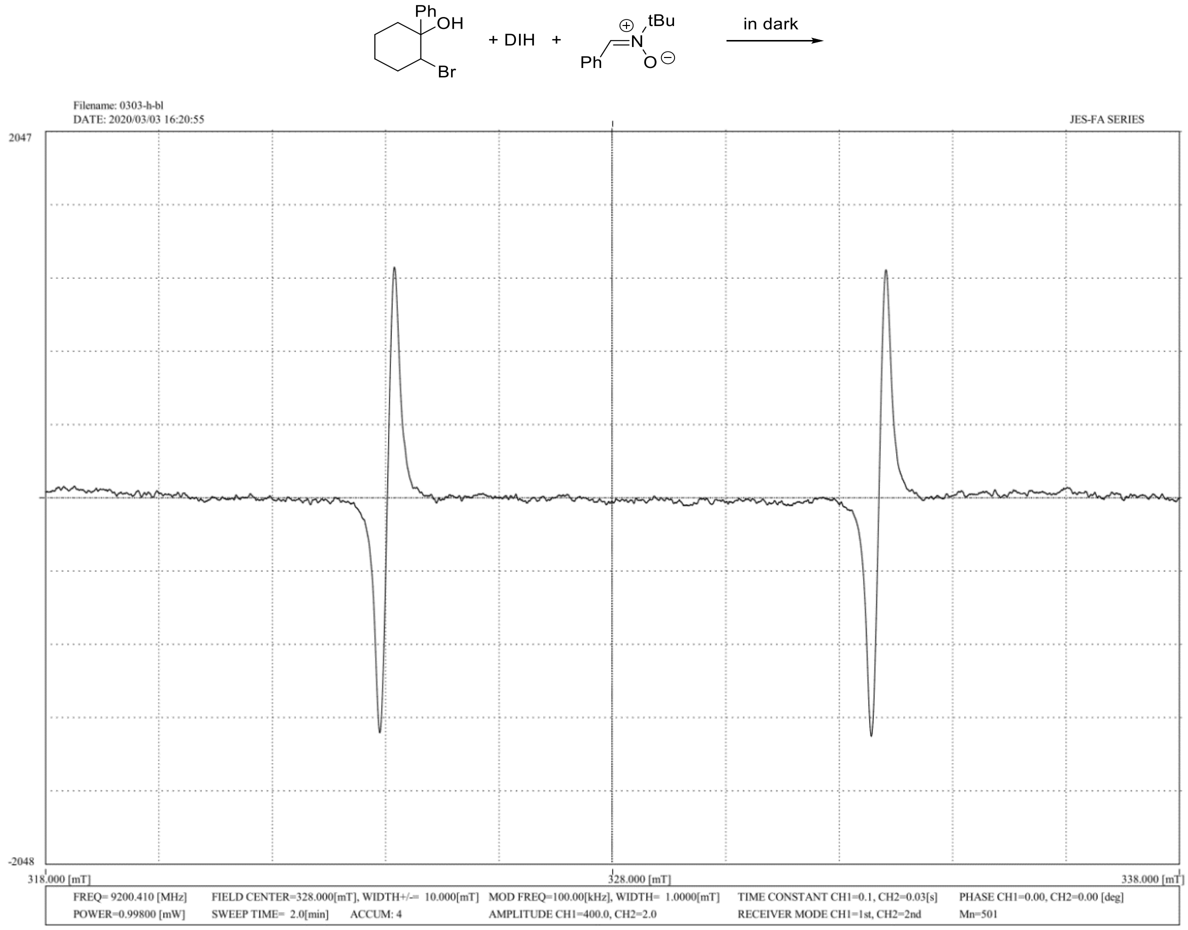

Supplementary Figure 9. ESR spectra observed before irradiation of the MeCN solution at 298 K

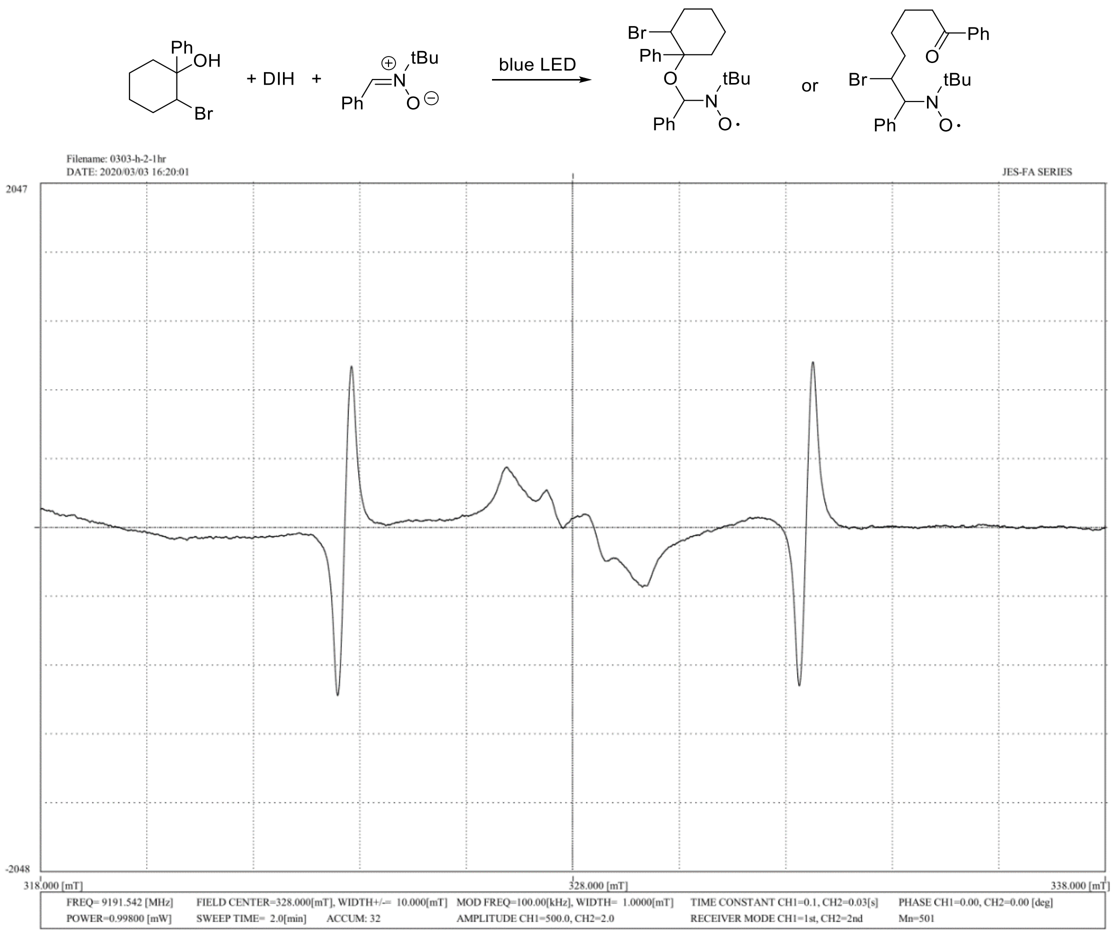

Supplementary Figure 10. ESR spectra observed after irradiation of the MeCN solution at 298 K.

Electron spin resonance (ESR) was conducted to detect the adduct before and after irradiation. The measurement was carried out in an argon saturated MeCN solution of **55** (0.1 M), DIH (0.1 M) and PBN (0.1 M). Before irradiation, the sample cell was stirred for 2 hours and the EPR spectra were recorded (Supplementary Figure 9). Then the sample cavity was irradiated by 80 W blue light for 3 hours at 323 K. After the irradiation, the sample cell was cooled to 298 K and the EPR spectra were recorded (Supplementary Figure 10). The ESR results demonstrated that there is no radical formation in the dark, but radical species was formed after light irradiation.

Gram-scale synthesis and synthetic diversification

Setup of the gram-scale reaction

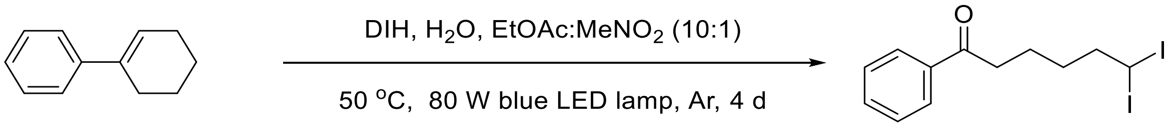

1, 8 mmol **2,** 77%

Following the standard procedure A: **1** (8 mmol, 1 equiv) and 1,3-diiodo-5,5-dimethylhydantoin (16 mmol, 2 equiv), H_2O (400 mmol, 50 equiv), and EtOAc:$MeNO_2$ (10:1, 50 mL) were added to a schlenk tube equipped with a magnetic stirring bar. Then, the reaction mixture was operated by freeze-pump-thaw procedures for three times and backfilled with argon. The resulting solution was irradiated by blue LED lamps (2*40 W) and magnetically stirred at 50 °C. After 4 days, the reaction solution was concentrated, and the product was purified by column chromatography (SiO_2). Product **2** was obtained (2.64g, 77%).

Synthetic diversification

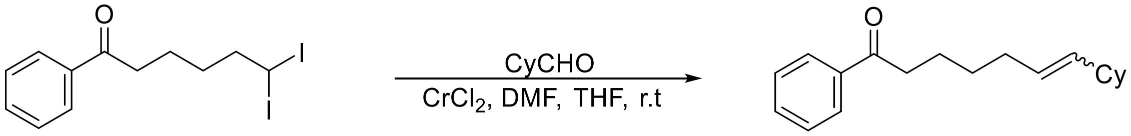

2 **57** E/Z = 3 : 1

To a stirring suspension of anhydrous $CrCl_2$ (98 mg, 0.8 mmol) in THF (2 mL), DMF is added (62 uL, 0.8 mmol) at 25 °C under an argon atmosphere. After 30 min of stirring, a solution of cyclohexanecarboxaldehyde (0.1 mmol) and **2** (0.2 mmol) in THF (0.3 mL) is added at 25 °C. The pale green suspension turns to dark green and then to a dark brown solution. The resulting mixture is stirred at 25 °C for 12 hours. The mixture is subjected to aqueous workup. *E/Z* Ratio was determined by GC analysis of the crude mixture. Purification by column chromatography gave the desired olefin **57**.

Product **57** was obtained as colourless oil (20 mg, 74%). ^1H NMR (400 MHz, Chloroform-*d*) δ 7.98 – 7.93 (m, 2H), 7.59 – 7.53 (m, 1H), 7.49 – 7.43 (m, 2H), 5.39 – 5.30 (m, 2H), 3.00 – 2.93 (m, 2H), 2.08 – 1.99 (m, 2H), 1.93 – 1.85 (m, 1H), 1.79 – 1.65 (m, 6H), 1.49 – 1.41 (m, 2H), 1.23 – 1.05 (m, 4H); ^{13}C NMR (126 MHz, $CDCl_3$) δ 200.52, 136.92, 132.86, 128.58, 128.54, 128.06, 127.09, 40.67, 38.51, 33.25, 32.41, 29.34, 26.13, 23.88; HRMS ESI (m/z): $[M+H]^+$ calcd. for $C_{19}H_{27}O$, 271.2056; found, 271.2055.

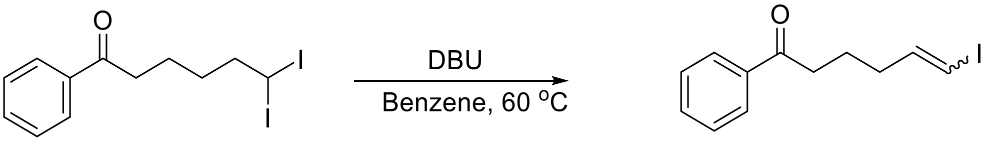

2 **58** *E*/*Z* = 3.5 : 1

2 (0.2 mmol), DBU (0.2 mmol) and benzene (3 mL) were added to a sealed tube equipped with a magnetic stirring bar. The reaction mixture was heated to 60 °C for 4 hours. The mixture is subjected to aqueous workup. *E/Z* Ratio was determined by GC analysis of the crude mixture. Purification by column chromatography gave the desired olefin **58**.

Product **58** was obtained as colourless oil (57 mg, *E/Z* = 3.5:1, 95%). ^1H NMR (400 MHz, Chloroform-*d*) δ 7.99 – 7.91 (m, 2H), 7.60 – 7.52 (m, 1H), 7.50 – 7.42 (m, 2H), 6.53 (dt, *J* = 14.3, 7.2 Hz, 1H), 6.29 – 6.18 (m, 0H), 6.04 (dt, *J* = 14.4, 1.4 Hz, 1H), 3.02 (t, *J* = 7.4 Hz, 0H), 2.97 (t, *J* = 7.2 Hz, 2H), 2.29 – 2.23 (m, 0H), 2.16 (qd, *J* = 7.3, 1.5 Hz, 2H), 1.93 – 1.82 (m, 2H); ^{13}C NMR (126 MHz, $CDCl_3$) δ 199.67,

145.71, 140.53, 136.91, 133.07, 133.01, 128.62, 128.59, 128.03, 128.01, 83.41, 75.46, 37.65, 37.33, 35.41, 34.16, 22.73, 22.39; HRMS ESI (m/z): [M+H]$^+$ calcd. for $C_{12}H_{14}IO$, 301.0084; found, 301.0080.

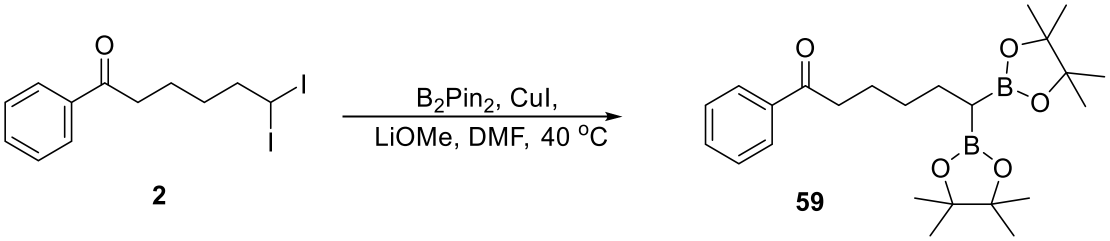

Following reported procedure,[4] CuI (0.02 mmol), LiOMe (0.6 mmol), and bis(pinacolato)diboron (0.44 mmol) were added to a Schlenk tube equipped with a stirring bar. The tube was evacuated and filled with argon (three cycles). DMF (1.2 mL), and **2** (0.2 mmol) were added via syringe under argon atmosphere. The reaction mixture was stirred at 40 °C for 24 h, and then diluted with EtOAc, filtered through silica gel with copious washings (EtOAc) and concentrated. Purification by column chromatography gave the desired olefin **59**.

Product **59** was obtained as colourless oil (62 mg, 72%). ^{1}H NMR (400 MHz, Chloroform-*d*) δ 7.97 – 7.90 (m, 2H), 7.56 – 7.51 (m, 1H), 7.48 – 7.41 (m, 2H), 2.98 – 2.91 (m, 2H), 1.76 – 1.68 (m, 2H), 1.43 – 1.35 (m, 2H), 1.28 – 1.23 (m, 2H), 1.22 (d, *J* = 4.4 Hz, 24H), 0.72 (t, *J* = 7.8 Hz, 1H); ^{13}C NMR (126 MHz, $CDCl_3$) δ 200.66, 137.15, 132.74, 128.48, 128.08, 82.95, 38.62, 32.21, 25.41, 24.87, 24.52, 24.44; HRMS ESI (m/z): [M+H]$^+$ calcd. for $C_{24}H_{39}B_2O_5$, 429.2978; found, 429.2995.

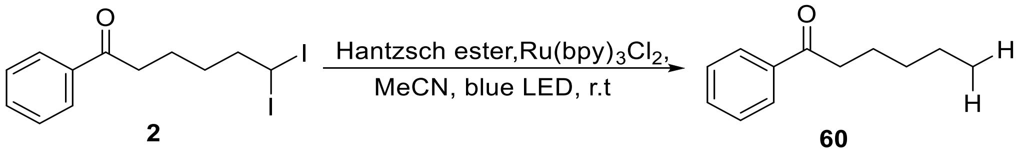

In a glovebox, **2** (0.1 mmol), Hantzsch ester (0.3 mmol), $Ru(bpy)_3Cl_2$ and MeCN (2 mL) were added to a Schlenk tube equipped with a stirring bar. Then the reaction mixture was irradiated by blue LED strips and magnetically stirred. After 24 hours, the reaction solution was concentrated, and the product **60** was purified by column chromatography (SiO_2).

Product **60** was obtained as colourless oil (15 mg, 85%). ^{1}H NMR (400 MHz, Chloroform-*d*) δ 7.98 – 7.92 (m, 2H), 7.58 – 7.52 (m, 1H), 7.50 – 7.43 (m, 2H), 3.00 – 2.93 (m, 2H), 1.79 – 1.70 (m, 2H), 1.39 – 1.33 (m, 4H), 0.95 – 0.86 (m, 3H); ^{13}C NMR (126 MHz, $CDCl_3$) δ 200.63, 137.12, 132.85, 128.54, 128.05, 38.60, 31.57, 24.09, 22.54, 13.96.[5]

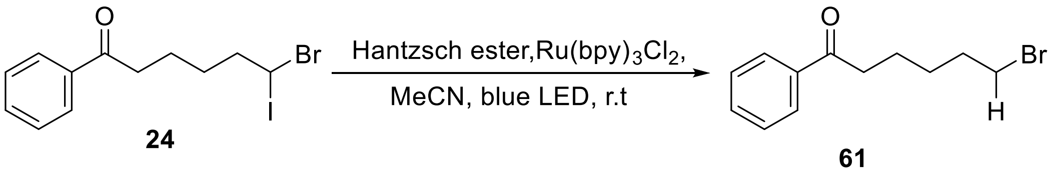

In a glovebox, **24** (0.1 mmol), Hantzsch ester (0.3 mmol), $Ru(bpy)_3Cl_2$ and MeCN (2 mL) were added to a Schlenk tube equipped with a stirring bar. Then the reaction mixture was irradiated by blue LED strips and magnetically stirred. After 24 hours, the reaction solution was concentrated, and the product was purified by column chromatography (SiO_2).

Product **61** was obtained as colourless oil (23 mg, 90%). ^{1}H NMR (400 MHz, Chloroform-*d*) δ 8.02 – 7.82 (m, 2H), 7.60 – 7.54 (m, 1H), 7.52 – 7.41 (m, 2H), 3.43 (t, *J* = 6.8 Hz, 2H), 3.00 (t, *J* = 7.3 Hz, 2H), 2.00 – 1.87 (m, 2H), 1.83 – 1.73 (m, 2H), 1.60 – 1.46 (m, 2H); ^{13}C NMR (126 MHz, $CDCl_3$) δ 199.98, 136.97, 133.00, 128.60, 128.01, 38.29, 33.61, 32.64, 27.89, 23.34; HRMS ESI (m/z): $[M+H]^+$ calcd. for $C_{12}H_{16}BrO$, 255.0379; found, 255.0378.

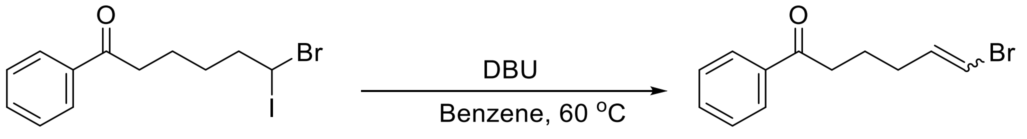

24 **62** *E/Z* = 3.2:1

24 (0.2 mmol), DBU (0.2 mmol) and benzene (3 mL) were added to a sealed tube equipped with a magnetic stirring bar. The reaction mixture was heated to 60°C for 4 hours. The mixture is subjected to aqueous workup. *E/Z* Ratio was determined by GC analysis of the crude mixture. Purification by column chromatography gave the desired olefin **62**.

Product **62** was obtained as colourless oil (48 mg, *E/Z* = 3.2:1, 95%). ^{1}H NMR (400 MHz, Chloroform-*d*) δ 8.00 – 7.91 (m, 2H), 7.63 – 7.53 (m, 1H), 7.50 – 7.43 (m, 2H), 6.24 – 6.05 (m, 2H), 3.05 – 2.95 (m, 2H), 2.32 (qd, *J* = 7.3, 1.4 Hz, 0.5H), 2.16 (qd, *J* = 7.3, 1.3 Hz, 1.5H), 1.95 – 1.81 (m, 2H). ^{13}C NMR (126 MHz, $CDCl_3$) δ 199.65, 137.28, 136.92, 134.09, 133.07, 132.99, 128.62, 128.58, 128.02, 128.00, 108.69, 105.10, 37.67, 37.35, 32.34, 29.20, 22.97, 22.57. HRMS ESI (m/z): $[M+H]^+$ calcd. for $C_{12}H_{14}BrO$, 253.0223; found, 253.0214.

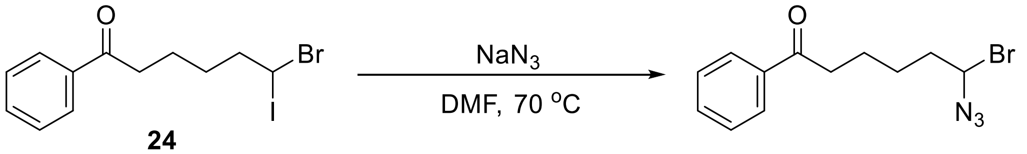

24 **63**

24 (0.2 mmol), NaN_3 (0.8 mmol) and DMF (2 mL) were added to a sealed tube equipped with a magnetic stirring bar. The reaction mixture was heated to 70 °C for 18 hours. Purification by column chromatography gave the product **63**.

Product **63** was obtained as a white solid (51 mg, 86%). ^{1}H NMR (400 MHz, Chloroform-*d*) δ 8.01 – 7.89 (m, 2H), 7.65 – 7.53 (m, 1H), 7.50 – 7.43 (m, 2H), 4.66 (t, *J* = 6.6 Hz, 1H), 3.00 (t, *J* = 7.2 Hz, 2H), 1.87 – 1.72 (m, 4H), 1.56 – 1.43 (m, 2H); ^{13}C NMR (126 MHz, $CDCl_3$) δ 199.65, 136.88, 133.09, 128.63, 128.00, 77.89, 38.09, 33.98, 24.63, 23.38; HRMS ESI (m/z): $[M+H]^+$ calcd. for $C_{12}H_{15}BrN_3O$, 296.0393; found, 296.0392.

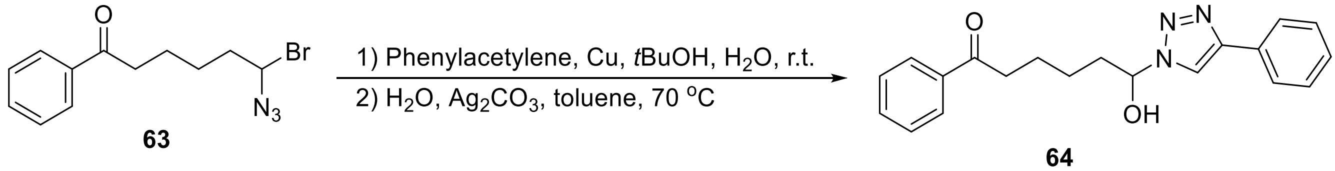

63 **64**

Following reported procedure,[6] **63** (0.2 mmol) and phenylacetylene (0.2 mmol) were suspended in 4 mL of a 1:1 water/tert-butanol mixture. Sodium ascorbate (0.02 mmol, in 0.2mL water) was added, followed by copper(II) sulfate pentahydrate (0.01 mmol, in 0.1mL water). The heterogeneous mixture was stirred vigorously overnight at ambient temperature. The reaction mixture was diluted with 20 mL of

water and cooled in ice to obtain white precipitate which was collected by filtration and used directly in next step.
The white solid, which was obtained from above reaction, was added to a solution of H_2O (10 mmol) and Ag_2CO_3 (0.2 mmol) in toluene (4 mL). Then, the reaction mixture was stirred at 70 °C. After 10 hours, the reaction was concentrated, and the corresponding product was purified by column chromatography.

Product **64** was obtained as a white solid (46 mg, 69%). ^{1}H NMR (400 MHz, Chloroform-*d*) δ 7.96 – 7.81 (m, 5H), 7.59 – 7.52 (m, 1H), 7.49 – 7.41 (m, 4H), 7.39 – 7.31 (m, 1H), 6.00 (t, *J* = 7.2 Hz, 1H), 2.99 (t, *J* = 7.1 Hz, 2H), 2.23 – 2.15 (m, 2H), 1.87 – 1.80 (m, 2H), 1.57 – 1.37 (m, 2H); ^{13}C NMR (126 MHz, $CDCl_3$) δ 199.51, 148.51, 136.78, 133.15, 130.01, 128.93, 128.65, 128.58, 127.98, 125.87, 117.18, 75.43, 37.95, 34.50, 24.64, 23.16; HRMS ESI (m/z): $[M+H]^+$ calcd. for $C_{20}H_{22}N_3O_2$, 336.1707; found, 336.1714.

Supplementary Note 1

NMR Spectra

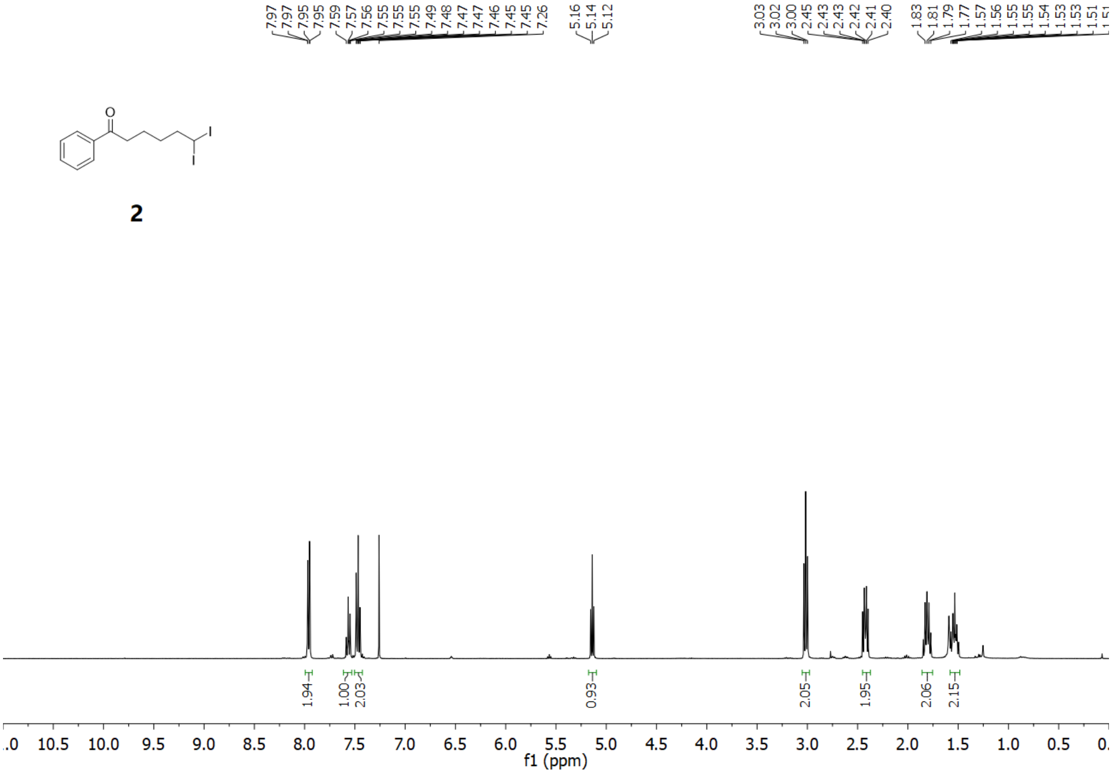

2

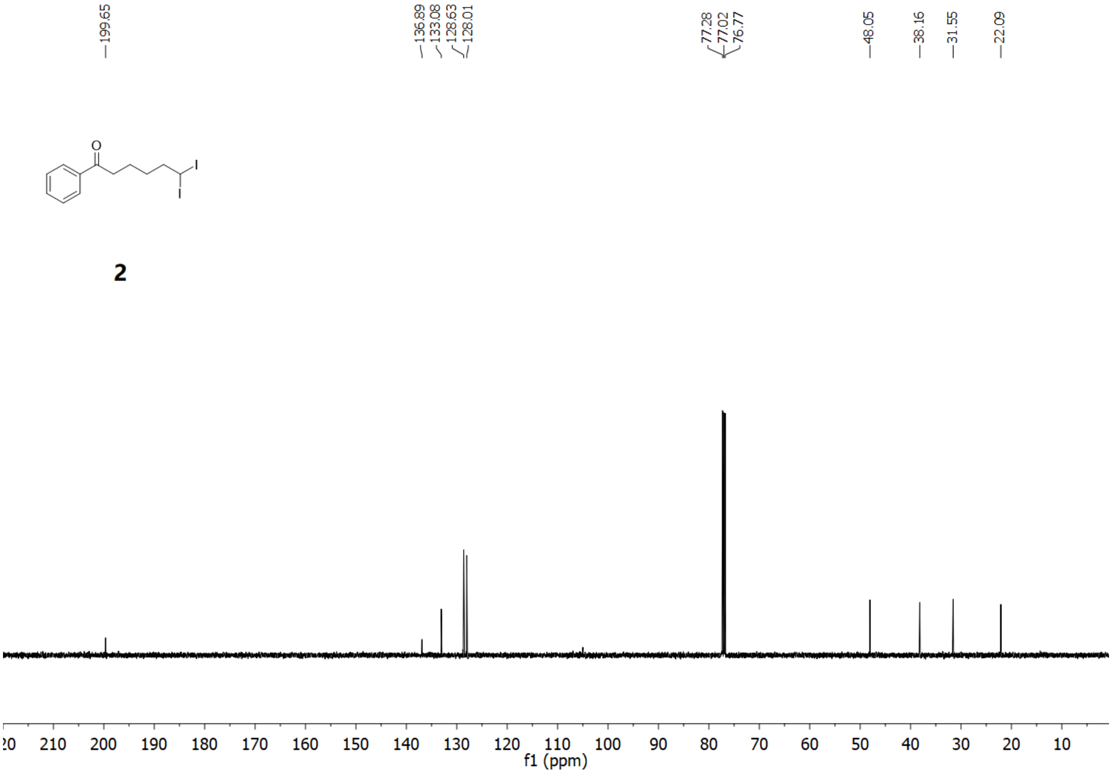

2

Supplementary Figure 11. ^{1}H and ^{13} C NMR spectra for compound **2**

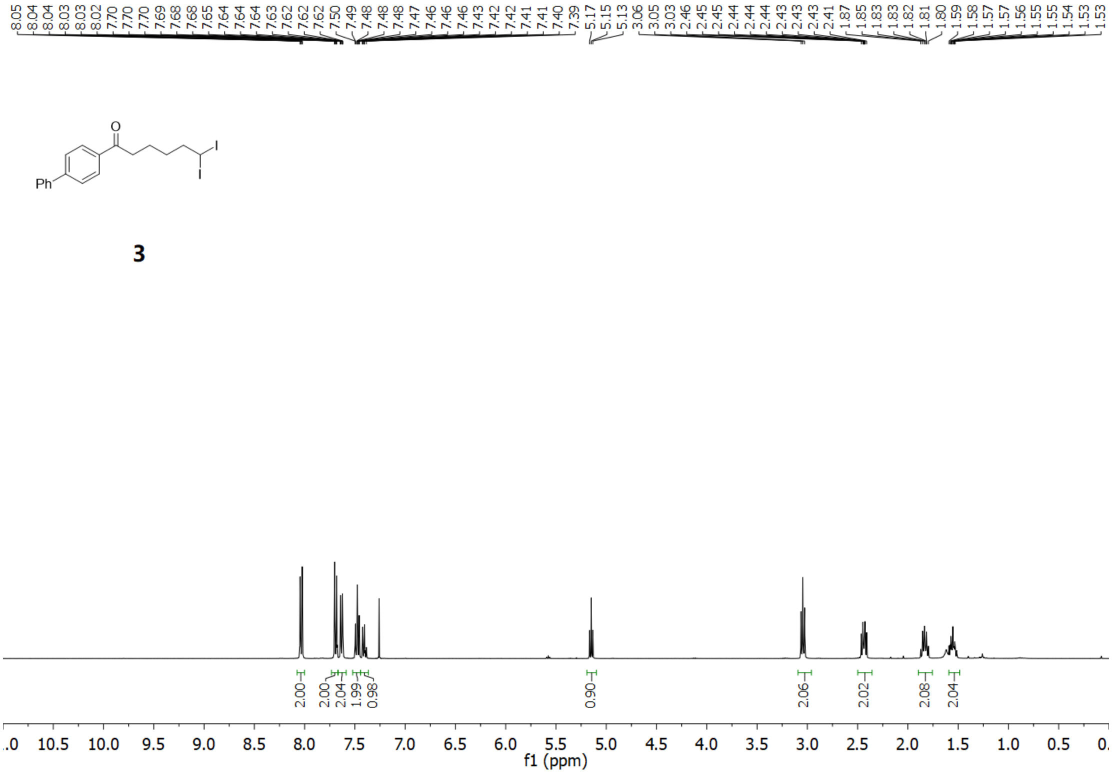

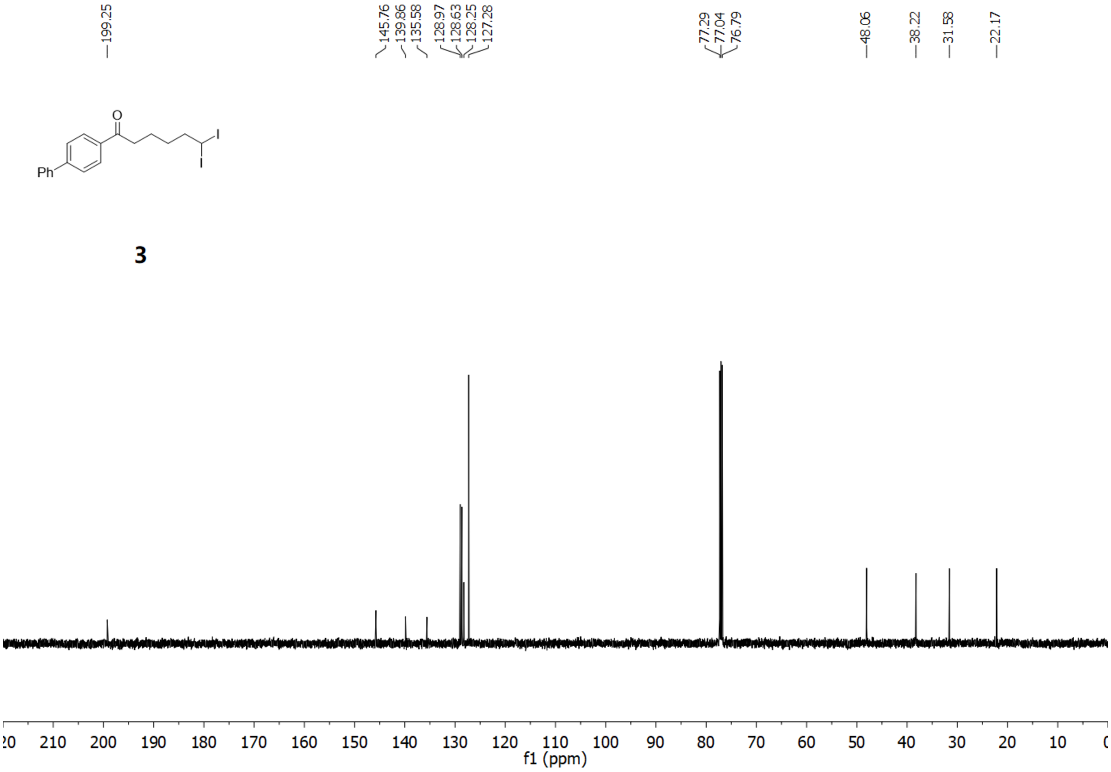

Supplementary Figure 12. ^{1}H and ^{13} C NMR spectra for compound **3**

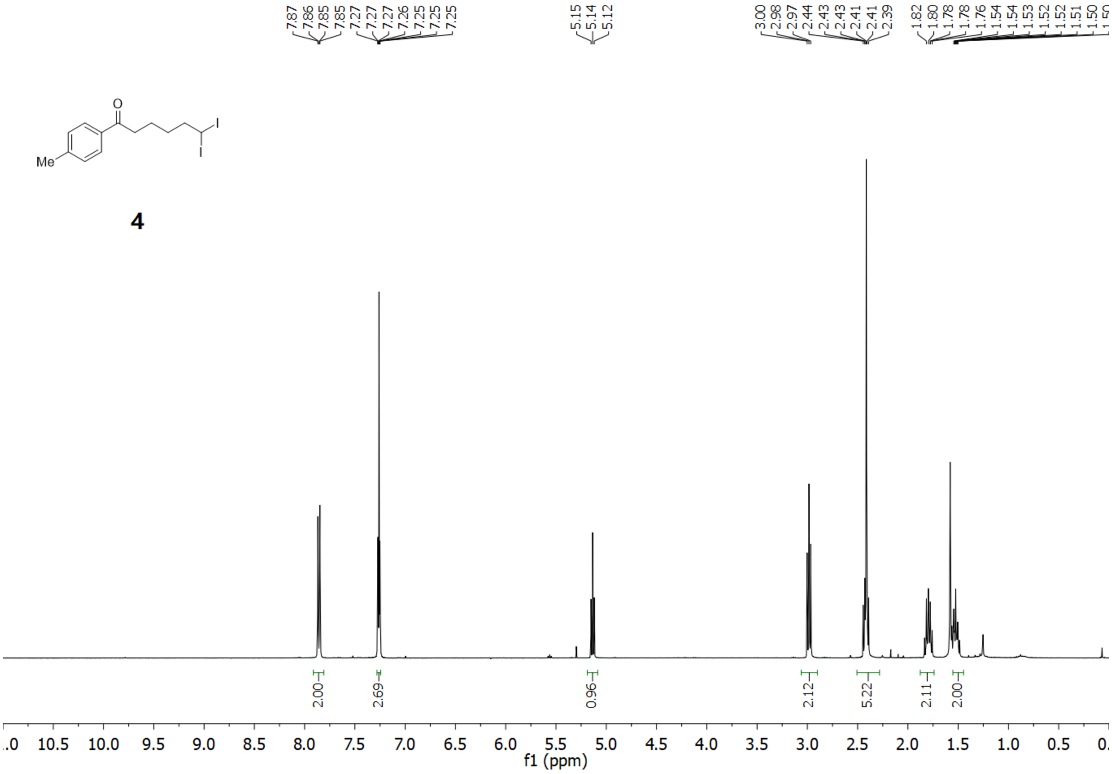

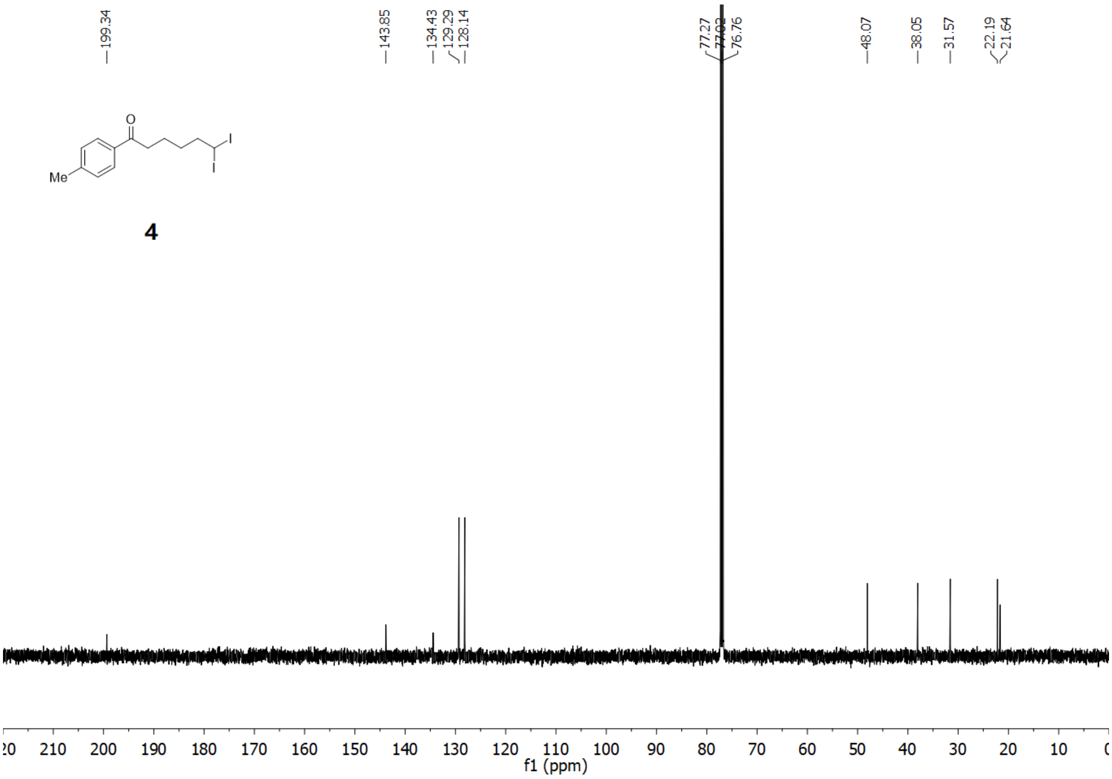

Supplementary Figure 13. ^{1}H and ^{13}C NMR spectra for compound **4**

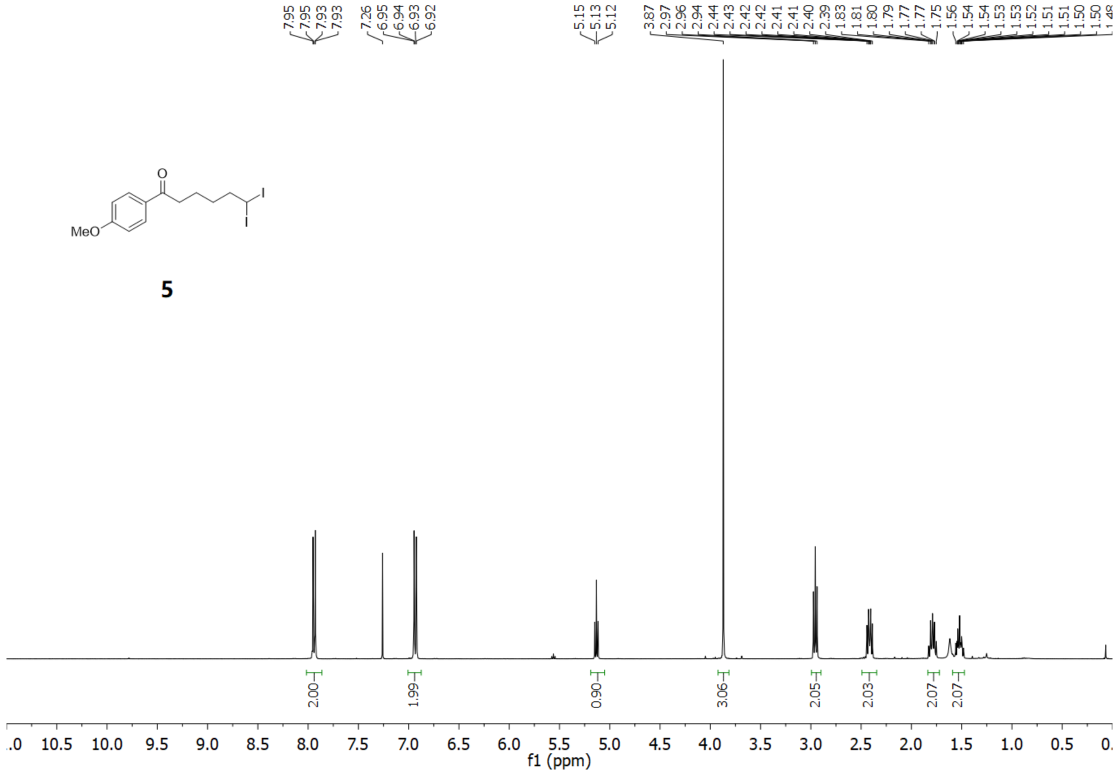

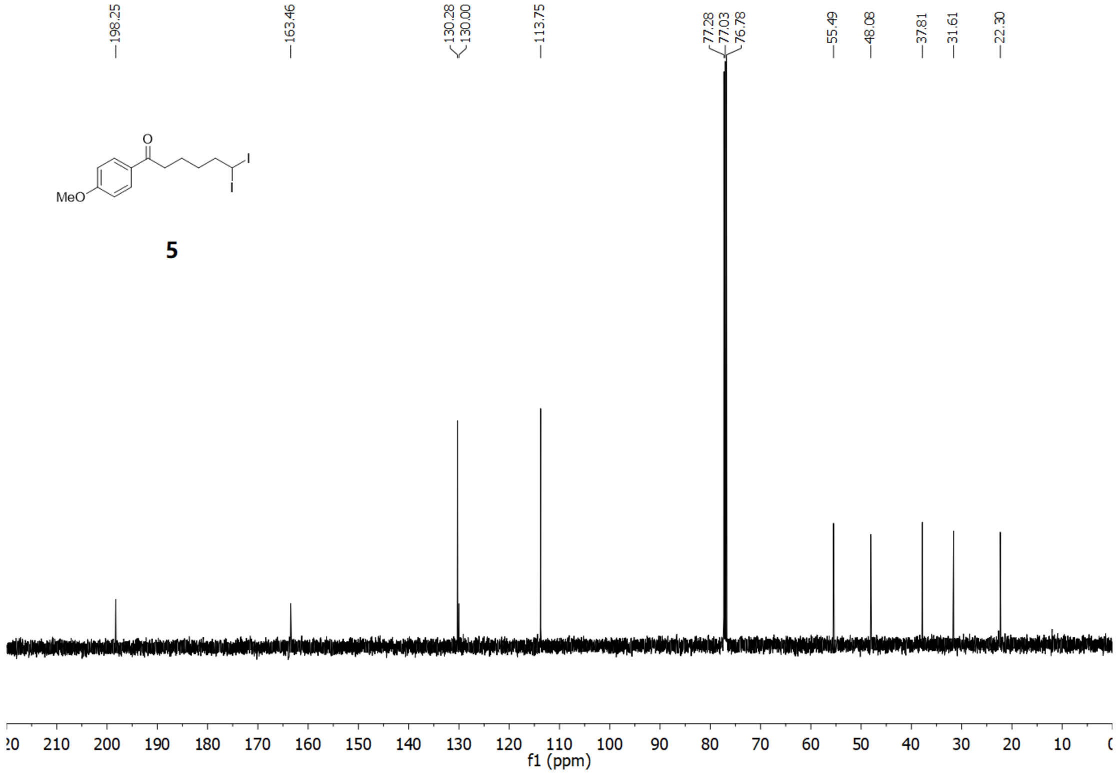

Supplementary Figure 14. ^{1}H and ^{13}C NMR spectra for compound **5**

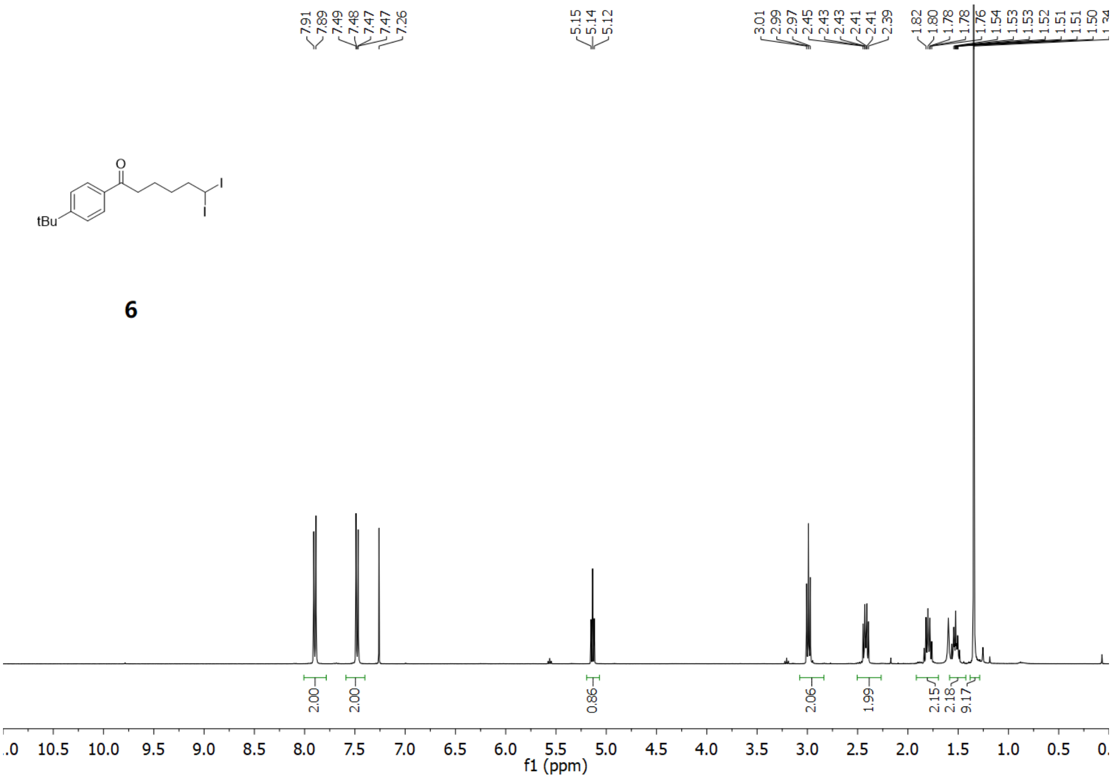

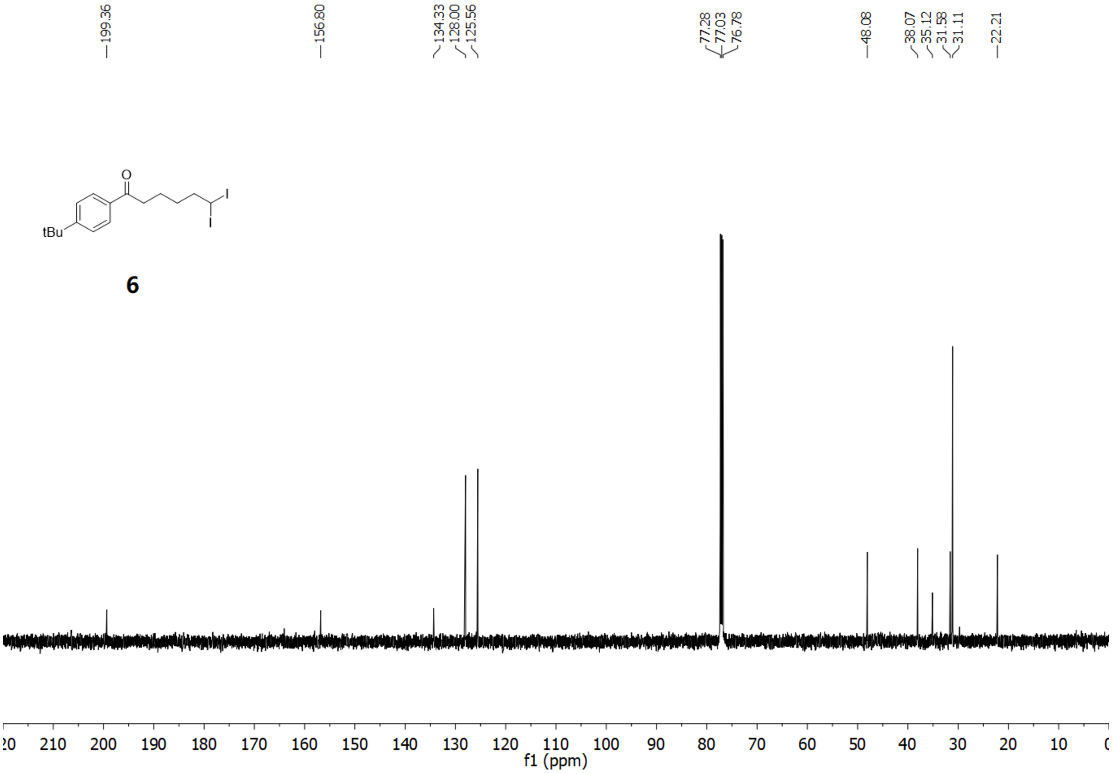

Supplementary Figure 15. ^{1}H and ^{13}C NMR spectra for compound **6**

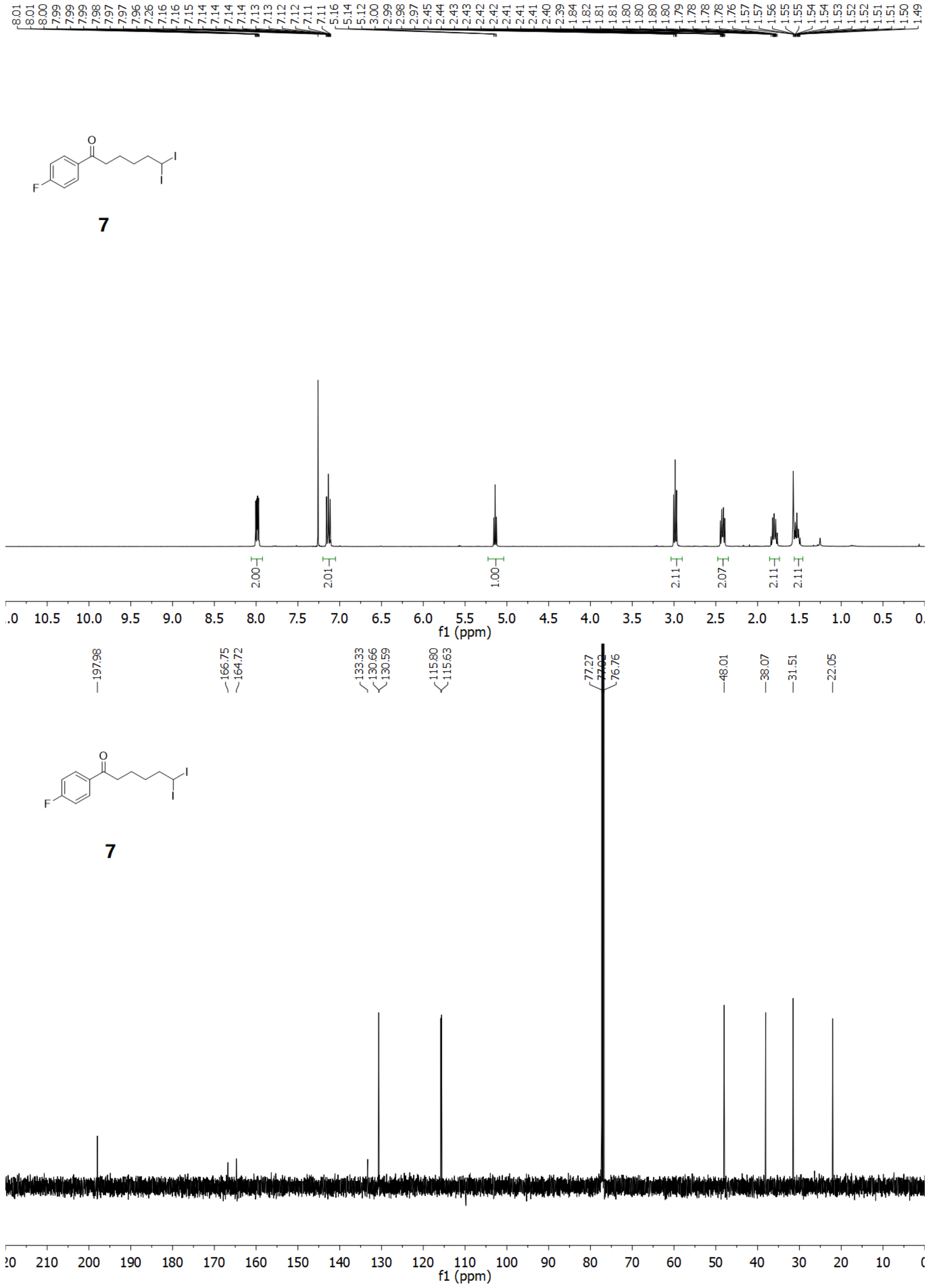

Supplementary Figure 16. ^{1}H and ^{13}C NMR spectra for compound **7**

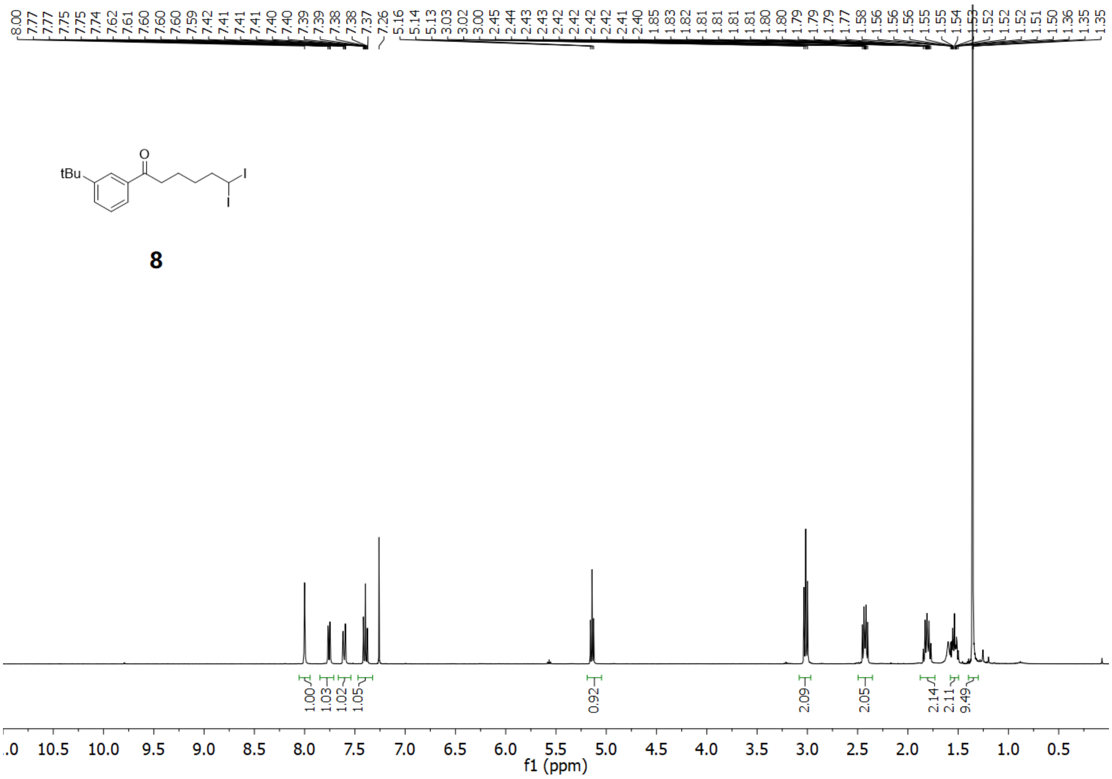

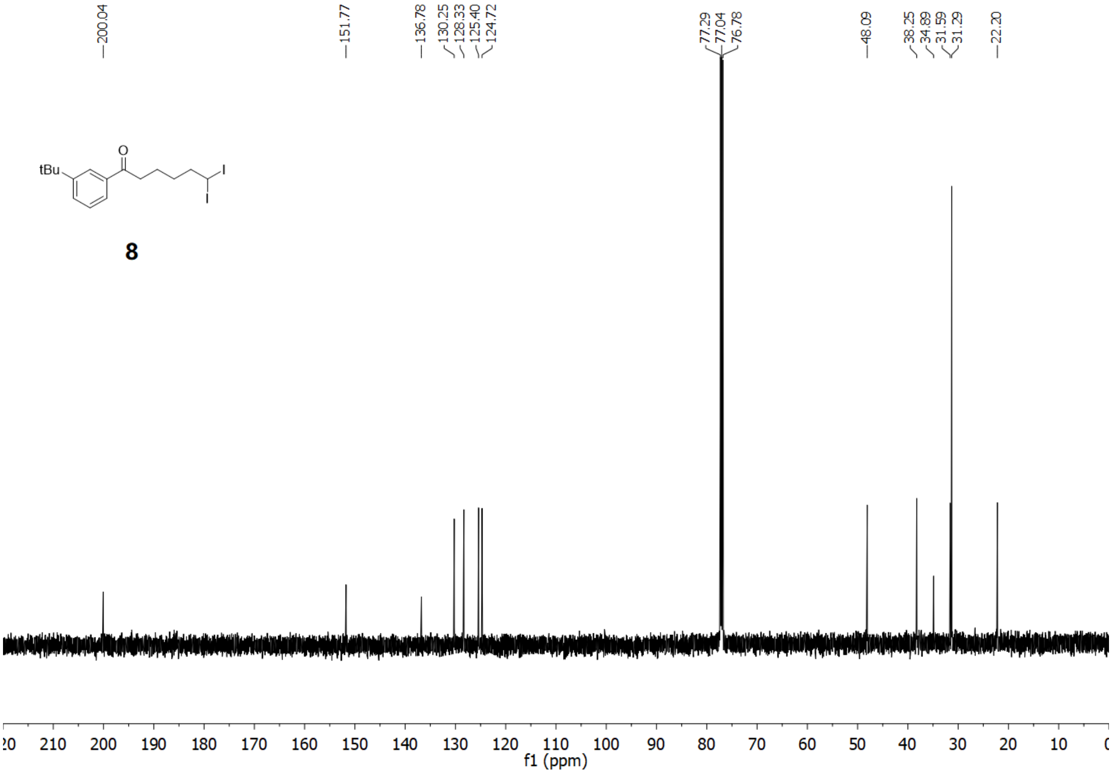

Supplementary Figure 17. ^{1}H and ^{13}C NMR spectra for compound **8**

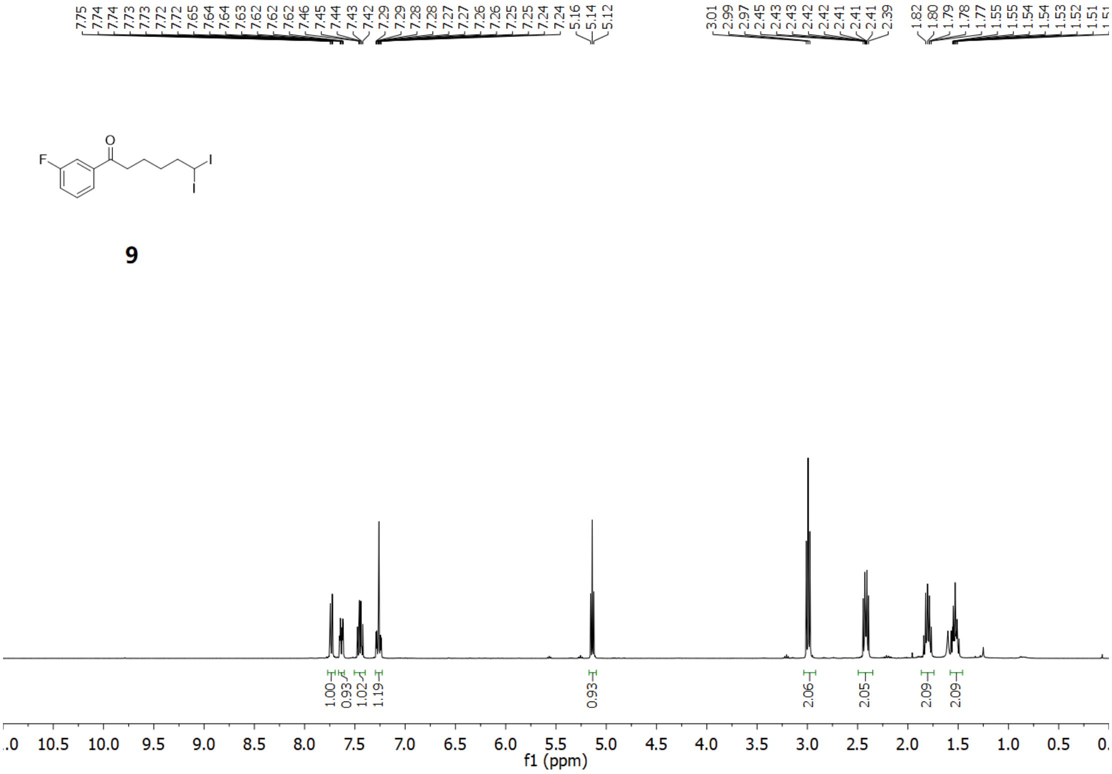

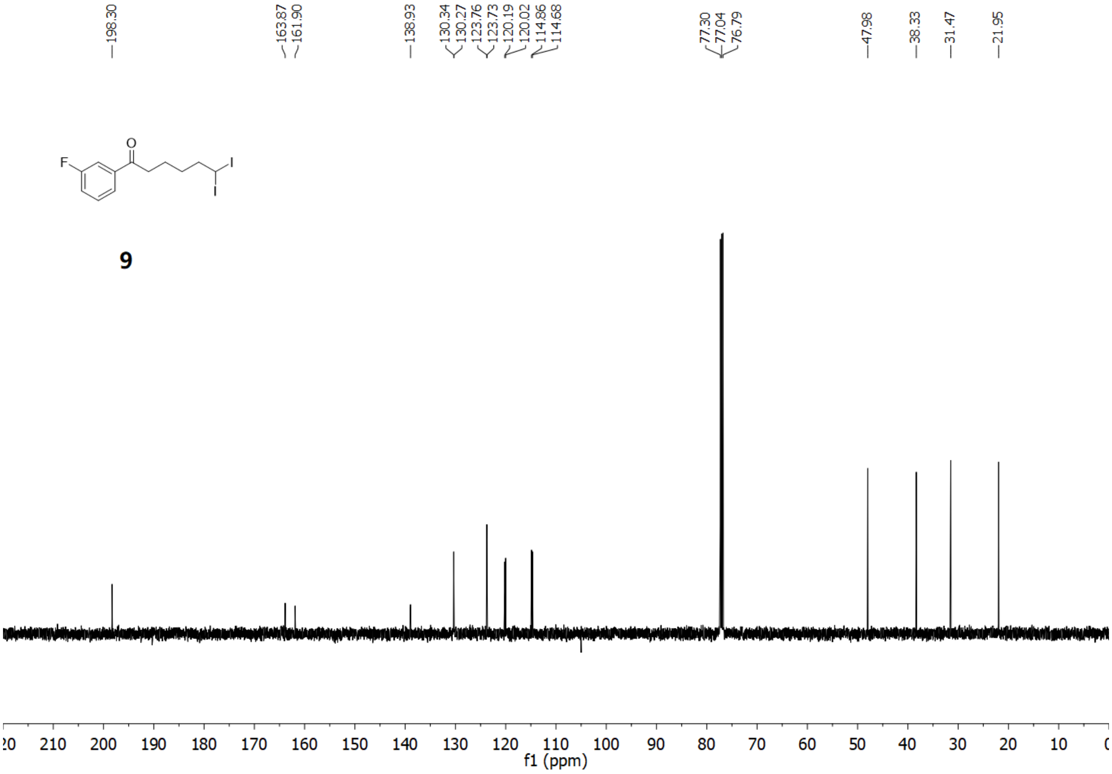

Supplementary Figure 18. ^{1}H and ^{13}C NMR spectra for compound **9**

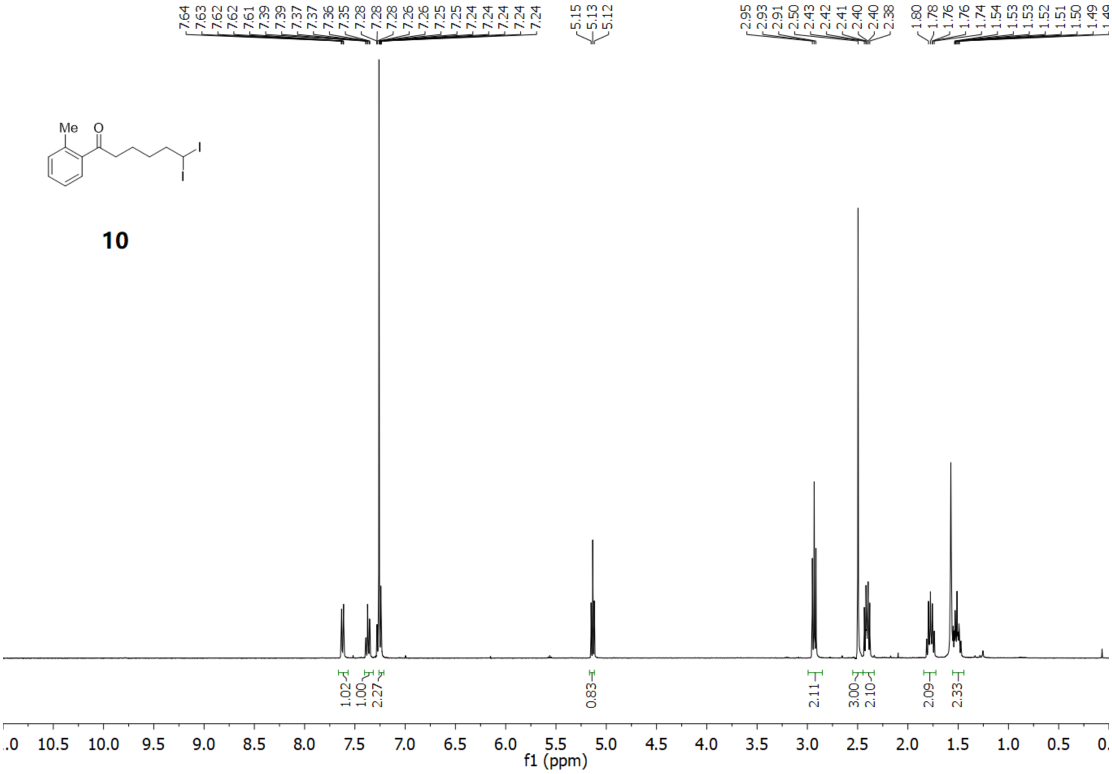

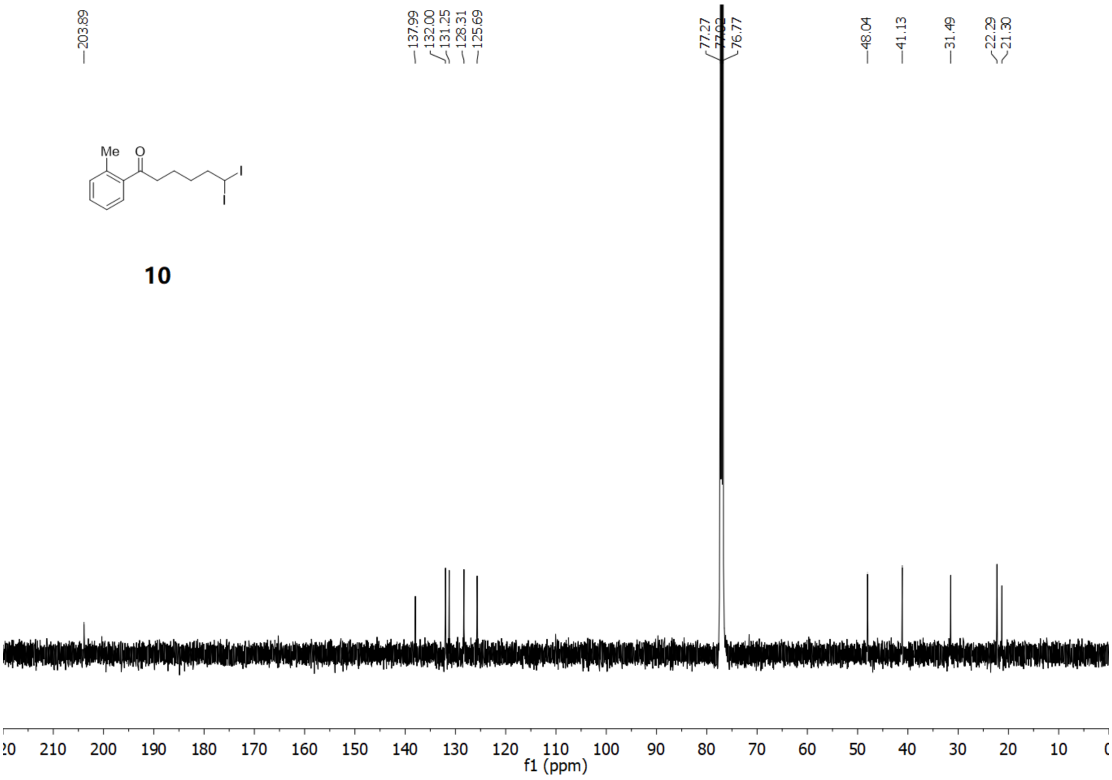

Supplementary Figure 19. ^{1}H and ^{13}C NMR spectra for compound **10**

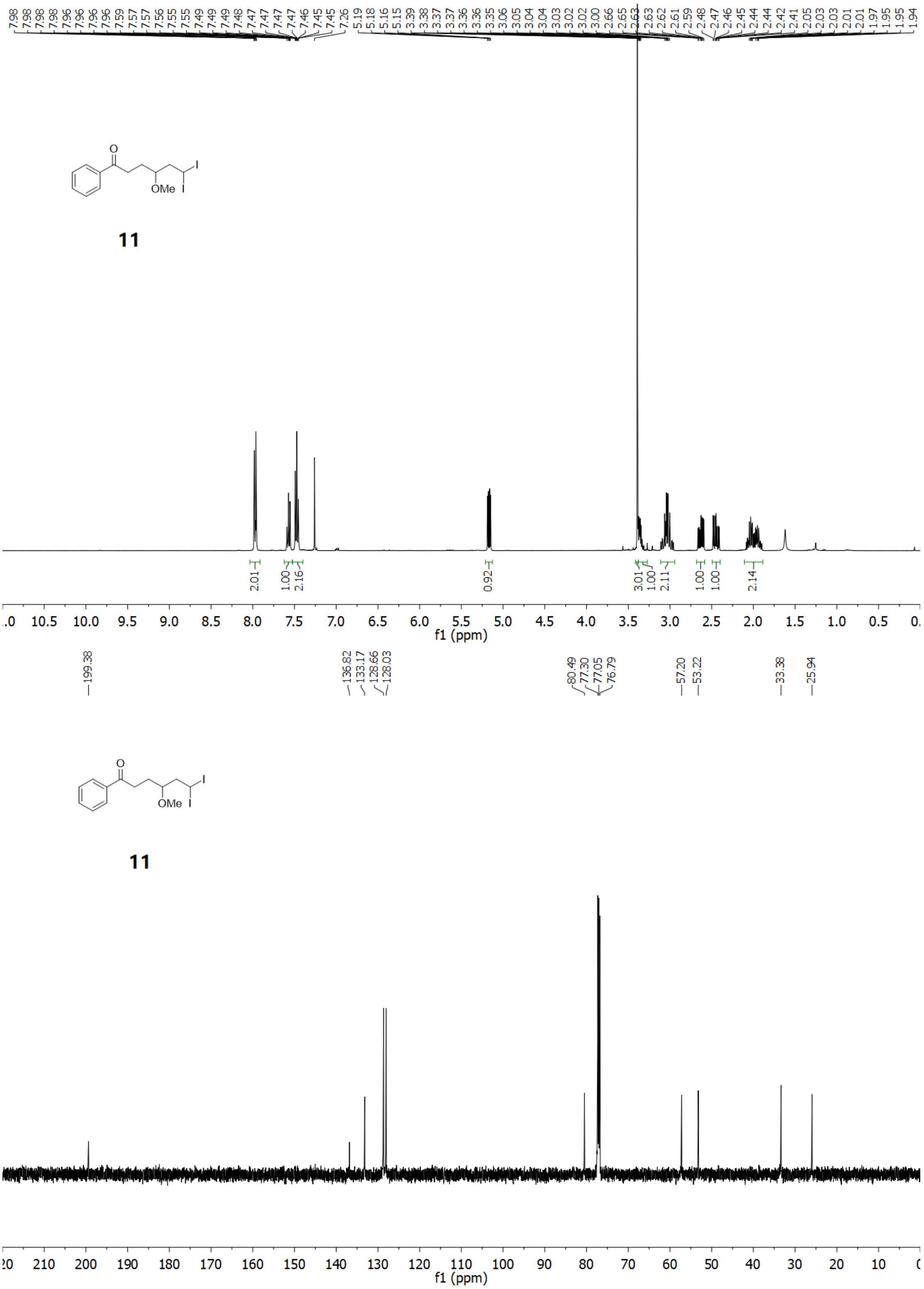

Supplementary Figure 20. ^{1}H and ^{13}C NMR spectra for compound **11**

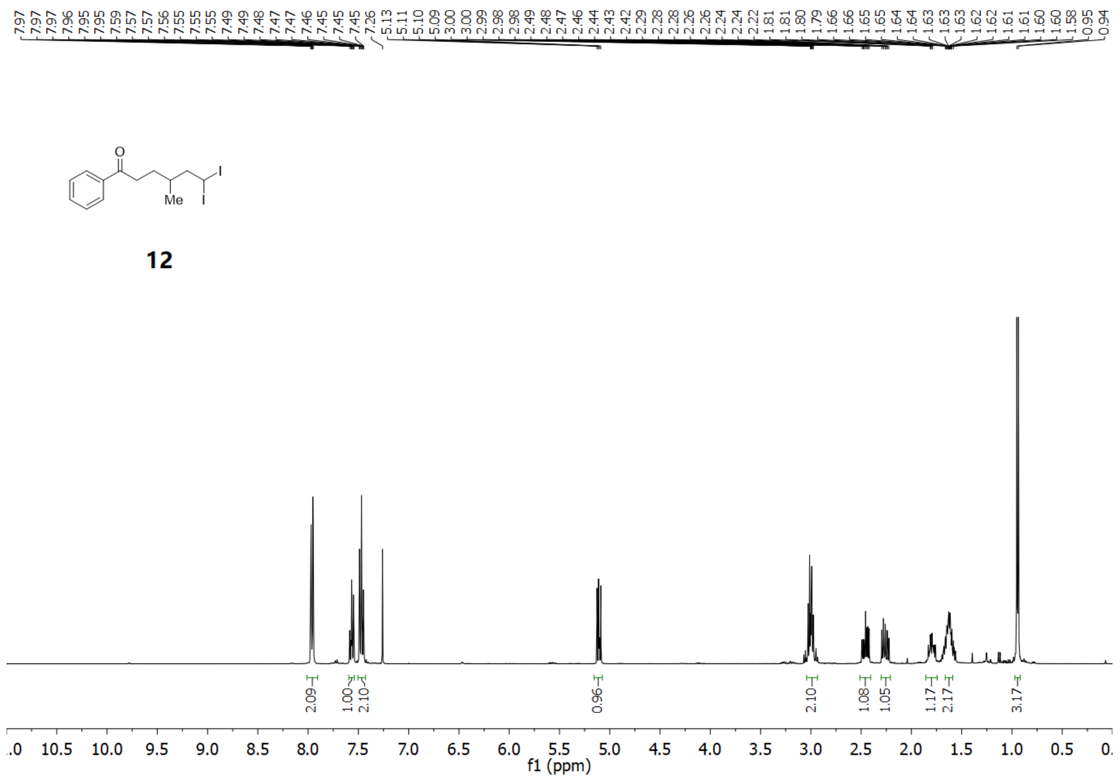

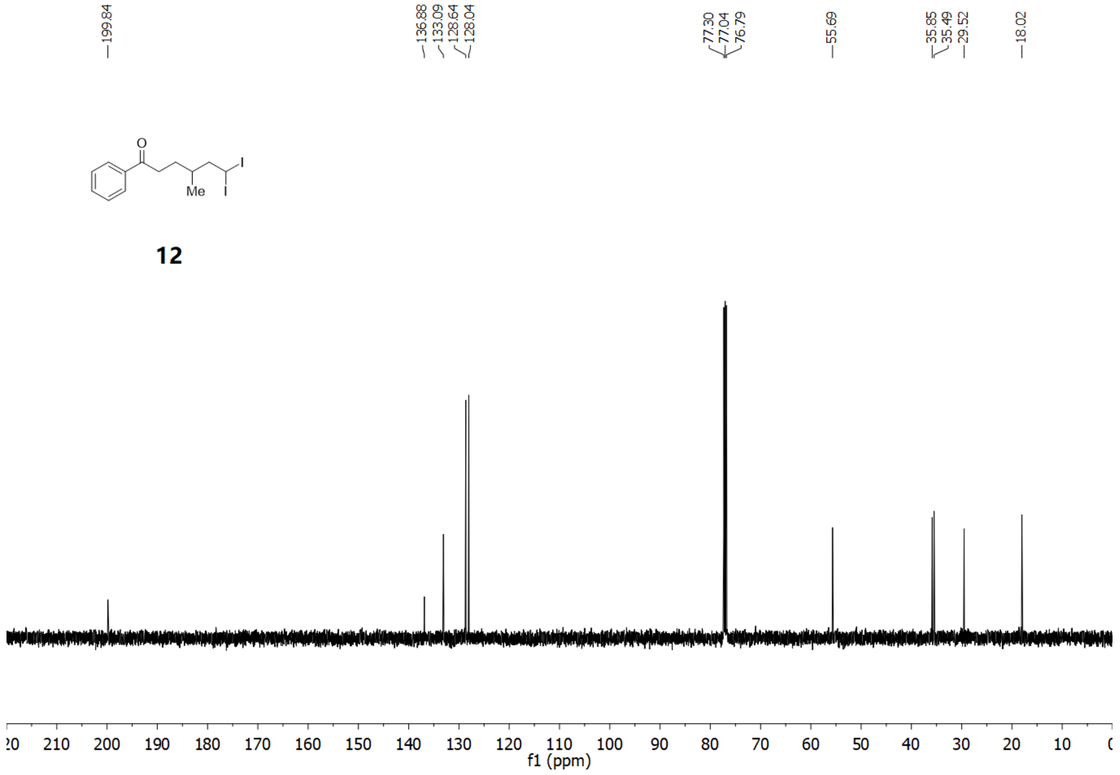

Supplementary Figure 21. ^{1}H and ^{13}C NMR spectra for compound **12**

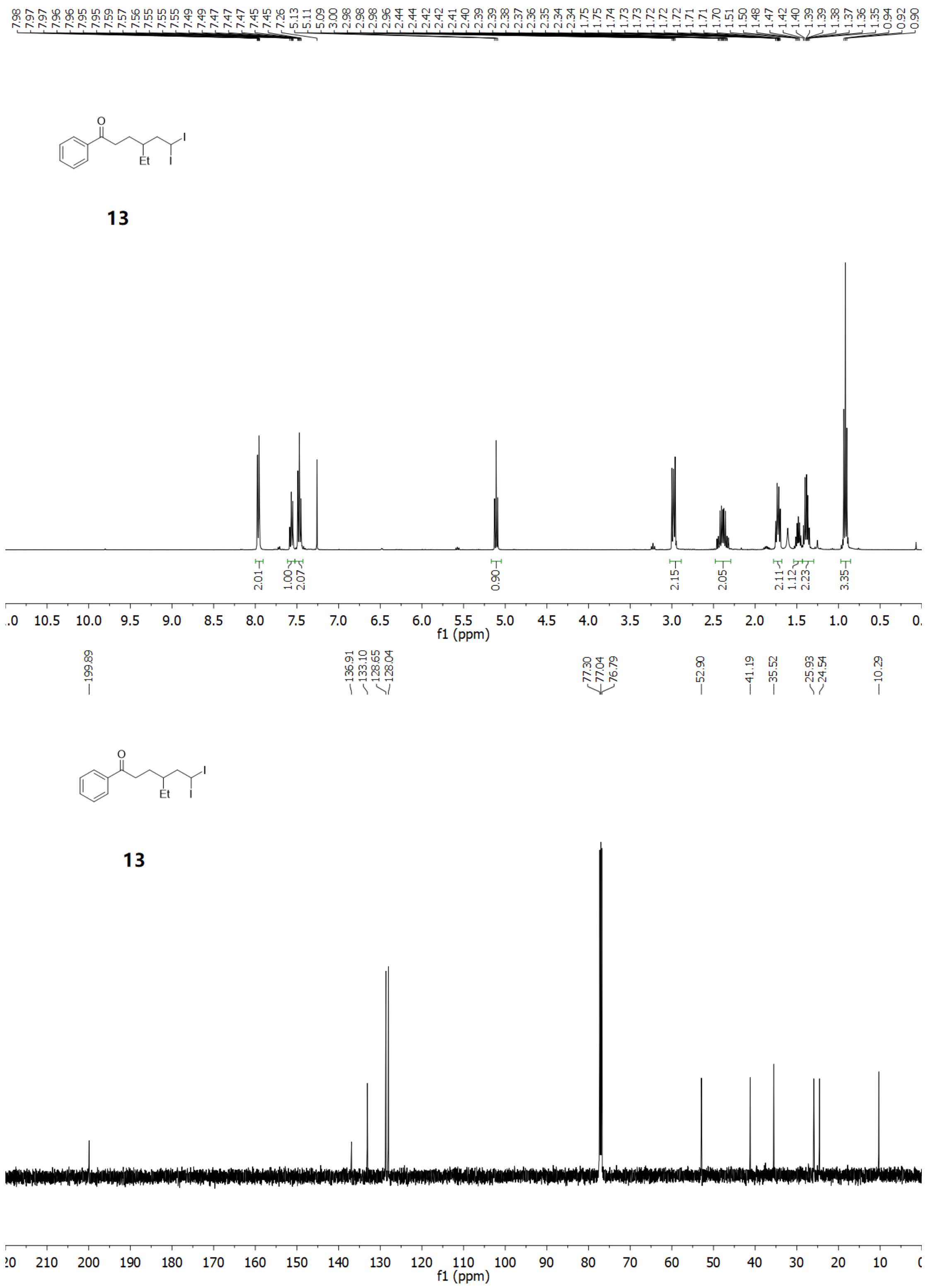

Supplementary Figure 22. ^{1}H and ^{13}C NMR spectra for compound **13**

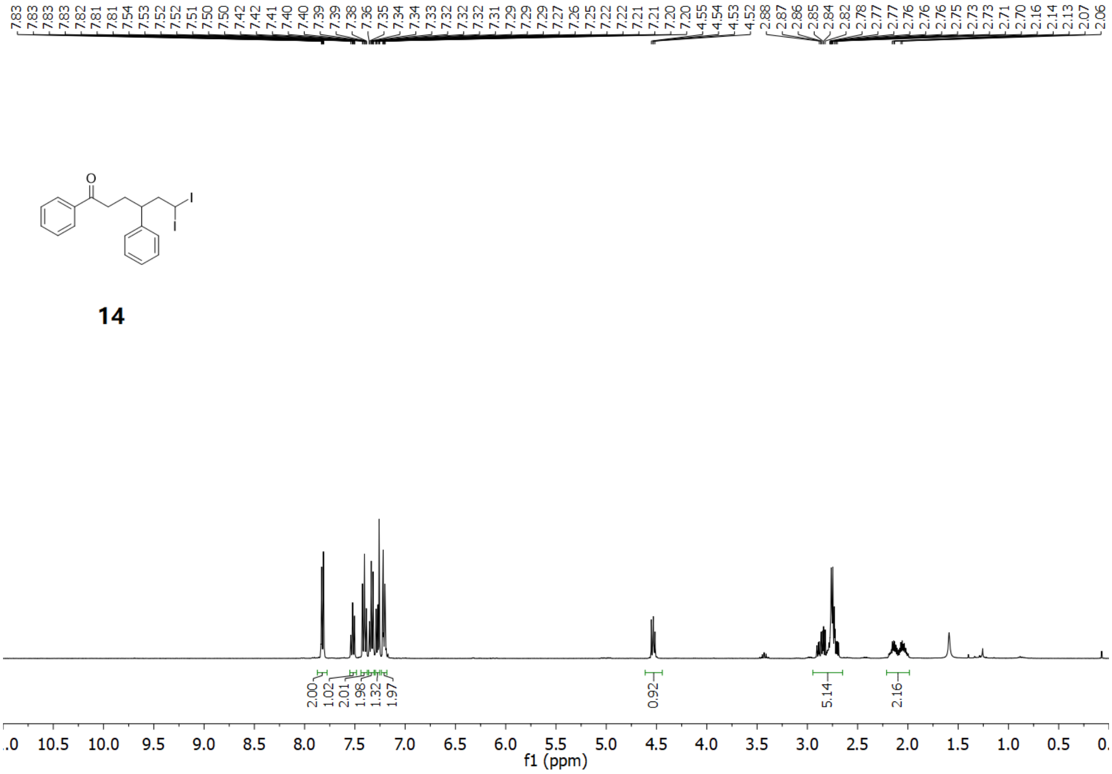

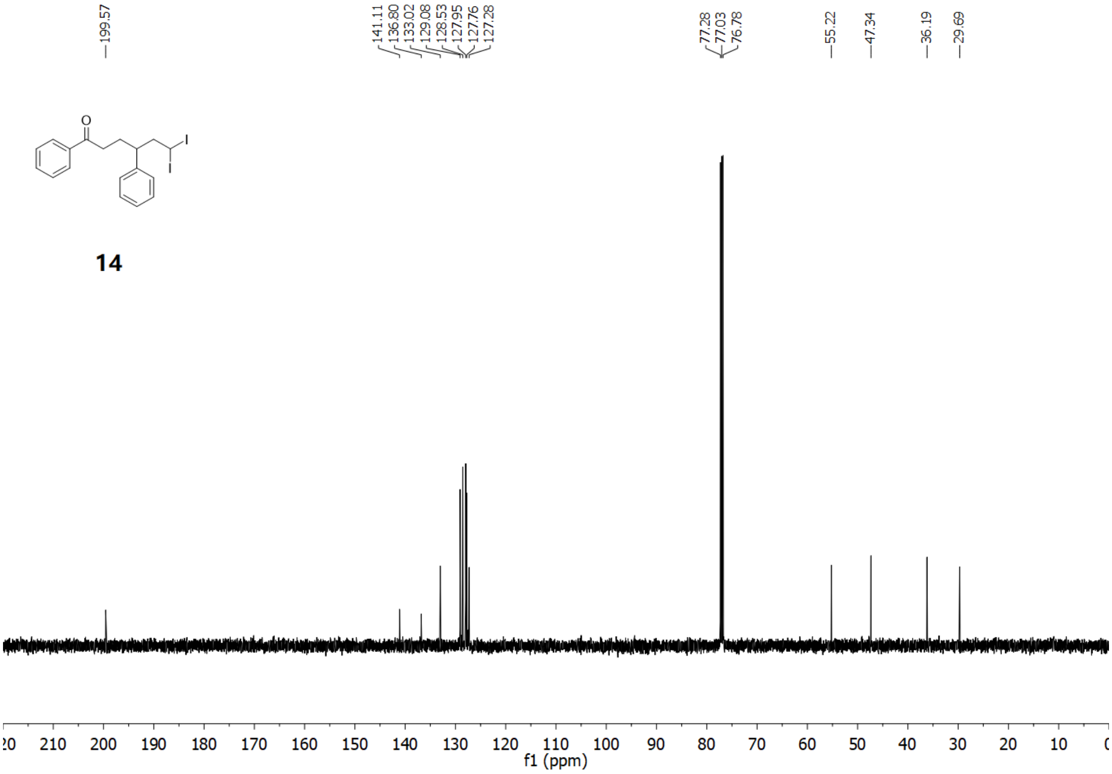

Supplementary Figure 23. ^{1}H and ^{13}C NMR spectra for compound **14**

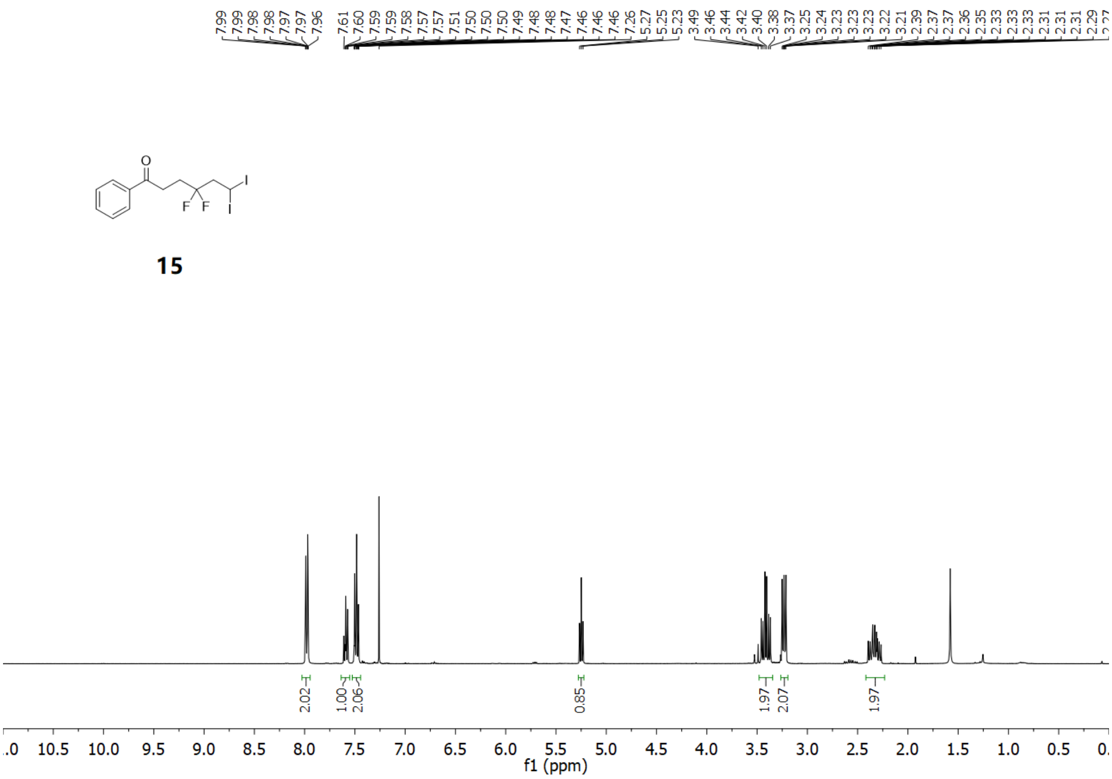

15

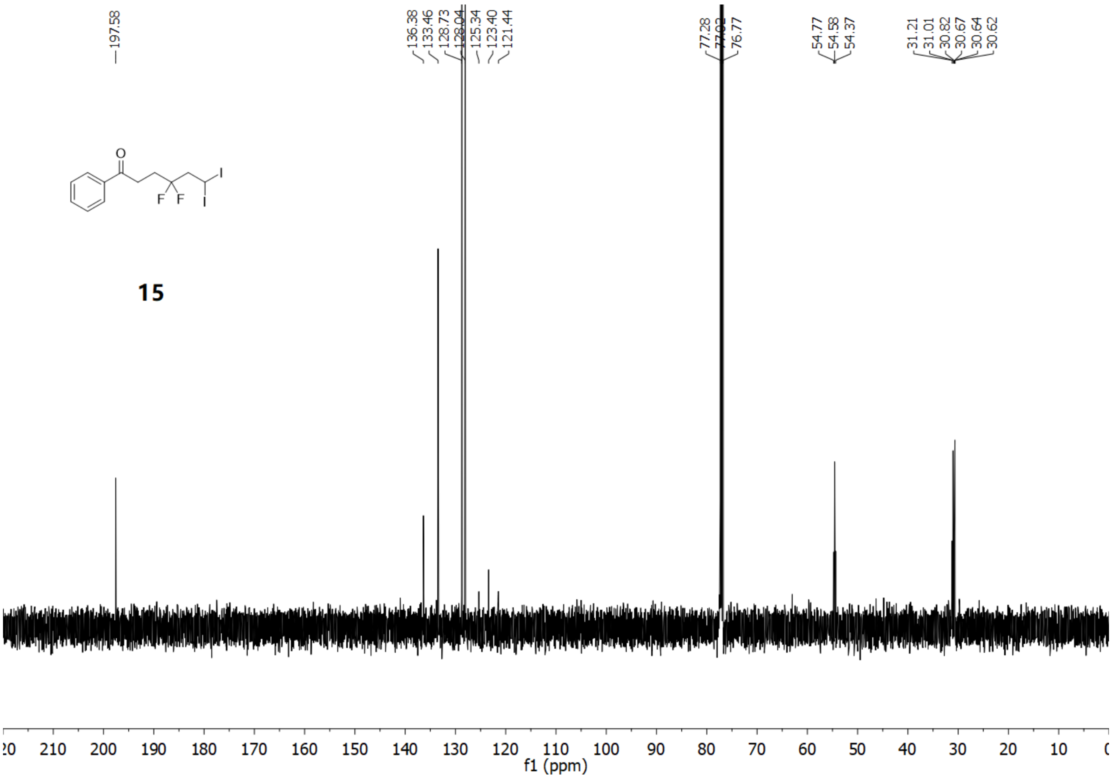

15

Supplementary Figure 24. ^{1}H and ^{13}C NMR spectra for compound **15**

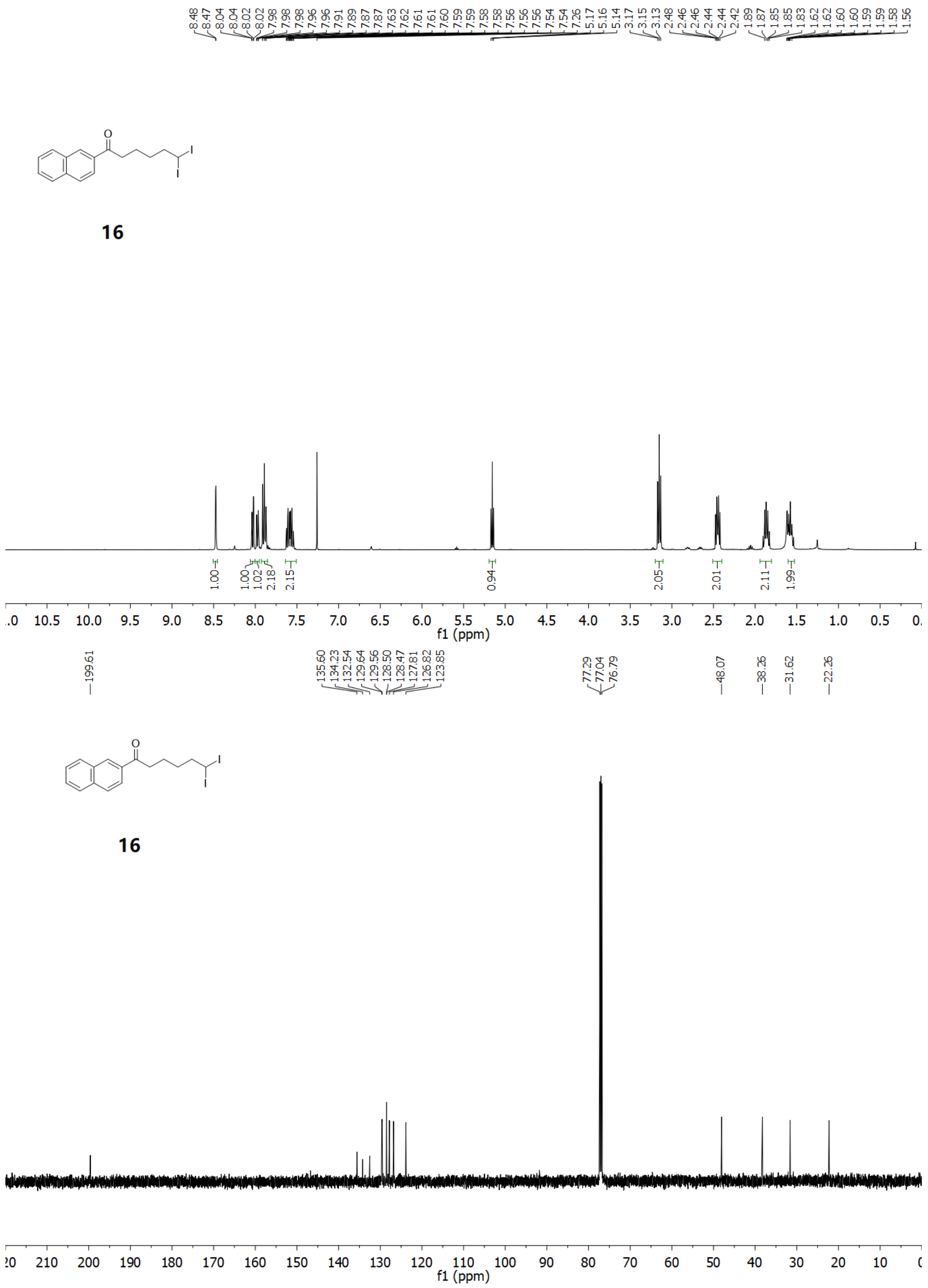

Supplementary Figure 25. ^{1}H and ^{13}C NMR spectra for compound **16**

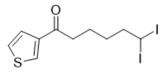

17

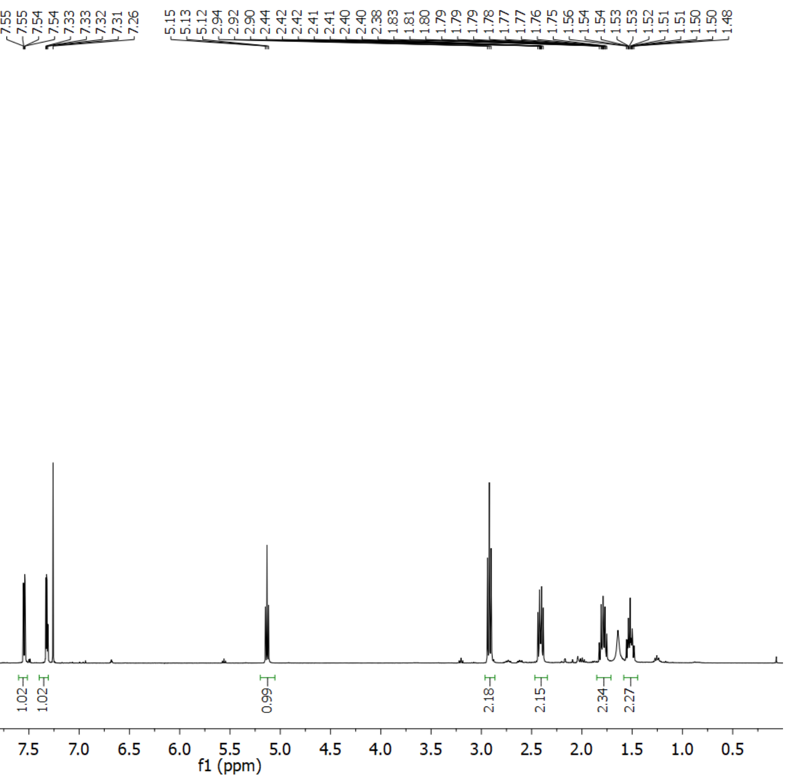

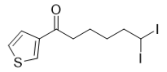

17

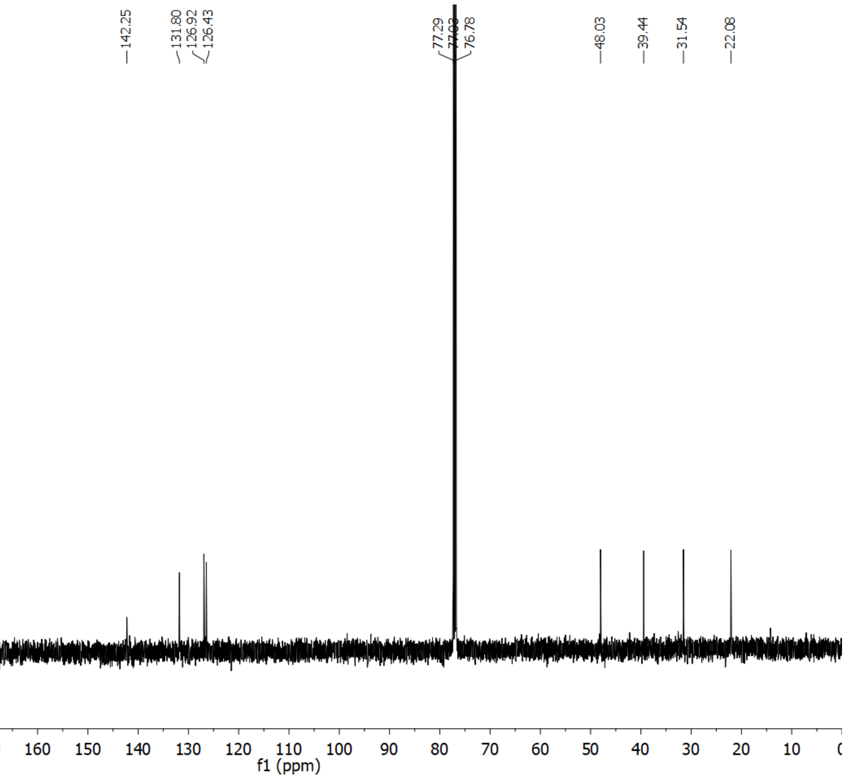

Supplementary Figure 26. ^{1}H and ^{13}C NMR spectra for compound **17**

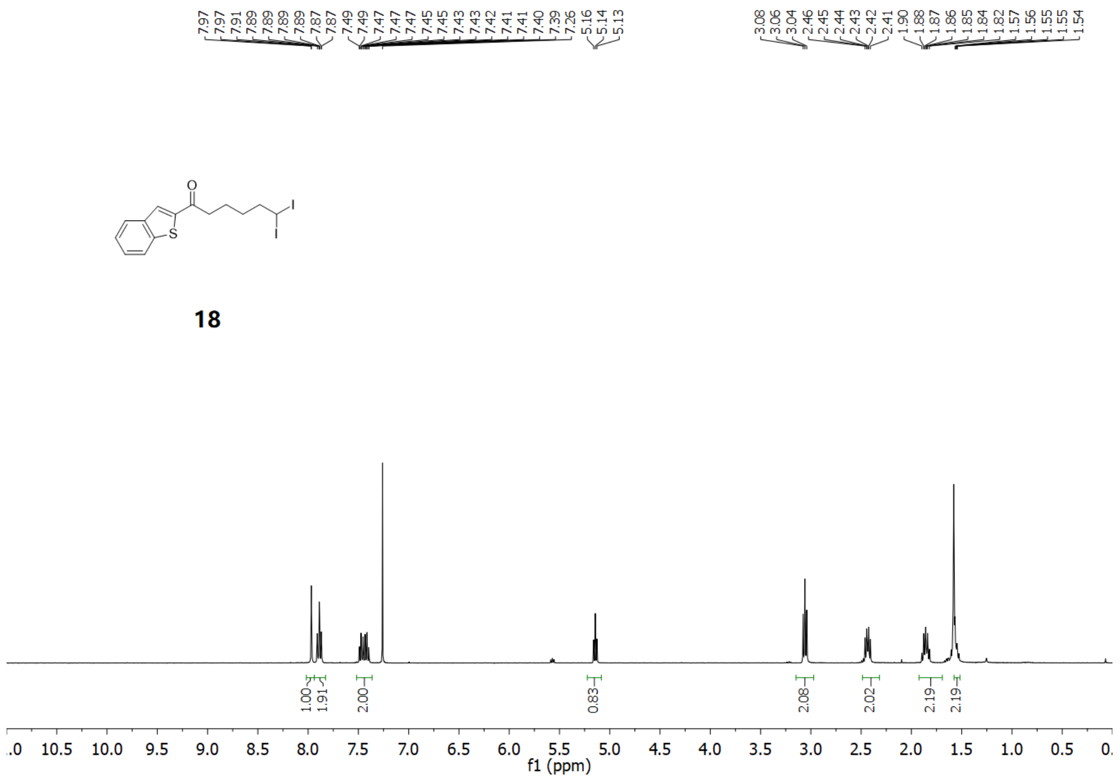

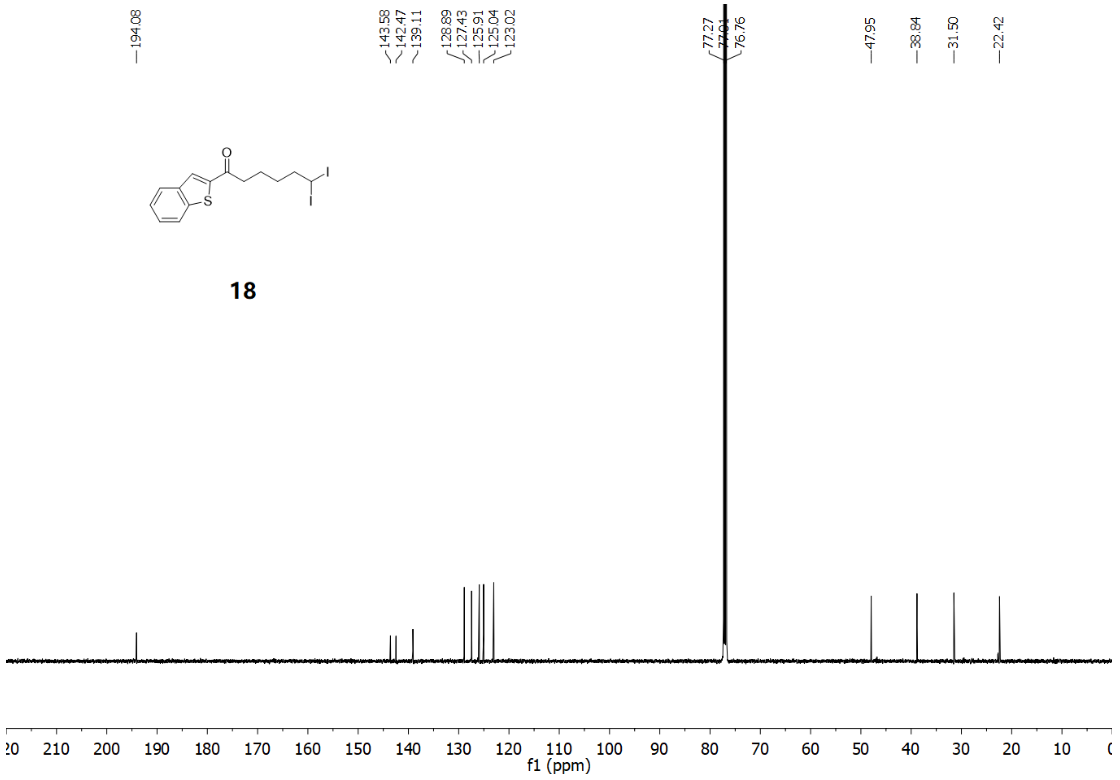

Supplementary Figure 27. ^{1}H and ^{13}C NMR spectra for compound **18**

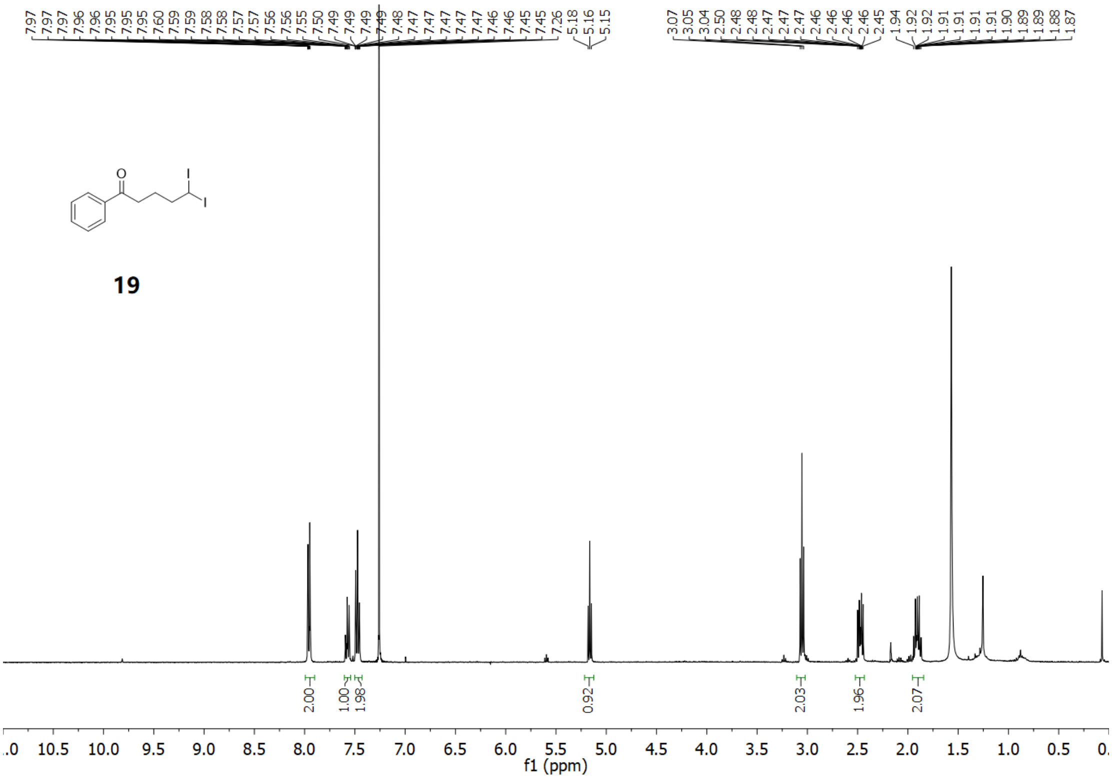

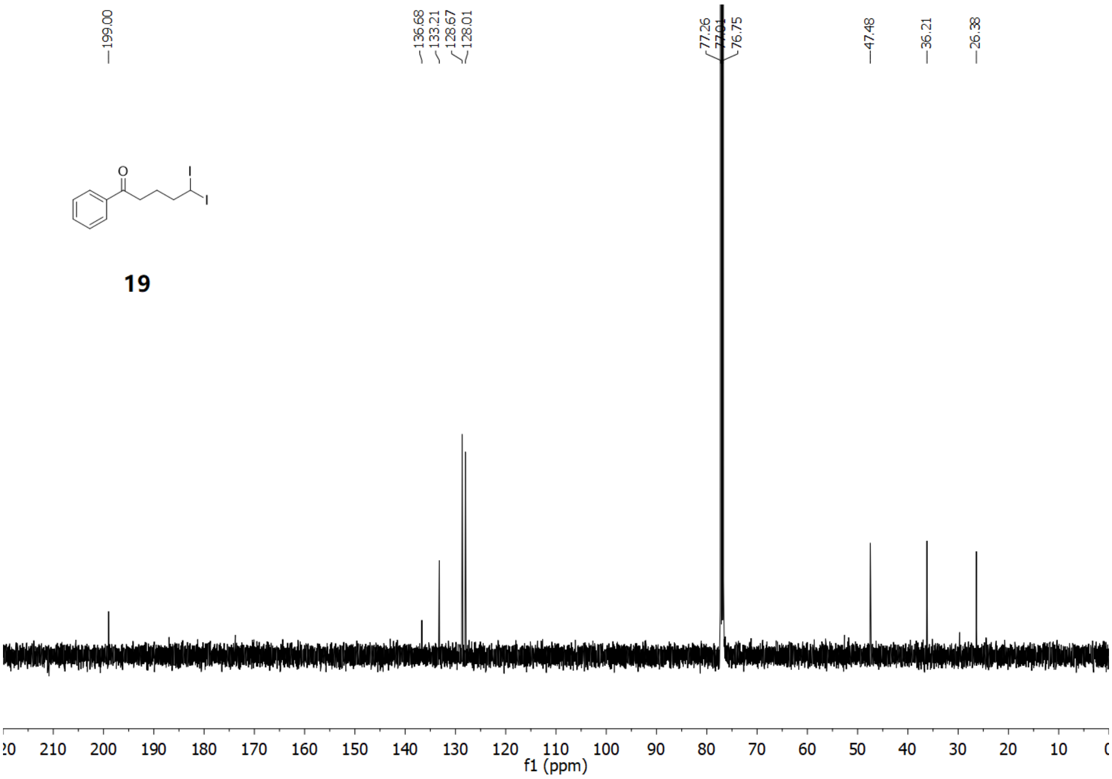

Supplementary Figure 28. ^{1}H and ^{13}C NMR spectra for compound **19**

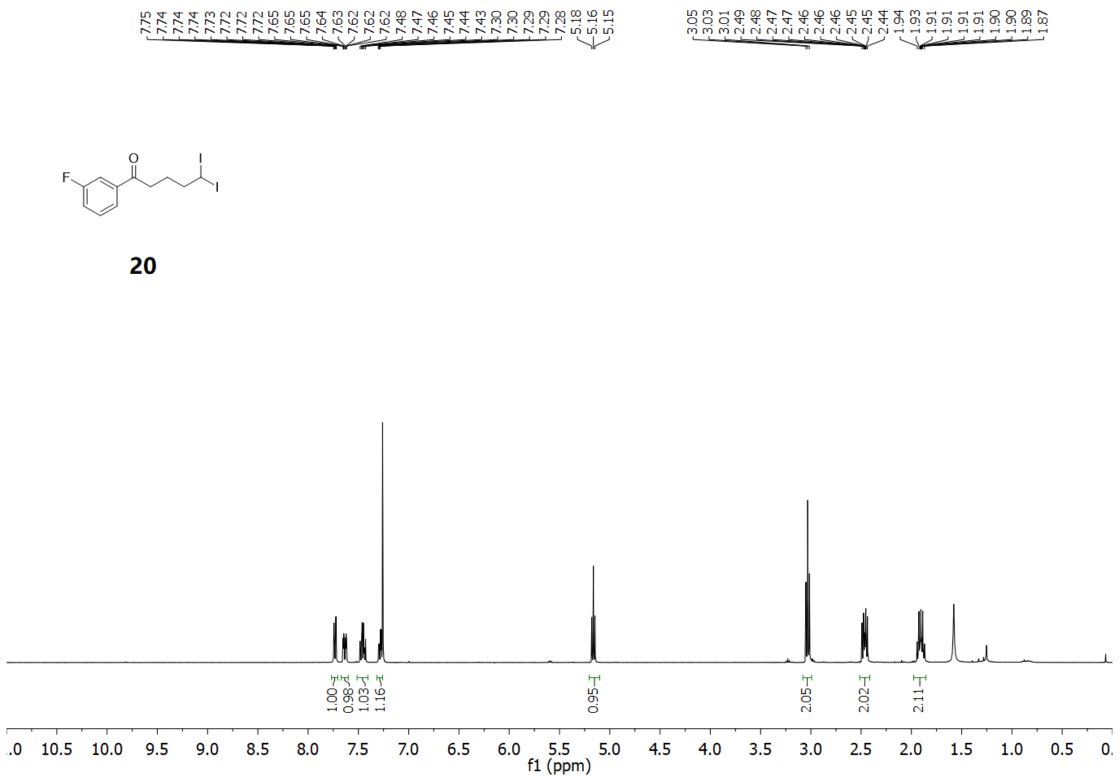

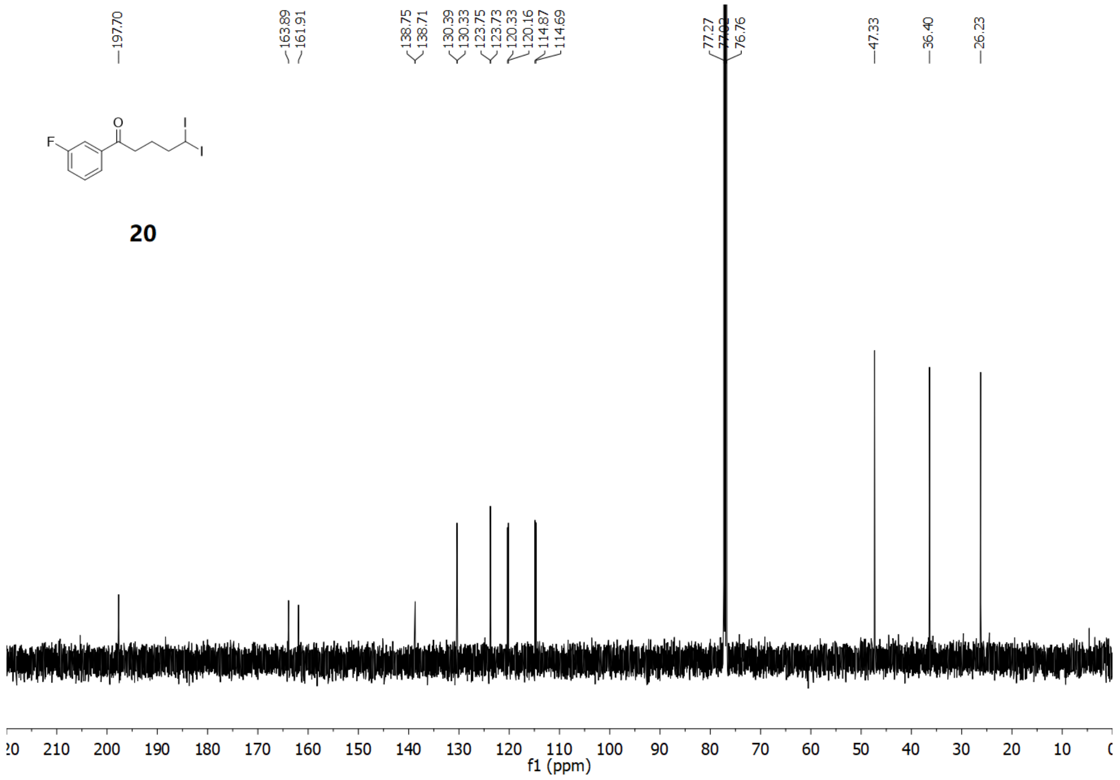

Supplementary Figure 29. ^{1}H and ^{13}C NMR spectra for compound **20**

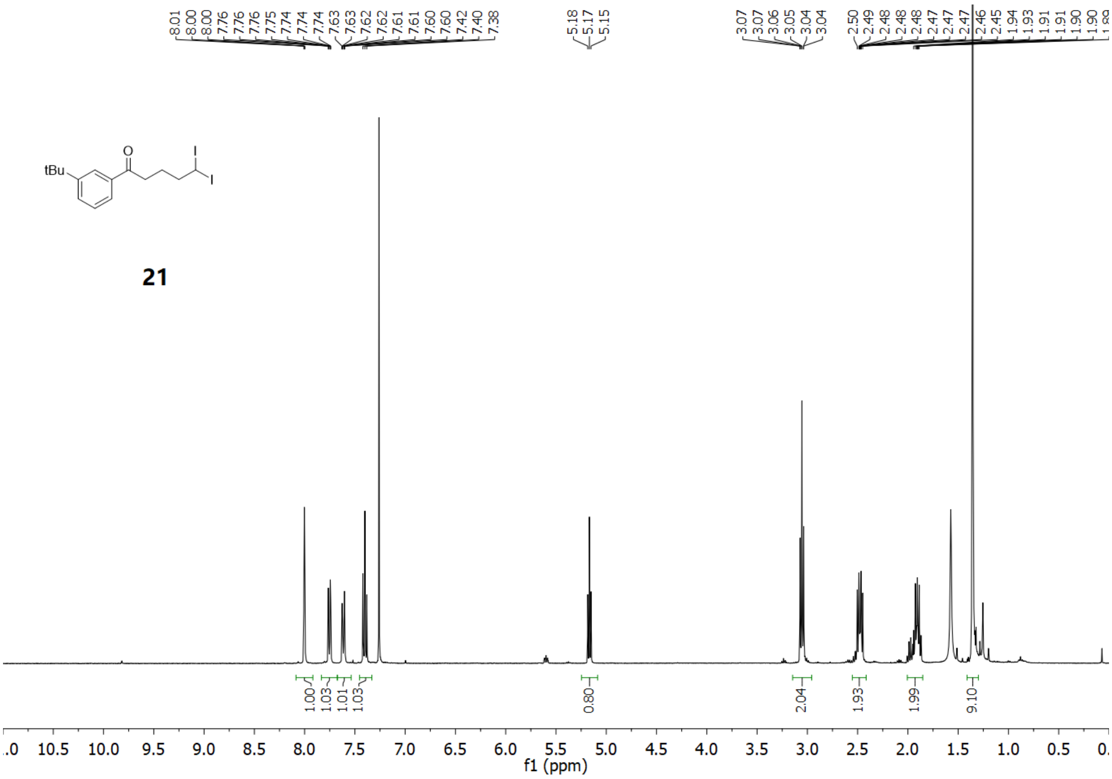

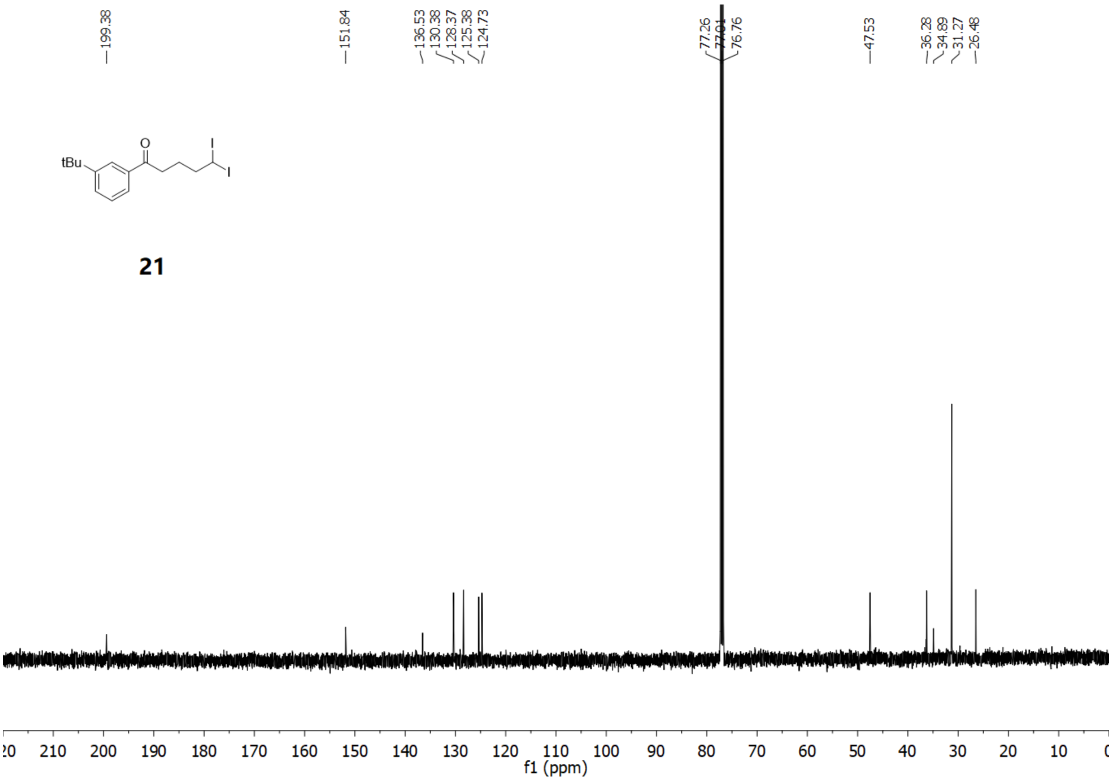

Supplementary Figure 30. ^{1}H and ^{13}C NMR spectra for compound **21**

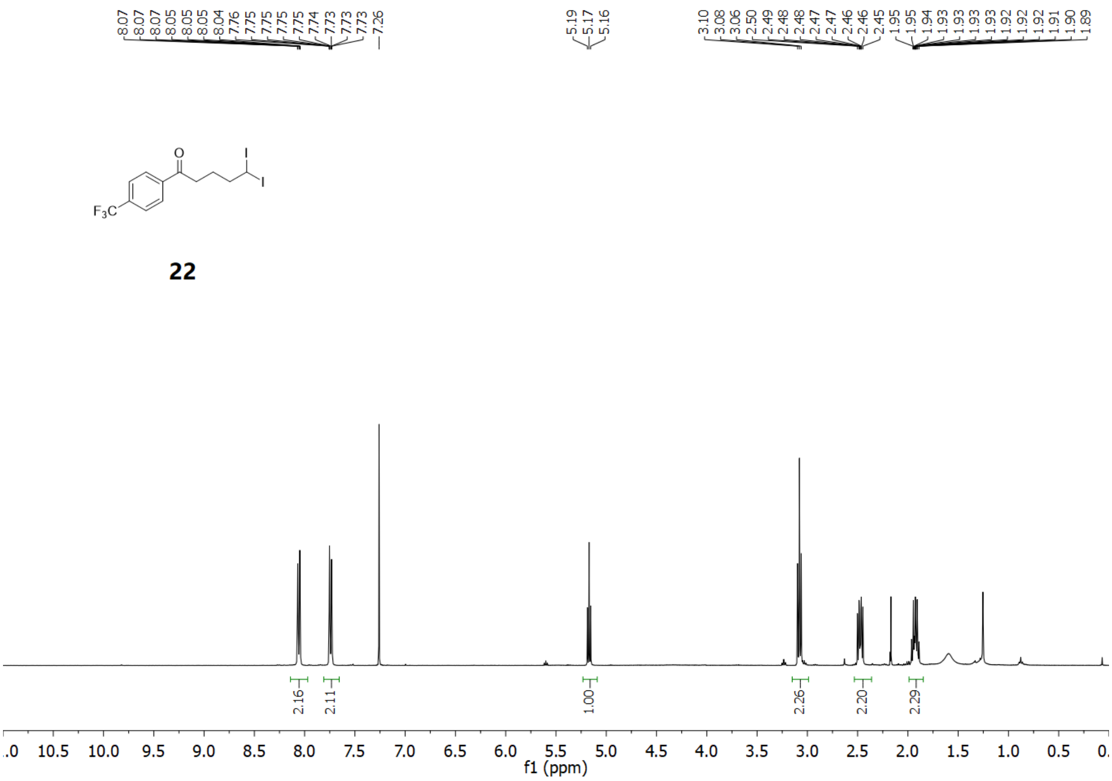

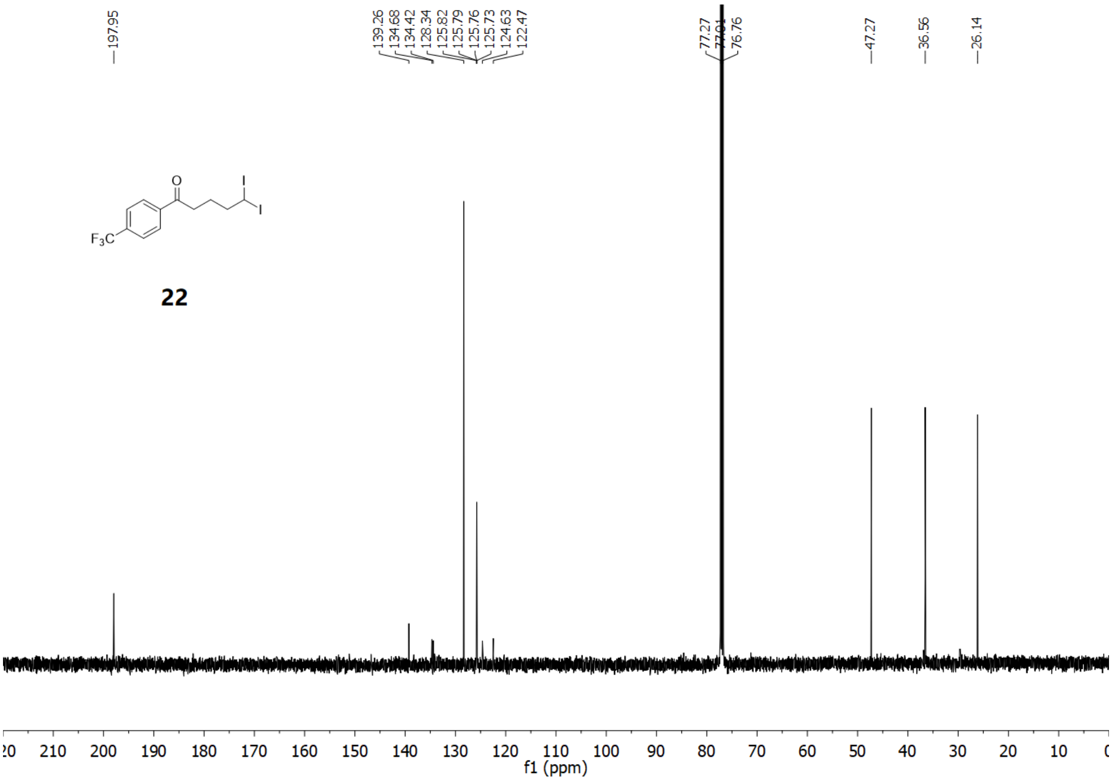

Supplementary Figure 31. ^{1}H and ^{13}C NMR spectra for compound **22**

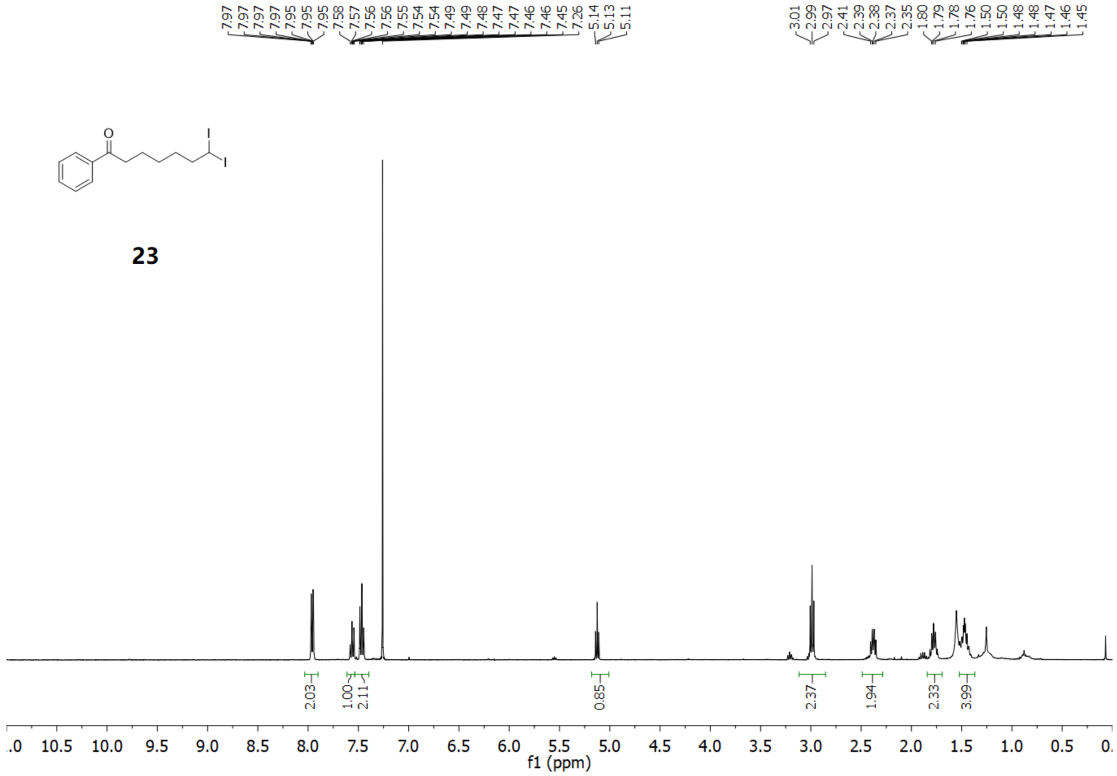

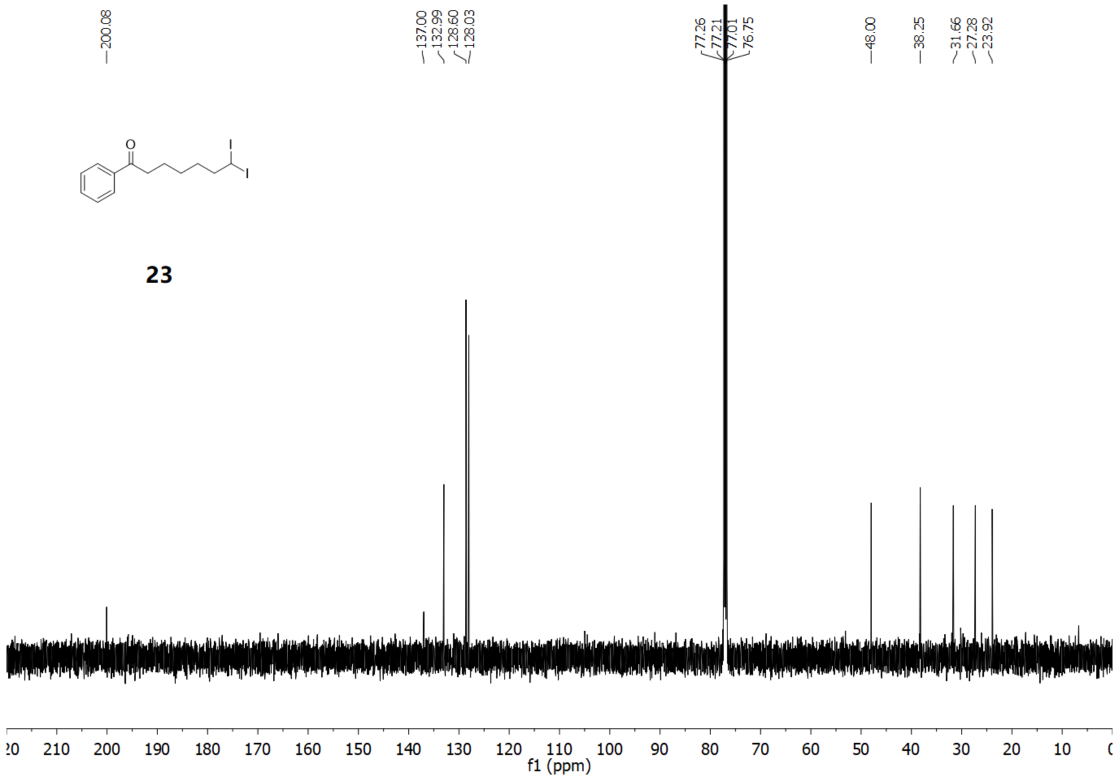

Supplementary Figure 32. ^{1}H and ^{13}C NMR spectra for compound **23**

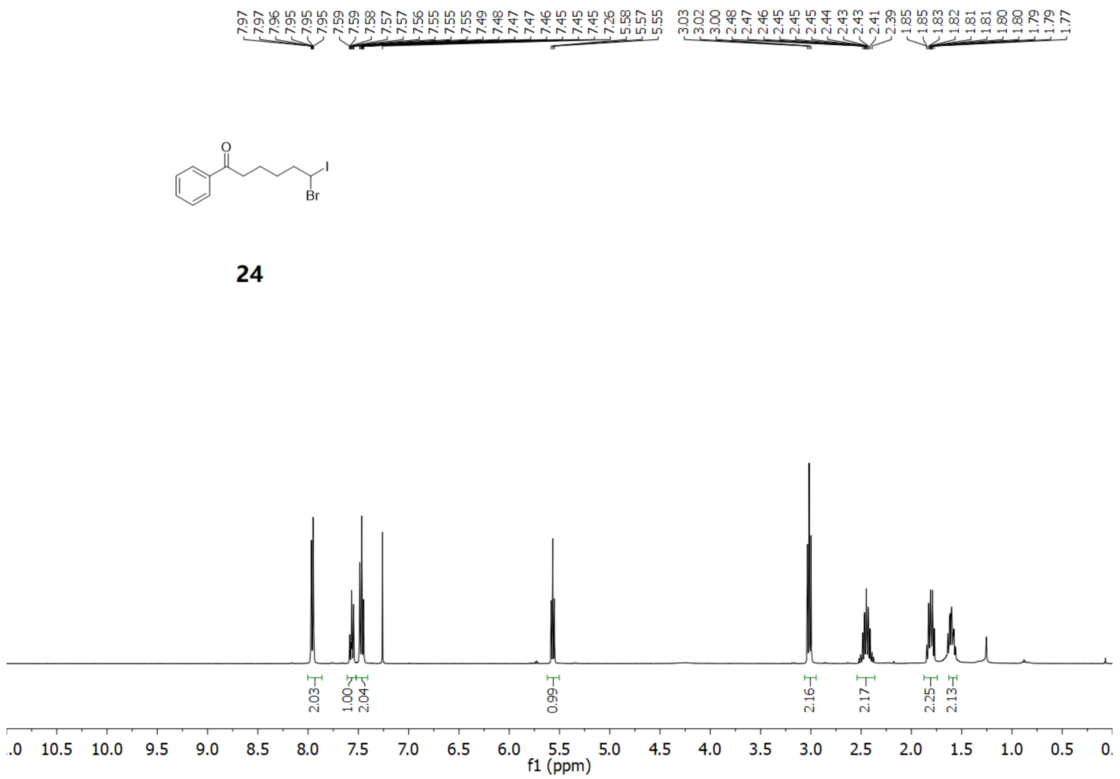

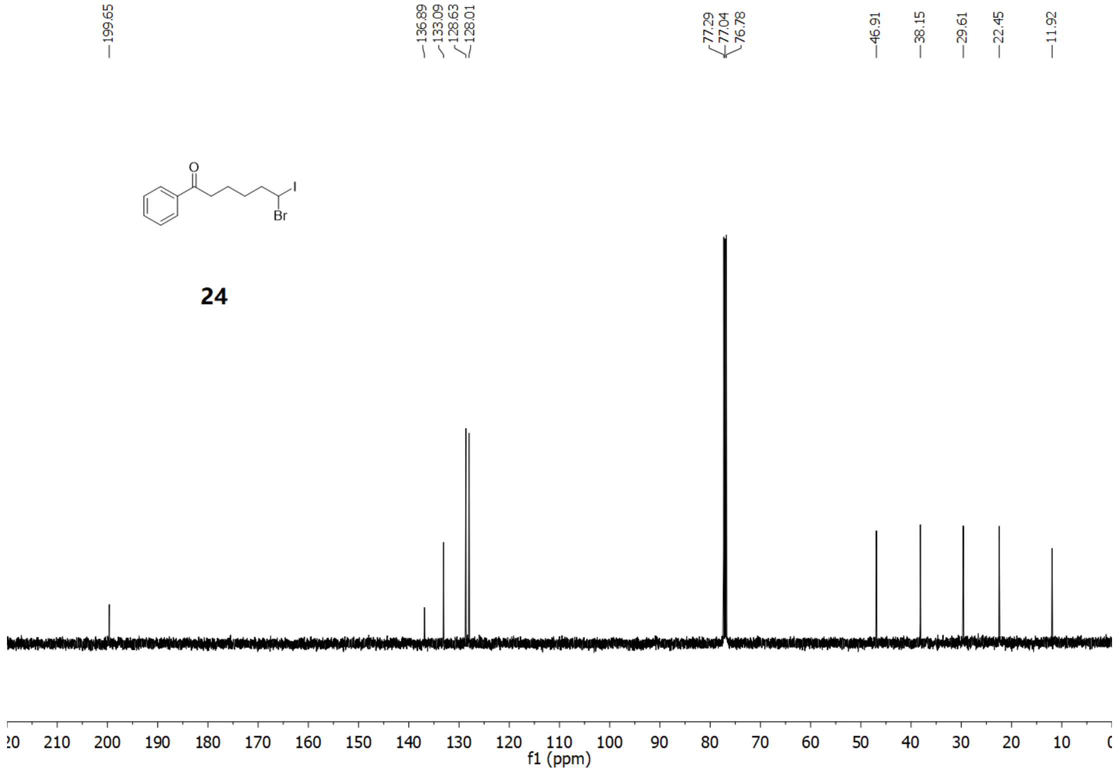

Supplementary Figure 33. ^{1}H and ^{13}C NMR spectra for compound **24**

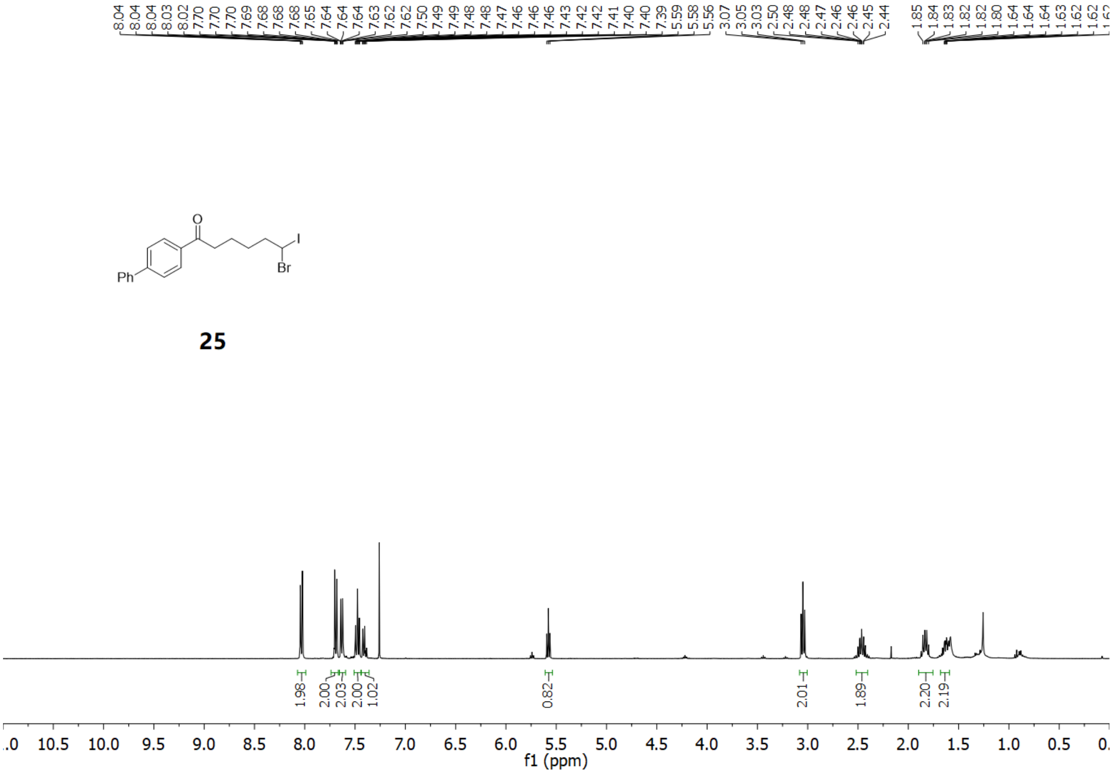

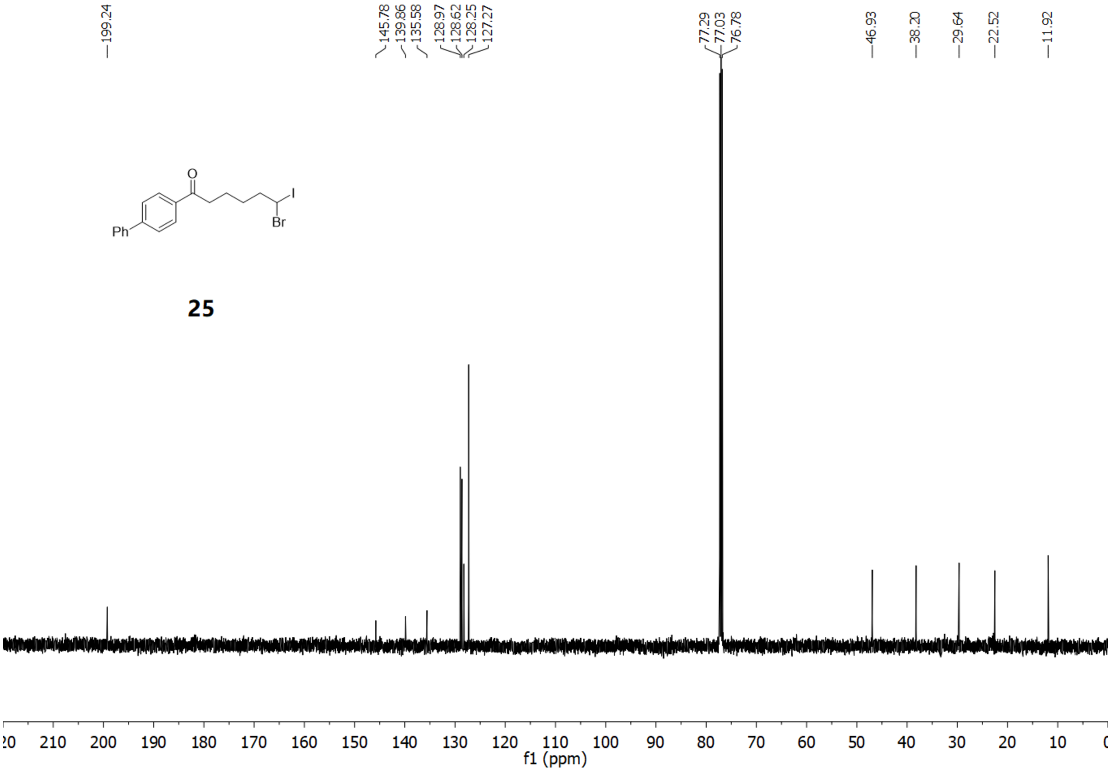

Supplementary Figure 34. ^{1}H and ^{13}C NMR spectra for compound **25**

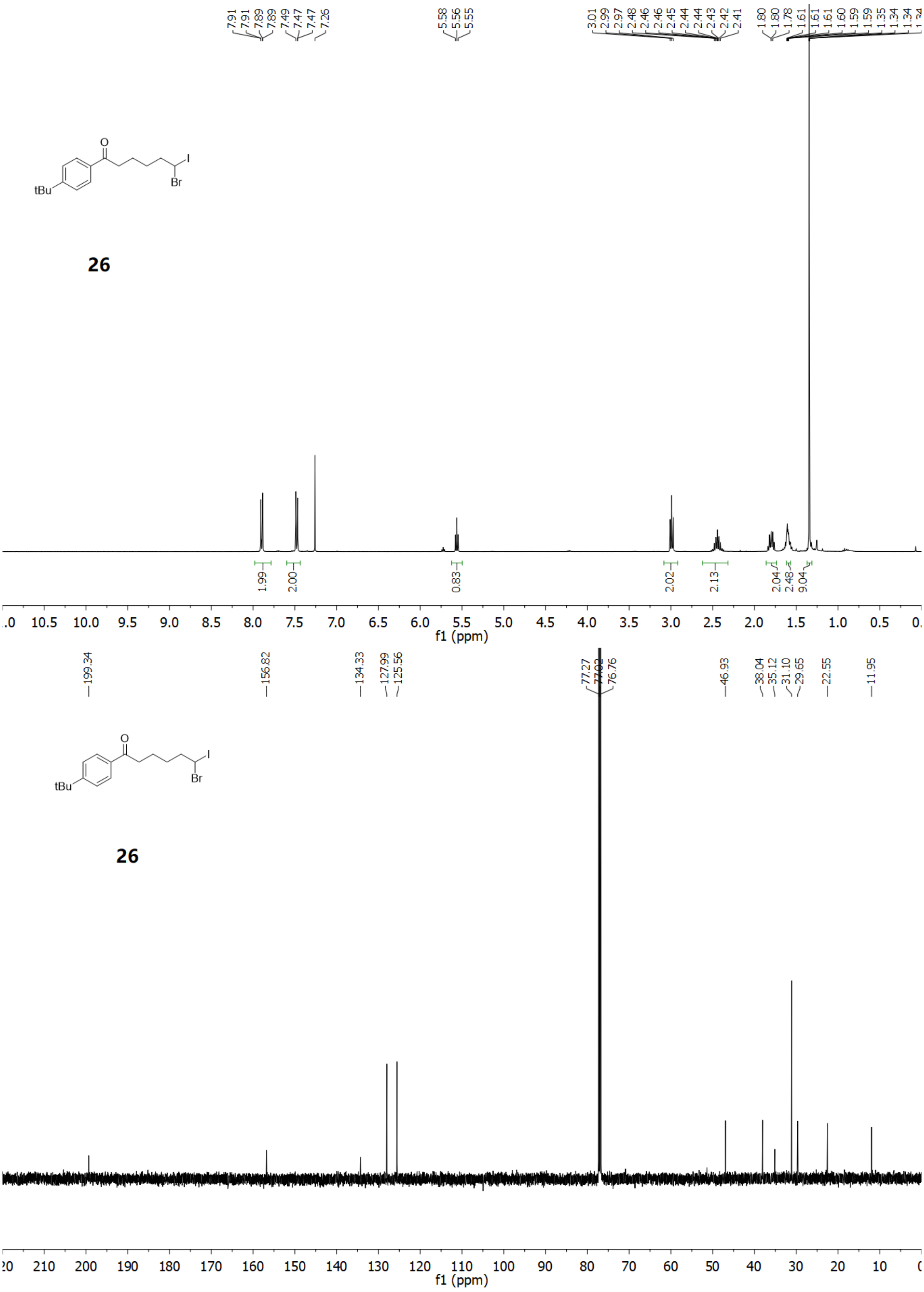

Supplementary Figure 35. ^{1}H and ^{13}C NMR spectra for compound **26**

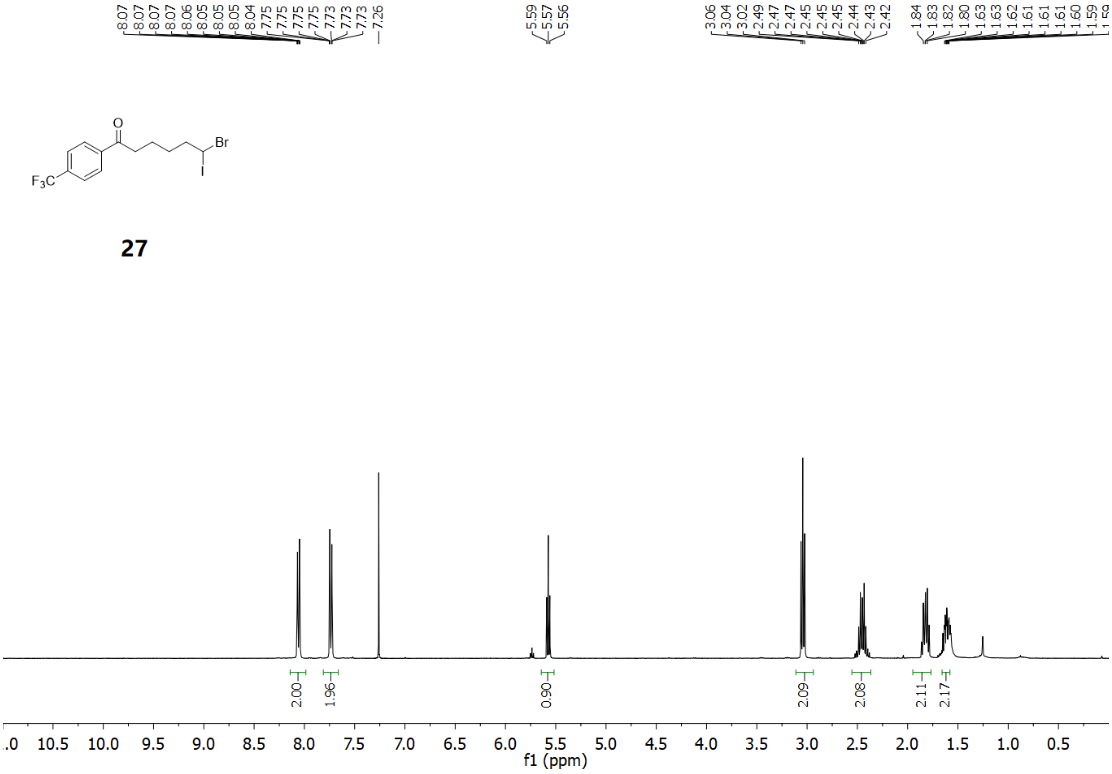

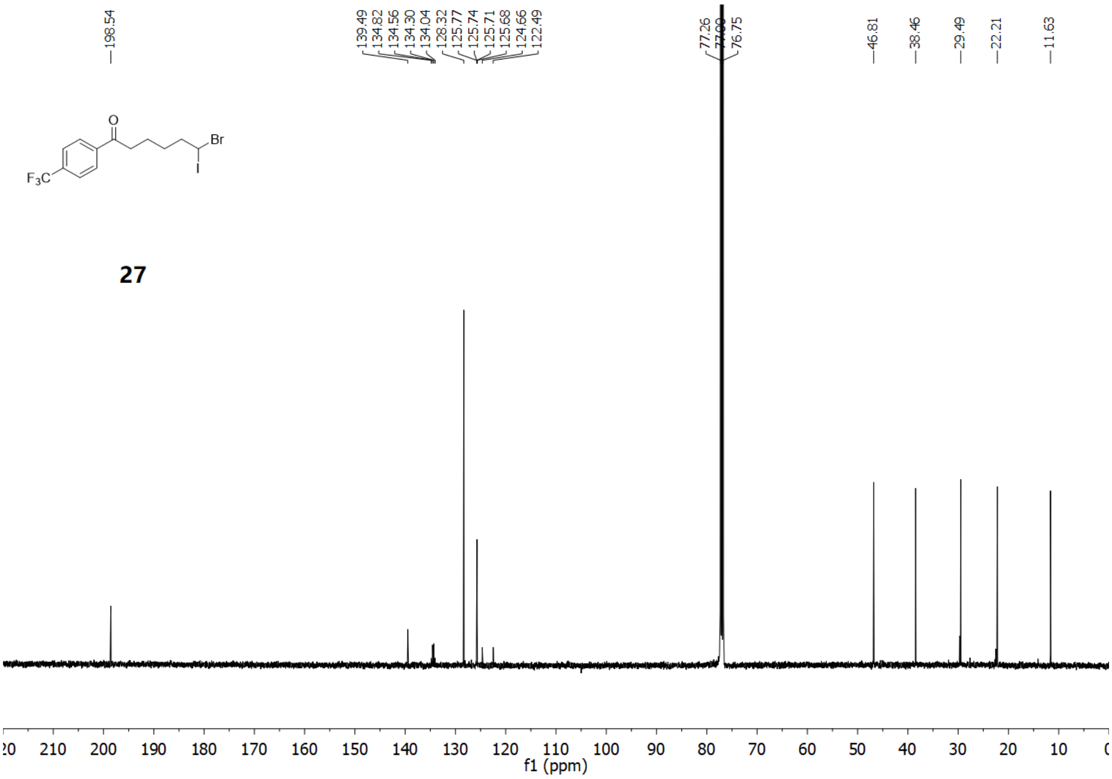

Supplementary Figure 36. ^{1}H and ^{13}C NMR spectra for compound **27**

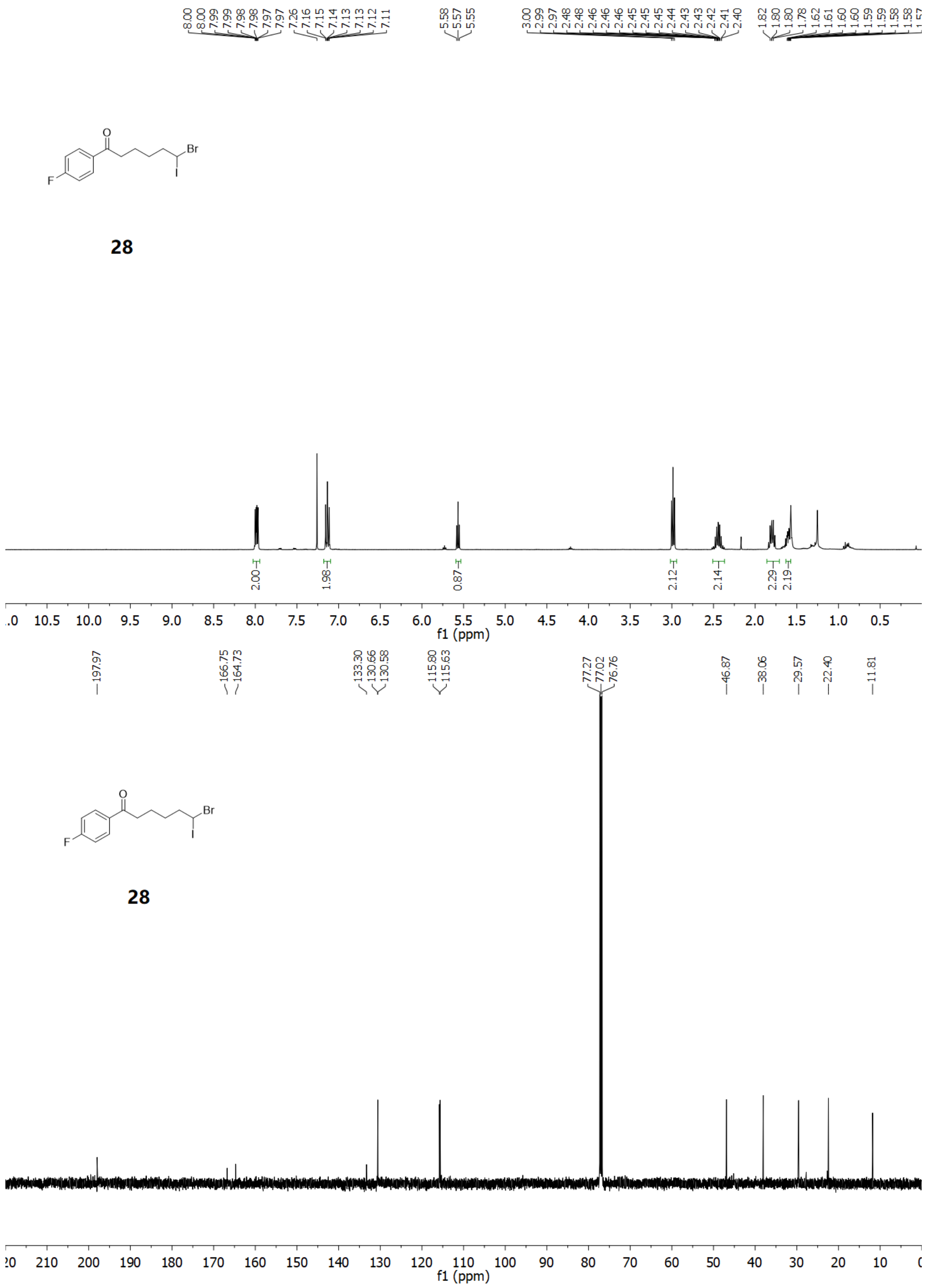

Supplementary Figure 37. ^{1}H and ^{13}C NMR spectra for compound **28**

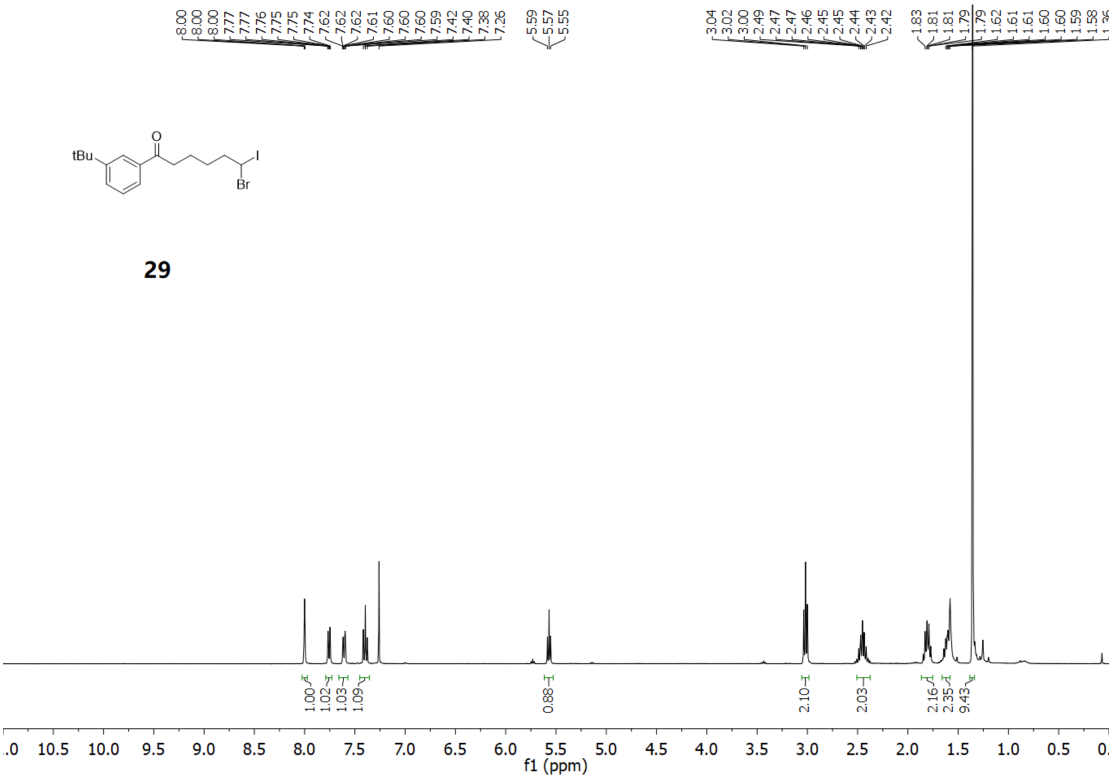

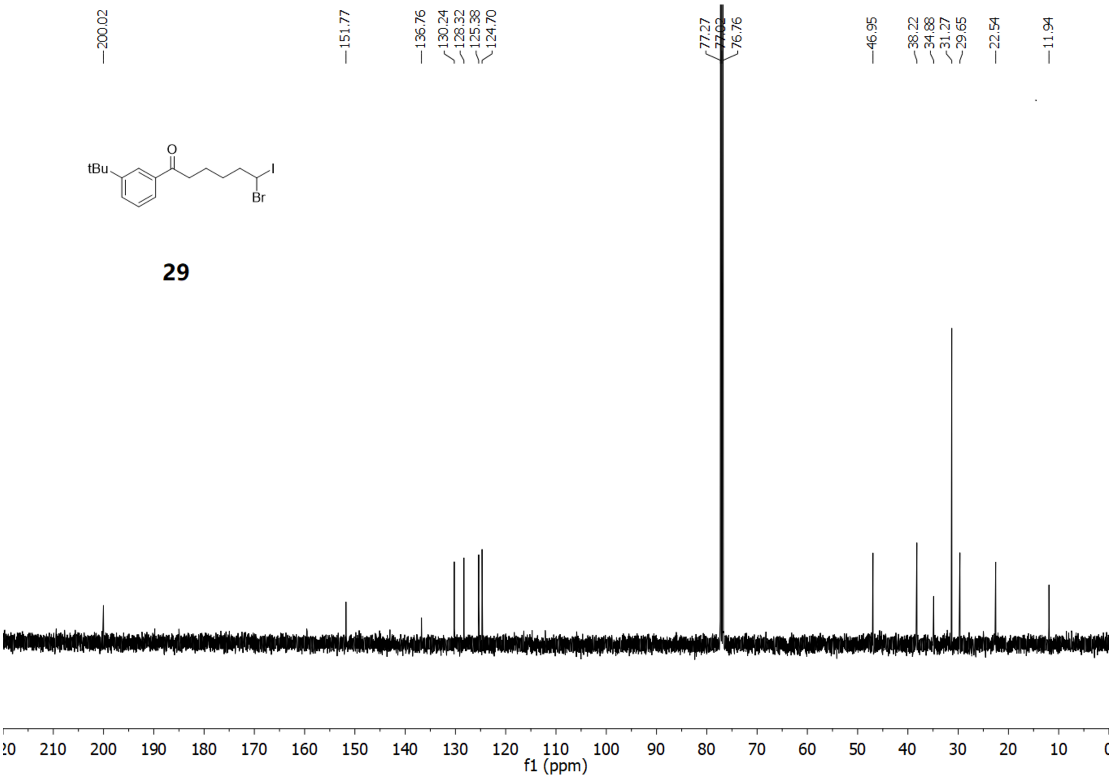

Supplementary Figure 38. ^{1}H and ^{13}C NMR spectra for compound **29**

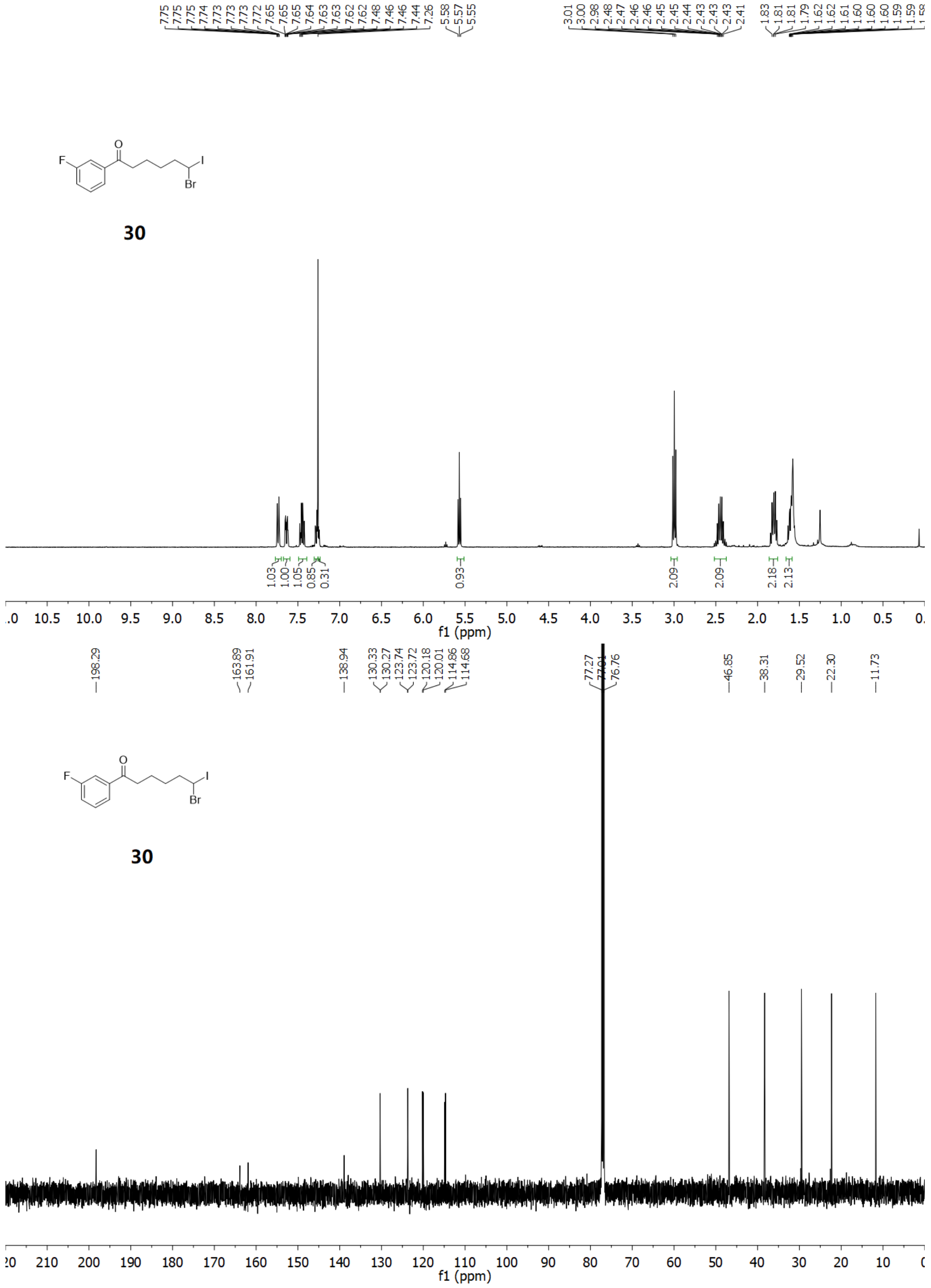

Supplementary Figure 39. ^{1}H and ^{13}C NMR spectra for compound **30**

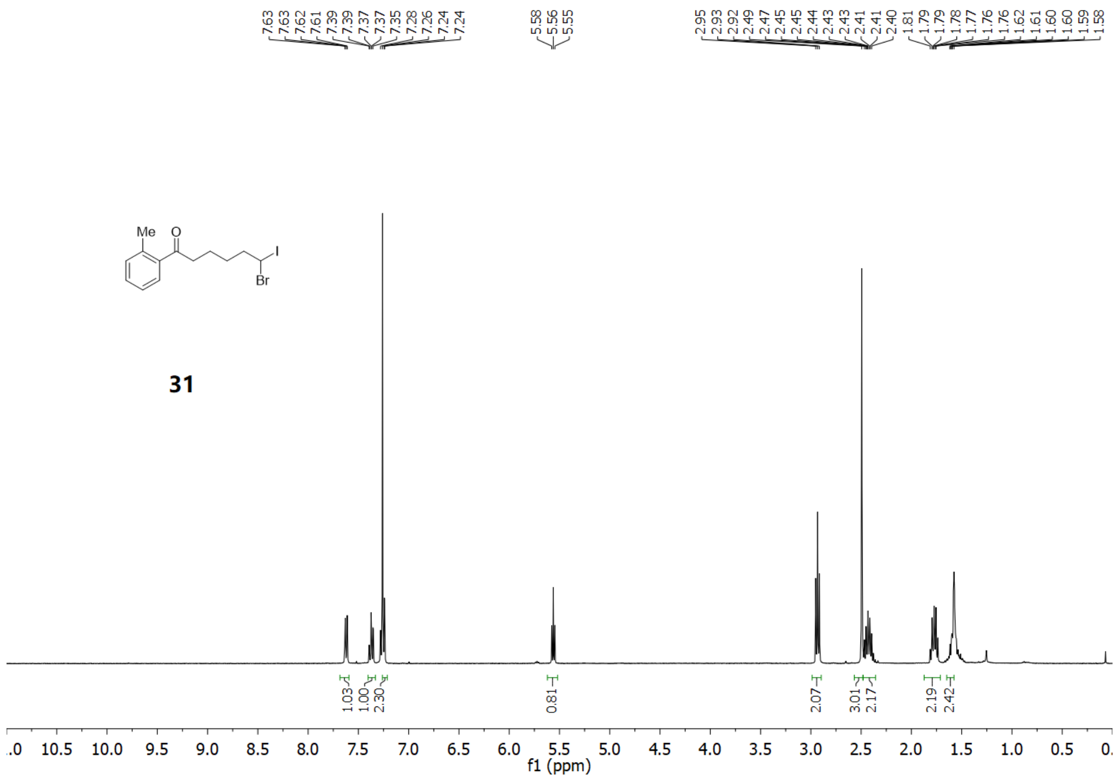

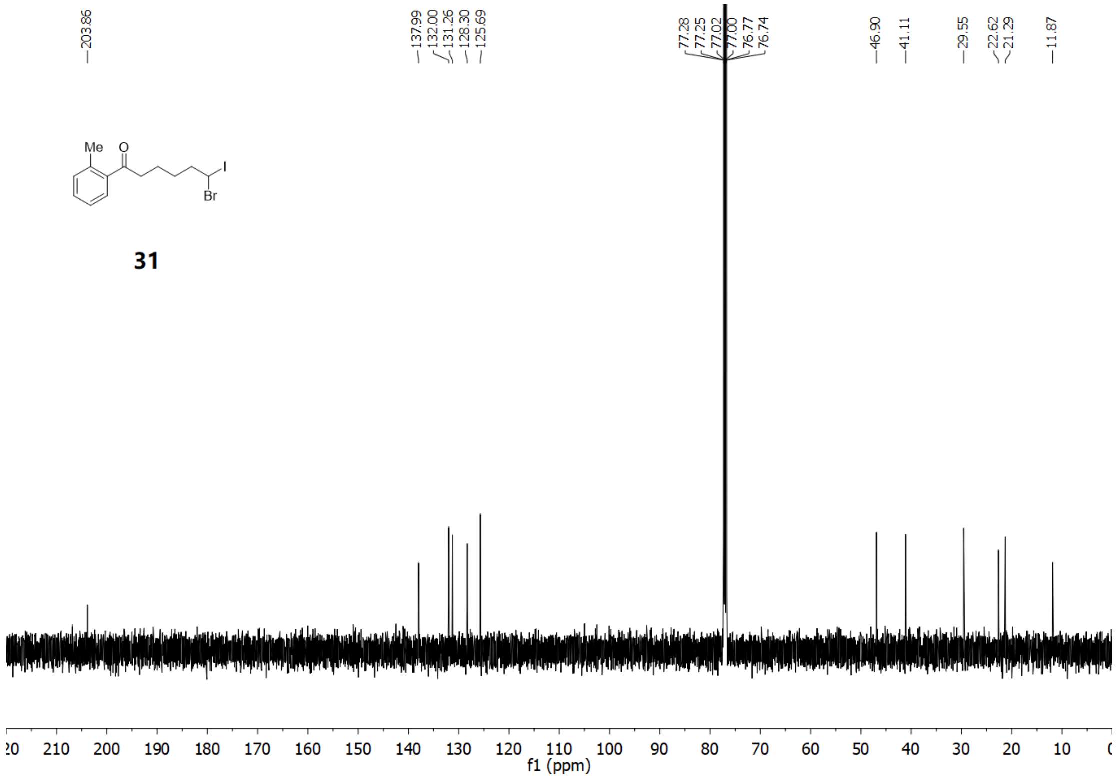

Supplementary Figure 40. ^{1}H and ^{13}C NMR spectra for compound **31**

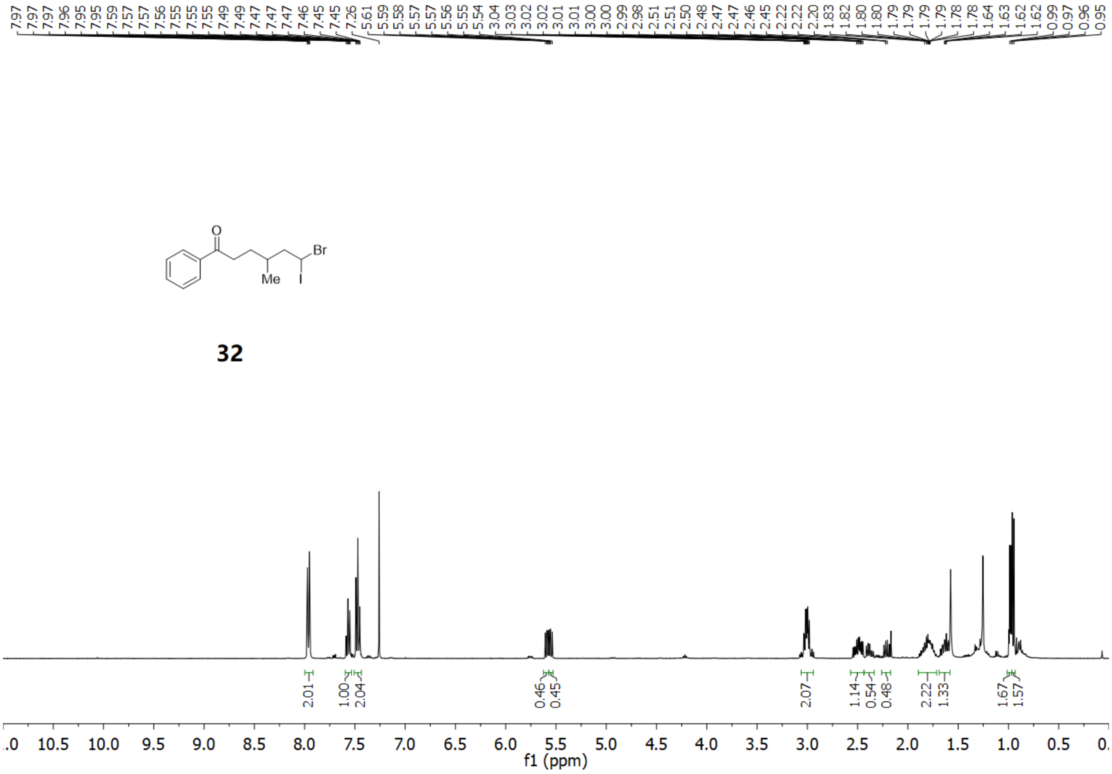

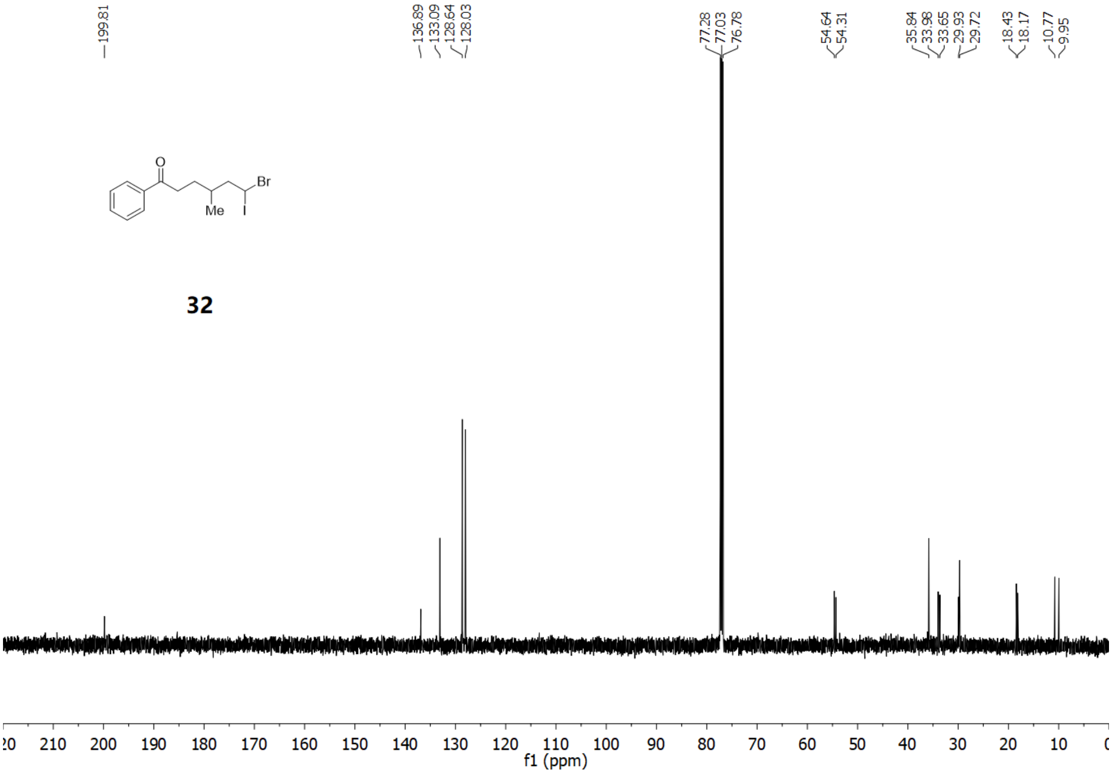

Supplementary Figure 41. ^{1}H and ^{13}C NMR spectra for compound **32**

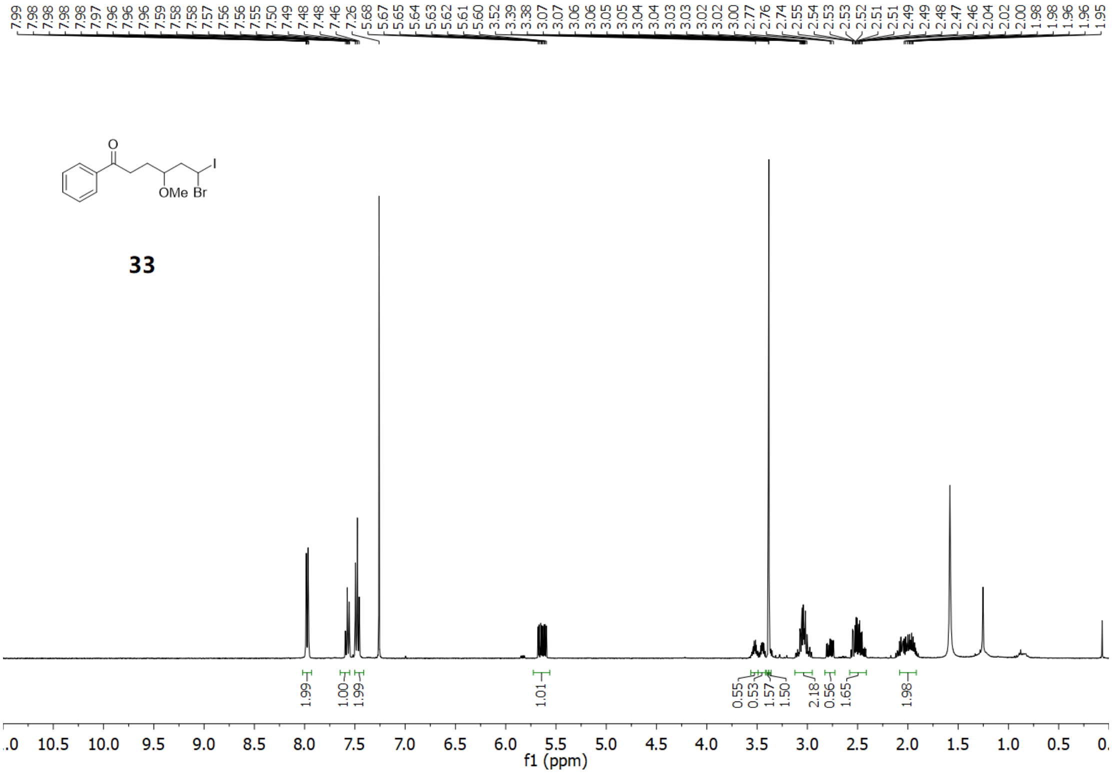

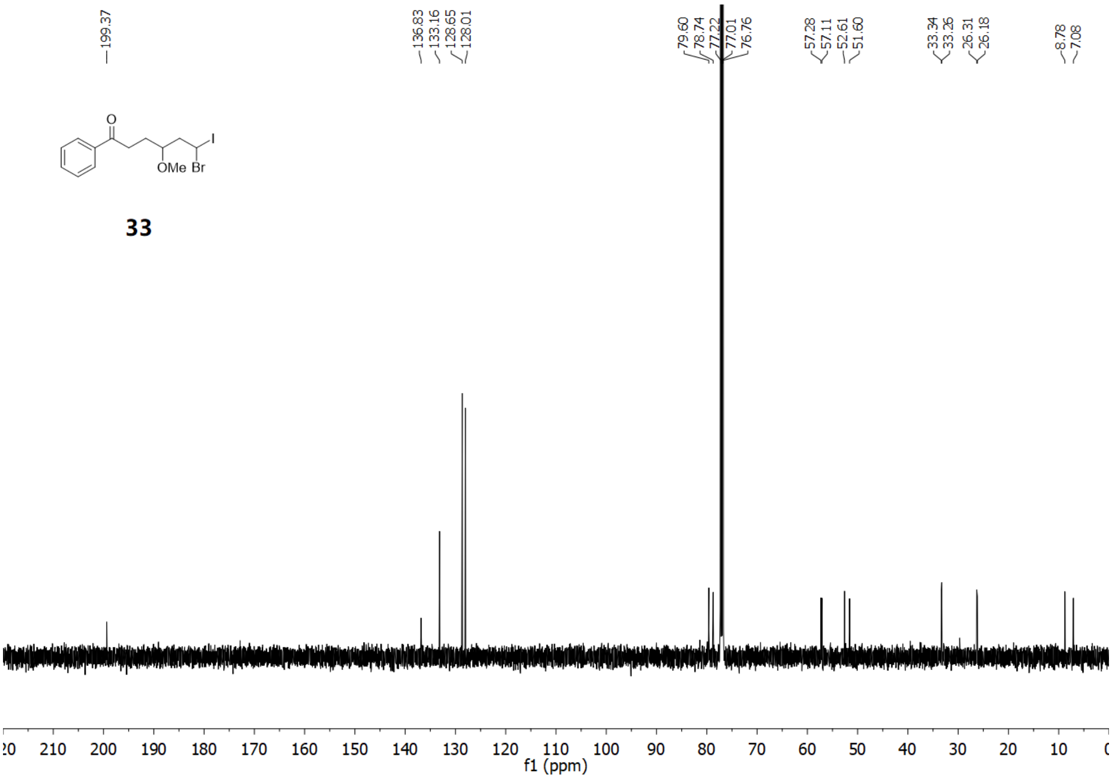

Supplementary Figure 42. ^{1}H and ^{13}C NMR spectra for compound **33**

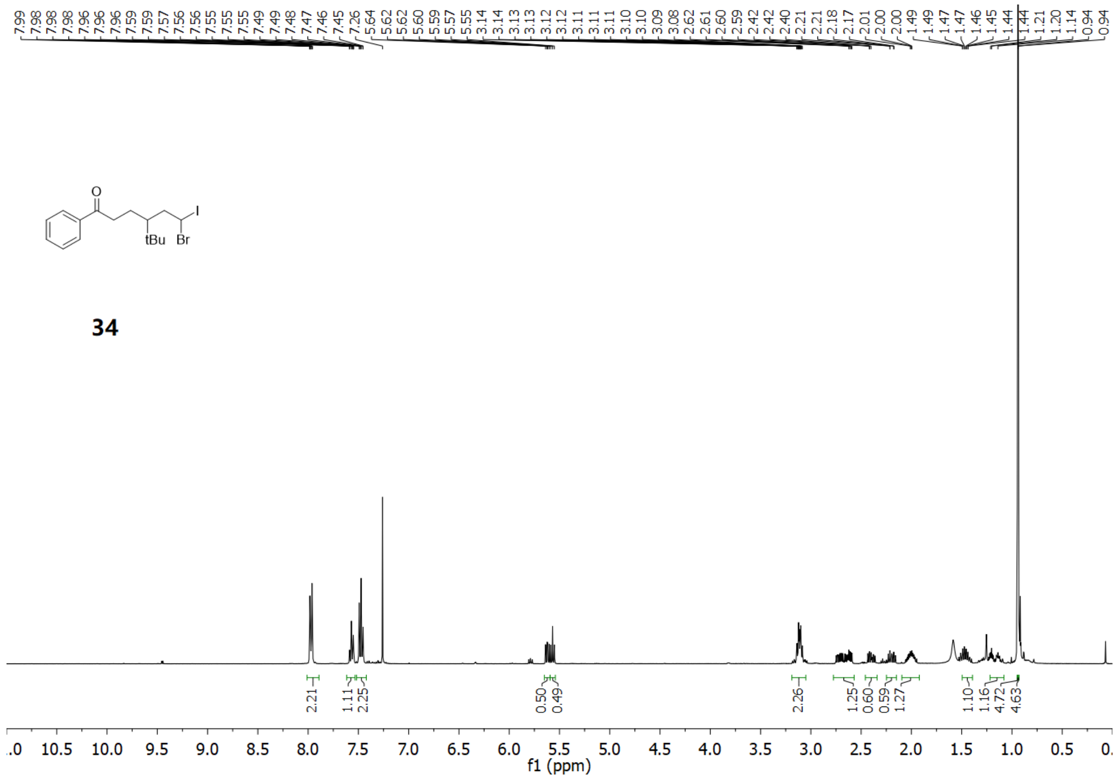

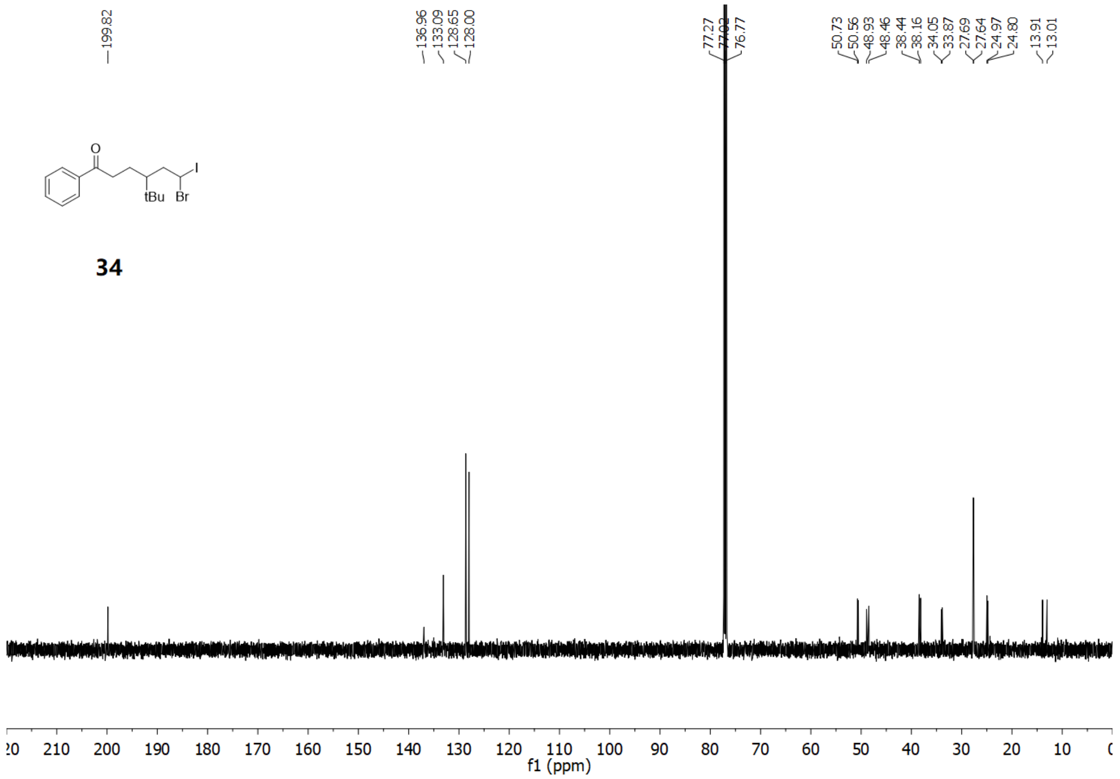

Supplementary Figure 43. ^{1}H and ^{13}C NMR spectra for compound **34**

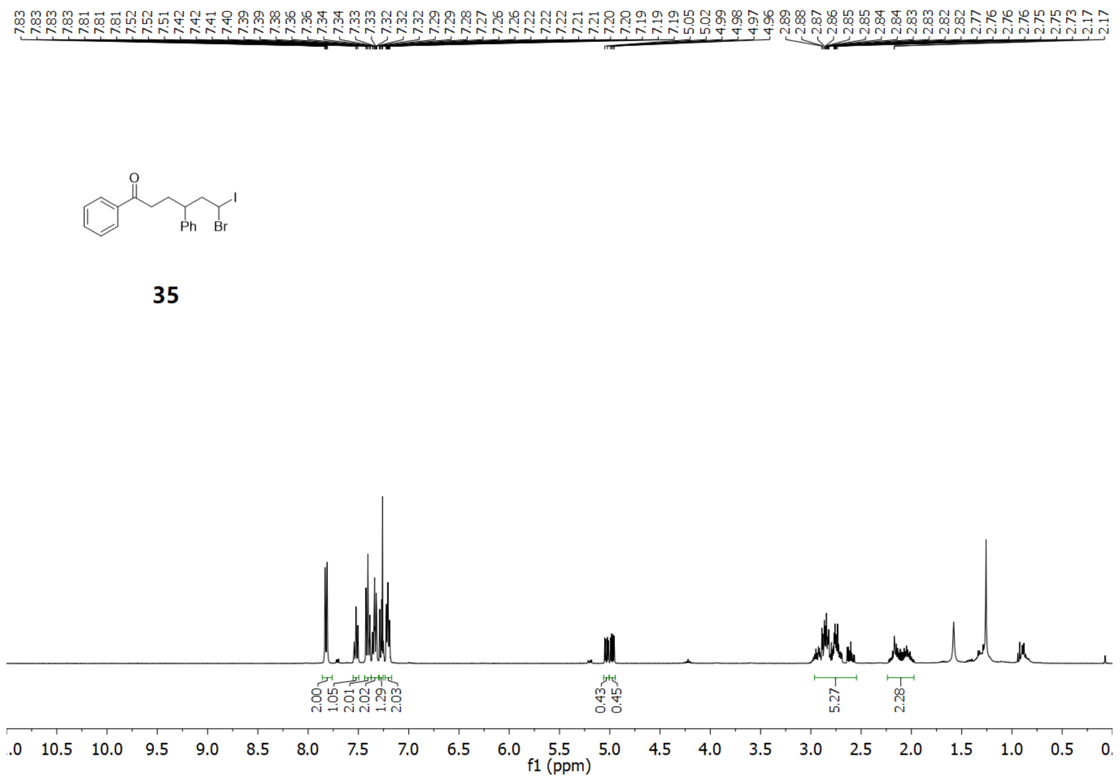

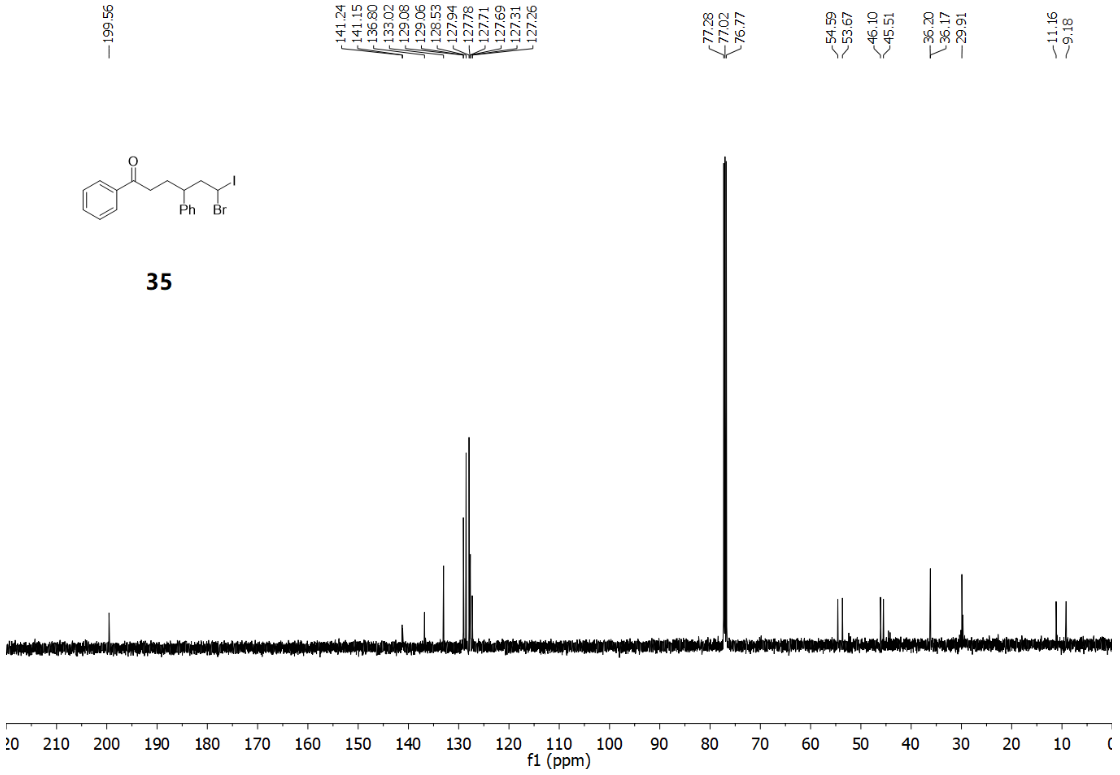

Supplementary Figure 44. ^{1}H and ^{13}C NMR spectra for compound **35**

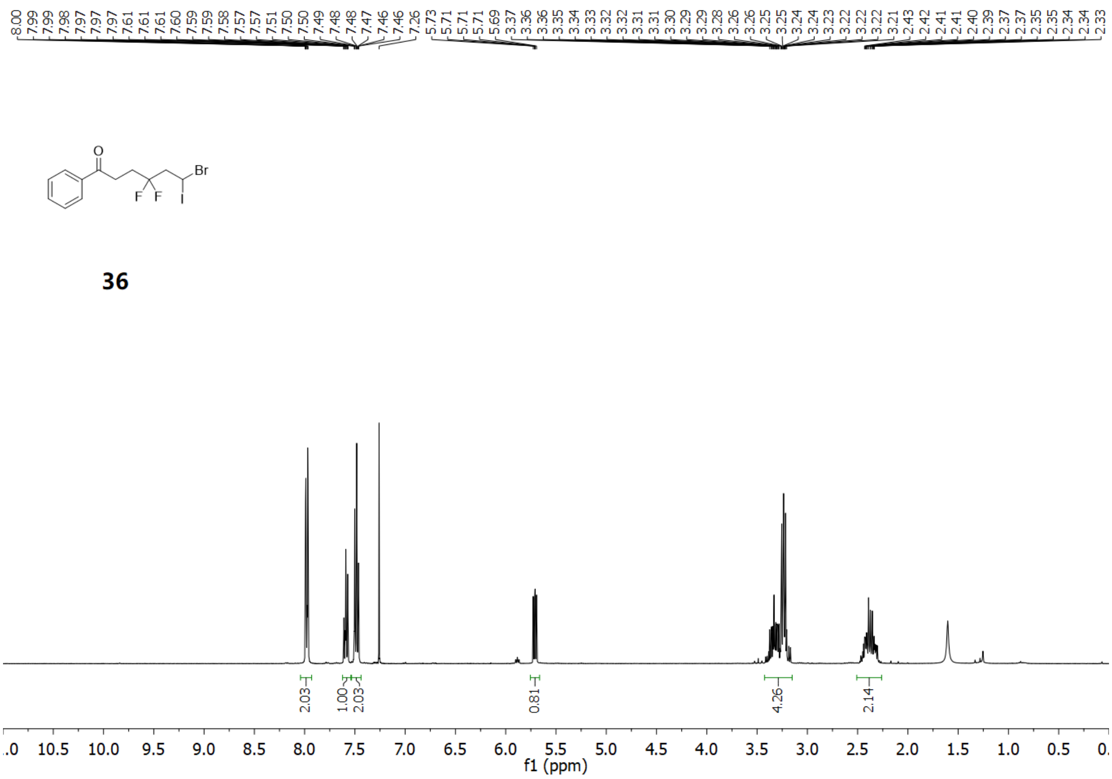

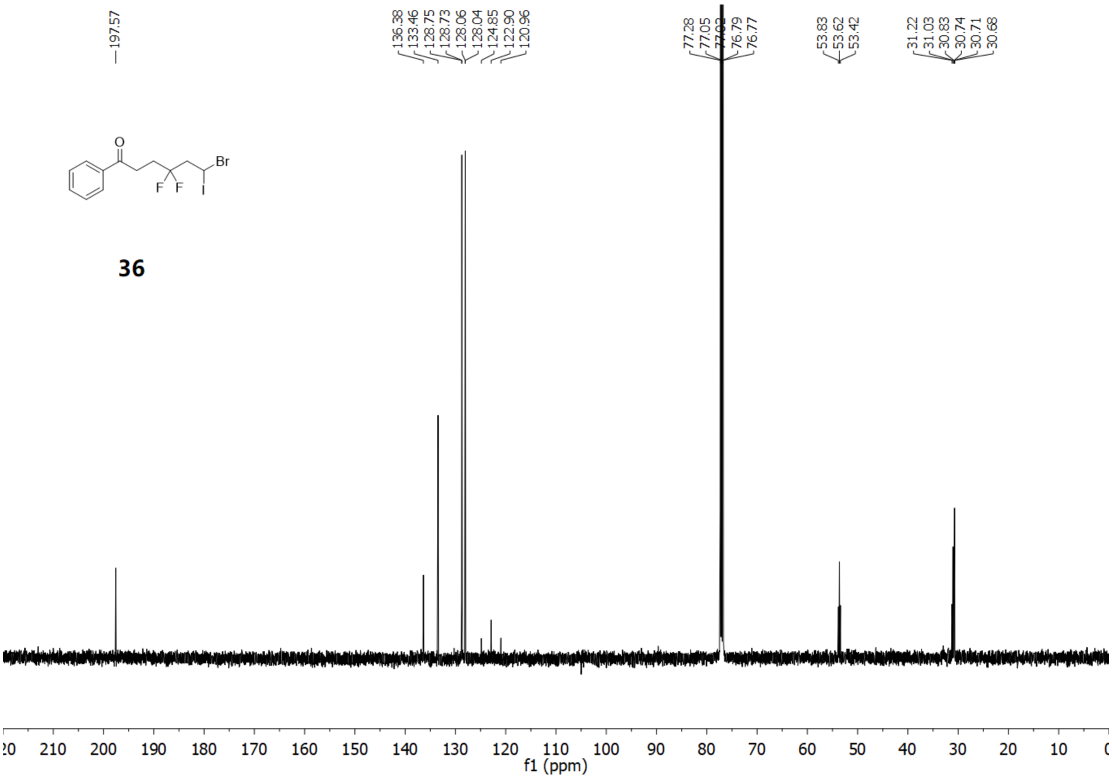

Supplementary Figure 45. ^{1}H and ^{13}C NMR spectra for compound **36**

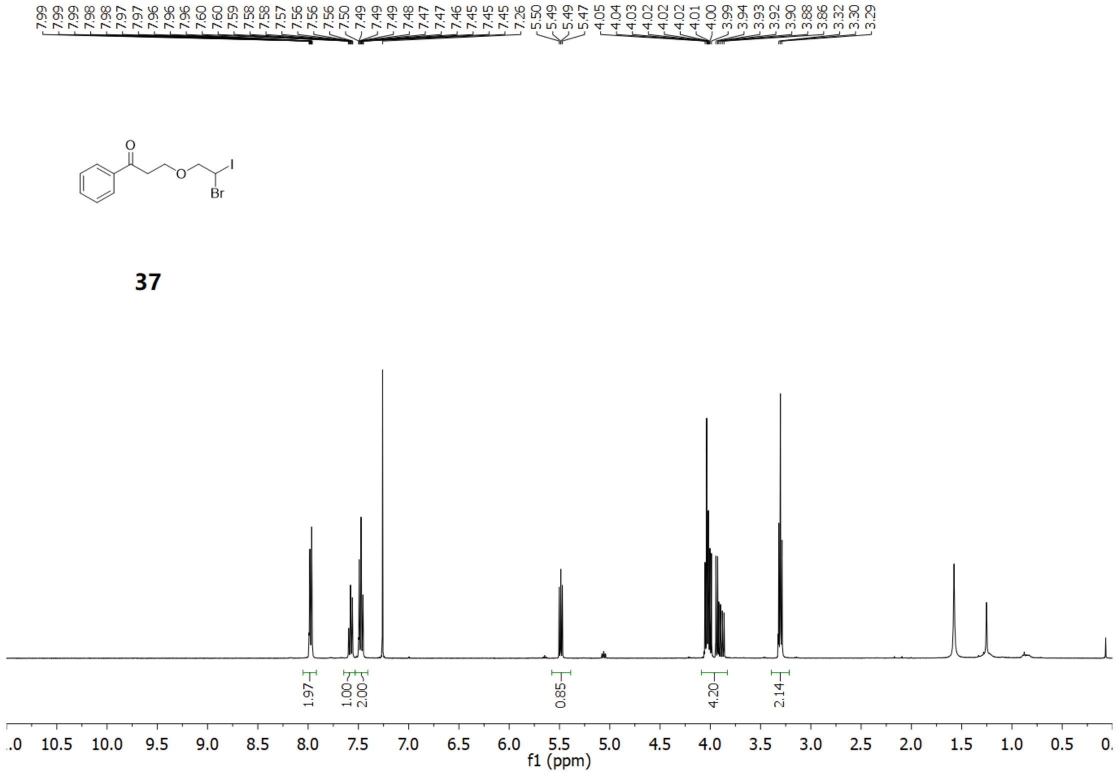

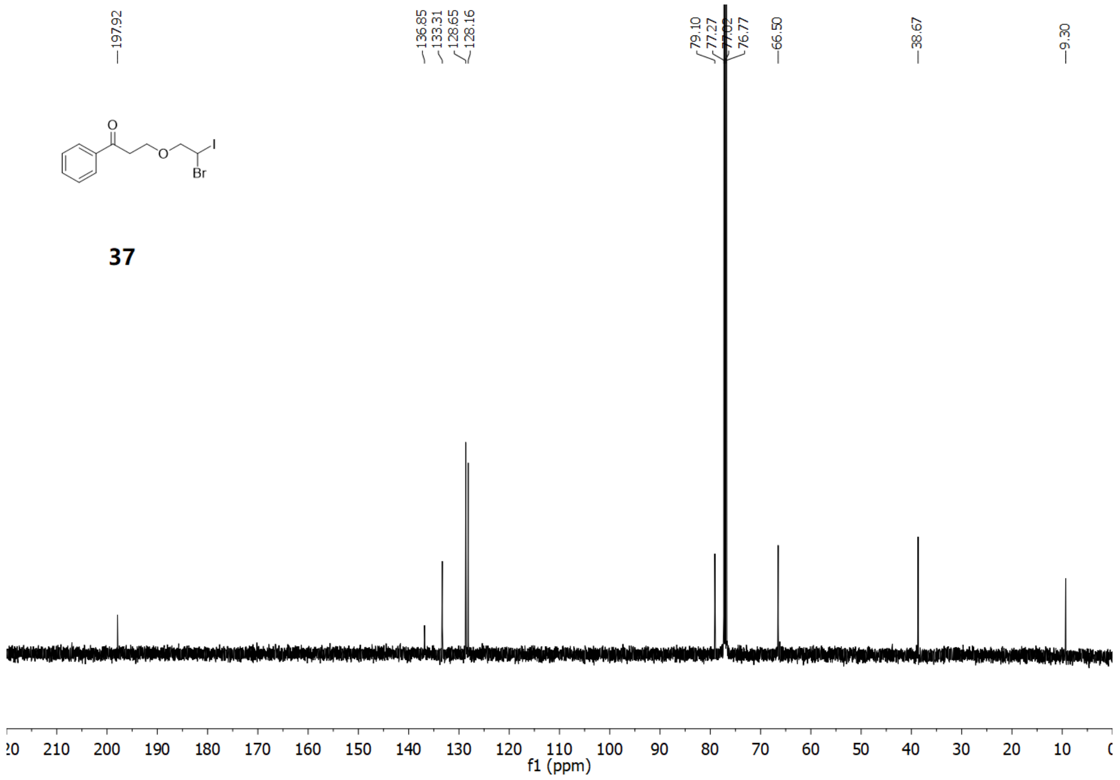

Supplementary Figure 46. ^{1}H and ^{13}C NMR spectra for compound **37**

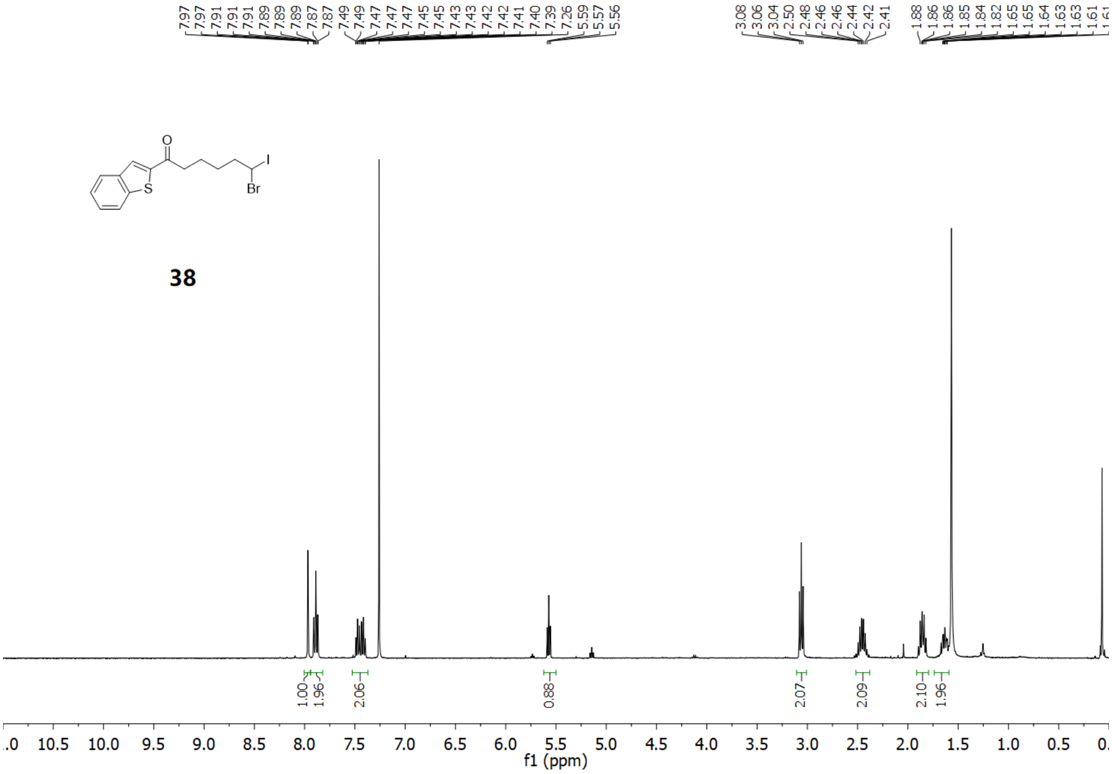

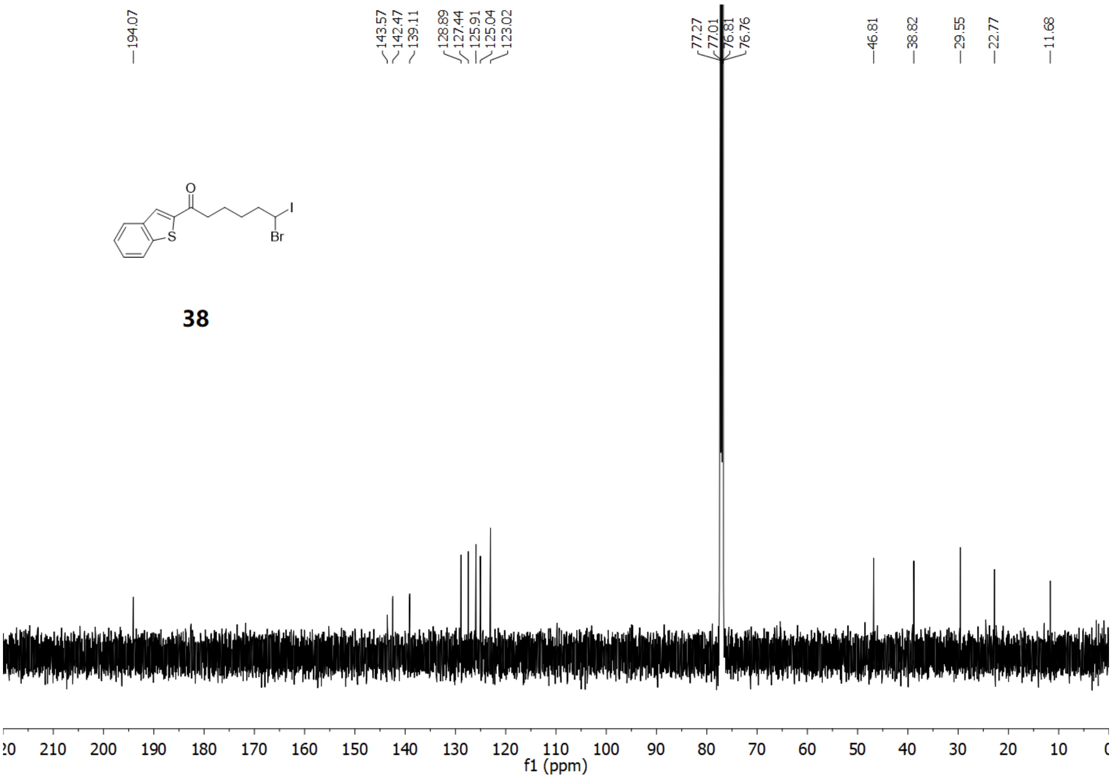

Supplementary Figure 47. ^{1}H and ^{13}C NMR spectra for compound **38**

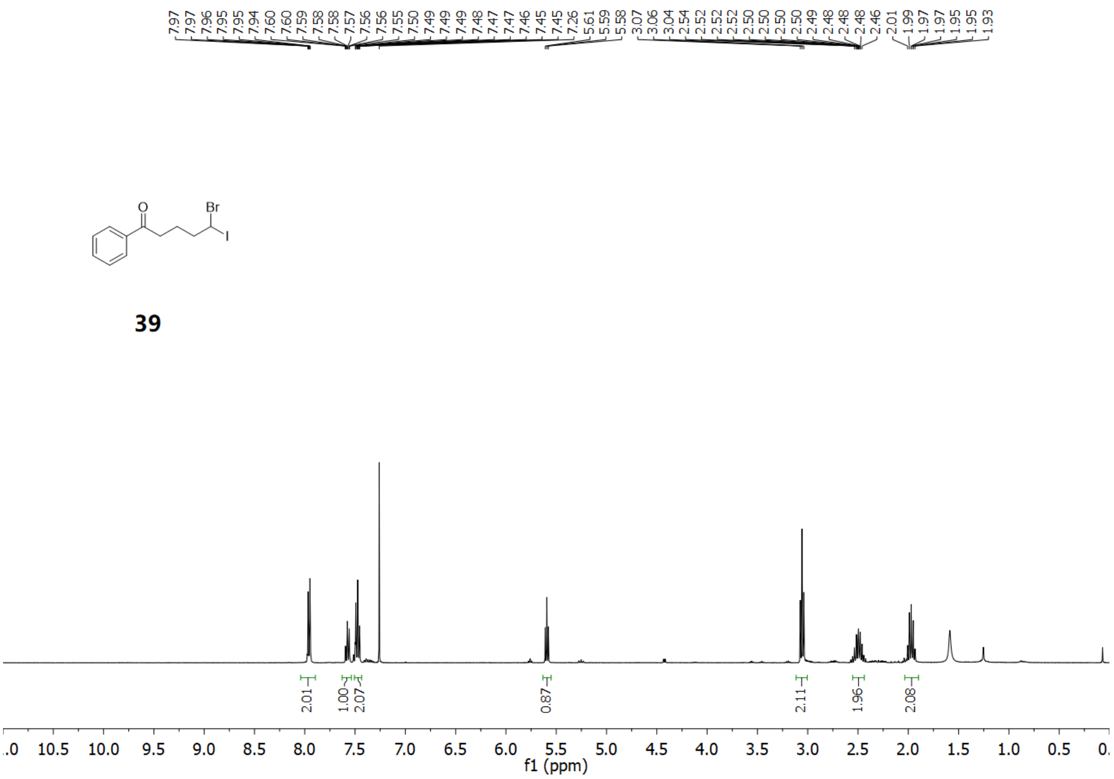

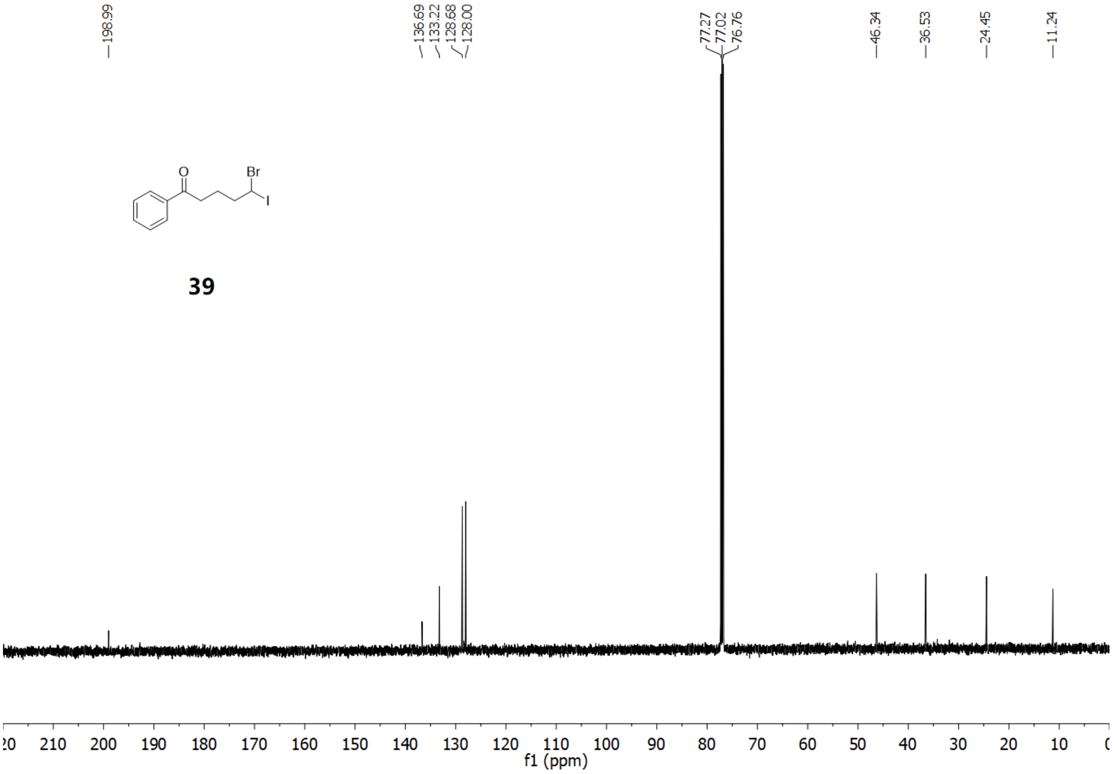

Supplementary Figure 48. ^{1}H and ^{13}C NMR spectra for compound **39**

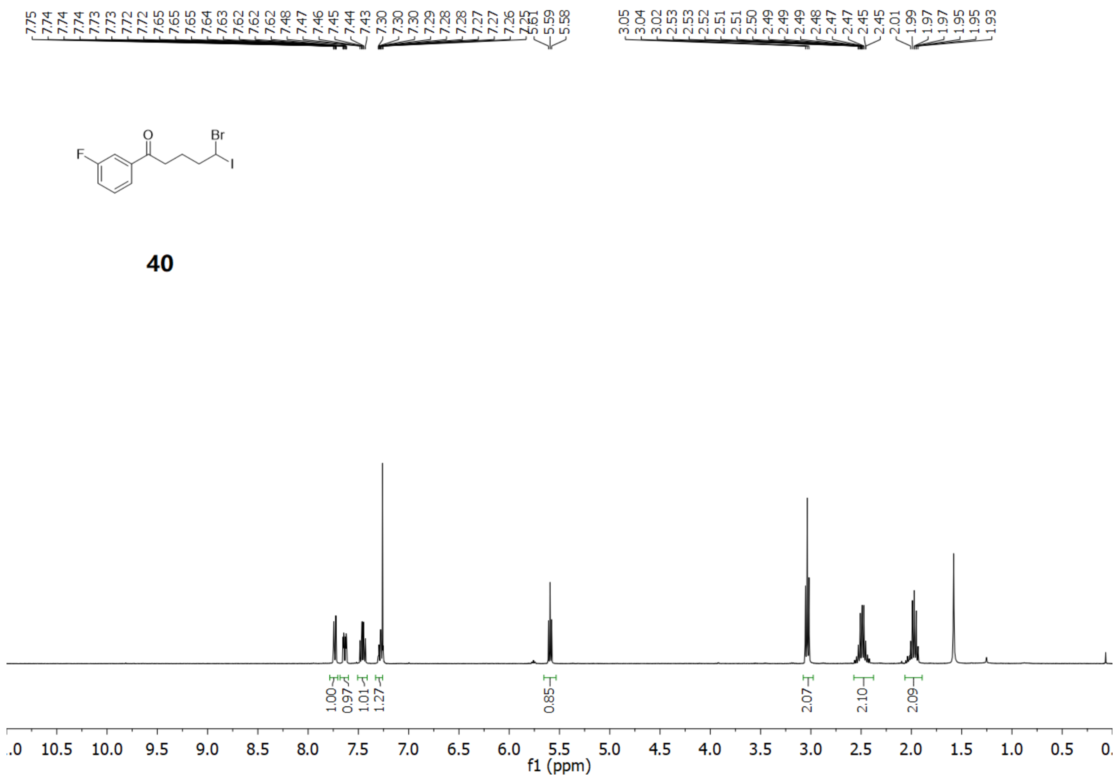

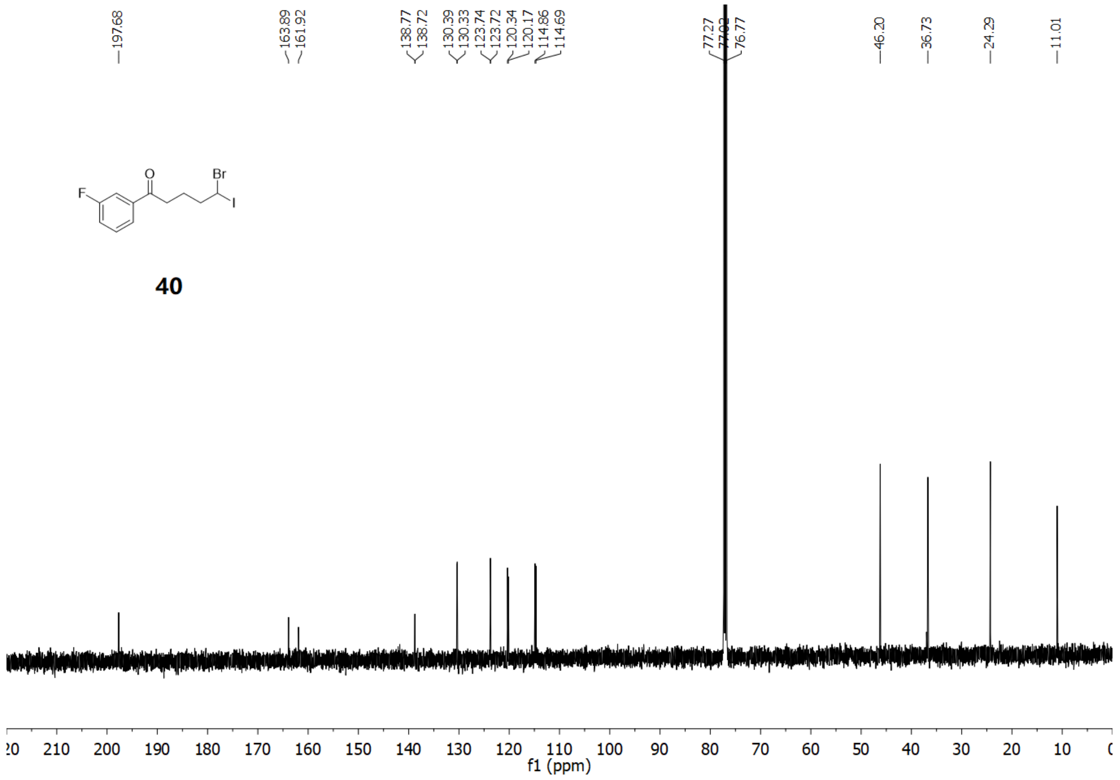

Supplementary Figure 49. ^{1}H and ^{13}C NMR spectra for compound **40**

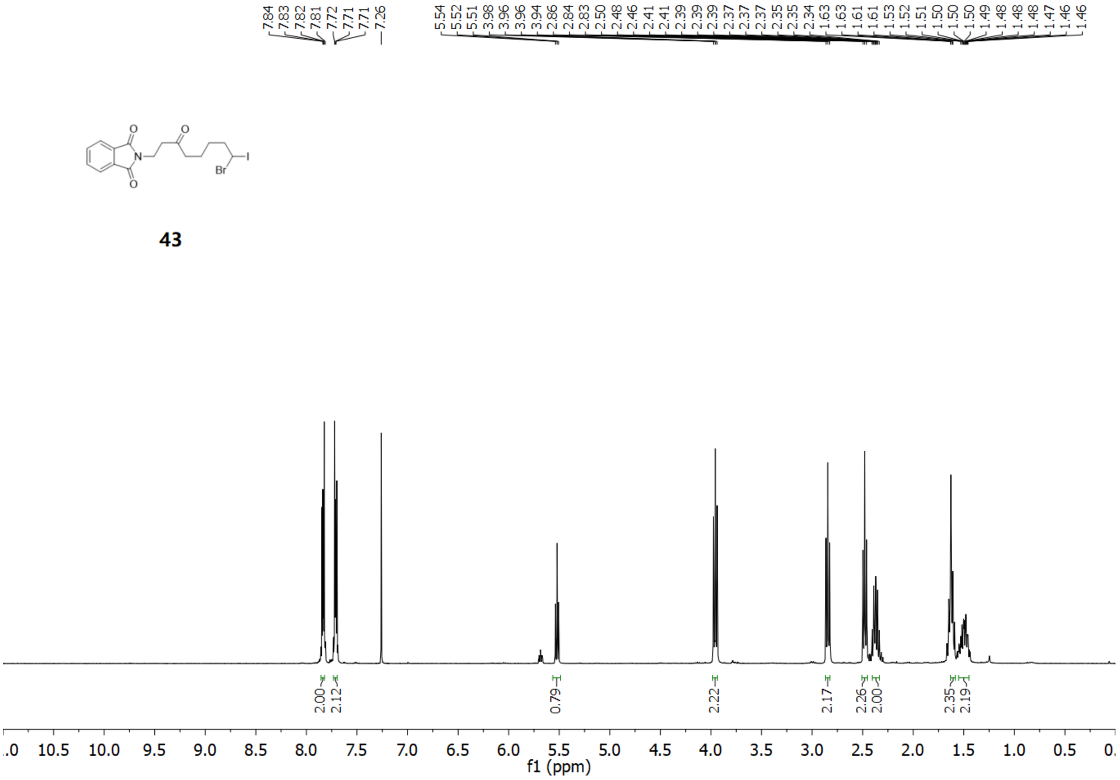

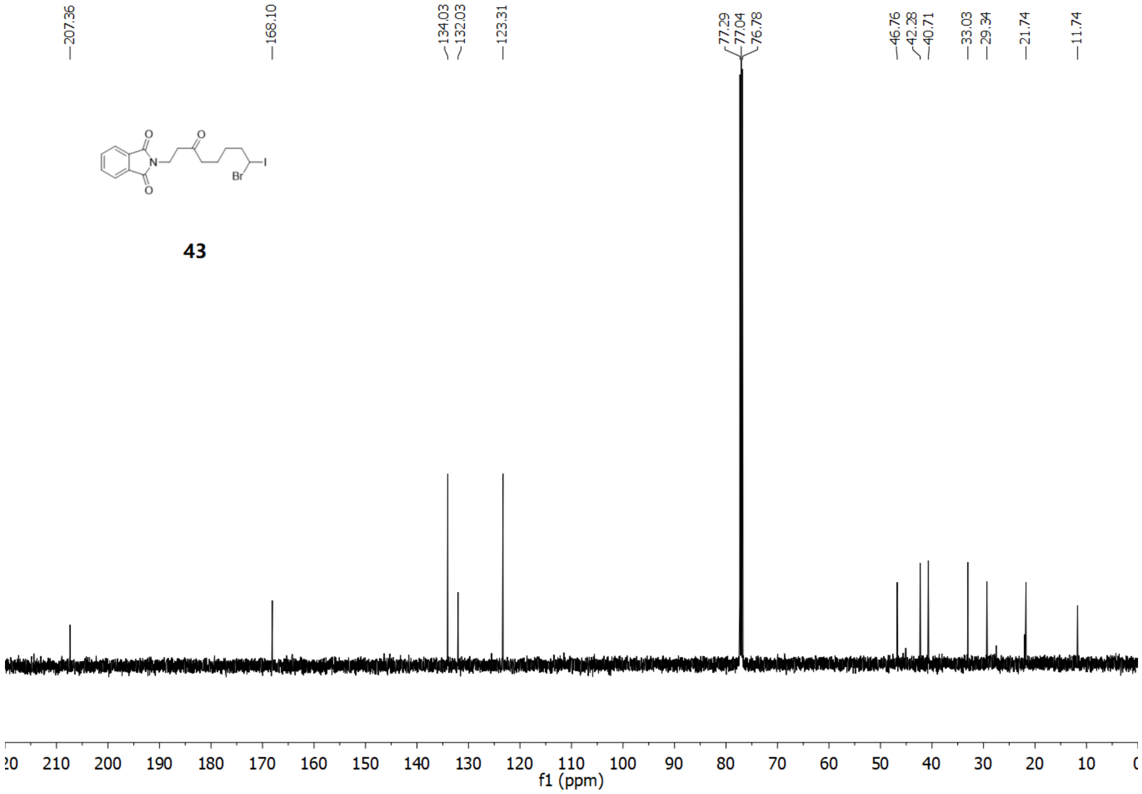

Supplementary Figure 50. ^{1}H and ^{13} C NMR spectra for compound **43**

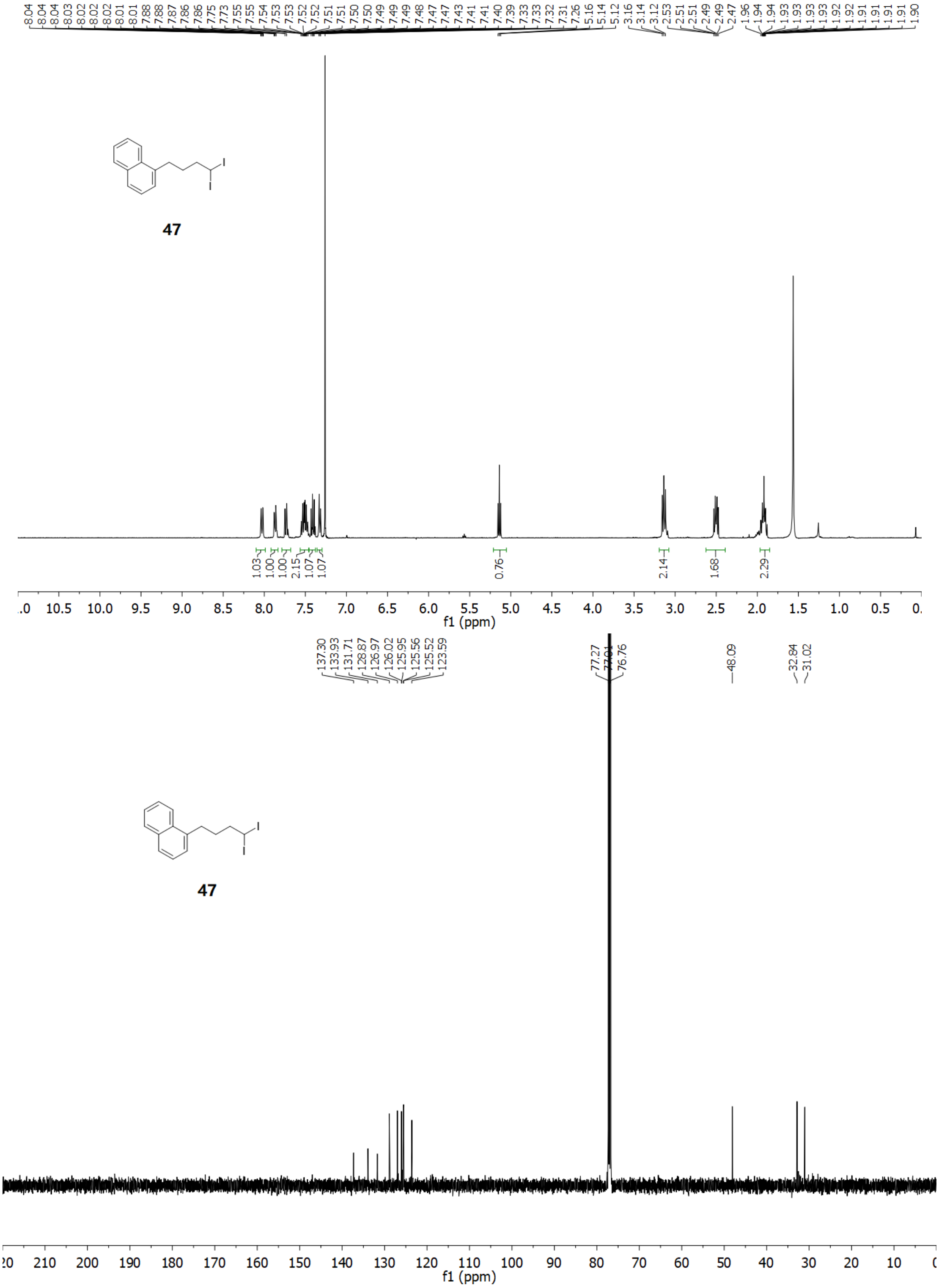

Supplementary Figure 51. ^{1}H and ^{13}C NMR spectra for compound **47**

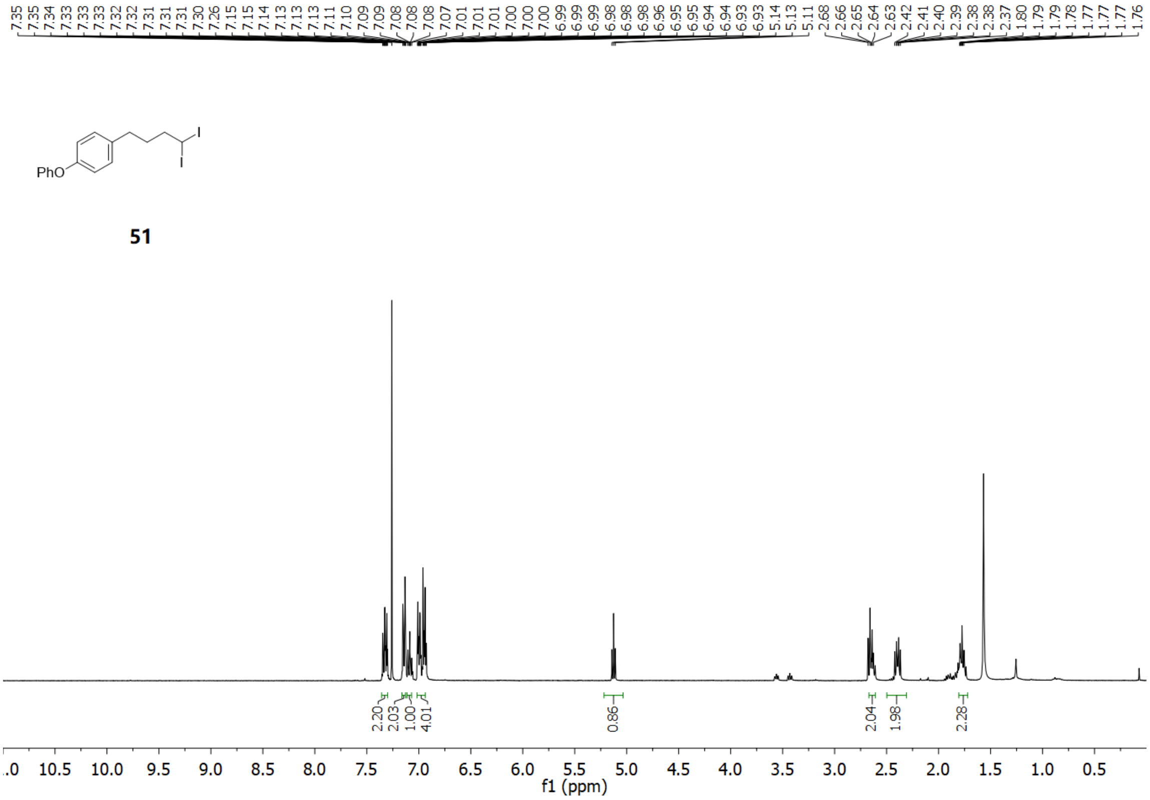

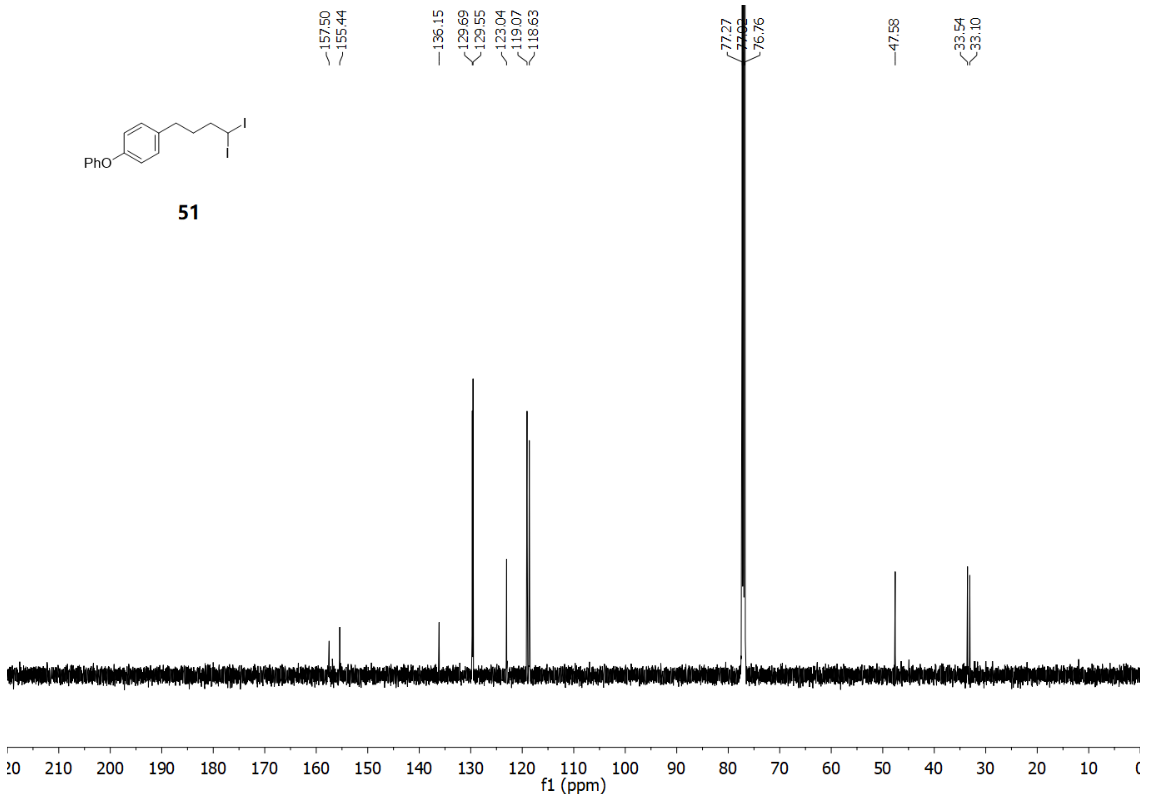

Supplementary Figure 52. ^{1}H and ^{13}C NMR spectra for compound **51**

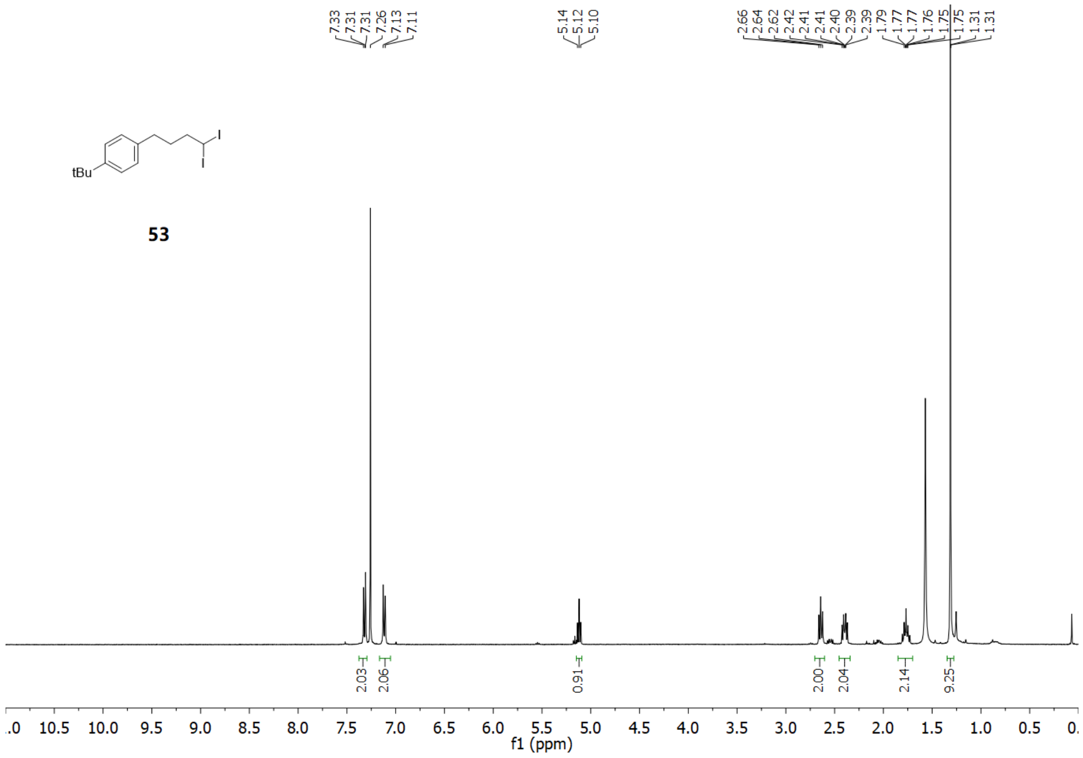

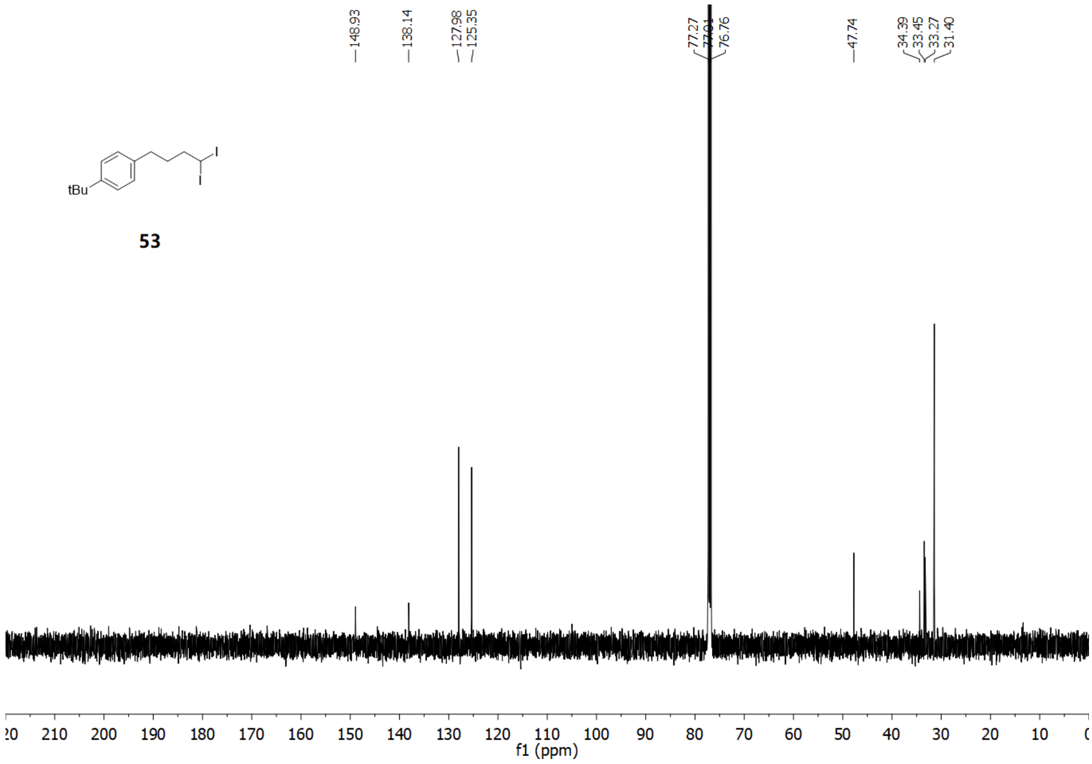

Supplementary Figure 53. ^{1}H and ^{13}C NMR spectra for compound **53**

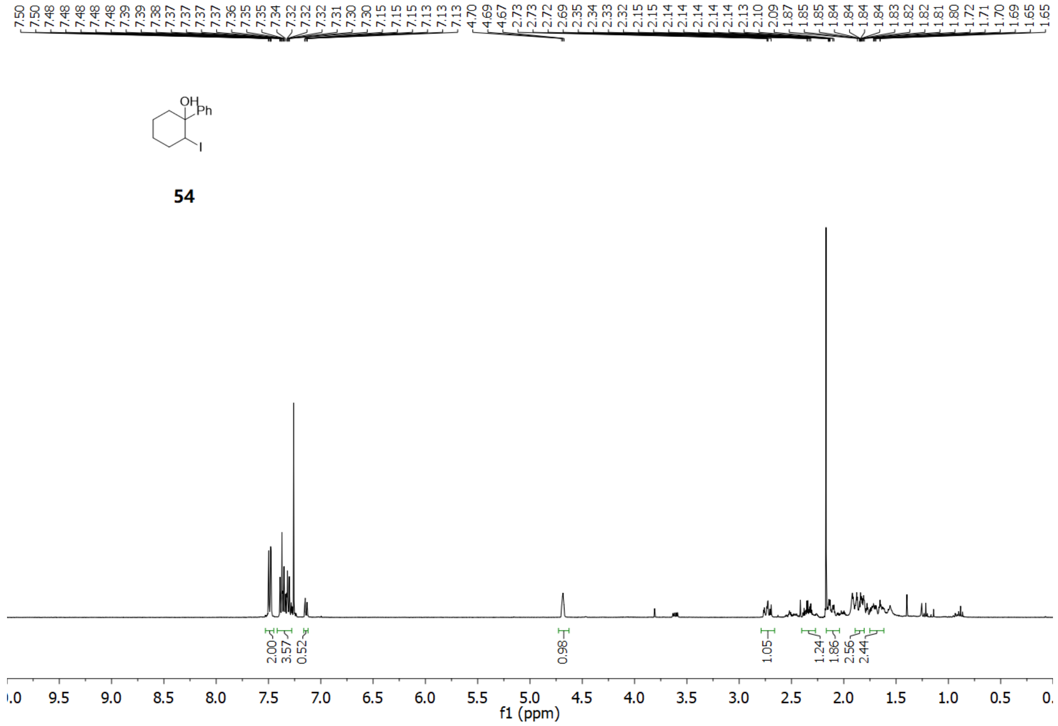

Supplementary Figure 54. ^{1}H spectra for compound **54**

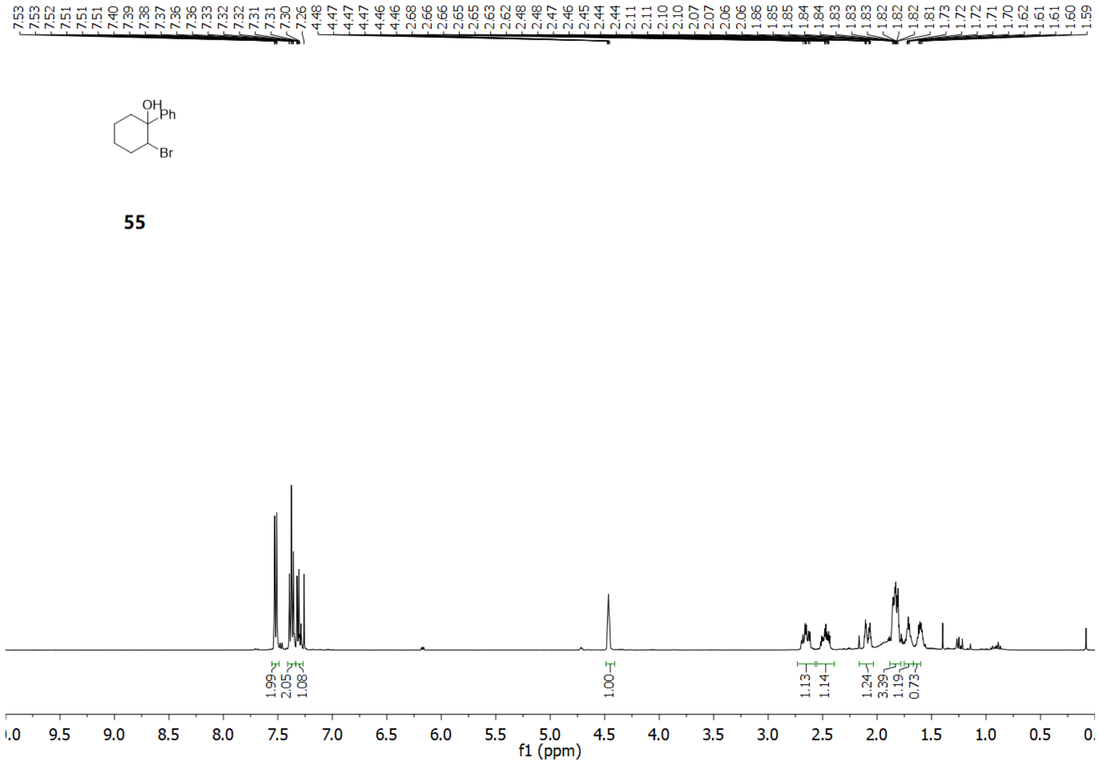

Supplementary Figure 55. ^{1}H NMR spectra for compound **55**

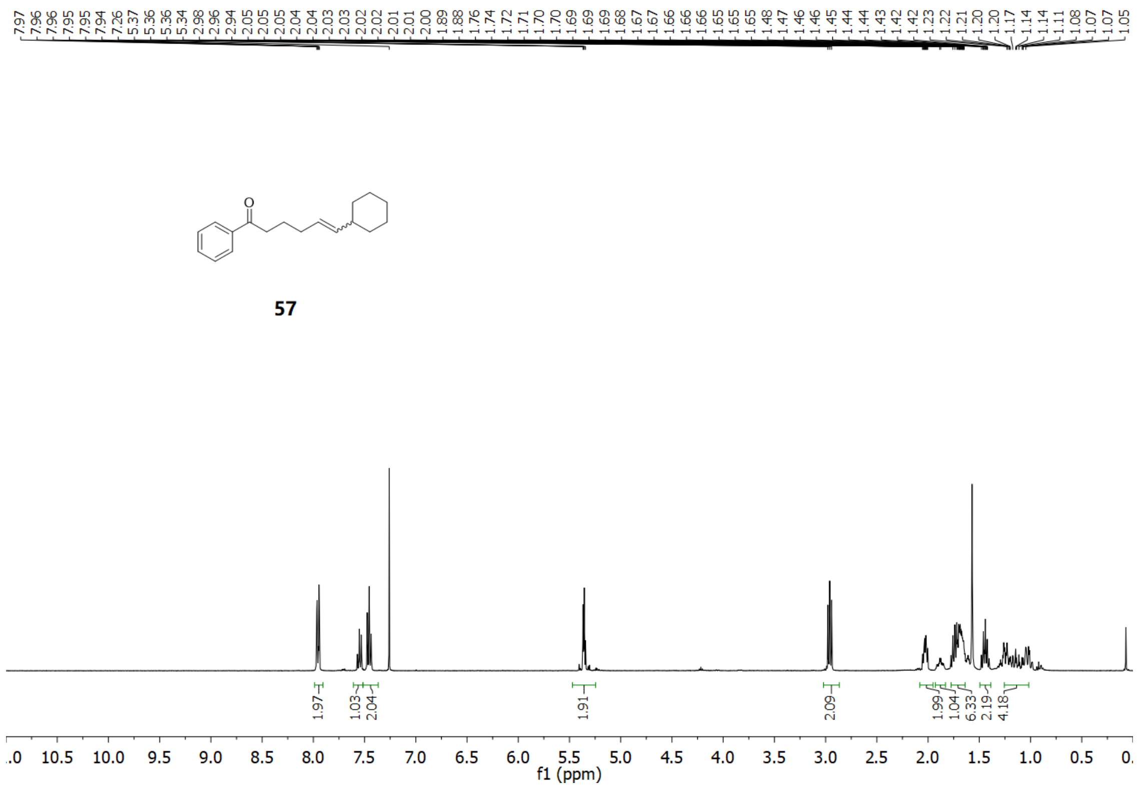

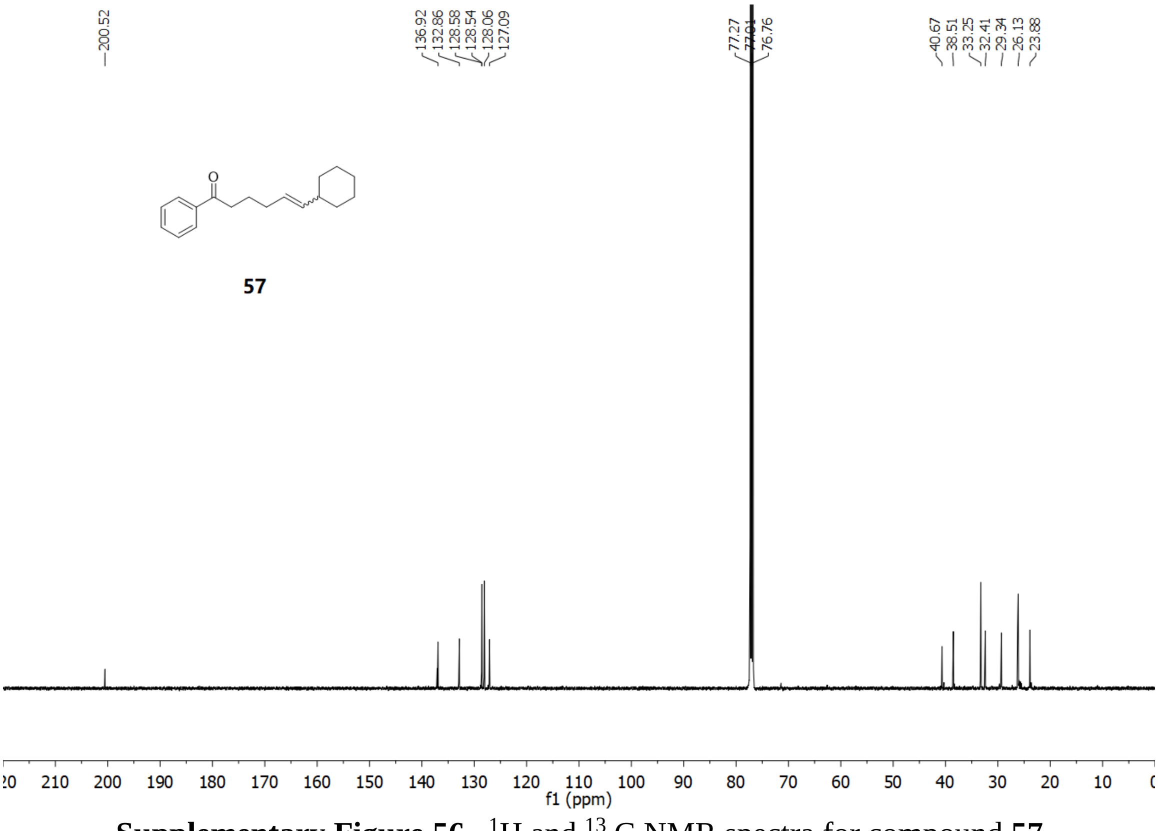

Supplementary Figure 56. ^{1}H and ^{13}C NMR spectra for compound **57**

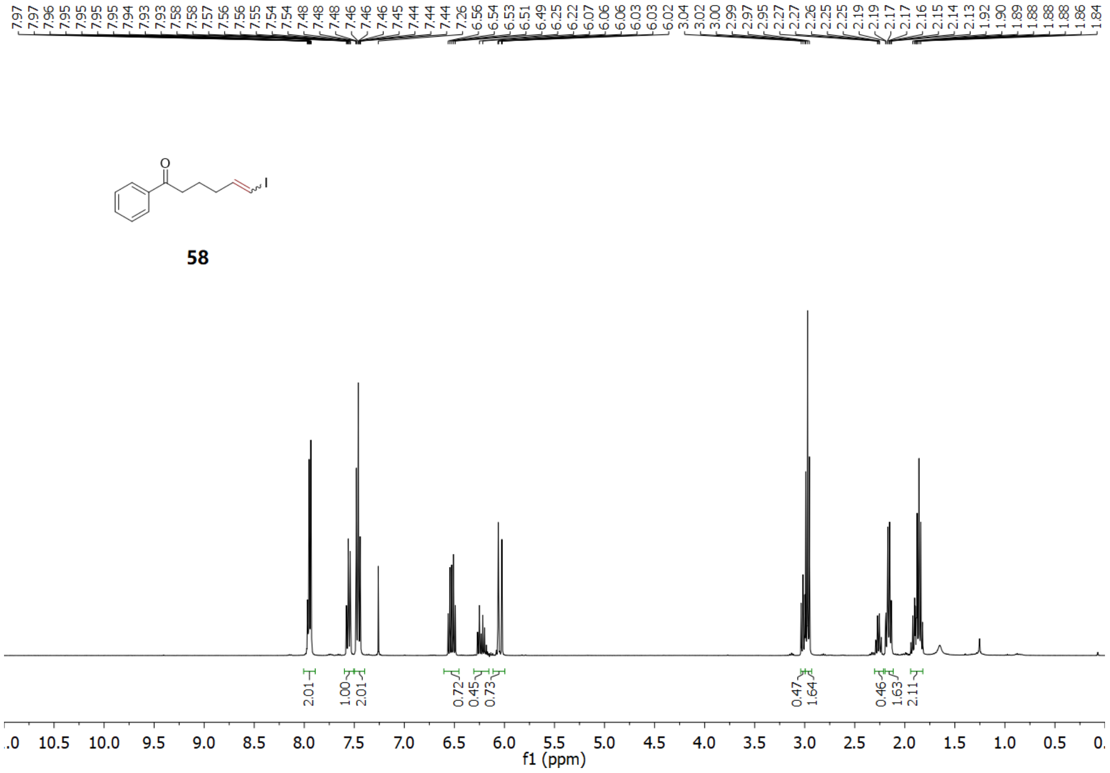

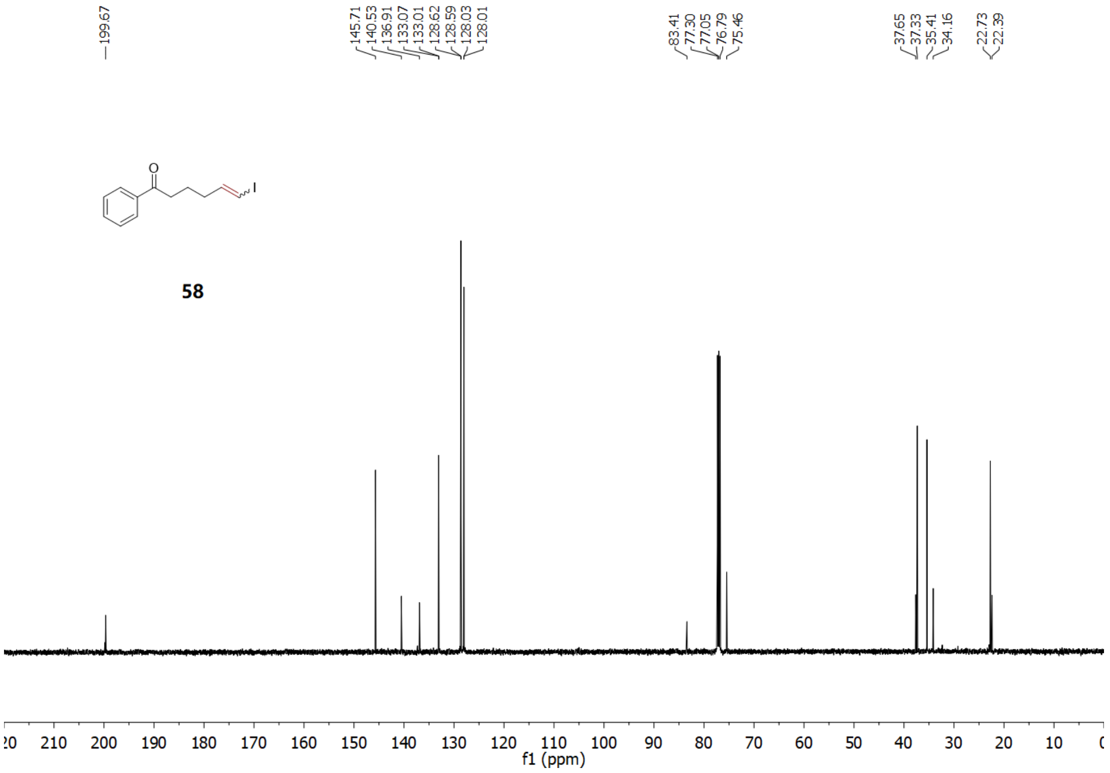

Supplementary Figure 57. ^{1}H and ^{13}C NMR spectra for compound **58**

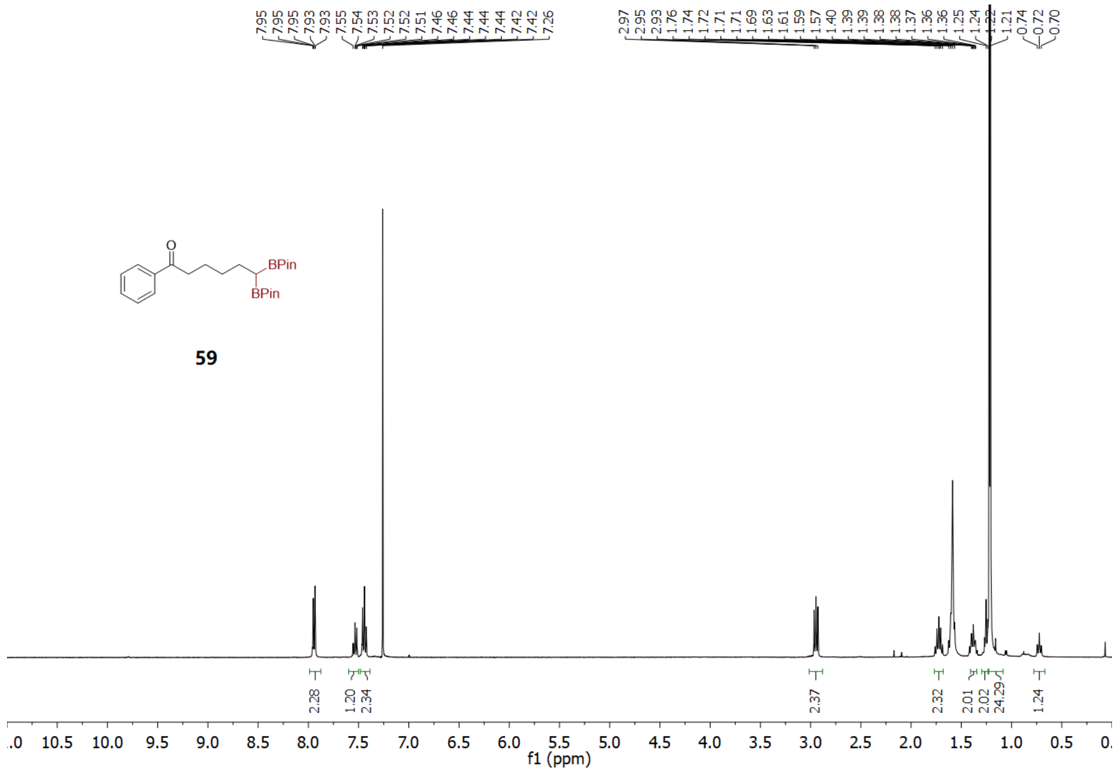

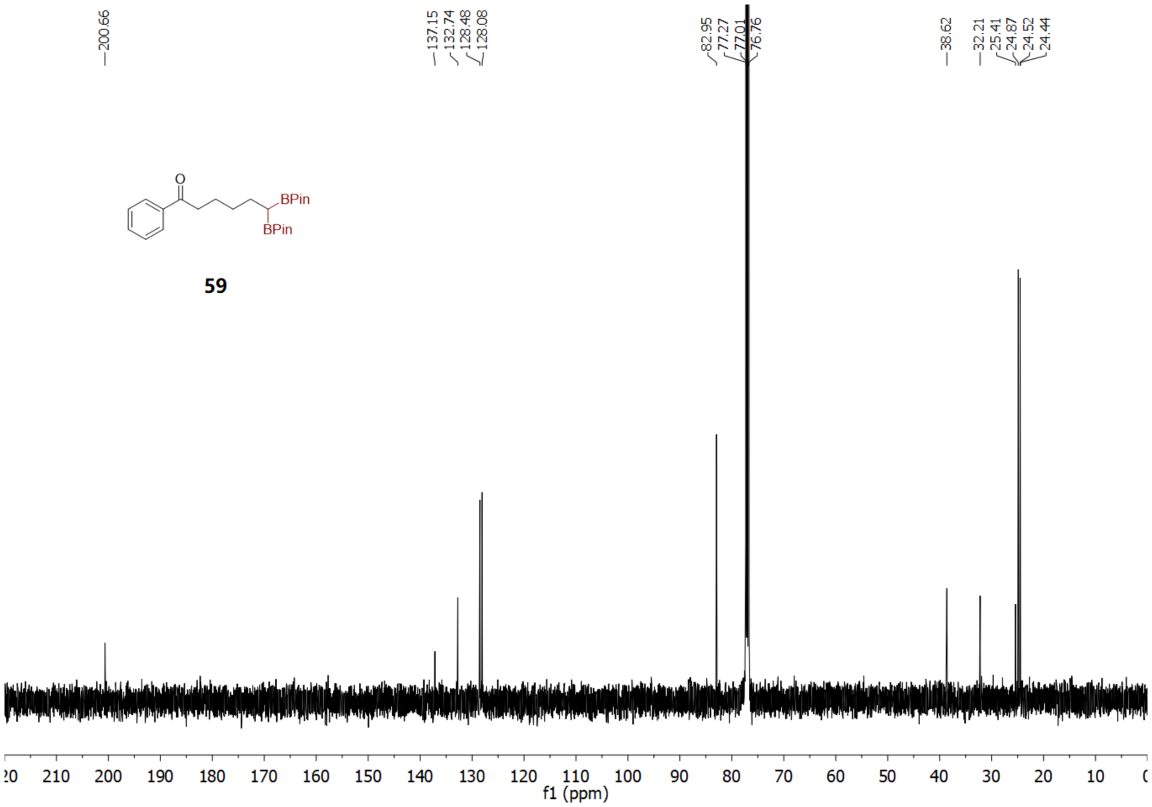

Supplementary Figure 58. ^{1}H and ^{13}C NMR spectra for compound **59**

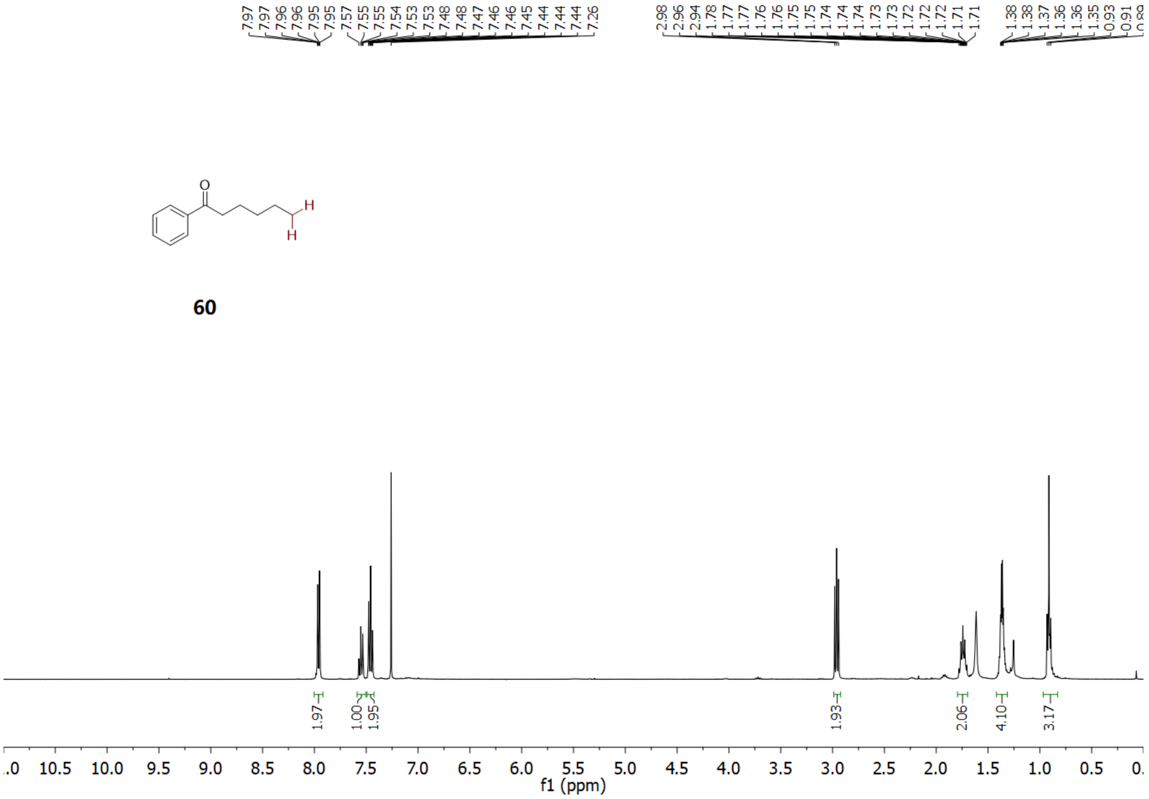

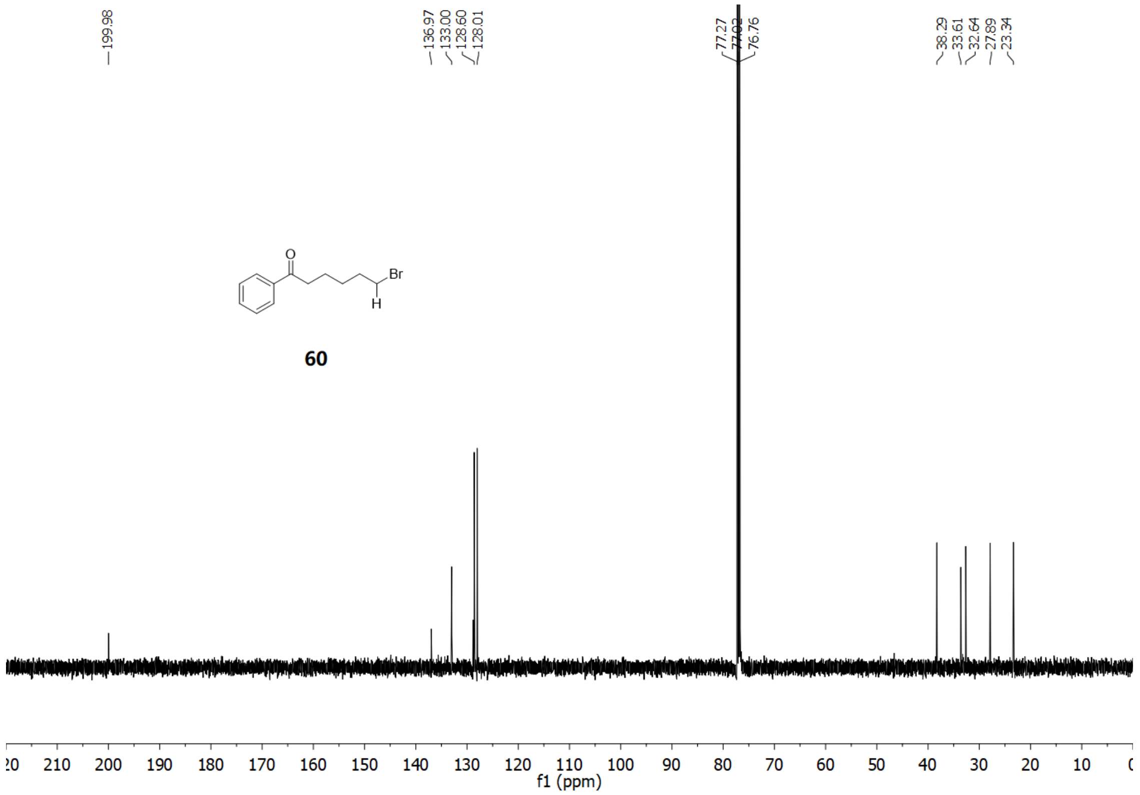

Supplementary Figure 59. ^{1}H and ^{13}C NMR spectra for compound **60**

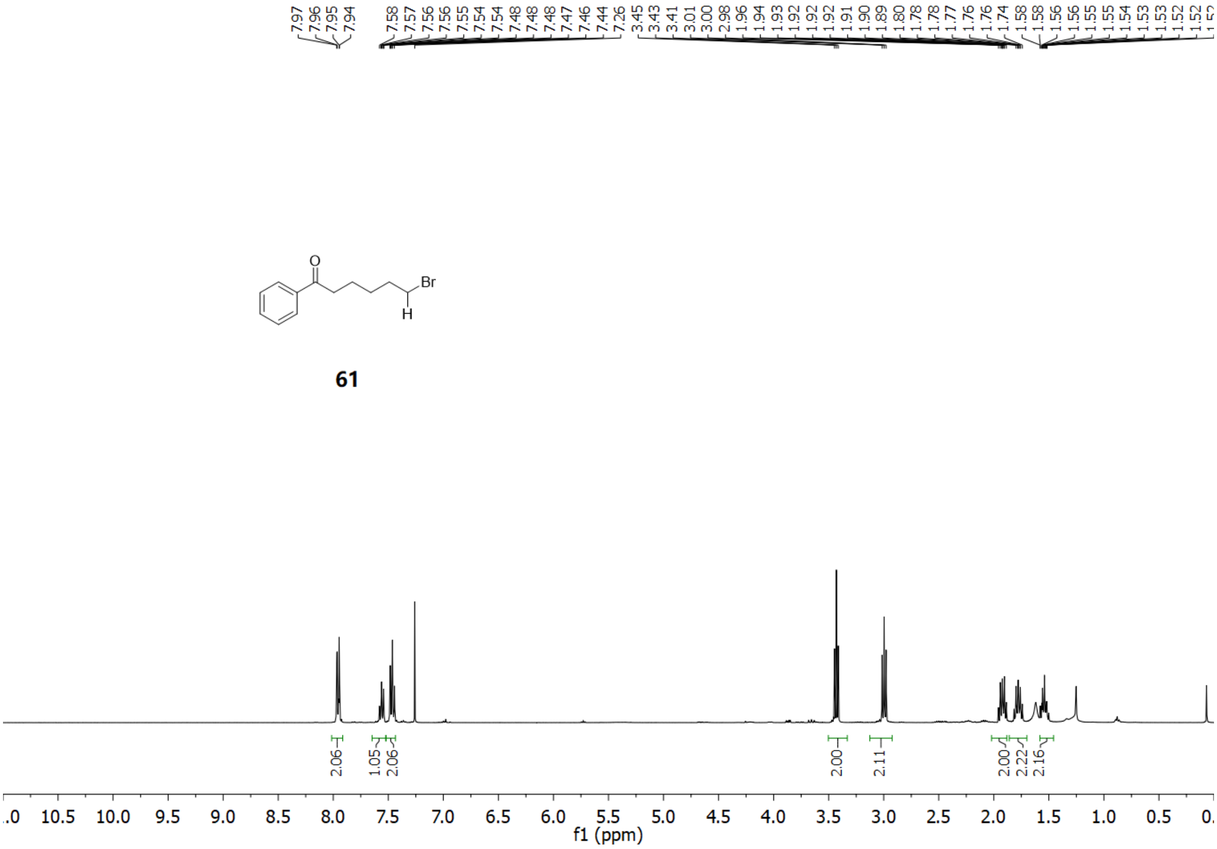

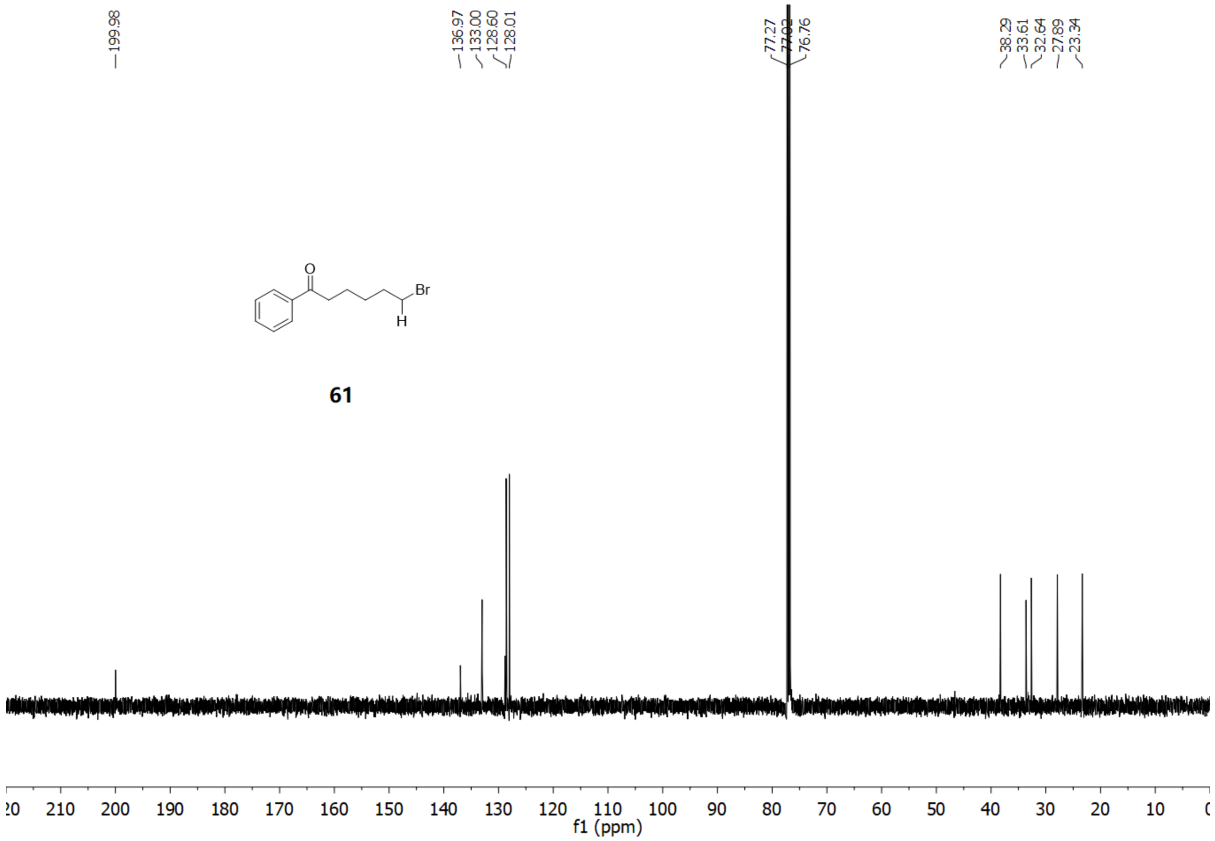

Supplementary Figure 60. ^{1}H and ^{13}C NMR spectra for compound **61**

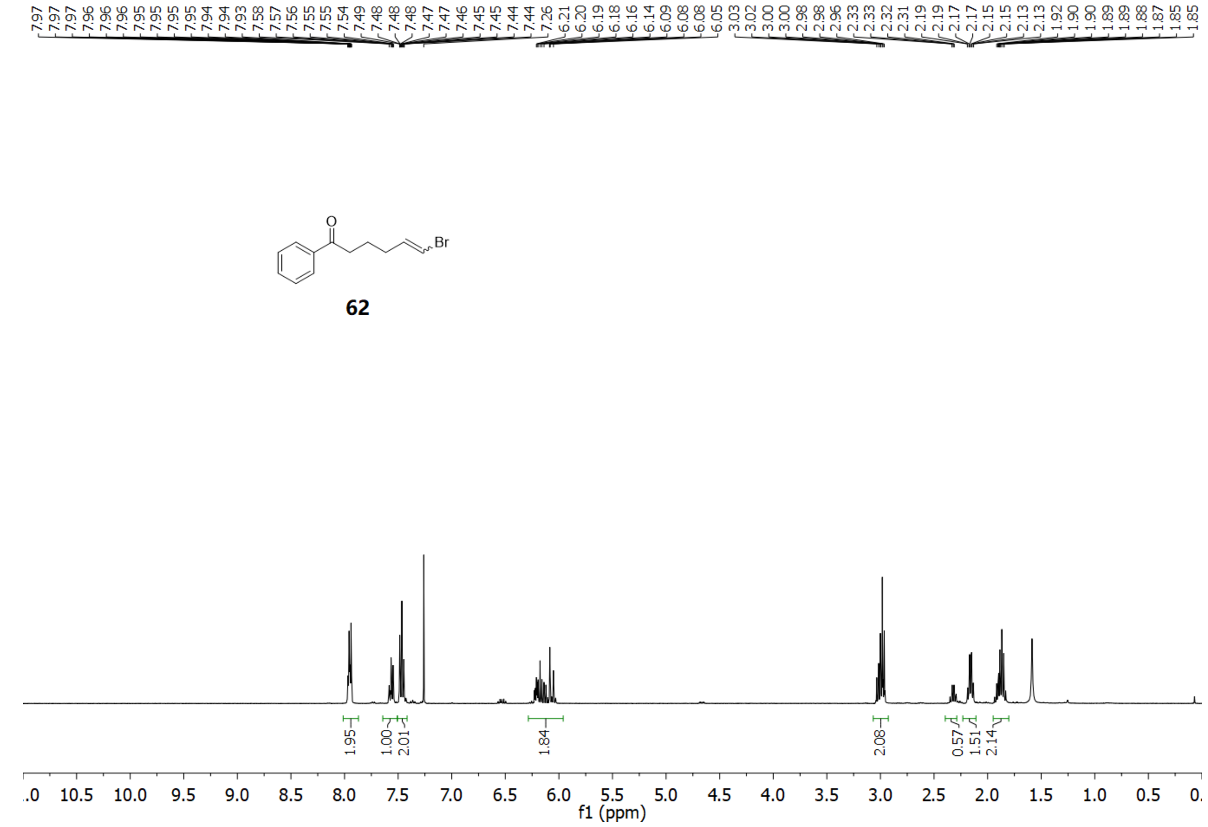

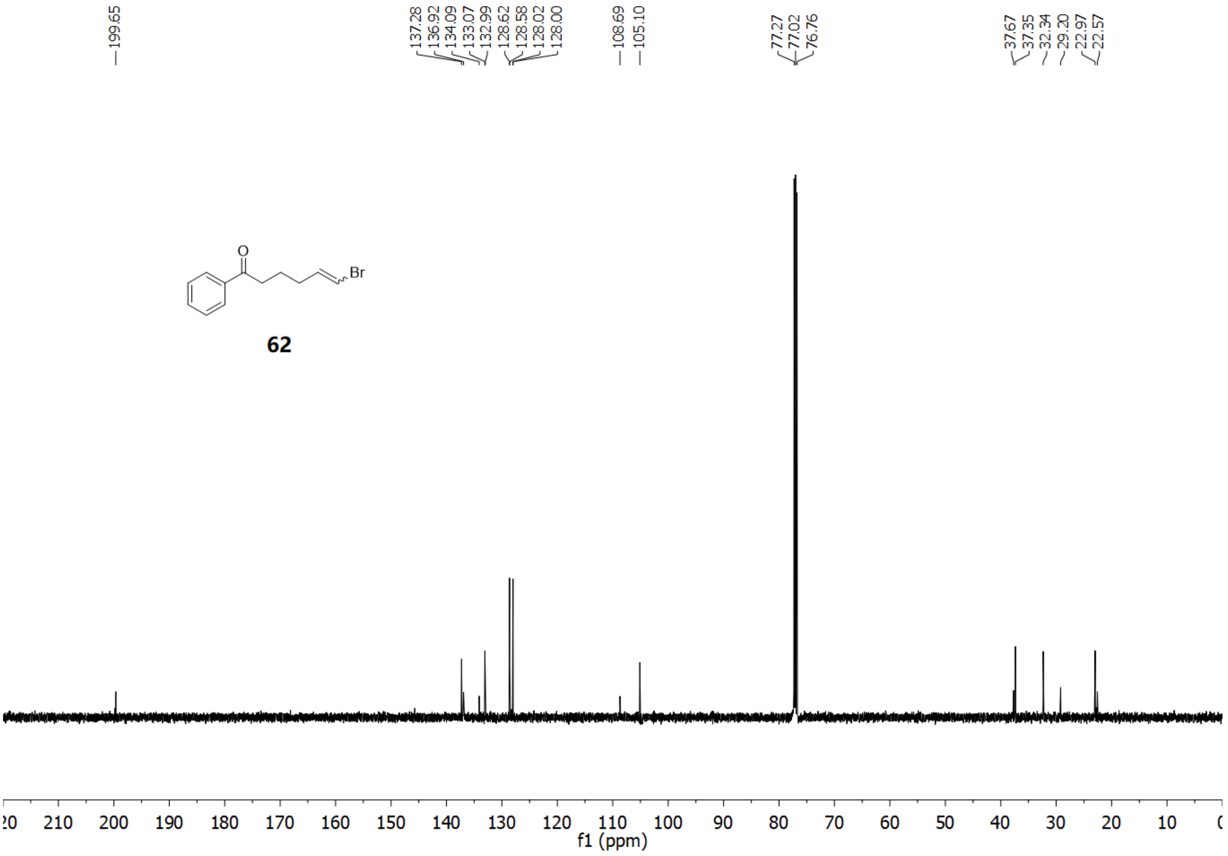

Supplementary Figure 61. ^{1}H and ^{13}C NMR spectra for compound **62**

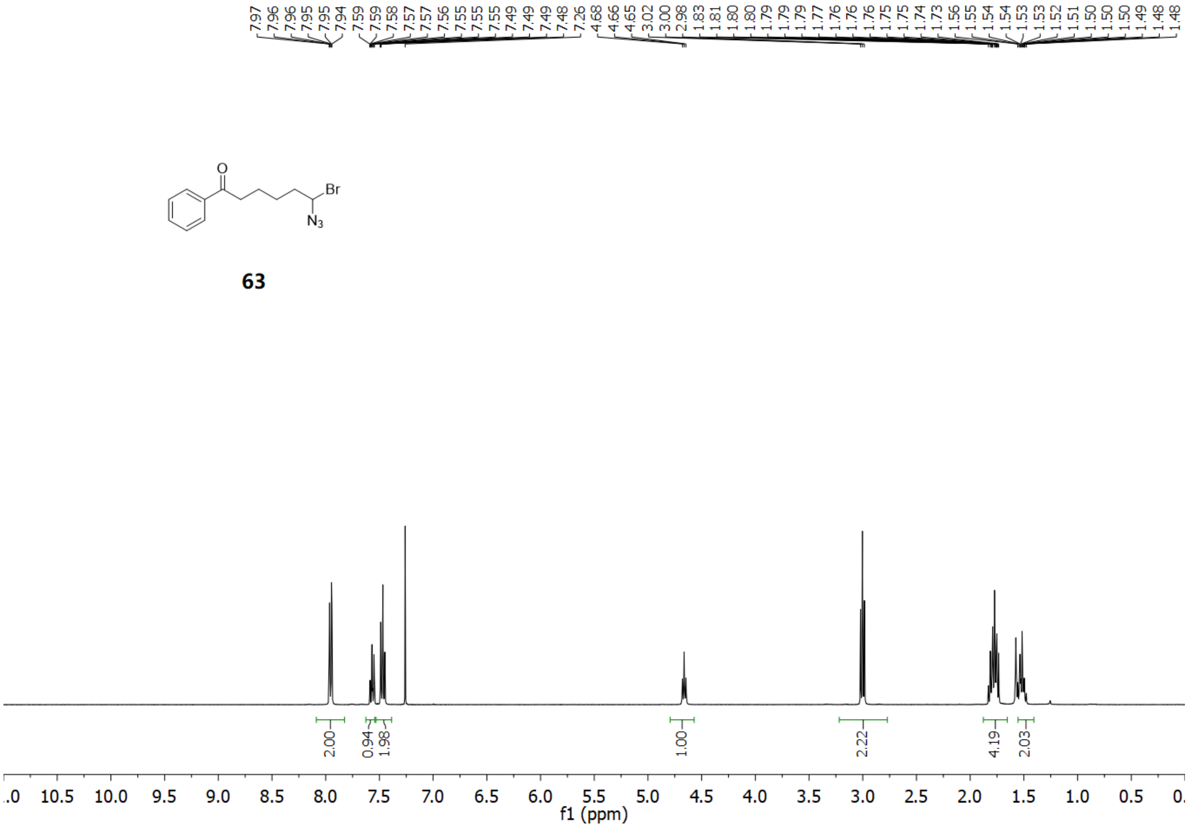

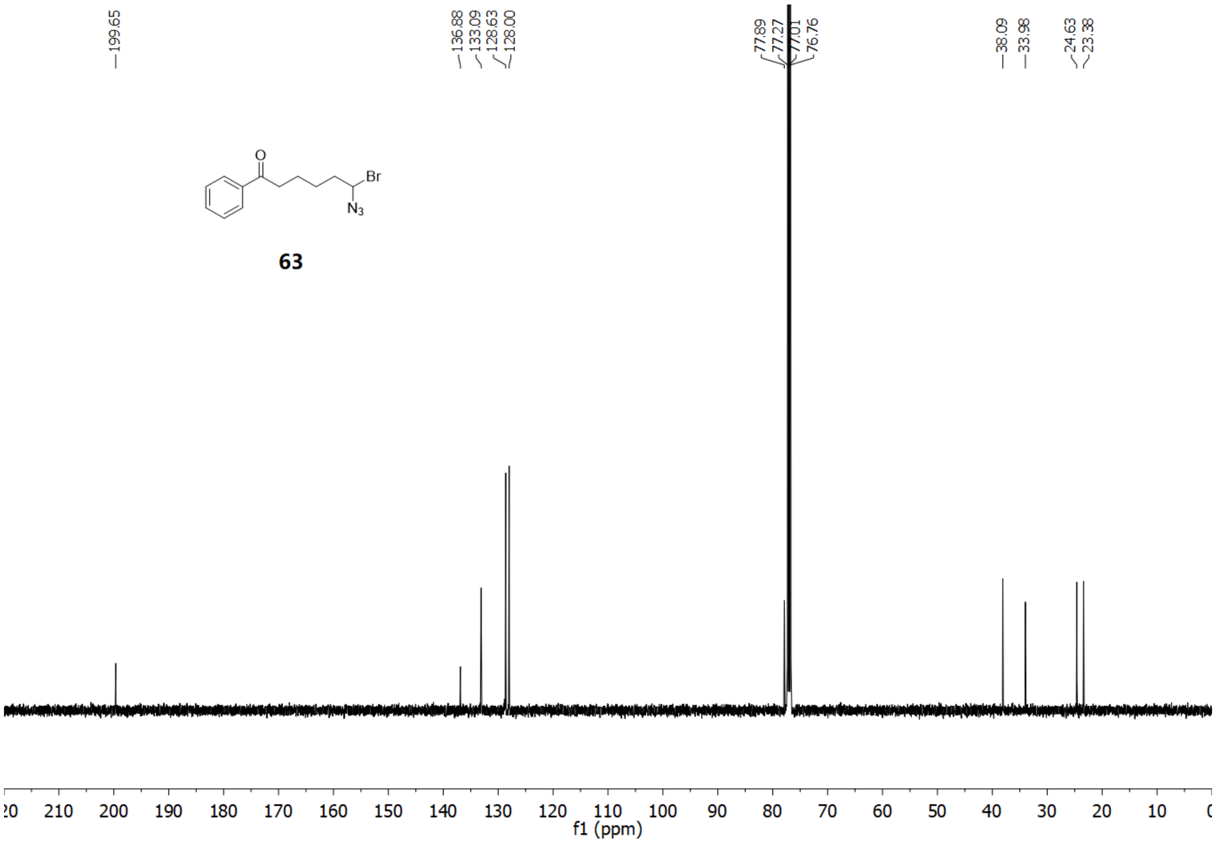

Supplementary Figure 62. ^{1}H and ^{13}C NMR spectra for compound **63**

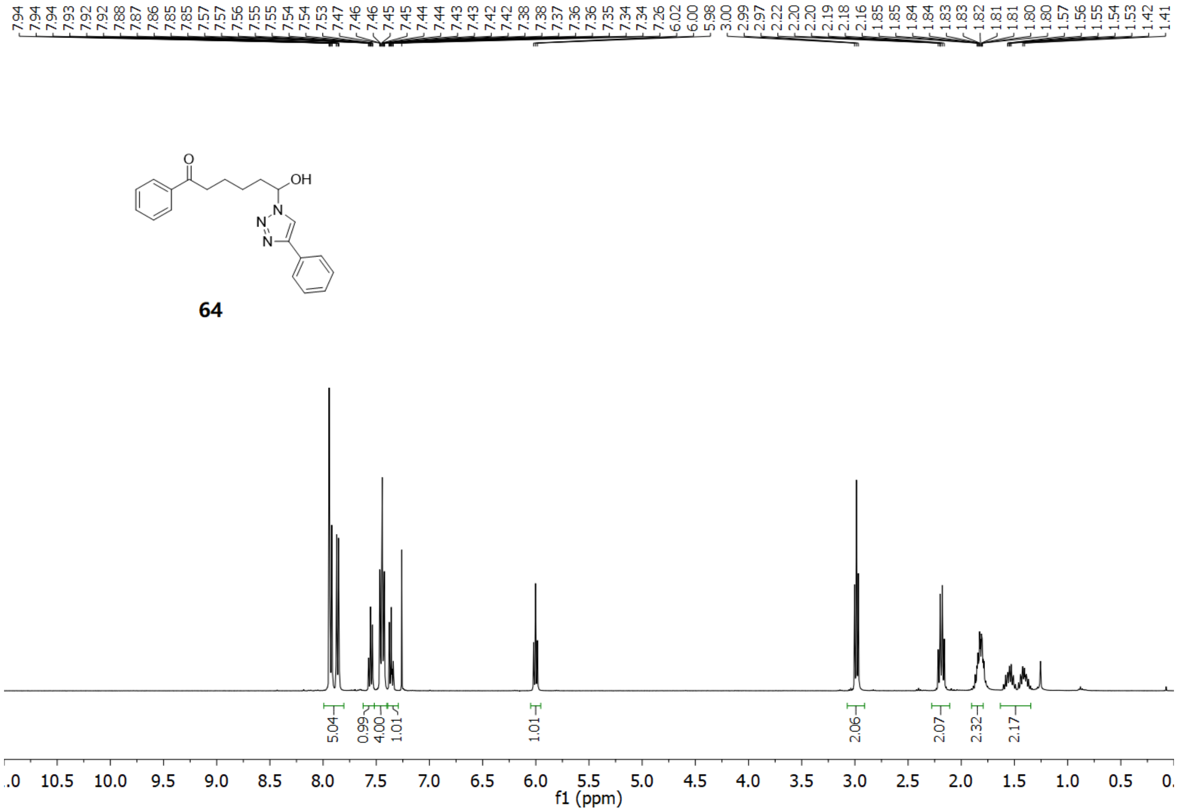

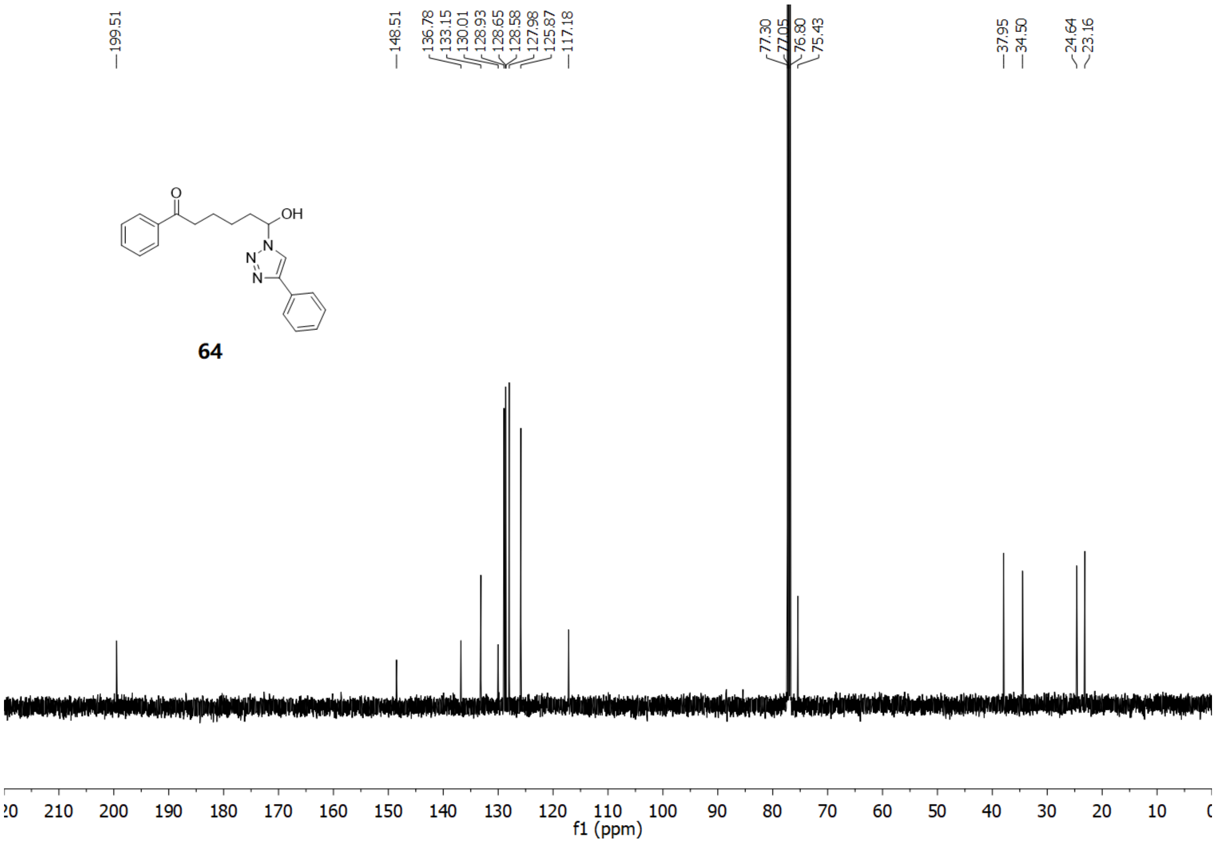

Supplementary Figure 63. ^{1}H and ^{13}C NMR spectra for compound **64**

Supplementary Note 2

Density functional theory (DFT) calculations were performed for the verification of the mechanism. The geometries optimization in this study (except **56** and **56***) was performed at the (u)B3LYP- D3(BJ) level of theory. The 6-311+g(d,p) basis set was used for all H, C, N, and O atoms, and the Stuttgart–Dresden basis set (SDD) was employed for Br and I atoms. The nature of the stationary points (minima with no imaginary frequency or transition states with one imaginary frequency) was confirmed. The free energies of the optimized geometries were calculated at the same level of theory, taking into account the solvent effect of acetonitrile using Solvent Polarizable Continuum Model (PCM). Unless specified otherwise, the Gibbs free energy was used throughout. Considering the deviation in the free energies is ~1.89 kcal/mol from the standard state (1 atm) to 1 M in solution, we reduced by 1.89 kcal/mol to the free energy for additional steps and added by 1.89 kcal/mol for the dissociation steps.[7] For transition state, intrinsic reaction coordinate (IRC) calculations were performed to verify whether it connected with correct reactants and products or intermediates. Time-dependent density functional theory (TD-DFT) was performed to calculate the vertical excitation energies of the photodissociation process, using the CAM-B3LYP-D3(BJ)[8] level of theory with the same basis set. All calculations were performed using the Gaussian 16 Rev. A.03 software suite.[9] The geometries were realized using CYLview, 1.0.[10]

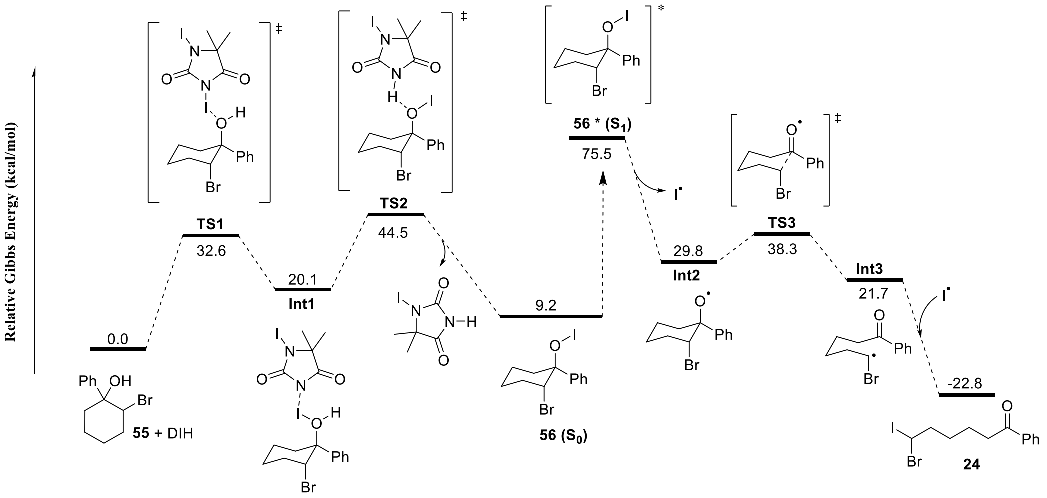

Supplementary Figure 64. Computational investigation of the ring-opening iodination process of **55** with DIH.

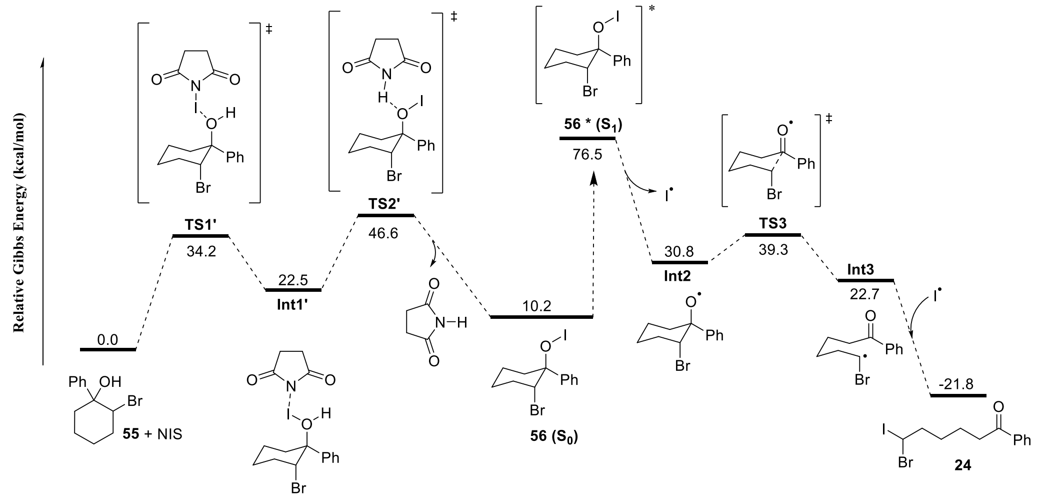

Supplementary Figure 65. Computational investigation of the ring-opening iodination process of **55** with NIS.

Calculated Cartesian Coordinates:

55

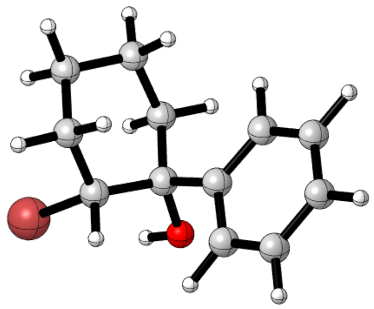

Sum of electronic and zero-point Energies= -554.903907
Sum of electronic and thermal Energies= -554.891291
Sum of electronic and thermal Enthalpies= -554.890347
Sum of electronic and thermal Free Energies= -554.943324

———

C	0.77292393	0.82858384	-1.58037006
C	1.20006386	2.14729017	-0.93352000
C	0.34444248	2.45830676	0.29710940
C	0.30024140	1.28266678	1.28681549
C	-0.08950607	-0.06986435	0.66467884
C	0.75557390	-0.33062739	-0.60375791
H	0.73719707	3.33792561	0.81537621
H	2.25516742	2.08725909	-0.65022614
H	1.11233343	2.95448738	-1.66559086
H	-0.25384790	0.92133248	-1.95665466
H	1.39978506	0.57990800	-2.43872581
H	1.29549954	1.15493226	1.72285750
H	-0.37462568	1.49541574	2.11842383
H	0.48449727	-1.26809115	-1.07480596
H	-0.66553601	2.71450935	-0.03225077
C	-1.56745333	-0.18588763	0.25871707
C	-2.01338287	-1.37180267	-0.34154865
C	-2.50331806	0.81938130	0.51257221
C	-3.34815894	-1.53703637	-0.69964462
H	-1.31945732	-2.18359358	-0.51919848
C	-3.84272009	0.65634296	0.15498283
H	-2.20834369	1.73770147	0.99927617
C	-4.27000179	-0.51811095	-0.45834005
H	-3.66847155	-2.46344633	-1.16305773
H	-4.54999322	1.45154568	0.36212336
H	-5.30984077	-0.64304926	-0.73841451
O	0.08931627	-1.11901570	1.63108114
H	1.03767713	-1.19643254	1.80613808
Br	2.71089150	-0.76582095	-0.05657390

DIH

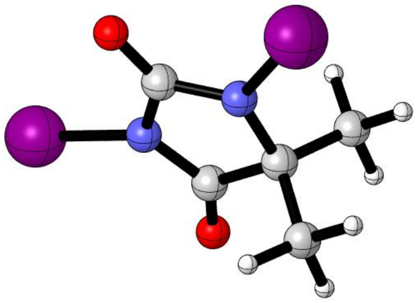

Sum of electronic and zero-point Energies= -476.997445

Sum of electronic and thermal Energies= -476.985480
Sum of electronic and thermal Enthalpies= -476.984536
Sum of electronic and thermal Free Energies= -477.038902

C	-0.73480488	1.54696234	-0.00016037
C	0.80844659	1.50539722	-0.00027957
C	0.04311602	-0.69847292	0.00013156
N	-1.04921568	0.10629220	0.00007668
N	1.16470495	0.18244766	-0.00009450
O	0.11166174	-1.90913181	0.00032483
I	3.13420517	-0.49940946	-0.00015533
I	-2.97970253	-0.66720933	0.00029333
O	1.54573811	2.46555510	-0.00049263
C	-1.23294192	2.23366870	-1.27149536
H	-2.32486725	2.22158373	-1.28598700
H	-0.89574135	3.27112000	-1.28792888
H	-0.86116642	1.72096731	-2.16094962
C	-1.23273189	2.23403335	1.27106049
H	-2.32465462	2.22193392	1.28574638
H	-0.86079230	1.72159859	2.16059990
H	-0.89554535	3.27149516	1.28713217

TS1

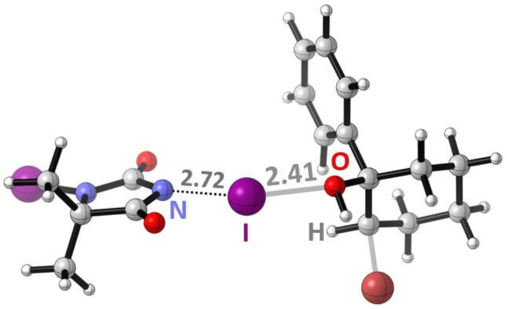

(Bond lengths are in Å)

Sum of electronic and zero-point Energies= -1031.865790
Sum of electronic and thermal Energies= -1031.839897
Sum of electronic and thermal Enthalpies= -1031.838953
Sum of electronic and thermal Free Energies= -1031.927204

C	4.58383210	0.37776000	-2.14966014
C	6.02924020	0.49603492	-1.66281538
C	6.09114235	1.15513215	-0.28370570
C	5.21983302	0.40460545	0.73011991
C	3.75396468	0.34234278	0.27078746
C	3.65448732	-0.27207681	-1.13677118
H	7.12018657	1.17366462	0.08387228
H	6.48752490	-0.49679467	-1.61662109
H	6.60208415	1.07822649	-2.38892215
H	4.18894553	1.38185937	-2.33565693
H	4.51934048	-0.16092859	-3.09653701
H	5.59380739	-0.61709970	0.84060487
H	5.27294981	0.87214121	1.71207210
H	2.62643551	-0.32289398	-1.47869230
H	5.75504129	2.19499934	-0.35353254

C	3.04523966	1.69547920	0.36543791
C	2.25949744	2.22605923	-0.66169123
C	3.12376097	2.39622521	1.57636218
C	1.58728283	3.43540872	-0.49165903
H	2.14116710	1.70694879	-1.60175674
C	2.44827650	3.59984798	1.74905031
H	3.69891102	1.99621548	2.40093363
C	1.68016054	4.12819580	0.71187093
H	0.98289985	3.82617857	-1.30196010
H	2.51981661	4.12241862	2.69597273
H	1.15416600	5.06664770	0.84463696
O	3.03275228	-0.49443516	1.25342660
Br	4.09846203	-2.28339502	-1.02789939
H	3.41730335	-1.38961181	1.23802339
C	-2.35663868	-1.14227703	0.86037276
C	-2.88107956	0.58039368	-0.47362665
N	-1.85912006	-0.12540373	0.18716415
O	-2.73456329	1.57006479	-1.17137636
I	0.64585342	-0.74998942	1.03066545
O	-1.64684846	-1.91738839	1.53262458
C	-3.88096990	-1.22896460	0.70528775
N	-4.06355034	-0.06033296	-0.18152003
I	-5.90772502	0.58679105	-0.88833445
C	-4.28481191	-2.53425062	0.02038303
H	-5.36171035	-2.53185957	-0.16200778
H	-3.76563405	-2.64808214	-0.93341488
H	-4.04254788	-3.38282220	0.66258698
C	-4.57177324	-1.03918893	2.05556099
H	-4.32671394	-1.87076875	2.71858250
H	-4.25744708	-0.10421025	2.52381476
H	-5.65419996	-1.01602502	1.91220582

Int1

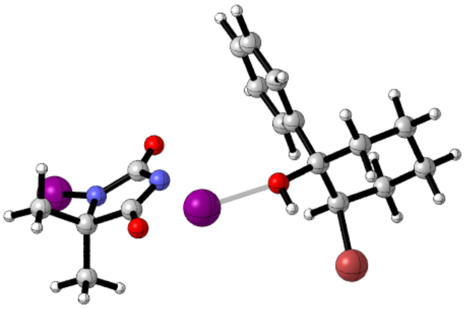

Sum of electronic and zero-point Energies= -1031.884798
Sum of electronic and thermal Energies= -1031.858350
Sum of electronic and thermal Enthalpies= -1031.857405
Sum of electronic and thermal Free Energies= -1031.947230

C	4.44653376	0.42876684	-2.24017320
C	5.91023660	0.66138707	-1.86088052
C	6.02035050	1.35379298	-0.50082574
C	5.27823209	0.56593562	0.58455243
C	3.79248102	0.38241129	0.23671328
C	3.64169461	-0.26429668	-1.15267240

H	7.06881563	1.45596315	-0.20952621
H	6.44178731	-0.29494559	-1.83035200
H	6.38674023	1.26660971	-2.63621438
H	3.96709080	1.39796995	-2.41330353
H	4.35487498	-0.13272455	-3.17146104
H	5.73350500	-0.42335683	0.68798046
H	5.36499072	1.05857856	1.55222139
H	2.59907742	-0.39702089	-1.42015713
H	5.60480994	2.36509163	-0.56560724
C	2.99449979	1.68639476	0.35204067
C	2.09931991	2.13171844	-0.62389697
C	3.10560994	2.42099138	1.53976192
C	1.34885279	3.29038164	-0.42758703
H	1.95389378	1.58262091	-1.54298620
C	2.35422490	3.57472497	1.73920615
H	3.76874545	2.08548047	2.32622015
C	1.47400275	4.01773493	0.75226193
H	0.65871687	3.61352886	-1.19826433
H	2.45401835	4.12438230	2.66816988
H	0.88751537	4.91632892	0.90596031
O	3.19620986	-0.47600356	1.27156889
Br	4.23858626	-2.23715317	-1.04690598
H	3.65365874	-1.33415376	1.25871610
C	-2.17642399	-0.66878914	0.59174446
C	-3.09774129	0.77367346	-0.78214088
N	-1.88592941	0.30600946	-0.21258611
O	-3.18575785	1.68765362	-1.58486975
I	0.76780504	-0.89133214	1.22445020
O	-1.35730143	-1.36492946	1.30780190
C	-3.66593751	-1.00144691	0.67594625
N	-4.13051918	0.02405362	-0.27423415
I	-6.11758730	0.31337141	-0.78411606
C	-3.94424710	-2.41954863	0.17480026
H	-5.02197319	-2.59352772	0.14906915
H	-3.53924648	-2.56133242	-0.82927675
H	-3.49087874	-3.14824877	0.84886077
C	-4.20547305	-0.76602932	2.08742138
H	-3.75380696	-1.47649769	2.78176644
H	-3.98432283	0.24963358	2.42186110
H	-5.28743009	-0.91312228	2.09442462

TS2

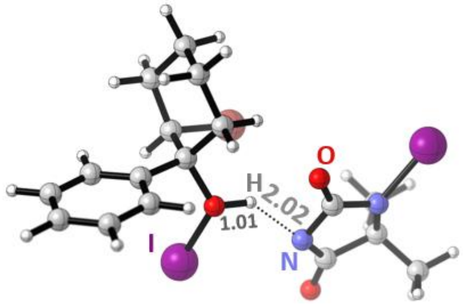

(Bond lengths are in Å)

Sum of electronic and zero-point Energies= -1031.848391

Sum of electronic and thermal Energies= -1031.822788
Sum of electronic and thermal Enthalpies= -1031.821843
Sum of electronic and thermal Free Energies= -1031.908264

C	-2.73565701	-2.44746192	1.74603974
C	-2.10512995	-3.60960040	0.97766835
C	-2.14931627	-3.35502699	-0.52912017
C	-1.47032442	-2.02482615	-0.88818245
C	-2.16781801	-0.86981744	-0.16174790
C	-2.16428046	-1.08646353	1.35648351
H	-1.63248395	-4.15365036	-1.06700790
H	-1.06763086	-3.74757101	1.29666305
H	-2.64115470	-4.52977423	1.22369129
H	-3.80810196	-2.42090302	1.53262082
H	-2.63521343	-2.57561496	2.82490038
H	-0.42448405	-2.04872187	-0.58831483
H	-1.49404028	-1.85399652	-1.96259650
H	-2.66046515	-0.27200488	1.87394353
H	-3.18709790	-3.34820021	-0.87748769
C	-3.54821805	-0.55272681	-0.72294482
C	-4.68281747	-0.38103178	0.07599730
C	-3.66718729	-0.36620674	-2.10791939
C	-5.91068222	-0.05664139	-0.49708809
H	-4.63246317	-0.48293962	1.15031946
C	-4.89061490	-0.03593721	-2.67856417
H	-2.79785180	-0.46036903	-2.74507067
C	-6.02060937	0.11397923	-1.87450472
H	-6.77831526	0.06951927	0.13975585
H	-4.96025388	0.10790934	-3.75042884
H	-6.97615939	0.36826318	-2.31850220
O	-1.36395036	0.37692794	-0.52572842
Br	-0.27169102	-0.93172521	2.09286558
I	-1.95058390	2.26853078	0.22308282
N	1.27559344	1.46853521	-0.38231296
C	2.21042148	2.26652196	0.18153538
C	1.87757183	0.30854509	-0.80487766
C	3.60210322	1.56566502	0.16113993
O	2.04683170	3.38609382	0.66425059
N	3.24211128	0.37446332	-0.62661270
O	1.24800363	-0.64246997	-1.28376820
C	4.02091964	1.22917094	1.59343112
C	4.65203688	2.40657044	-0.55700819
I	4.44608029	-1.32747244	-0.76080184
H	4.97958059	0.70500445	1.59919739
H	4.12862391	2.15208560	2.16672305
H	3.27407431	0.59884348	2.08197638
H	5.59705474	1.85931914	-0.61359719
H	4.32668520	2.64749195	-1.57173217
H	4.82448946	3.33684376	-0.01212236
H	-0.36031408	0.28907774	-0.53169677

1-iodo-5, 5-dimethylhydantoin

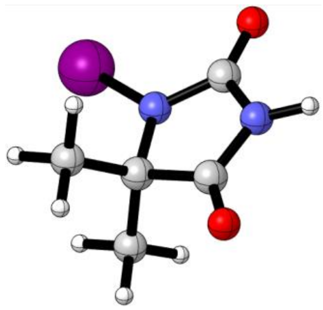

Sum of electronic and zero-point Energies= -466.201225
Sum of electronic and thermal Energies= -466.190939
Sum of electronic and thermal Enthalpies= -466.189995
Sum of electronic and thermal Free Energies= -466.238666

———

C	1.25777889	-0.76827846	0.00009403
C	2.51590644	0.12274714	0.00001197
C	0.68098658	1.53752707	0.00004469
N	0.20292765	0.26404581	0.00000775
N	2.08399091	1.41853922	0.00022766
O	0.07021633	2.58951770	0.00005291
I	-1.83331011	-0.14225731	-0.00003652
O	3.66619179	-0.26278691	-0.00012101
C	1.21742306	-1.61939458	1.26928961
H	0.29935250	-2.21044406	1.28444020
H	2.06959457	-2.30089984	1.28390935
H	1.24762179	-0.98975787	2.16107136
C	1.21770460	-1.61924410	-1.26923793
H	0.29917680	-2.20955920	-1.28523425
H	1.24915166	-0.98953265	-2.16093140
H	2.06943329	-2.30132049	-1.28316320
H	2.69261291	2.22906750	-0.00047376

56 / 56*

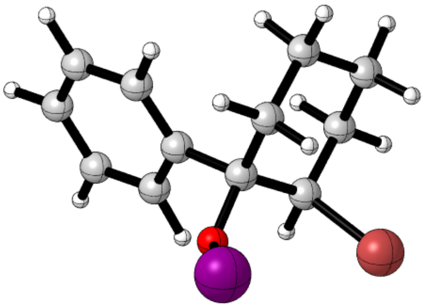

Sum of electronic and zero-point Energies= -565.684964
Sum of electronic and thermal Energies= -565.670691
Sum of electronic and thermal Enthalpies= -565.669747
Sum of electronic and thermal Free Energies= -565.728823

———

C	2.24404298	1.93086693	-0.05243737
C	2.61186724	1.74987817	1.41535092
C	1.39303201	1.33934140	2.23507609
C	0.66308795	0.13307009	1.63299321
C	0.32141916	0.27665299	0.14759180
C	1.57300442	0.70540175	-0.64406476
H	1.69326588	1.08842355	3.25540165

H	3.39491674	0.99234659	1.50361486
H	3.02708694	2.68272484	1.80357447
H	1.53340184	2.75864032	-0.15360153
H	3.11702864	2.18905173	-0.65354520
H	1.29896144	-0.75090790	1.71628422
H	-0.24548980	-0.07874203	2.19880218
H	1.32755135	0.84688598	-1.68945286
H	0.71746074	2.19361726	2.31122242
C	-0.80139932	1.27004619	-0.16648766
C	-1.18933435	1.43887881	-1.49774211
C	-1.51264584	1.94777905	0.81860554
C	-2.24021445	2.27680842	-1.83535533
H	-0.68174353	0.88967055	-2.28059085
C	-2.56792660	2.78926498	0.48354230
H	-1.26863706	1.81777083	1.86290879
C	-2.93191338	2.96253209	-0.84320000
H	-2.52447120	2.38951448	-2.87506250
H	-3.10898205	3.30435305	1.26865990
H	-3.75441510	3.61823163	-1.10385005
O	-0.02887492	-0.98989722	-0.42976592
Br	2.92519551	-0.79735473	-0.75227979
I	-1.73688486	-1.90637949	0.23472300

Int2

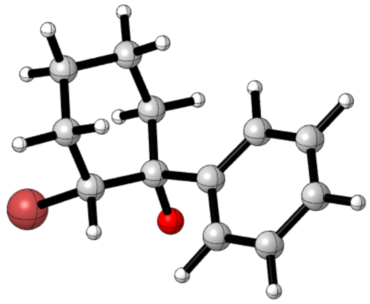

Sum of electronic and zero-point Energies= -554.243284
Sum of electronic and thermal Energies= -554.230835
Sum of electronic and thermal Enthalpies= -554.229890
Sum of electronic and thermal Free Energies= -554.283714

C	-0.86948025	0.75110977	1.62661387
C	-1.24531691	2.10574413	1.02338899
C	-0.33576739	2.45322613	-0.15832059
C	-0.31417408	1.33318283	-1.20735624
C	0.06288780	-0.04329101	-0.62501054
C	-0.81527234	-0.36632854	0.60216465
H	-0.67211716	3.37855248	-0.63506678
H	-2.28831340	2.08007340	0.69284338
H	-1.17373178	2.87738070	1.79465897
H	0.13885629	0.81224343	2.05692480
H	-1.54158613	0.47408509	2.44145683
H	-1.31175909	1.22266926	-1.63955255
H	0.35747537	1.57069017	-2.03318164
H	-0.54958469	-1.31784237	1.04623366
H	0.67715712	2.63939118	0.21164959

C	1.56353667	-0.18267766	-0.24646790
C	1.99391777	-1.33009502	0.43998781
C	2.51577362	0.76587971	-0.64348885
C	3.33409788	-1.49141587	0.77719526
H	1.28785945	-2.10196581	0.71349858
C	3.85376144	0.60316180	-0.29983151
H	2.22227313	1.64110025	-1.20445735
C	4.26797223	-0.52246848	0.41409874
H	3.64774038	-2.37759105	1.31651069
H	4.57509103	1.35705920	-0.59317793
H	5.31250026	-0.64659923	0.67575690
O	0.03015137	-1.03418047	-1.58456366
Br	-2.71413401	-0.79405628	-0.06815514

TS3

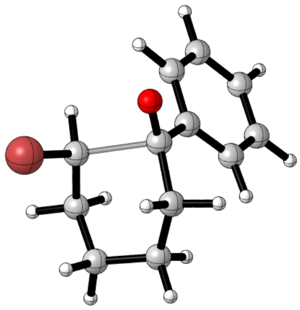

Sum of electronic and zero-point Energies= -554.229657
Sum of electronic and thermal Energies= -554.217187
Sum of electronic and thermal Enthalpies= -554.216243
Sum of electronic and thermal Free Energies= -554.270173

C	0.74743582	0.76530236	-1.55337291
C	1.18939423	2.09938323	-0.95028493
C	0.33745686	2.47025419	0.26265963
C	0.28900133	1.38290847	1.34387618
C	-0.21459889	-0.02886254	0.96996257
C	0.88216118	-0.40133507	-0.62734898
H	0.74076243	3.37670860	0.72463869
H	2.24548470	2.04212347	-0.67078403
H	1.10427122	2.87700435	-1.71332990
H	-0.30382639	0.81614967	-1.85610478
H	1.32581139	0.53833263	-2.45836997
H	1.28447305	1.23639756	1.76853929
H	-0.35548499	1.71593659	2.16535837
H	0.44278594	-1.34012179	-0.93517668
H	-0.66857320	2.72063755	-0.07884819
C	-1.61247142	-0.18130751	0.38386966
C	-2.02340682	-1.44235287	-0.06975295
C	-2.53902971	0.86594346	0.37439916
C	-3.31209485	-1.64352578	-0.55198165
H	-1.33051003	-2.27459011	-0.03227381
C	-3.83696905	0.66278625	-0.09677243
H	-2.27101481	1.84073062	0.75586510
C	-4.22494300	-0.58703925	-0.57252287
H	-3.60739143	-2.62475785	-0.90639467

H	-4.54334276	1.48532887	-0.08688597
H	-5.23068245	-0.74143484	-0.94662361
O	0.16268616	-0.97906637	1.73617000
Br	2.73640383	-0.78882411	-0.08843865

Int3

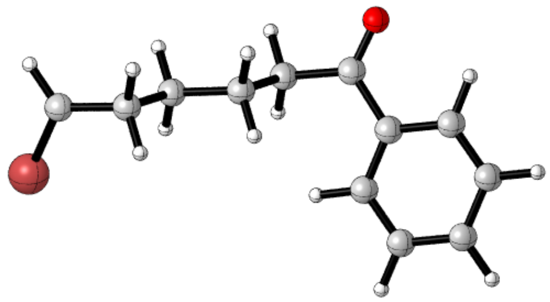

Sum of electronic and zero-point Energies= -554.249952
Sum of electronic and thermal Energies= -554.235490
Sum of electronic and thermal Enthalpies= -554.234546
Sum of electronic and thermal Free Energies= -554.296686

C	2.47731311	1.46055954	-0.44694202
C	1.02571906	1.39356811	-0.86793360
C	0.10455962	1.08490506	0.32780078
H	0.76487946	2.36554786	-1.29130058
H	0.88354663	0.63675696	-1.64326509
H	0.38576153	0.12811110	0.77791151
H	0.24695864	1.85205779	1.09655507
C	-1.36570387	1.03937179	-0.08722923
C	-2.29373559	0.74135702	1.11197892
H	-1.50648606	0.26845801	-0.85172685
H	-1.65585060	1.99464004	-0.53690351
C	-3.73373011	0.73764889	0.77539518
H	-2.14910213	1.51957416	1.87184283
H	-2.00240779	-0.21252897	1.56471286
H	-4.31227753	1.63634670	0.60444634
Br	-4.46135465	-0.77408476	-0.19517318
O	3.02124649	2.54343735	-0.27177451
C	3.22921492	0.18952824	-0.20048956
C	4.50883429	0.26466545	0.36971229
C	2.69876629	-1.06908002	-0.51637257
C	5.24046969	-0.88963245	0.62023203
H	4.91560902	1.23798198	0.61417784
C	3.43397138	-2.22578225	-0.26794147
H	1.71568179	-1.15632700	-0.95979347
C	4.70359191	-2.13900186	0.30121744
H	6.22697554	-0.81980689	1.06399420
H	3.01513696	-3.19353353	-0.51843307
H	5.27339114	-3.04045553	0.49647025

I·

Sum of electronic and zero-point Energies= -11.397841
Sum of electronic and thermal Energies= -11.396425
Sum of electronic and thermal Enthalpies= -11.395481

Sum of electronic and thermal Free Energies= -11.415344

24

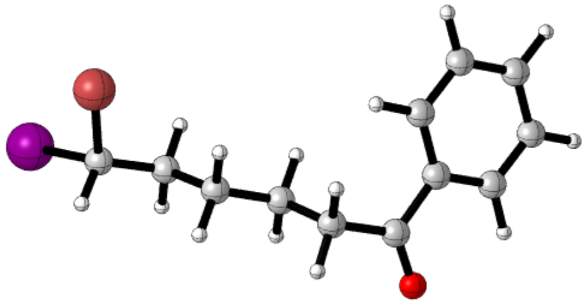

Sum of electronic and zero-point Energies= -565.728810
Sum of electronic and thermal Energies= -565.712914
Sum of electronic and thermal Enthalpies= -565.711970
Sum of electronic and thermal Free Energies= -565.779913

C	3.95838244	-0.63528619	1.39227736
C	2.61610426	-0.00943110	1.70104590
C	1.50516052	-0.57028602	0.79331290
H	2.38553630	-0.23216038	2.74471775
H	2.66570419	1.07655081	1.58844321
H	1.75088923	-0.38163916	-0.25579235
H	1.45442778	-1.65707492	0.91757841
C	0.14538590	0.05137771	1.11624174
C	-0.94946876	-0.48507701	0.18839300
H	0.21517376	1.13833395	1.02220021
H	-0.12007578	-0.16439126	2.15705901
H	-0.97038823	-1.57813942	0.25705679
H	-0.73152339	-0.23279245	-0.85254934
Br	-2.52996033	1.98417536	0.38351701
O	4.40338404	-1.52827300	2.10190742
C	4.71822501	-0.17925804	0.18577624
C	5.88317500	-0.87125990	-0.17754847
C	4.30539050	0.91237607	-0.59103639
C	6.61880181	-0.48222416	-1.29014357
H	6.19822904	-1.71519049	0.42331634
C	5.04441534	1.30319970	-1.70511553
H	3.41189066	1.46515134	-0.33228942
C	6.20029712	0.60770483	-2.05690461
H	7.51645730	-1.02528647	-1.56280025
H	4.71799251	2.15025064	-2.29726244
H	6.77368474	0.91233678	-2.92512506
C	-2.33398430	-0.01486759	0.55956327
H	-2.60418420	-0.22145741	1.58851553
I	-3.87950159	-1.02860859	-0.63031310

NIS

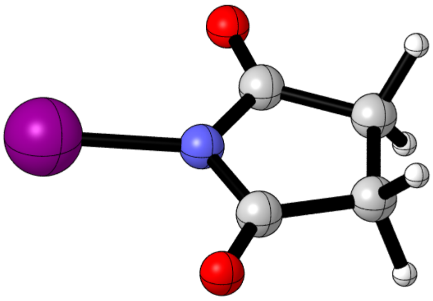

Sum of electronic and zero-point Energies= -371.522976
Sum of electronic and thermal Energies= -371.515473
Sum of electronic and thermal Enthalpies= -371.514528
Sum of electronic and thermal Free Energies= -371.557606

——

C	2.78650502	0.76652989	0.00009833
C	2.78633116	-0.76691502	-0.00014156
C	1.32684920	-1.17397034	0.00003931
C	1.32710266	1.17396812	-0.00008518
H	3.26314043	1.19657017	0.88277991
H	3.26363997	1.19695160	-0.88211388
H	3.26326477	-1.19738428	0.88216465
H	3.26295352	-1.19714273	-0.88273145
N	0.57035875	0.00006500	-0.00005238
I	-1.51494802	0.00004417	-0.00000351
O	0.86762186	-2.29314350	0.00009317
O	0.86812898	2.29321015	0.00003033

TS1'

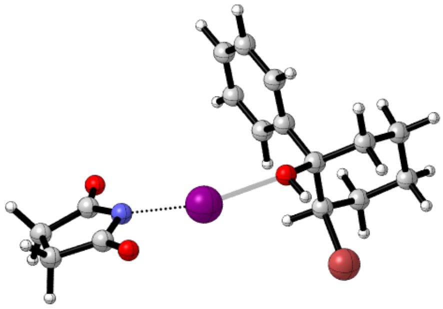

Sum of electronic and zero-point Energies= -926.388188
Sum of electronic and thermal Energies= -926.366695
Sum of electronic and thermal Enthalpies= -926.365751
Sum of electronic and thermal Free Energies= -926.443447

——

C	-3.21784092	-0.06858964	1.87752384
C	-4.60227598	0.05587756	1.23863312
C	-4.54665966	0.91425465	-0.02656653
C	-3.51871176	0.36701332	-1.02368257
C	-2.11400068	0.30088333	-0.40221862
C	-2.13876614	-0.51336826	0.90323960
H	-5.52593078	0.94258321	-0.51102981
H	-4.98735573	-0.93844953	0.99168085
H	-5.29117492	0.49330424	1.96557404
H	-2.91277294	0.91260513	2.25565793
H	-3.22867744	-0.74141372	2.73669291
H	-3.81226556	-0.64179185	-1.32756359

H	-3.48719280	0.97586716	-1.92619765
H	-1.15576909	-0.56575921	1.35851691
H	-4.28709250	1.94542886	0.23538307
C	-1.48721114	1.68369158	-0.20745932
C	-0.85939833	2.08457449	0.97511763
C	-1.46890328	2.55722250	-1.30268898
C	-0.24675138	3.33342900	1.06782793
H	-0.81992797	1.43372333	1.83649181
C	-0.85267212	3.80110808	-1.21336015
H	-1.92086852	2.26233577	-2.24044440
C	-0.24210567	4.19715297	-0.02364466
H	0.23531462	3.62158561	1.99478685
H	-0.84675778	4.45853331	-2.07518026
H	0.23780531	5.16643583	0.04822753
O	-1.23178300	-0.33940673	-1.39738670
Br	-2.43488829	-2.50330149	0.45241219
H	-1.55668122	-1.24282240	-1.56178209
C	4.12378954	-0.75060565	-0.41117561
C	4.23416600	0.34328078	1.54288487
N	3.42032421	-0.10796972	0.51669152
O	3.85547100	0.96291757	2.52213914
I	1.14247639	-0.51734243	-0.90842842
O	3.57021136	-1.23324523	-1.42193324
C	5.68340795	-0.06090588	1.25087305
H	6.30361522	0.83665249	1.20759284
H	6.05351827	-0.68133912	2.06958392
C	5.60243298	-0.80908237	-0.08909266
H	6.16744093	-0.33289922	-0.89273620
H	5.92015349	-1.85183463	-0.02963503

Int1’

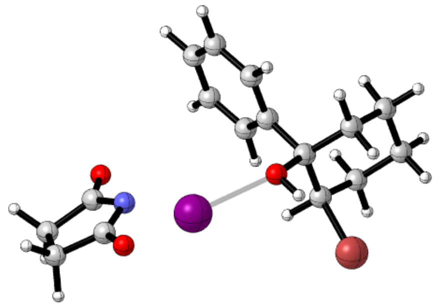

Sum of electronic and zero-point Energies= -926.406015
Sum of electronic and thermal Energies= -926.384044
Sum of electronic and thermal Enthalpies= -926.383100
Sum of electronic and thermal Free Energies= -926.462071

———

C	-3.08601844	0.38044062	1.97059109
C	-4.47083998	0.70948263	1.40973953
C	-4.36022227	1.46819285	0.08557870
C	-3.51505464	0.69074397	-0.92941023
C	-2.10130708	0.41230635	-0.39477982
C	-2.17215237	-0.30326031	0.96646838
H	-5.35290818	1.64032957	-0.33818946
H	-5.03780678	-0.21425469	1.25713186

H	-5.02020292	1.30323987	2.14469613
H	-2.59013264	1.31287716	2.25936538
H	-3.15097534	-0.22833323	2.87406188
H	-3.99823403	-0.26690761	-1.14391905
H	-3.44523247	1.23025010	-1.87317212
H	-1.18238141	-0.50200900	1.36342275
H	-3.90964306	2.45142800	0.25830330
C	-1.23696072	1.67662649	-0.32979537
C	-0.47558224	2.03318217	0.78603293
C	-1.13761437	2.46377671	-1.48439219
C	0.34974087	3.15660522	0.75633312
H	-0.49256351	1.43924627	1.68843145
C	-0.31116187	3.58240249	-1.51722925
H	-1.69104958	2.19492362	-2.37445604
C	0.43406311	3.93696084	-0.39291680
H	0.93396112	3.41027136	1.63326516
H	-0.24681719	4.17371412	-2.42349105
H	1.07900272	4.80794635	-0.41657397
O	-1.40762513	-0.42698129	-1.38176606
Br	-2.83942790	-2.23471685	0.67913040
H	-1.88924934	-1.26793941	-1.46229715
C	3.85028925	-0.80441424	-0.05962358
C	4.55364627	0.53451716	1.52543151
N	3.44741879	0.11006337	0.78030578
O	4.49780900	1.38160880	2.39802702
I	0.99693466	-0.91298101	-1.06128372
O	3.10909069	-1.42408570	-0.91818008
C	5.81992372	-0.20432229	1.08951883
H	6.55351420	0.52033393	0.73077896
H	6.25241494	-0.72223573	1.94747366
C	5.32310799	-1.15120119	-0.00608977
H	5.77876267	-0.97783238	-0.98284646
H	5.44270903	-2.20897969	0.23695825

TS2'

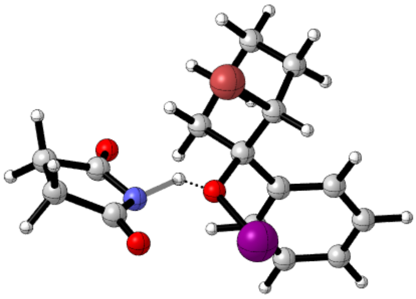

Sum of electronic and zero-point Energies= -926.369206
Sum of electronic and thermal Energies= -926.347924
Sum of electronic and thermal Enthalpies= -926.346980
Sum of electronic and thermal Free Energies= -926.423618

———

C	-2.10083862	2.49239669	-1.11641421
C	-1.96245096	3.48044885	0.04245487
C	-2.03569261	2.75725629	1.38752772
C	-0.97941696	1.64616458	1.48006990

C	-1.18920890	0.63009562	0.35148914
C	-1.13724091	1.31232868	-1.02133296
H	-1.86229307	3.45805531	2.20802124
H	-1.01087990	4.01475825	-0.03630746
H	-2.75662925	4.22773185	-0.03148141
H	-3.11523386	2.08292750	-1.11457045
H	-1.96367431	2.98212953	-2.08169468
H	0.01824493	2.07108645	1.38970658
H	-1.02694597	1.14311607	2.44333617
H	-1.28570327	0.60045644	-1.82628972
H	-3.03458625	2.33252272	1.52999842
C	-2.43077745	-0.23246678	0.54709410
C	-3.35895532	-0.48211516	-0.46844068
C	-2.61173110	-0.85598791	1.79003578
C	-4.45409160	-1.31275571	-0.23988643
H	-3.24113317	-0.05279072	-1.45280951
C	-3.70047653	-1.69001951	2.01579500
H	-1.89158739	-0.70681493	2.58349114
C	-4.63125363	-1.91683954	1.00188631
H	-5.16257854	-1.49147285	-1.04015924
H	-3.81985379	-2.16494146	2.98259819
H	-5.48243939	-2.56451117	1.17755490
O	-0.05152536	-0.37435612	0.49330764
Br	0.75009710	1.96937986	-1.41485398
I	0.08354778	-2.02550997	-0.82639930
N	2.77840960	-0.53762397	0.56961573
C	4.00776010	-0.94668961	0.12953754
C	2.92125089	0.39226603	1.53899713
O	4.21382814	-1.77881447	-0.75337217
O	1.94697103	0.93722254	2.08165331
H	0.86969397	-0.01456925	0.70927564
C	5.13063139	-0.22509131	0.89035718
H	5.75591989	0.31654115	0.17675327
H	5.76205374	-0.96585564	1.38628072
C	4.38102985	0.68878624	1.86038731
H	4.57744595	1.75228221	1.70527168
H	4.57446518	0.46736926	2.91271009

Succinimide

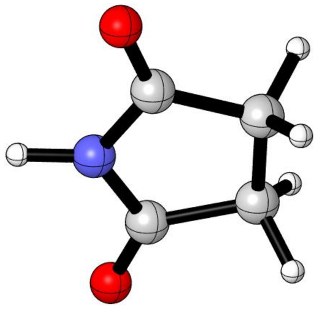

Sum of electronic and zero-point Energies= -360.725424
Sum of electronic and thermal Energies= -360.719482
Sum of electronic and thermal Enthalpies= -360.718538
Sum of electronic and thermal Free Energies= -360.755766

——

C	-0.76734790	1.25523364	0.00015143
C	0.76734805	1.25523376	-0.00015045
C	1.16539373	-0.20836713	-0.00000188
C	-1.16539349	-0.20836719	0.00000417
H	-1.19768324	1.73373324	0.88178853
H	-1.19809695	1.73411699	-0.88106579
H	1.19809611	1.73411768	0.88106709
H	1.19768395	1.73373309	-0.88178732
N	-0.00000024	-0.95803218	0.00001007
O	2.28522930	-0.67618738	0.00006051
O	-2.28522935	-0.67618713	-0.00007256
H	-0.00000017	-1.97287823	0.00000370

Supplementary References

1. Barluenga, J., Marco-Arias, M., González-Bobes, F., Ballesteros, A. & González, J. M. Reaction of alkenes with hydrogen peroxide and sodium iodide: a nonenzymatic biogenic-like approach to iodohydrins. *Chem. - A Eur. J.* **10**, 1677–1682 (2004).
2. Li, J., Li, Z., Zhang, X., Xu, B. & Shi, Y. Catalytic enantioselective bromohydroxylation of aryl olefins with flexible functionalities. *Org. Chem. Front.* **4**, 1084–1090 (2017).
3. Beebe, T. R. *et al.* Oxidation of alcohols with acetyl hypoiodite. *J. Org. Chem.* **40**, 1992–1994 (1975).
4. Zhang, Z.-Q. *et al.* Copper-catalyzed/promoted cross-coupling of gem-diborylalkanes with nonactivated primary alkyl halides: an alternative route to alkylboronic esters. *Org. Lett.* **16**, 6342–6345 (2014).
5. Yousuf, M., Das, T. & Adhikari, S. Palladium catalyzed decarboxylative acylation of arylboronic acid with ethyl cyanoacetate as a new acylating agent: synthesis of alkyl aryl ketones. *New J. Chem.* **39**, 8763–8770 (2015).
6. Himo, F. *et al.* Copper(I)-catalyzed synthesis of azoles. DFT study predicts unprecedented reactivity and intermediates. *J. Am. Chem. Soc.* **127**, 210–216 (2005).
7. Zhu, C. *et al.* A multicomponent synthesis of stereodefined olefins via nickel catalysis and single electron/triplet energy transfer. *Nat. Catal.* **2**, 678-687 (2019).
8. Yanai, T., Tew, D. P. & Handy, N. C. A new hybrid exchange–correlation functional using the coulomb-attenuating method (CAM-B3LYP). *Chem. Phys. Lett.* **393**, 51-57 (2004).
9. Frisch, M. J. *et al.* Gaussian 16, revision C.01 (Gaussian, 2016).
10. Legault, C. Y. *CYLview 1.0b* (Unversité de Sherbrooke, 2009); http://www. cylview.org.
